# Differences in Phage Recognition and Immunogenicity Contribute to Divergent Human Immune Responses to *Escherichia coli* and *Klebsiella pneumoniae* Phages

**DOI:** 10.1002/eji.202451543

**Published:** 2025-03-12

**Authors:** Huu Thanh Le, Carola Venturini, Alicia Fajardo Lubian, Bethany Bowring, Jonathan Iredell, Jacob George, Golo Ahlenstiel, Scott A. Read

**Affiliations:** ^1^ Blacktown Clinical School Western Sydney University Sydney NSW Australia; ^2^ Storr Liver Centre Westmead Institute for Medical Research Sydney NSW Australia; ^3^ Centre for Infectious Diseases and Microbiology (CIDM) Westmead Institute for Medical Research Sydney NSW Australia; ^4^ Sydney School of Veterinary Science, Faculty of Science University of Sydney Sydney NSW Australia; ^5^ Faculty of Medicine and Health University of Sydney Sydney NSW Australia; ^6^ Department of Hepatology and Gastroenterology Westmead Hospital Sydney NSW Australia; ^7^ Blacktown Mt Druitt Hospital Sydney NSW Australia

**Keywords:** bacteriophage, inflammation innate immunity, phage immunity, phage therapy

## Abstract

Bacteriophages (phages) are emerging as a viable adjunct to antibiotics for the treatment of multidrug‐resistant (MDR) bacterial infections. While intravenous phage therapy has proven successful in many cases, clinical outcomes remain uncertain due to a limited understanding of host response to phages. In this study, we conducted a comprehensive examination of the interaction between clinical‐grade phages used to treat MDR *Escherichia coli* and *Klebsiella pneumoniae* infections, and human peripheral blood immune cells. Using whole transcriptome as well as proteomic approaches, we identified a strong inflammatory response to *E. coli* phage vB_EcoM‐JIPh_Ec70 (herein, JIPh_Ec70) that was absent upon exposure to *K. pneumoniae* phage JIPh_Kp127. We confirmed that JIPh_Ec70's DNA recognition by the STING pathway was principally responsible for the activation of NF‐kB and the subsequent inflammatory response. We further show that monocytes and neutrophils play a dominant role in phage uptake, primarily through complement‐mediated phagocytosis. Significant differences in complement‐mediated phagocytosis of JIPh_Kp127 and JIPh_Ec70 were observed, suggesting that reduced recognition, phagocytosis, and immunogenicity all contribute to the significantly decreased response to JIPh_Kp127. Our findings contribute to the progress of our understanding of the innate immune response to therapeutic phages and offer potential insights into how to improve the safety and effectiveness of phage therapy.

## Graphical abstract

Clinical grade JIPh_Ec70 phages but not JIPh_Kp127 phages elicit a potent inflammatory response in peripheral immune cells. JIPh_Ec70 phagocytic engulfment is facilitated by complement opsonization, resulting in STING activation by phage DNA, driving an inflammatory signaling cascade. Understanding phage immunogenicity will be a key factor in developing effective phage therapies in the coming years.

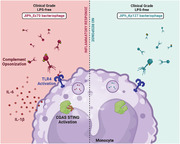

## 1 Introduction

Bacteriophages (phages), viruses that exclusively infect bacteria, are rapidly emerging as a novel therapeutic adjunct to antibiotics for treating multidrug‐resistant (MDR) bacterial infections, the cause of more than 1.2 million deaths a year [1]. Thus far, phage therapy has only received approval for compassionate use, typically in conjunction with ongoing antibiotic treatment [2, 3, 4, 5, 6]. Nevertheless, promising indications have been reported against several MDR bacterial infections [4, 5, 7]. The intravenous injection of low‐endotoxin phage preparations has become the preferred and most effective administration method for phage therapy, as only a small proportion of orally administered phages reach circulation [8, 9]. Effective therapeutic concentrations of 10^9^–10^11^ plaque‐forming units (pfu) introduced intravenously have raised concerns regarding the potential immune recognition of phage particles and the underlying safety of phage therapy [10].

A diverse body of work has examined the immune response to phages, in clinical cases, animal experiments, and in vitro [4, 5, 7, 11, 12, 13, 14]. In humans, up to 99% of therapeutic phages administered intravenously disappear from circulation within an hour, suggesting an extremely efficient method of phage sequestration in vivo [7]. Mouse studies have shown that the highest concentration of intravenously administered phages can be found in the liver and spleen 24 h after injection, suggesting that resident macrophages and endothelial cells at these sites are primarily responsible for phage clearance [15, 16, 17]. While phage‐targeting antibodies have been detected in the weeks following phage treatments, consistent with the development of adaptive immunity [18, 19], reports on the innate immune responses to different phage species are inconsistent [11, 12, 13, 20]. In vitro, treatment with the myovirus T4 *Escherichia coli* phage does not generate inflammatory or antiviral responses in human peripheral blood mononuclear cells (PBMCs) [11], but *Staphylococcus aureus* and *Pseudomonas aeruginosa* tailed phages regardless of morphology, produce potent pro‐ and anti‐inflammatory responses [12]. *P. aeruginosa* filamentous phages and *E. coli* phage cocktails have also been shown to stimulate interferon (IFN) responses in murine models [13, 20]. Together, these studies indicate that phages are recognized and elicit an immune response but cannot establish whether these differences in the immune response can be attributed to differences in phage preparation, inherent phage immunogenicity (genomic material, immunogenic proteins), or host‐specific memory responses based on previous exposure.

Here, we investigated the phage‐specific innate immune response of peripheral blood circulating immune cells using purified endotoxin‐depleted phages from different families targeting MDR *E. coli* and *Klebsiella pneumoniae* strains. To our knowledge, this is the first study using suitable controls for bacterial immunogen contamination. We identified a phage‐specific proinflammatory response driven primarily by STING recognition of phage DNA. We also confirmed that monocytes and neutrophils are the main drivers of phage clearance via phagocytosis, aided by complement factor opsonization. These findings represent a significant step toward developing safer, more effective phage therapy.

## 2 Results

### 2.1 Phage‐Specific Innate Response to JIPh_Ec70 and JIPh_Kp127

To assess phage‐specific peripheral immune responses, two phages were chosen: vB_EcoM‐JIPh_Ec70 (SRA PRJNA764821; herein, JIPh_Ec70; Herelleviridae; myovirus) and JIPh_Kp127 (GenBank MN434096; Demerecviridae; syphovirus), targeting *E. coli* and *K. pneumoniae* clinical isolates, respectively. Following purification, the concentration of both phages ranged from 10^10^–10^12^ pfu, and endotoxin concentration was <0.5 EU/10^7^ pfu/mL (Table S1).

**FIGURE 1:**
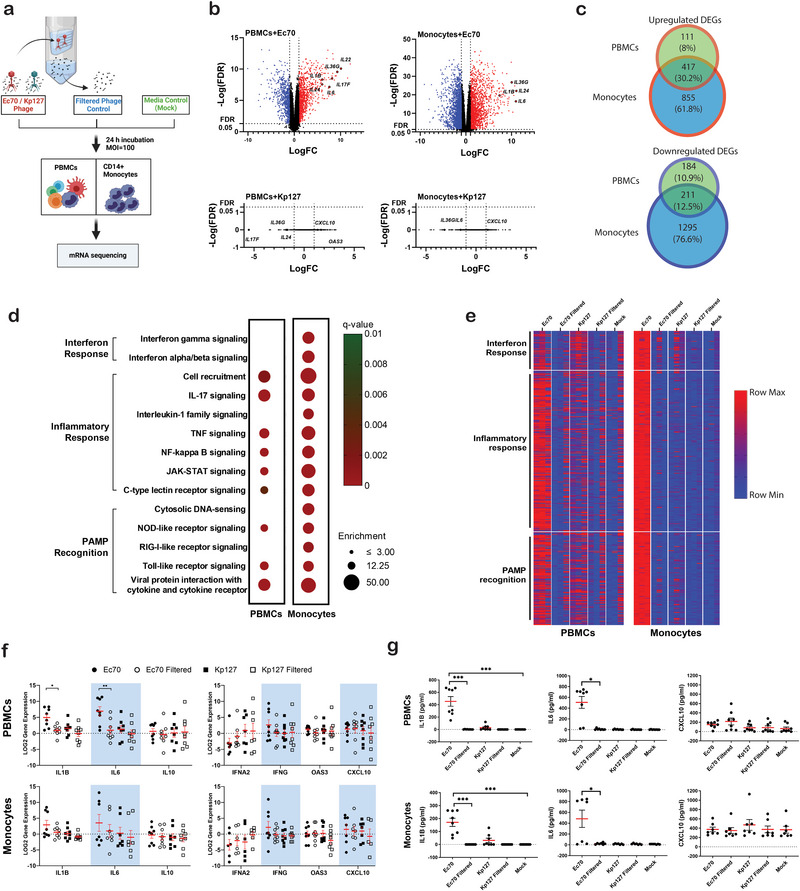
Unique peripheral blood immune cell responses to phages JIPh_Ec70 and JIPh_Kp127. (A) Diagram outlining PBMC and monocyte phage treatments and controls prior to RNA sequencing. Filtered phage controls contained no viable phage but bacterial contaminants, if any, were able to pass through 100 kDa filters. (B) Volcano plot of RNA sequencing data showing upregulated DEGs (red), downregulated DEGs (blue), and nondifferentially expressed genes (black) in PBMCs and monocytes treated with either JIPh_Ec70 or JIPh_Kp127 (*n = 3*). (C) Overlapping upregulated DEGs in PBMCs and monocytes treated with JIPh_Ec70. (D) Enriched pathway analysis of upregulated DEGs in PBMCs and monocytes treated with JIPh_Ec70. (E) Relative expression of the pathway‐enriched DEGs across all samples treated with either phages or controls. (F) Relative expression of selective proinflammatory cytokine genes (*IL1B*, *IL6*), anti‐inflammatory cytokine gene (*IL10*), and interferon response‐related genes (*IFNA2*, *IFNG*, *OAS3*, and *CXCL10*) in PBMCs and monocytes from eight donors treated with either JIPh_Ec70 or JIPh_Kp127. Paired‐t test, *p < 0.05, **p < 0.01. (G) Concentration of secreted proinflammatory cytokines (IL‐1β and IL‐6) and interferon‐stimulate cytokine (CXCL10) in PBMC and monocytes treated with either JIPh_Ec70, JIPh_Kp127 or controls (*n = 8*). Friedman test, *p < 0.05, ***p < 0.005. For (F, G), data presented with mean ± standard error of the mean (SEM). Ec70 = JIPh_Ec70, Kp127 = JIPh_Kp127. DEG, differentially expressed gene; FDR, false discovery rate; LogFC, log fold change; PBMC, peripheral blood mononuclear cell.

To investigate the immune response to JIPh_Ec70 and JIPh_Kp127, 5 × 10^7^ pfu of these phages were applied to either 5 × 10^5^ PBMCs or CD14^+^ monocytes isolated from healthy individuals for 24 h (*n* = 3/treatment) (Figure 1A). To confirm that phage preparations did not contain immunogenic bacterial components, we prepared filtered controls using a 100 kDa Amicon filter. This control did not contain viable phage particles but preserved immunogenic bacterial components if present. Poly‐A mRNA sequencing was performed and sequenced short reads were aligned to the Ensembl build 98 reference genome (GRCh38) resulting in 16,070 genes with detectable reads. Paired gene expression comparisons were performed between media‐only control, phage‐filtered controls, and phage treatments to define differentially expressed genes (DEGs; Table S2).

Prior to assessing the transcriptional response to phages, we confirmed that our preparations themselves did not contain any immunogenic bacterial components by comparing filtered‐phage controls and media‐only controls (Table S2). There was no significant difference in gene expression between these treatments, suggesting that our phage purification method removed bacterial contaminants and that any change in gene expression following phage treatment was indeed phage‐specific.

PBMC treatment with JIPh_Ec70 for 24 h resulted in 923 and 2778 DEGs in PBMCs and monocytes, respectively, while there was no significant transcriptional dysregulation following JIPh_Kp127 treatment (Figure 1B, Table S2). Included in the upregulated genes stimulated by JIPh_Ec70 (528 in PBMC and 1272 in monocytes) were pro‐inflammatory cytokines such as *IL1B*, *IL36G*, *IL17F*, *IL6* as well as wound healing‐related cytokines such as *IL22*, *IL24* (Full list in Supporting Information File S1). A majority of DEGs in PBMCs treated with JIPh_Ec70 were also identified in monocytes (Figure 1C), suggesting a significant contribution of the innate immune cells to the overall response against the phage. Multiple interferon‐stimulated genes (e.g., *OAS3*, *CXCL10*) increased following JIPh_Kp127 treatment, but not significantly (FDR > 0.05) (Figure 1B). Interestingly, some inflammatory cytokine genes (e.g., *IL6*, *IL36G*) induced by JIPh_Ec70 were suppressed in cells treated with JIPh_Kp127 (log_2_(FC) < −2, FDR > 0.05) (Figure 1B).

Pathway‐enrichment analysis of JIPh_Ec70 upregulated genes revealed numerous innate immune response pathways that could be largely categorized into interferon response, inflammatory response, and recognition of pathogen‐associated molecular patterns (Figure 1D). Relative expression of upregulated genes belonging to these pathways in all samples indicated the highest expression in the cells treated with JIPh_Ec70 compared with JIPh_Kp127 and control groups (Figure 1E).

To confirm transcriptional profiling by RNA‐Seq, qPCR and ELISA were employed to measure the pro/anti‐inflammatory (IL1B, IL6, IL10) and interferon response (IFNA2, IFNG, OAS3, and CXCL10) (Figure 1F). These data agreed with RNA‐Seq results, demonstrating an increase in *IL1B* and *IL6* transcript expression (Figure 1F) and protein secretion (Figure 1G) in both PBMCs and monocytes treated with JIPh_Ec70. Conversely, there were no significant differences in the expression of the anti‐inflammatory gene (*IL10*) and interferon‐related genes in immune cells treated with JIPh_Ec70 in comparison to those treated with JIPh_Kp127 or any of the control groups.

### 2.2 JIPh_Ec70 DNA Activates Inflammatory Signaling via Activation of STING

**FIGURE 2:**
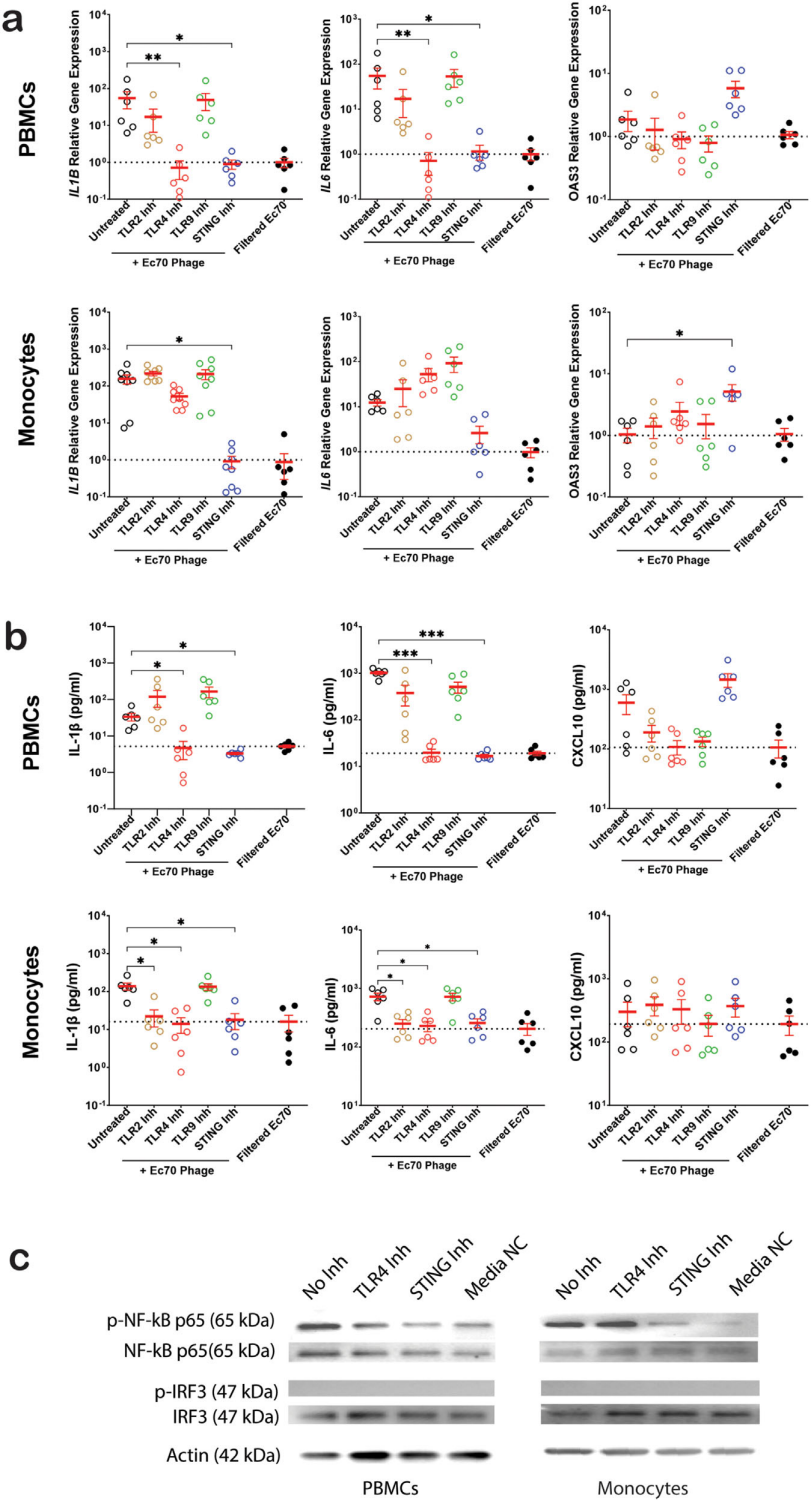
Interrogation of the JIPh_Ec70‐specific inflammatory response. (A) Expression of *IL1B, IL6*, and *OAS3* in PBMCs and monocytes treated with JIPh_Ec70 phages with/without pretreatment with PPRs inhibitors, measured by qPCR (*n* = 8). (B) The concentration of IL‐1β, Il‐6, and CXCL10 in cell media after treatment with JIPh_Ec70 phages with/without pretreatment with PPRs inhibitors measure by ELISA (*n* = 6). (C) Western blot showing NF‐kβ p65 and IRF3 and their phosphorylated forms in PBMCs and monocytes treated with JIPh_Ec70 phages with/without pretreatment with TLR4 and STING inhibitor. Data presented with mean ± SEM, Friedman test, *p < 0.05, **p < 0.01 ***p < 0.005. Inh, inhibitor; kDa, kilodalton; PBMC, peripheral blood mononuclear cell.

To identify the mechanism by which JIPh_Ec70 stimulates the inflammatory cascade, we next investigated a subset of pattern recognition receptors (PRRs) that have been shown to recognize the immunogenic components of viruses, including tailed phages: TLR2 [21] and TLR4 [22] recognition of human viral capsid proteins, and TLR9 [20] and STING [14] recognition of phage DNA. PBMCs and monocytes were incubated with validated PRR inhibitors (Table S3, Figure S1) before JIPh_Ec70 phage treatment, and IL1B and IL6 levels were measured by qPCR and ELISA (Figure 2A,B). OAS3 and CXCL10 were also detected by qPCR and ELISA, respectively, as markers of the interferon response. Both TLR4 and STING inhibitors abolished the expression of IL1B and IL6 in PBMCs while the STING inhibitor alone was sufficient to inhibit IL1B and IL6 expression. STING inhibition also resulted in a small but significant increase in OAS3.

We next tested the inflammatory signaling cascades induced by JIPh_Ec70 upon recognition by STING or TLR4, which rely on NF‐κB and IRF3 to initiate inflammatory and interferon‐based responses, respectively. JIPh_Ec70 phage treatment for 2 h stimulated the phosphorylation of NF‐κB p65 but not of IRF3 in both PBMCs and monocytes (Figure 2C). Inhibition of STING, and to a lesser extent TLR4, reduced the phosphorylation of NF‐κB p65 in both PBMCs and monocytes (Figure 2C), suggesting that JIPh_Ec70‐mediated STING activation drives NF‐κB phosphorylation and subsequent inflammatory cytokine expression in peripheral blood.

**FIGURE 3:**
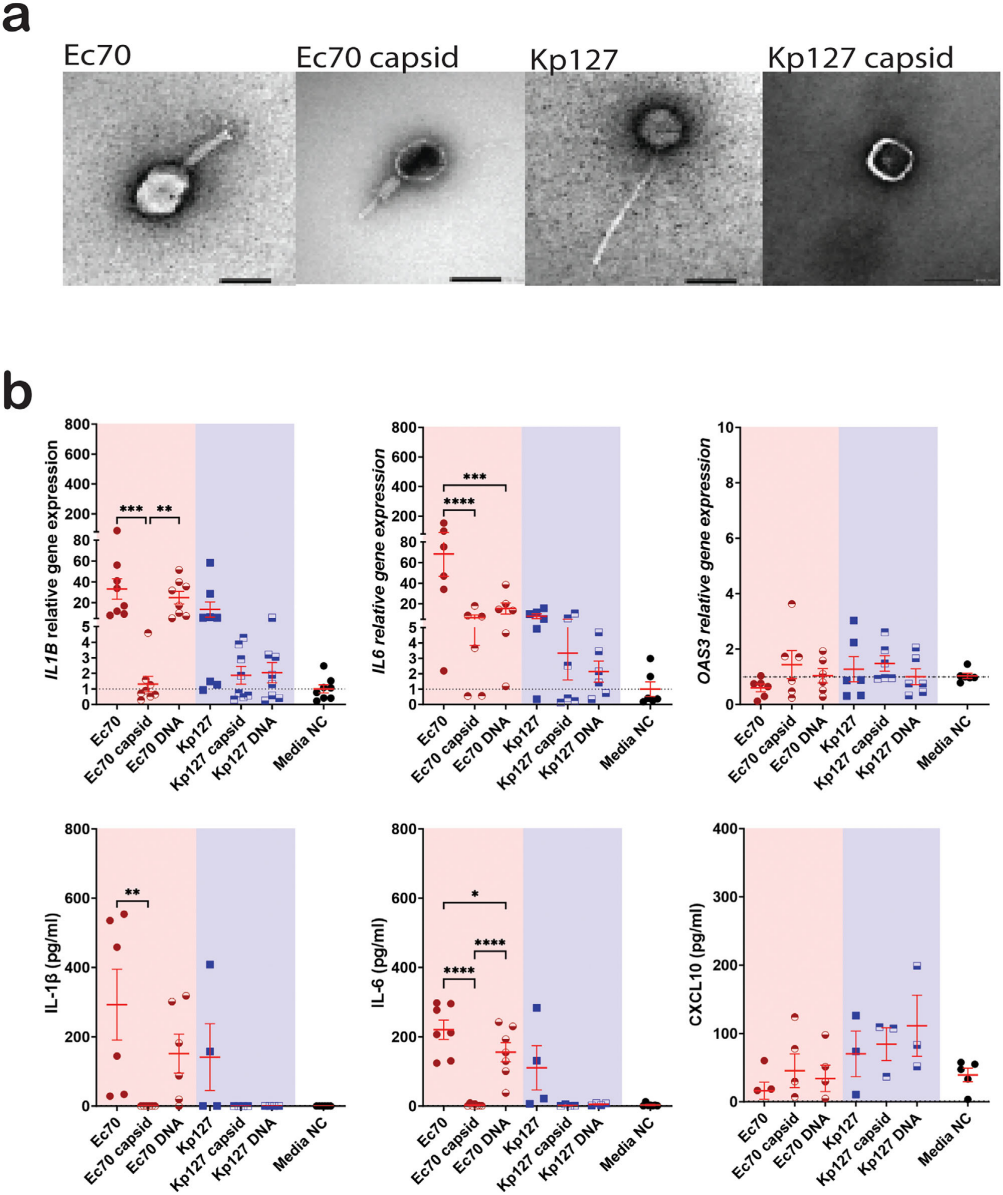
JIPh_Ec70 DNA stimulates an inflammatory response. (A) EM images of intact phages and empty capsids (scale bar 50 nm). (E) Expression of *IL‐1B, IL6*, and *OAS3* in PBMCs treated with intact JIPh_Ec70 or JIPh_Kp127 phages, corresponding empty capsids, and phage DNA (*n* = 6). (B) Relative PBMC gene expression of *IL1B*, *IL6*, and *OAS3*, and concentration of IL‐1β, Il‐6, and CXCL10 in cell media after treatment with intact phages, empty phage capsid, and phage DNA (*n* = 6–8). Data presented with mean ± SEM, Friedman test, *p < 0.05, **p < 0.01 ***p < 0.005. NC, negative control.

To differentiate between inflammatory responses to phage proteins versus phage DNA, we separated phage DNA from its capsid casing and incubated 5 × 10^5^ PBMCs with 5 × 10^7^ whole phages, or empty capsid shells/phage DNA extracted from 5 × 10^7^ phages for 24 h (Figure 3A). The expression of inflammatory cytokines IL1B, IL6, and OAS3/CXCL10 were examined using qPCR and ELISA. JIPh_Ec70 DNA stimulated a similar upregulation of IL1B and IL6 as intact JIPh_Ec70 phage, whereas the empty capsid did not enact a response (Figure 3B). DNAse treatment of JiPh_Ec70 DNA reduced *IL6* and *IL1B* expression to near baseline levels (Figure S2), indicating that DNA was indeed the inflammatory stimulus. As expected, neither the JIPh_Kp127 phage nor its individual components (empty capsid and core DNA) stimulated IL1B or IL6. No stimulation of interferon‐based pathways, as measured by *OAS3*/CXCL10, was observed.

### 2.3 Differences in Phage Engulfment by Peripheral Blood Mononuclear Cells in Vitro

**FIGURE 4:**
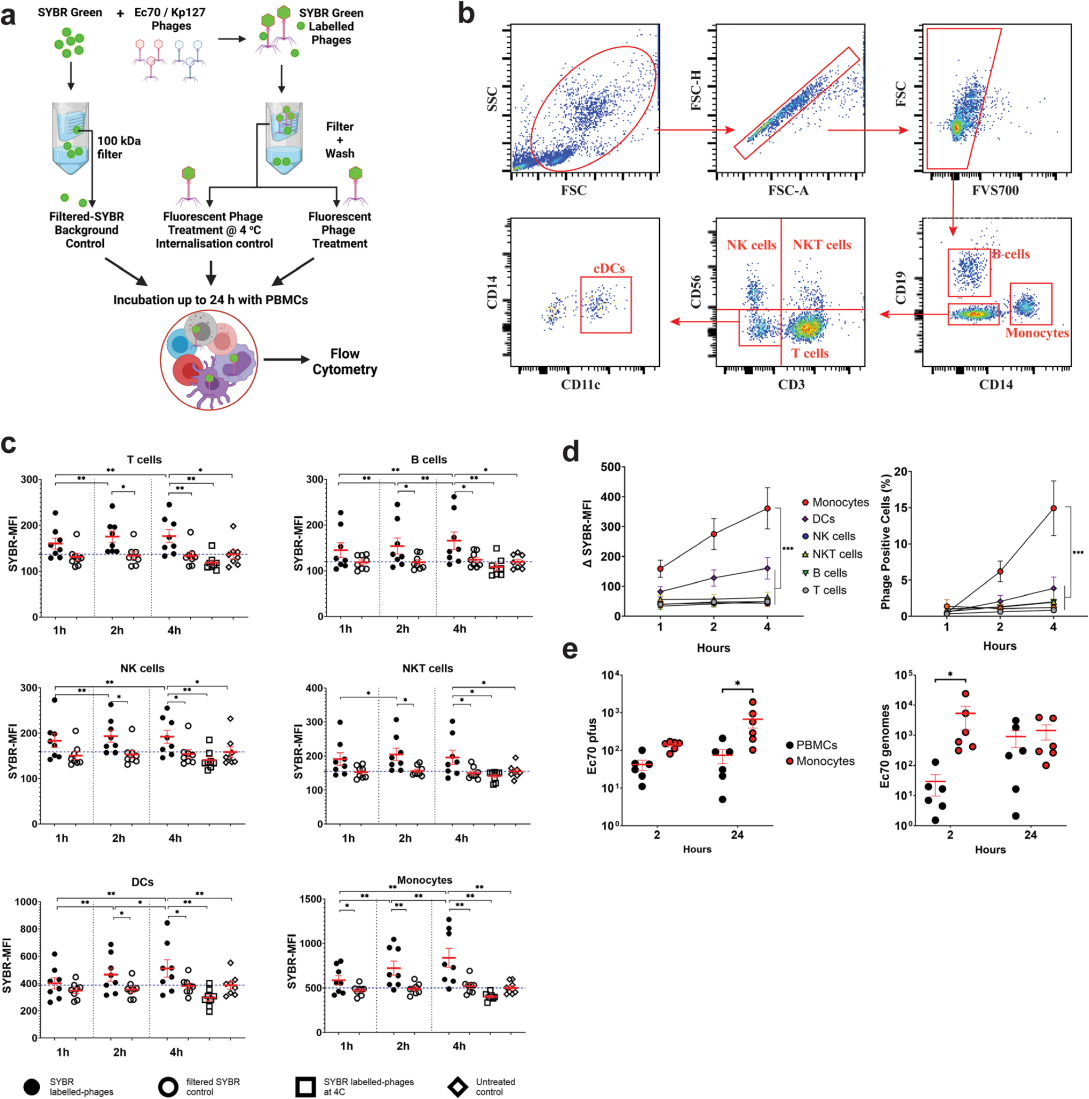
Phage internalization by peripheral blood mononuclear cells is dominated by monocytes. (A) Diagram of PBMC phage internalization assay outlining phage labeling treatment and filtered SYBR control. (B) Gating strategies used to identify broad PBMC populations. (C) Median fluorescent intensity of SYBR of PBMC populations, representing phage engulfment over a 4 h time‐course (*n* = 8). (D) Δ SYBR‐MFI (cell‐specific phage treated MFI—filtered SYBR MFI) and percentage of phage‐SYBR positive cells in different cell types of PBMCs (*n* = 8). (E) Titer of internalized JIPh_Ec70 and a number of the internalized phage genomes in PBMCs versus monocytes (*n* = 7). (C, D) data presented with mean ± SEM, Friedman test, (E) data presented with mean ± SEM, paired *t*‐test, *p < 0.05, **p < 0.01 ***p < 0.001. DC, dendritic cell; MFI, median fluorescent intensity; pfu, plaque‐forming unit; PBMC, peripheral blood mononuclear cell.

To track phage internalization, phages were fluorescently labeled with SYBR‐green, a dye that does not affect phage structure or viability [23]. To ensure that the fluorescent signals obtained were not due to the carryover of fluorescent dye, filtered‐SYBR controls were prepared with SYBR‐green labeling in the absence of phage substrate, followed by extensive washing. As an additional negative control, PBMCs were incubated with phages at 4°C to inhibit virus internalization (Figure 4A) [24]. After 1,2 and 4 h, engulfment was measured by flow cytometry among broad leukocyte populations: B cells (CD19^+^), monocytes (CD14^+^), T cells (CD3^+^), natural killer (NK) cells (CD3‐CD56^+^), NKT cells (CD3^+^CD56^+^) and conventional dendritic cells (DCs, CD14‐CD11c^+^) (Figure 4B). Flow cytometry gating of phage‐positive cells was performed using the filtered SYBR control as a negative cutoff to account for SYBR carryover (Figure S3). All cell types were capable of internalizing phages over the course of 4 h, as measured by SYBR green median fluorescence intensity (SYBR‐MFI), with significantly different capacities and rates of engulfment (Figure 4C). Importantly, only monocytes and DCs showed a significant increase in SYBR‐green intensity over the time course, with increased SYBR MFI and percentage of phage engulfing cells (Figure 4D). These findings suggest that monocytes and DCs actively engulfed phages throughout 4 h in contrast to lymphocyte populations where phage fluorescence remained constant and low.

To compare the number of internalized phages in monocytes versus total PBMCs, we incubated 10^7^ pfu/mL JIPh_Ec70 with 10^5^ monocytes or PBMCs for 2 and 24 h. Phage genomes and viable intracellular phages were quantified by qPCR and double agar plaque assays, respectively. As expected, monocytes exhibited a greater capacity to engulf phages compared with PBMCs at both time points (Figure 4E). Quantification of phage genomes identified up to a log‐fold increase in phage DNA compared with viable phage among both populations, indicating that internalized phages lost infectivity following engulfment.

### 2.4 Complement Factors Mediate Bacteriophage Phagocytosis by Monocytes and Neutrophils

**FIGURE 5:**
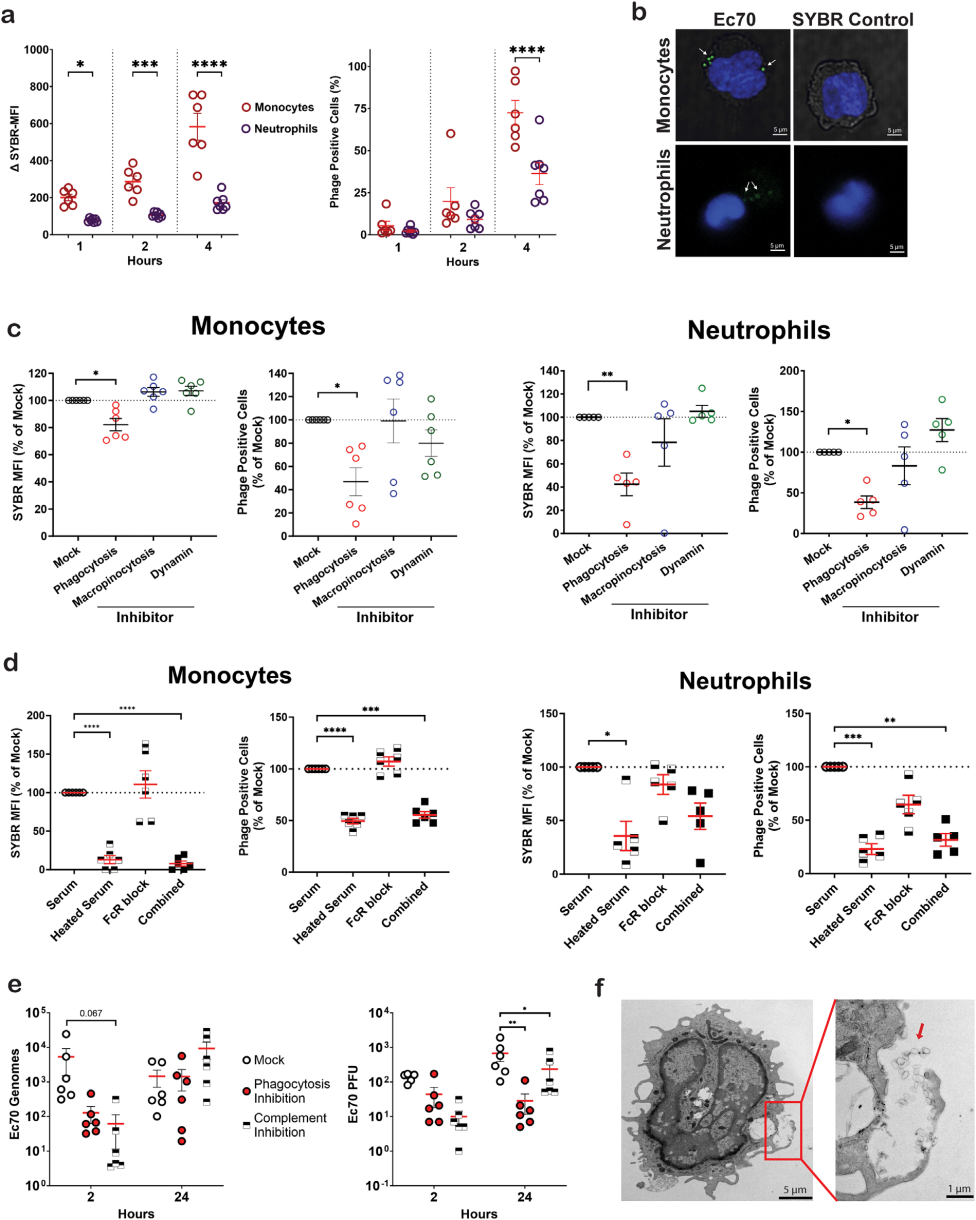
Phage internalization pathways in monocytes and neutrophils. (A) SYBR‐labeled JIPh_Ec70 internalization in isolated neutrophils and monocytes (*n* = 7). (B) Fluorescent images of internalized SYBR‐labelled JIPh_Ec70 phages (green) in live monocytes (DIC image in grey) and neutrophils (nuclear counterstained with Hoechst). (C) SYBR‐labeled JIPh_Ec70 internalization in monocytes (*n = 6*) and neutrophils (*n = 5*) following inhibition of engulfment pathways specific to phagocytosis, macropinocytosis, or dynamin‐dependent endocytosis. (D) SYBR‐labeled JIPh_Ec70 internalization in monocytes (*n = 7*) and neutrophils (*n = 5*) cultured in media supplemented with either heated serum to inactivate complement, pretreated with FcR block to inhibit antibody opsonization, or both. (E) Titer and relative genome content from monocyte internalized JIPh_Ec70 phages following inhibition of phagocytosis or complement. (F) Electron micrographs of monocytes treated with SYBR‐labeled JIPh_Ec70 phages, demonstrating phage aggregates (red arrow) within an endosomal compartment (red square). (A, B) Data presented with mean ± SEM, paired *t*‐test, and in (C–E), data presented with mean ± SEM, RM one‐way ANOVA test, *p < 0.05, **p < 0.01 ***p < 0.001, ****p < 0.0001. FcR, Fc receptor; MFI, median fluorescent intensity; PFU, plaque‐forming unit.

As neutrophils are absent from PBMC preparations, we next investigated phage uptake by neutrophils; polymorphonuclear phagocytes that outnumber monocytes in blood by approximately 10:1. Equal numbers of >95% pure neutrophils or monocytes were incubated with SYBR‐labelled phages or filtered SYBR‐green controls for 4 h. Compared with monocytes, neutrophils exhibited lower phage engulfment and a slower rate of uptake (Figure 5A). After 4 h, approximately 60% of monocytes compared with 30% of neutrophils were phage positive. Phage uptake was considerably higher in pure monocytes compared with monocytes in PBMCs (16%, Figure 4D), likely owing to increased contact and recognition of phage particles in pure cultures. Fluorescent imaging confirmed the internalization of SYBR‐labelled JIPh_Ec70 in both monocytes and neutrophils (Figure 5B), demonstrating what appears to be vacuolar aggregates of fluorescent particles in both cell types.

To identify the mechanisms of phage engulfment, we used inhibitors of dynamin‐dependent clathrin and caveolae‐mediated internalization pathways (Dynasore) [25], as well as inhibitors of macropinocytosis (EIPA) [26] and phagocytosis (Cytochalasin D) [27, 28]. Confirmation of pathway inhibition and cell viability was performed using fluorescence‐tagged control cargos specific to each pathway (Figure S4). Inhibition of phagocytosis alone significantly reduced phage engulfment by monocytes and neutrophils, as measured by SYBR‐MFI and the proportion of phage‐engulfing cells (Figure 5C).

To investigate two well‐characterized opsonins, antibodies, and complement factors, JIPh_Ec70 was incubated with media containing pooled serum from eight donors for 30 min and then added to monocyte and neutrophil cultures. To inactivate complement, serum was heat‐treated [29], whereas Fc blocking reagent was used to inhibit antibody‐mediated opsonization [30]. Inactivating complement factors alone significantly reduced SYBR intensity and phage‐positive cells in both monocytes and neutrophils (Figure 5D), while Fc receptor (FcR) block only modestly reduced phage uptake by neutrophils (*p* > 0.05).

To further assess complement opsonized phagocytosis as a mechanism of phage engulfment, we quantified intracellular phage DNA and infective phage at 2 and 24 h post‐JIPh_Ec70 treatment (Figure 5E). As expected, we observed a significant reduction in internalized viable phages and phage genomes in monocytes treated with either cytochalasin D or heated serum at 2 h. After 24 h, both treatments stimulated a significant reduction in intracellular phage, in contrast to intracellular phage genomes that were unaffected by either treatment. Electron microscopy of monocytes incubated with phages for 2 h indicated the presence of large endosomes enclosing phage aggregates, suggesting that complement‐mediated aggregation can facilitate monocyte phagocytosis (Figure 5F).

### 2.5 Increased Monocyte Engulfment of JIPh_Ec70 versus JIPh_Kp127 Is Not Dependent on Complement

**FIGURE 6:**
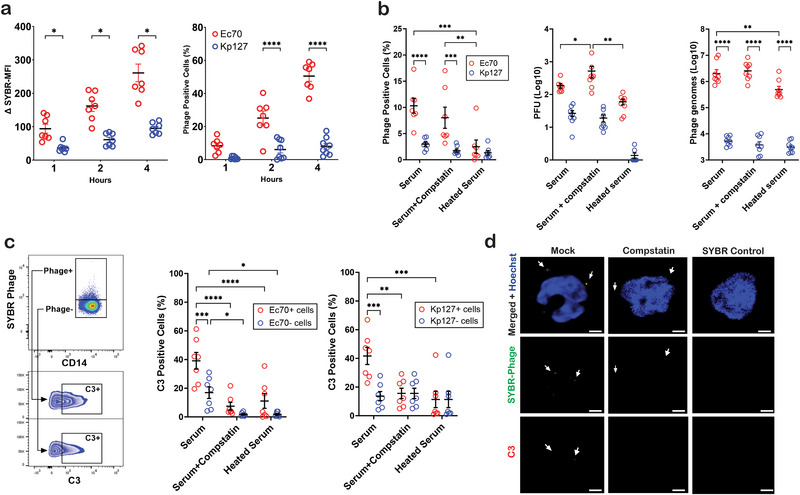
The Role of Complement in JIPh_Ec70 and JIPh_Kp127 Internalisation. (A) Comparison of SYBR‐labelled JIPh_Ec70 and JIPh_Kp127 monocyte internalization over a 4 h time‐course (*n* = 6). (B) Ec70 and Kp127 phage internalization following inhibition of complement using compstatin (C3 inhibitor) or heat inactivation. Intracellular phage was measured by flow cytometry (SYBR labeling), plaque‐forming units (viable phage), and qPCR (intracellular genomes) (*n = 8*). (C) C3 internalization was quantified by flow cytometry in phage+ and phage‐monocytes following complement inactivation using compstatin and heat inactivation (*n = 7*). (D) Immunofluorescent imaging of monocytes treated with SYBR‐labelled phages (green) ± C3 inhibitor compstatin. Internalized C3 was labelled in red and cell nucleus in blue. Detected phages and C3 marked with a white arrow, scale bar = 2 µm. Data presented with mean ± SEM, a paired *t*‐test, (E, F) RM two‐way ANOVA test, *p < 0.05, **p < 0.01 ***p < 0.001, ****p < 0.0001. C3, complement factor 3; MFI, median fluorescent intensity; PFU, plaque‐forming unit.

We next compared JIPh_Ec70 and JIPh_Kp127 monocyte internalization using equivalent amounts of SYBR‐labelled JIPh_Ec70 and JIPh_Kp127. JIPh_Ec70 phages were internalized faster and to a greater degree compared to JIPh_Kp127 phages (Figure 6A).

To determine whether complement factor C3, a critical opsonin that stimulates pathogen recognition and engulfment, plays a significantly crucial role in phage phagocytosis, we inhibited C3 using compstatin [31, 32]. Monocytes were treated with SYBR‐labelled JIPh_Ec70 or JIPh_Kp127 in media containing pooled human serum, alone or in combination with compstatin, or media containing heat‐inactivated serum for 4 h (Figure 6B). Intracellular phage was quantified by flow cytometry, plaque‐forming assay (viable phage), and qPCR (phage DNA). While compstatin treatment marginally reduced phage uptake as measured by flow cytometry, it increased viable intracellular phage (pfu), suggesting that C3 may be involved in intracellular phage degradation. Heat‐inactivated serum significantly reduced JIPh_Ec70 uptake, with a modest reduction in JIPh_Kp127 as well.

C3 was next measured alongside fluorescent phage by flow cytometry to determine its role in phage binding, and uptake, as well as the effects of compstatin and heated serum on phage‐C3 binding. As expected, both JIPh_Ec70 and JIPh_Kp127‐containing cells possessed significantly higher C3 positivity, suggesting that C3 binds phage (Figure 6C). There was, however, no significant difference in C3 binding between phages. Confirming their inhibition of complement activity, both compstatin and heat‐inactivated serum reduced C3 uptake, particularly in phage‐positive monocytes. Internalization of C3 bound phage was confirmed by immunofluorescent imaging, demonstrating co‐localization of SYBR‐labelled phages with C3 in cells treated with normal serum only; a co‐localization absent following the addition of compstatin (Figure 6D).

## 3 Discussion

In this study, we examined phages JIPh_Ec70 and JIPh_Kp127 targeting the clinically relevant pathogens *E. coli* and *K. pneumoniae*, ranking 1st and 3rd in global deaths attributed to antibiotic‐resistant bacteria, respectively [33], placing them at the top of the list of serious pathogenic threats [34, 35]. As traditional antibiotics become less reliable against these highly MDR bacteria [36], phage therapy is seen as a viable, potentially life‐saving, adjunct in the treatment of refractory infections [37, 38]. Nonetheless, clinical success is still unpredictable due to a poor understanding of phage‐bacteria interactions and their impact on the human immune system leading to limited applications in critical care [39, 40]. We used phages belonging to different morphological groups (*Herelleviridae myovirus* and *Demerecviridae syphovirus*) to assess the peripheral immune response with a focus on phage recognition, uptake, and response. Phage concentrations used as part of this study are consistent with effective phage doses used clinically that range from 10^9^ to 10^11^ [10]. This dosage would represent approximately 10^5^–10^7^ circulating pfu/ml in the average individual with 5000 mL of blood. Using carefully designed controls to ensure that bacterial immunogens did not contaminate phage‐specific responses, we have demonstrated that these different phages elicit potent but different inflammatory responses based on recognition of phage DNA. Using the immunogenic JIPh_Ec70 myovirus, we demonstrated that monocytes and neutrophils are likely the primary mediators of phage clearance in blood; a process that is facilitated by complement‐assisted phagocytosis. These findings have broad implications regarding phage‐type selection for therapeutic use, suggesting perhaps that phages should be assessed for their immunogenicity as well as their capability to evade clearance by host phagocytes prior to clinical use.

Our study revealed major differences in the inflammatory response to the two different phages, shedding light on the inconsistent findings regarding phage immunogenicity in prior research [7, 11, 12, 13, 20, 40]. To our knowledge, our study is the first to include necessary phage filtrate controls to ensure that the carryover of bacterial immunogens from the phage preparation is not responsible for initiating immune responses. While LPS is measured in most studies, other bacterial immunogens that can contaminate phage preparations such as flagellin, peptidoglycan, or bacterial genomic material are generally not accounted for. Though this aspect renders comparison of our findings with those of other studies difficult, consistent with the inflammatory response to JIPh_Ec70, *S. aureus* and *P. aeruginosa* phages have been shown to elicit inflammatory (*IL1B, IL6*) as well anti‐inflammatory gene expression profiles in human PBMCs [12]. We observed a far greater inflammatory response to JIPh_Ec70 characterized by significantly stronger induction of inflammatory genes (*IL1B, IL6, IFNG*, ∼6‐7 Log_2_FC) as compared with anti‐inflammatory genes (*IL1RN, SOCS3, IL10*, ∼2 Log_2_FC) (Supporting Information File S1). Moreover, we identified high expression of wound‐healing cytokines such as *IL22* and *IL24*, supporting the initiation of inflammatory resolution following acute inflammation triggered by the JIPh_Ec70 phage. In line with the IFN response to JIPH_Ec70 identified in this study, other phages have also been shown to stimulate IFN‐γ‐driven mucosal inflammation in a murine colitis model and IFN‐β antiviral responses in a murine chronic wound model [13, 20]. Gogokhia et al. demonstrated that phage DNA was responsible for IFN‐γ induction through recognition by TLR9 in resident DCs, in contrast to our identification of STING‐based recognition of JIPh_Ec70 DNA, which induced the NF‐kB signaling pathway rather than the interferon‐induced IRF3 pathway. Consistent with our findings, oral phage administration in chickens has been shown to activate “incomplete” cGAS‐STING pathway signaling, halting prior to phosphorylation of NF‐κB or IRF‐3; an interesting finding hypothesized to be caused by a lack of RNA polymerase III binding to phage DNA and thus no production of double‐stranded RNA (dsRNA) necessary for signal propagation [14]. Importantly, TLR9 is abundantly expressed in DCs but not in monocytes [41], while STING is broadly expressed in all immune and nonimmune cell types. As the STING pathway recognizes cytosolic DNA, additional work is needed to understand how JIPh_EC70 DNA enters the cell, and subsequently, the cytosol. Naked DNA is readily taken up into endosomes by myeloid cells [42] and can be facilitated by antimicrobial peptides [43]; however, the mechanism of DNA internalization remains unclear. Intriguingly, bacterial ligands in phagosomes can be transported to the cytosol to activate cytosolic PRRs such as NOD2 [44], providing a possible mechanism for JIPh_Ec70 DNA activation of STING. Notably, we measured no inflammatory response to the JIPh_Ec70 capsid, consistent with previous works on T4 capsid proteins [11].

Interestingly, both TLR4 and STING inhibitors could independently prevent the inflammatory response to Ec70 phage. Having confirmed that these inhibitors do not interfere with one another's pathway activation (Figure S5), these data suggest that activation of both PRRs is necessary to initiate a response. As viral glycoproteins and histones have both been shown to activate TLR4 [45, 46], it is possible that TLR4 can mediate phage recognition and endocytosis to enable STING activation by phage DNA. As STING and TLR4 ligands also demonstrate synergism [47], the combined signal may also be necessary to stimulate an inflammatory response.

Phages have been proposed to enter eukaryotic cells, including immune cells, through macropinocytosis [48, 49], transcytosis [50], and phagocytosis [51]. Importantly, while phagocytosis has often been assumed as a mechanism of phage endocytosis, to our knowledge it has not yet been validated experimentally. Using inhibitors of endocytic pathways, phagocytosis was identified as the primary mechanism of JIPh_Ec70 phage internalization in human immune cells, emphasizing the critical role of complement factors such as opsonins. Importantly, the phagocytosis inhibitor Cytochalasin D has been shown to interfere with actin filament dynamics independent of phagocytosis which may have contributed to the inhibition of phage uptake we observed [52]. While inhibition of phagocytosis and complement opsonization was pronounced in the short term (Figure 5C–E), 24 h quantification of intracellular JIPh_Ec70 genomes demonstrated no difference from untreated controls (Figure 5E). These data suggest that phage internalization may occur bimodally: short‐term receptor‐mediated phagocytosis with the help of complement opsonization, for example [53], and long‐term endocytosis via another nonspecific mechanism such as micropinocytosis [50]. Stronger and faster engulfment of JIPh_Ec70 was observed as compared with JIPh_Kp127; however, there was no difference in complement‐mediated phagocytosis, suggesting that an alternative mechanism is stimulating JIPh_Ec70 recognition and/or uptake.

Examination of phage genomes indicated that JIPh_Ec70 (SRA PRJNA764821) but not JIPh_Kp127 (GenBank MN434096) contained a sequence with high similarity to the T4 phage highly antigenic outer capsid protein Hoc (Supporting Information File S2). Hoc has been shown to elicit potent antibody responses and subsequent complement binding *in vivo* that can inactivate T4 phage infectivity [54], suggesting that a JIPh_Ec70 Hoc‐like protein may facilitate phage opsonization to increase phage uptake. Conversely, T4 phages engineered to express Hoc‐fusion proteins are more quickly inactivated by complement and rapidly disappear from circulation [55]. These data suggest that Hoc may indeed be protective against opsonization; however, it is unclear whether the described fusion proteins increased phage immunogenicity, inhibited potential Hoc functions, or both. In summary, the role of complement, phage‐specific antibodies, and their potential interactions with Hoc or other immunogenic capsid antigens remains largely uncertain. Nonetheless, other studies using T4 capsid proteins demonstrated no induction of inflammatory cytokine secretion by Hoc [11], suggesting that it is not sufficient to drive an inflammatory response, in agreement with our findings.

Importantly, we could not confirm the role of antibody opsonization, measuring a minor but significant effect of FcR blockade in neutrophils. Nonetheless, the role of neutrophils in phage clearance remains unclear, as highlighted by a recent mouse study demonstrating little effect of neutropenia on phage clearance [56]. Importantly, serum samples used in this study were obtained from individuals who worked closely with JPh_Ec70, though no specific detection of JPh_Ec70‐specific antibodies was performed.

Recent studies have also suggested that the extent of phage internalization in various human cell lines depends on phage size, with smaller phages being preferred [48]. JIPh_Kp127 phage has a similar head size to JIPh_Ec70, but its tail is twice as long (Table S1), providing a potential mechanism for steric hindrance of phage engulfment, particularly in the context of complement‐mediated aggregation.

Some critical limitations to this study should be noted for future research. Filtration was used to generate controls with no phage, but these may have contained other potential contaminants generated during phage production such as bacterial cell residues (*e.g*., peptidoglycan, host DNA) that may remain bound to the phage particles and therefore not eliminated during filtering as expected. Differences in phage immunogenicity measured in our study could be a consequence of excess host material bound to JIPh_Ec70 phage but not to JIPh_Kp127. How likely it is for this to occur remains uncertain. Moreover, in vitro phage treatment excludes numerous factors present in the blood, including serum, platelets, and erythrocytes, that could improve or hinder phage recognition and uptake in vivo and alter cell‐phage concentrations and ratios.

In summary, our study demonstrates that JIPh_Ec70 and JIPh_Kp127 phage isolates are phagocytosed in different quantities and elicit unique immune responses. These data support previous works [53] indicating that phage immunogenicity is likely a critical factor affecting phage half‐life in circulation and subsequent antibacterial efficacy, warranting the recommendation to incorporate phage immunogenicity analysis as a part of the existing pipelines for phage discovery and clinical application. This will undoubtedly enhance our understanding of the immune response to phages and aid in developing more effective phage‐based therapies.

## 4 Methods

### 4.1 Phage Production and Purification

Phage and bacterial hosts (listed in Table S1) were selected from our existing collections [57, 58]. Phages vB_EcoM‐JIPh_Ec70 (SRA PRJNA764821; herein, JIPh_Ec70; Herelleviridae; myovirus) and JIPh_Kp127 (GenBank MN434096; Demerecviridae; syphovirus), targeting *E. coli* and *K. pneumoniae*, respectively (Table S1). Phages were amplified using their original isolation hosts *E. coli* JIE3454 (clinical isolate) and *K. pneumoniae* ATCC 13883. Phages were amplified in Lysogeny Broth (LB) at 37°C, 110 rpm shaking for 3 h by co‐incubation with host strains, followed by centrifugation (4700 *g*, 30 min) and filtering (0.22 µm filter). Bacterial DNA and RNA were degraded by adding 1 U/ml DNase I and 10 µg/ml RNase and incubating 37°C for 1 h. Phage filtrates were concentrated by PEG precipitation as described before [57]. Endotoxins were removed by cesium chloride (CsCl) gradient ultracentrifugation at 110,000*g* for 2 h at 4°C and CsCl was consequently removed by dialysis [59]. The resulting purified phages were then transferred to endotoxin‐free tubes and stored at 4°C. Phage titers (as plaque forming units per milliliter, pfu/mL) were measured using the double agar overlay method after every step of phage amplification and purification, as before [57, 60].

Endotoxin concentration was measured using the Pierce Limulus amoebocyte lysate (LAL) Chromogenic Endotoxin Quantitation Kit (Thermofisher, 88282) following the company's instructions. All steps of endotoxin quantitation were carried out in a safety cabinet and endotoxin‐free water was used throughout the procedure. A standard curve was prepared using stock endotoxin and endotoxin‐free water.

### 4.2 Phage DNA and Empty Phage Capsid Preparation

Genomic DNA of JIPh_Ec70 and JIPh_Kp127 was extracted using the Qiagen DNeasy Blood and Tissue Kits. Proteinase K (20 mg/mL) was added to 10^11^ pfu of purified phage in SM buffer (200 mM NaCl_2_, 10 mM MgSO_4_, 50 mM Tris‐HCl, pH 7.5) and incubated for 1.5 h at 56°C to digest phage capsid proteins [61]. Phage DNA was purified following the manufacturer's standard protocol.

Empty capsids of JIPh_Ec70 and JIPh_Kp127 were generated using approximately 10^8^ pfu of phage in 40 µL of SM buffer, incubated with 16 mM EDTA at 60, 70, 75, or 80°C for 1 h (Figure S6) [62]. Following heat treatment, 16 mM CaCl_2_ was added to saturate EDTA for 10 min before treatment with DNase I (10 U/mL). Phage DNA and capsid derived from 5 × 10^7^ phages were applied to PBMCs in SM buffer.

### 4.3 Peripheral Blood Immune Cell Isolation

Peripheral blood was collected from healthy donors in EDTA‐collection tubes. Project ethics (HREC Reference Number: (4192) AU RED HREC /15 WMEAD/11) were approved by Western Sydney Local Health District Human Research Ethics Committee, NSW, Australia. Formal written consent was obtained from all participants. PBMCs were separated by Ficoll‐Paque gradient centrifugation at 400* g* at room temperature for 30 min. Peripheral blood mononuclear cells (PBMCs) were collected and washed twice with cold DPBS up to 50 mL total volume by centrifuging at 400*g*, 4°C for 10 min.

### 4.4 CD14^+^ Monocyte Isolation

CD14^+^ monocytes were isolated from PBMCs using CD14 Microbeads (Miltenyi). PBMCs were resuspended in selection buffer (0.5% FBS and 2 mM EDTA in DPBS) and 20 µL of CD14 Microbeads were added for every 10^7^ PBMCs and incubated for 15 min at 4°C. Cells were then washed with 5 mL of selection buffer by centrifuging at 400*g* for 10 min. Cells were resuspended in 500 µL of selection buffer and magnetic separation was performed using the autoMACS Pro Separator (Miltenyi). After separation, CD14^+^ monocytes were washed with 1 mL of cold DBPS, and purity was assessed (Figure S7).

### 4.5 Neutrophil Isolation

Neutrophils were isolated from peripheral blood using the EasySep Direct Human Neutrophil Isolation Kit. Briefly, 100 µL of RapidSpheres and 100 µL of Isolation Cocktail were added to 2 mL blood in a 5 mL round‐bottom tube. The mixture was gently mixed followed by an incubation at room temperature for 5 min. Next, 2 mL of isolation buffer (DBPS with 1 mM EDTA) was added and mixed by gently pipetting. The tube was then placed in a magnet EasySep (STEMCELL Technology, #18000) for 5 min. The enriched cell suspension was poured into a new 5 mL round‐bottom tube in one continuous motion. 100 µL of RapidSpheres were added to the cell suspension, gently mixed, incubated for another 5 min then placed in the magnet for 5 min. The enriched cells were again transferred to a new tube by one continuous inverting motion. The new tube was then placed in the magnet for 5 min and the final enriched neutrophils were inverted to a new tube in one motion. Neutrophils were washed with 2 mL cold DBPS by centrifuging at 400*g* for 5 min at 4°C followed by purity assessment (Figure S8).

### 4.6 Preparation and Administration of Phages and Controls

Phage‐free controls were prepared by adding phage preparations into culture media (RPMI plus 10% FBS) and filtering through a 100 kDa cut‐off Amicon filtration unit by centrifuging at 4000*g* for 5 min. A control medium was also prepared by filtering culture media through a 100 kDa cut‐off Amicon filtration unit. PBMCs or monocytes (5 × 10^5^ cells) in Falcon round bottom polystyrene tubes were treated with either 5 × 10^7^ pfu of phages in 200 µL of filtered media, 200 µL of phage‐filtered control, or 200 µL of media control and incubated for 24 h. Cells were pelleted by centrifugation (400*g*, 4°C, 5 min) and the supernatant was carefully transferred to a new 1.5 tube and frozen at −80°C for ELISA‐based measurements. Cells were washed with 500 µL of cold DBPS and proceeded to RNA extraction.

### 4.7 Inhibition of Phage Pattern Recognition Receptors

PBMCs or monocytes (5 × 10^5^ cells) were pretreated with either 50 mM C29 (TLR2 inhibitor), 10 mM TAK‐242 (TLR4 inhibitor), 1 g/mL ODN NH‐18 (TLR9 inhibitor), or 1 µM of H‐151 (STING inhibitor) for 15 min in 100 µL serum‐free media prior to the addition of 100 µL of phages or phage‐filter controls in 100 RPMI plus 20% FBS. Cells were incubated for 24 h followed by a collection of media and cells for ELISA and RNA extraction, respectively.

### 4.8 RNA Extraction and Quantitative PCR

Total RNA was extracted from cells using FavorPrep Tissue Total RNA Mini Kit according to the manufacturers’ specifications. RNA concentration was measured using NanoDrop 2000/2000c spectrophotometer (Thermo Scientific). Reverse transcription was performed in 10 µL reaction volumes containing 120 ng sample RNA, 0.5 µl dNTPs (Bioline, # BIO‐39044), 0.5 µL random primers (Meridian Bioscience, #BIO‐38028), 0.5 µL reverse transcriptase (Promega, M170B) and 2 µL of 5X buffer (Promega, #M531A) in 1 h at 37°C. Resulting cDNA was diluted 1:10 prior to PCR amplification. Quantitative polymerase chain reaction (qPCR) was carried out using the CFX384 Touch Real‐Time PCR System (Bio‐Rad) using 5 µL of iTaq Universal SYBR green supermix (Bio‐Rad, #1725120), 0.5 µL of primers of target genes and 4.5 µL of cDNA. 18S was chosen as the reference gene. PCR cycling was set as follows: 1 activation cycle at 95°C for 2 min, 40 cycles of denaturing at 95°C for 5 s, and annealing/extension at 60°C for 10 s followed by melting curve analysis. Primer sets are listed in Table S4. qPCR data were analyzed using CFX Manager software (Bio‐Rad) and Microsoft Excel. Sample relative gene expression was calculated using the ΔCt method.

### 4.9 RNA‐Sequencing

RNA integrity was assessed by electrophoresis on the Agilent TapeStation by the Genomics facility at the Westmead Institute for Medical Research. cDNA library preparation of poly A‐mRNA was conducted by the Genomics facility, Westmead Research Hub using the Illumina Stranded mRNA Prep, Ligation kit with IDT for Illumina RNA UD Indexes Set A following the manufacturer's protocol (Illumina Stranded mRNA Prep Ligation Reference Guide, Illumina). Short‐read sequencing was performed by the Australian Genome Research Facility. The RNA‐sequencing data was processed by the Bioinformatics Facility, Westmead Research Hub. Data integrity and read quality were confirmed before mapping the reads onto the human genome reference (GRCh38) of Ensembl build 98 and counting against gene annotations. DEGs were identified based on criteria of count per million reads (CPM) greater than 1, a change in expression ≥2, and an adjusted *p*‐value (FDR) less than 0.05.

Pathway enrichment analysis was conducted using the ConsensusPathDB‐human database (http://cpdb.molgen.mpg.de/) [63]. Upregulated DEGs were subject to overrepresentation analysis using the Kyoto Encyclopedia of Genes and Genomes (KEGG) and REACTOME pathway reference databases, gene ontology level 4 and 5 categories with a *p*‐value cut‐off of 0.01.

### 4.10 ELISA

IL‐1β, IL‐6, and CXCL10 R&D DuoSet ELISAs were performed according to manufacturer protocols. Duplicate sample and standard absorbance were measured at 450 nm and subtracted to the background absorbance at 540 nm on the SpectraMax iD5 plate reader. Concentrations of target proteins were calculated from 7‐point standard curves.

### 4.11 Flow Cytometry‐Based Analysis of Phage Internalization

Phages were labeled with SYBR Gold Nucleic Acid Gel Stain (Invitrogen) at 2.5X concentrate for 30 min at 37°C according to established methods [48, 50]. Phages and a SYBR control were washed 15 times through Amicon Ultra‐15 100 kDa cut‐off centrifugal filter units (Merk) with 10 mL of DBPS by centrifuging at 4000 RPM for 1 min.

PBMCs, CD14^+^ monocytes, or neutrophils (5 × 10^5^ cells) in culture media (RPMI plus 10% FBS) were incubated with 10^7^ pfu SYBR‐labelled phages or an equivalent volume of filtered‐SYBR or DBPS for 1, 2, or 4 h. Cells were washed with 2 mL cold DBPSs by centrifuging at 400*g* for 5 min. To assess viability, cells were stained with FVS700 (PBMCs and monocytes) or Zombie Aqua dye (neutrophils) at 1:2000 dilution in cold DPBS for 10 min. Cells were washed with 1 mL of FACS buffer (1% FBS in DBPS) before labeling for 20 min with the following antibody cocktails: (1) PBMCs: anti‐CD3_BUV395, anti‐CD56_BUV737, anti‐CD19_APCCy7, anti‐CD14_BV711, anti‐CD11c_PE‐CF594, (2) monocytes: anti‐CD14_BV421, and (3) neutrophils: anti‐CD66b‐AF700. Cells were washed with 2 mL flow buffer and analyzed on a BD Fortessa X‐20 flow cytometer. Flow cytometry antibodies are listed in Table S5.

To investigate phage endocytosis pathways, monocytes or neutrophils were preincubated with either 2 µM Cytochalasin, 2.5 µM EIPA (5‐[N‐ethyl‐N‐isopropyl]‐Amiloride) and 10 µM Dynasore [28, 64] for 15 min in serum‐free media. Cells were then treated with phages in culture media as described earlier. To investigate opsonic factors, the cells were incubated in culture media supplement with either heated serum or full serum plus Fc receptor (FcR) blocking reagent (1%) before SYBR‐labeled phages or controls were added. To investigate the role of complement factor 3 (C3) in phage internalization, monocytes were preincubated with 50 µM compstatin (MedchemExpress, # HY‐P1036) for 15 min in media with serum before treating with phages. Cells were then incubated for 2 h before proceeding to antibody labeling and flow cytometry as previously described.

### 4.12 Quantification of Intracellular Phage Genomes and Plaque Forming Units

PBMCs or monocytes (5 × 10^5^ cells) were incubated with 5 × 10^7^ phages for up to 24 h. Cells were collected by centrifuging at 400*g* for 5 min and washed three times with cold DBPS and resuspended in 100 µL cold DBPS. The cells were lysed by performing 3 freezing‐thaw cycles coupled with vortexing [65]. Phage lysate was used for quantification of internalized phage genomes by qPCR (primer sets listed in Table S2). A 10‐dilution series of phage genome standards were created using purified phages based on phage titer, thus representing “infective genome equivalents”. For plaque forming unit (pfu) quantification, 50 µL phage lysate was added to 400 µL of bacterial LB culture at 0.4 OD and incubated for 10 min before mixing with 4 mL of 0.35% soft LB agar and overlaying on a 1.5% LB agar dish. Agar dishes were incubated for 24 h at 37°C and the number of phage plaques was counted.

### 4.13 Western Blotting

Cells were washed in cold DBPS and lysed with 100 µL RIPA buffer supplemented with protease and phosphatase inhibitors cocktail (Sigma, PPC101). Cells were freeze‐thawed once, followed by 1 min of vortexing to enhance cell lysis. Cell lysates were centrifuged at 12,500 rpm at 4°C for 15 min to remove cell debris. Protein concentrations were measured using the Bradford DC protein assay (Bio‐Rad). Total protein (20 µg) was loaded on 12 % poly‐acrylamide gel run at 90 V for 1.5 h and transferred to PVDF 0.45 µm membrane (Millipore) at 35 V overnight at 4°C. Membranes were incubated with primary antibodies targeting p‐IRF3 (Ser396) (Cell Signalling, 4947), p‐NF‐κB p65 (Ser536) (Cell Signalling, 3033) overnight at 4°C, followed by and HRP‐conjugated secondary antibodies for 2 h. Proteins were visualized using the SuperSignal Western Blot Substrate Bundle, Pico PLUS + trial‐size Femto (Thermofisher, A43840), and the ChemiDoc imaging system (Bio‐Rad). Antibodies were stripped using 0.2 M NaOH for 15 min and re‐incubated with antibodies targeting IRF‐3 (Cell Signalling, 4302), NF‐κB p65 (Cell Signalling, 8242), and beta Actin (Abcam, ab8227) overnight followed by secondary antibody incubation and detection as previously described. Full Western blots are supplied in Figure S9.

### 4.14 Immunofluorescent Microscopy

To assess phage internalization, 5 × 10^5^ monocytes treated with SYBR‐tagged JIPh_Ec70 phages for 2 h were transferred to 35 mm MatTek gridded petri dishes (Mattek, # P35G‐1.5‐14‐CGRD‐D) precoated with Poly‐D‐lysine (Sigma Aldrich, P6407) to ensure adherence. Culture supernatant was aspirated, and cells were fixed with cold 4% paraformaldehyde (Sigma Aldrich, # 158127) for 10 min and permeabilized with 0.3 % Triton X‐100 (Sigma Aldric, #X100) for 10 min at room temperature. Cells were incubated with 1:500 dilution of goat anti‐C3 Abs (Thermo Fisher, # PA1‐29715) for 1 h, and complement factor C3 was detected using 1 µg/mL rabbit anti‐goat AF 546 (Thermo Fisher, #A‐21085) for 30 min. Cell nuclei were stained with a 1:2000 dilution of Hoechst 3342 (Thermo Fisher, #H3570) in 10 min. Cells were visualized using the confocal microscope Leica TCS SP5 at 37°C and 5% CO_2_. The resulting images were processed using Fiji‐ImageJ.

### 4.15 Transelectron Microscopy

Adhered monocytes from immunofluorescent imaging assay were washed with warm DPBS and fixed with 2.5% glutaraldehyde (ProScitech, C16537), washed with 0.1 M cacodylate buffer (ProSciTech, EMS11650) 3 times (10 min each) then fixed with 2% osmium tetroxide (ProSciTech, C011) for 1 h. After washing 3 times with water, samples were dehydrated in 50%, 70%, 95%, and 100% ethanol for 15 min each. Cells were infiltrated with 50% soft T‐Butyl Perbenzoate (TAAB) resin (Emgrid Australia, E030) for 1 h at room temperature then 100% TAAB soft resin 3 times (10 min each). The samples were embedded and cured in TAAB hard resin (Emgrid Australia, E028) at 70°C. The resin block containing target cells (identified using live‐cell fluorescent imaging) was sectioned at 50 nm thickness with ultramicrotome Leica UC6. The cells were visualized with the JEOL 1400 transmission electron microscope.

### 4.16 Statistical Analysis and Data Presentation

qPCR and ELISA data are presented as individual data points, mean, and standard error of the mean (SEM). Paired *t‐tests* were used to compare the mean between the treatment and control groups. Friedman tests were used to compare differences among different treatments and controls. Statistical analysis and graph construction were performed in GraphPad Prism and are described in graphs and graph legends with replicate number (*n*), mean ± SEM, and *p* values (*p < 0.05, **p < 0.01, ***p < 0.005, ****p < 0.0001). *p* < 0.05 was considered statistically significant. The pathway gene set covering percentage and adjusted p‐values for the matched pathways were extracted and visualized using GraphPad Prism 9. Volcano plots and heatmaps of the DEGs were also constructed in GraphPad Prism 9, and an enrichment Venn diagram was created using BioVenn (http://www.biovenn.nl/index.php). Microscopic images were processed on Fiji‐ImageJ. Sketching diagrams and multiple‐graph figures were created using BioRender (https://app.biorender.com/). Multiple graphs/images figure was created and exported from Adobe Illustration 2022.

## Author Contributions

Huu Thanh Le, Carola Venturini, Alicia Fajardo Lubian, Golo Ahlenstiel, and Scott A. Read contributed to the study concept and design. Carola Venturini, Alicia Fajardo Lubian, Bethany Bowring, and Jonathan Iredell provided essential bacteriophage isolates. Huu Thanh Le, Carola Venturini, Golo Ahlenstiel, and Scott A. Read participated in the acquisition, analysis, and interpretation of the data. Huu Thanh Le, Carola Venturini, and Scott A. Read drafted the manuscript. All authors critically revised the manuscript. Jonathan Iredell, Jacob George, and Golo Ahlenstiel provided funding and infrastructure support.

## Conflicts of Interest

The authors declare no conflicts of interest.

### Peer Review

The peer review history for this article is available at https://publons.com/publon/10.1002/eji.202451543

## Supporting information



Supporting Information

Supporting Information

Supporting Information

## Data Availability

The data that support the findings of this study are available from the corresponding author upon reasonable request.
