# Supplementary material for: Differences in Phage Recognition and Immunogenicity Contribute to Divergent Human Immune Responses to Escherichia coli and Klebsiella pneumoniae Phages
Source: Eur J Immunol. 2025 Mar 12;55(3):e202451543. doi: 10.1002/eji.202451543 (PMC11898580; doi:10.1002/eji.202451543)
Supplement: Supplementary file 1 — Supporting Information [file EJI-55-e202451543-s003.docx]

**Supporting Information**

**Supplementary Tables**

**Supplementary Table 1. Characteristics of phages used in this study.**

| **Phage** | **Morphology** | **Genome material** | **Phage size (width and length)** | **Bacterial host** | **Phage titre (pfu/ml)** | **Average endotoxin (Units/10^7^ phage pfu/ml)** | **SRA/GenBank accession no.** |
| --- | --- | --- | --- | --- | --- | --- | --- |
| JIPh_Ec70 | Myovirus | 163 Kbp DNA | 87.505 nm  239.654 nm | E*. coli* JIE3454 | 10^11^-10^14^ | 5.75x10^-5^ | SRA: PRJNA764821 |
| JIPh_Kp127 | Siphovirus | 113.7 Kbp DNA | 82.966 nm  374.799 nm | *K. pneumoniae*  ATCC 13883 | 10^10^-10^13^ | 7.75-10.9x10^-5^ | MN434096.1 |

**Supplementary Table 2: Pairwise comparison for DEGs between phages treatments, filtered phage controls and media control in PBMCs and monocytes**

| PBMCs DEGs | JIPh_  Ec70 | JIPh_  Kp127 | Media |  | Monocyte DEGs | JIPh_  Ec70 | JIPh_  Kp127 | Media |
| --- | --- | --- | --- | --- | --- | --- | --- | --- |
| filtered -JIPh_Ec70 | 923 |  | 0 |  | filtered -JIPh_Ec70 | 2778 |  | 0 |
| filtered -JIPh_Kp127 |  | 0 | 0 |  | filtered -JIPh_Kp127 |  | 0 | 0 |
| Media | 916 | 0 |  |  | Media | 2706 | 0 |  |

**Supplementary Table 3: Pattern recognition receptor inhibitors, agonists and targets**

| **PRR** | **TLR2** | **TLR4** | **TLR9** | **STING** |
| --- | --- | --- | --- | --- |
| **Inhibitor** | C29 (25 µM) | TAK-242 (1 µM) | ODN INH-18 (5 µM) | H-151(10 µM) |
| **Agonist** | Pam3CSK4 (1 ng/ml) | *E. Coli* LPS (1ng/ml) | ODN2006 (1 µM) | 2'3'-cGAMP (10 µg/ml) |

**Supplementary Table 4: Primers and probes for qPCR**

| Target genes | Sequences | Supplier |
| --- | --- | --- |
| Human 18S RNA probe | X03205.1 | Applied Biosystems (#43333760T) |
| Human *IL1B* | F: TCGCCAGTGAAATGATGGCT  R: GGTCGGAGATTCGTAGCTGG | Geneworks |
| Human *IL6* | F: GGTACATCCTCGACGGCATC  R: GCCTCTTTGCTGCTTTCACAC | Integrated DNA Technologies |
| Human *IL10* | F: 5’ GGCACCCAGTCTGAGAACAG  R: 5’ ACTCTGCTGAAGGCATCTCG | Integrated DNA Technologies |
| Human *IFNA2* | F: CTTGAAGGACAGACATGACTTTGGA  R: GGATGGTTTCAGCCTTTTGGA | Geneworks |
| Human *IFNG* | F: TGAATCTCCAACGCAAAGCA  R: CTGTTTTAGCTGGCGAC | Geneworks |
| Human *OAS3* | F: GTCAAACCCAAGCCACAAGT  R: GGGCGAATGTTCACAAAGTT | Geneworks |
| Human *CXCL10* | F: GTGGCATTCAAGGAGTACCTC  R: TGATGGCCTTCGATTCTGGATT | Integrated DNA Technologies |
| JIPh_Ec70-Major capsid protein | F: TGATTCAGGCGCATCTGACG  R: AGAAGTAGCCATACCTTCAGCGA | Integrated DNA Technologies |
| JIPh_Kp127 -Major head protein | F: GGCTAAAGCTCTGGAACTGA  R: CGTCTTCGAAATCTTCCTGC | Integrated DNA Technologies |

**Supplementary Table 5: Flow cytometry antibodies**

| Antibody | Supplier | Cat no. | Clone no. |
| --- | --- | --- | --- |
| Anti-human CD14-BV421 | Biolegend | 325628 | HCD14 |
| Anti-human CD14-BV711 | Biolegend | 367140 | 63D3 |
| Anti-human CD19-APC/Cy7 | Biolegend | 302218 | HIB19 |
| Anti-human CD3-BUV395 | BD Biosciences | 564001 | SK7(leu-4) |
| Anti-human CD56-BUV737 | BD Biosciences | 612766 | NCAM16.2 |
| Anti-human CD11c-PE/CF594 | BD Biosciences | 562393 | B-ly6 |
| Anti-human CD66b-AF700 | Biolegend | 305113 | G10F5 |

**Supplementary Figures**

**Supplementary Figure 1: Verification of PRR inhibitors.**

PBMCs were treated with PRR agonists in presence or absence the corresponding PRR inhibitors for 24 hours (see Supplementary Table 3). Expression of genes *IL1B*, *IL6* and *OAS3* were measured by qPCR. TLR, Toll-like receptors. STING, stimulator of IFN genes. Mean (red bar) and SEM (black bars) were presented. Inh, Inhibitor. Wilcoxon test, n=5, * p<0.05, ** p< 0.01.


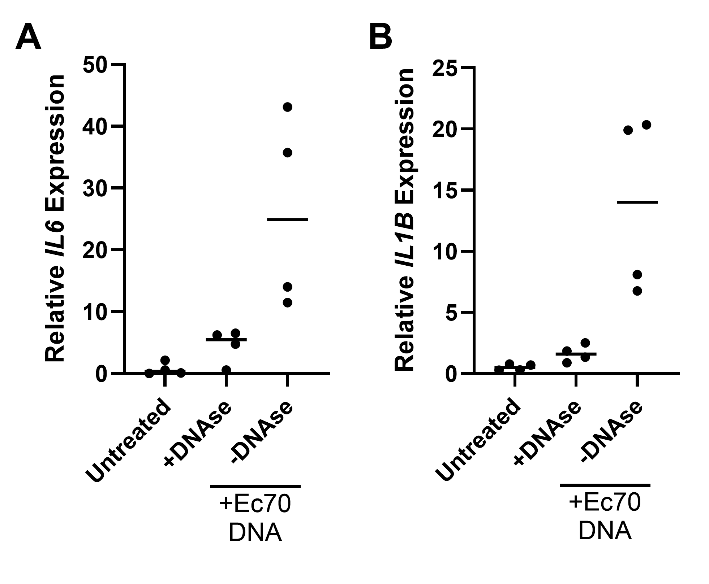


**Supplementary Figure 2: DNAse treatment of JiPh_Ec70 DNA inhibits inflammatory gene induction.** PBMCs were treated with 2 units of DNAse I for 10 minutes at 37 ^o^C followed by inactivation using 5 mM EDTA. Digested and undigested DNA was added to PBMCs for 24 h followed by quantification of A. *IL6* and B. *IL1B*.


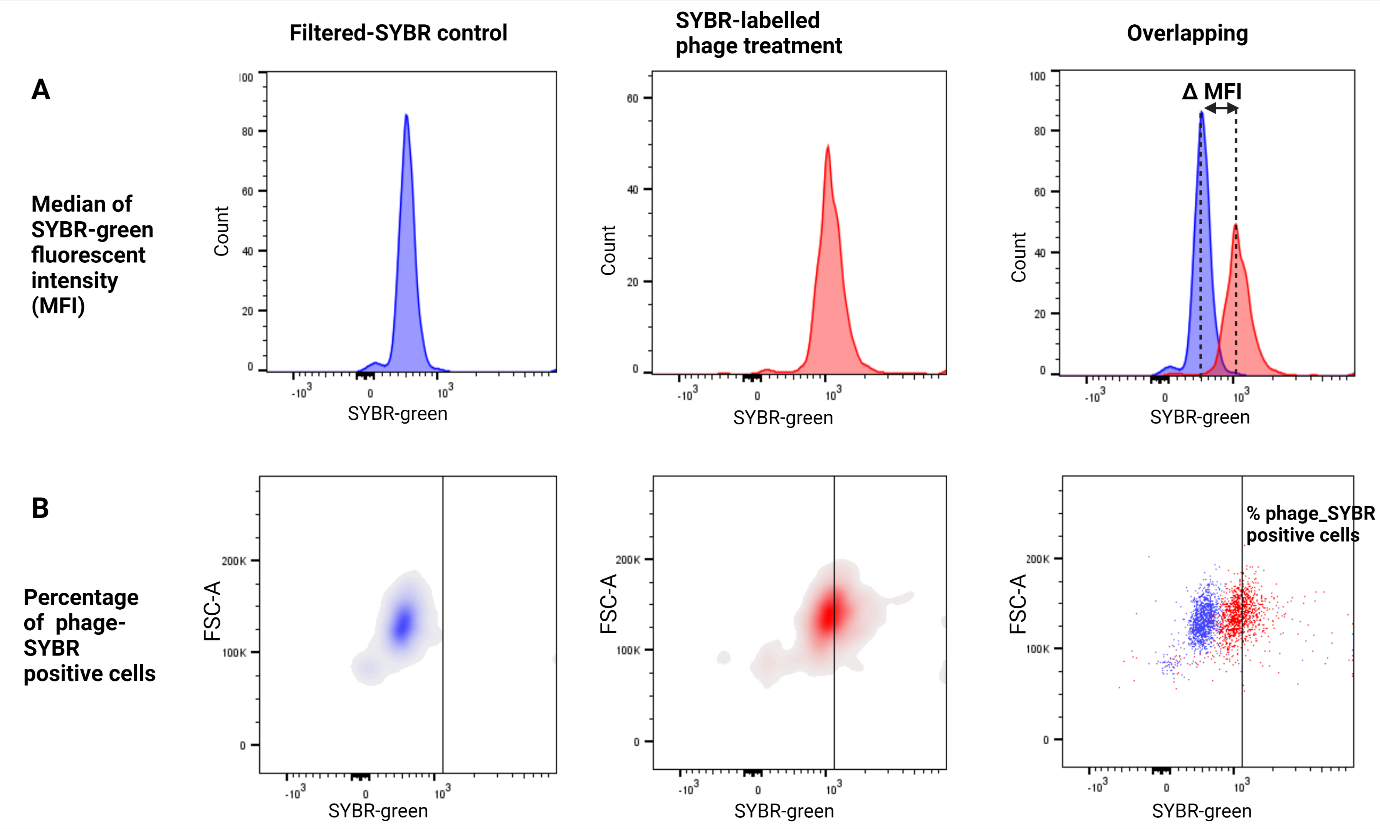


**Supplementary Figure 3: Calculation of cell population-specific ΔSYBR-MFI and phage positive cells.** A. Upon gating to identify PBMC populations, the difference in median SYBR green intensity (ΔSYBR-MFI) was calculated by subtracting the median SYBR intensity of cells treated with phage with cells treated with filtered-SYBR control. B. To determine the percentage of SYBR phage positive cells, filtered-SYBR controls were used to assign a cutoff threshold.

**Supplementary Figure 4: Verification of endocytosis inhibitor toxicity and efficacy.**

Monocytes (**a**) and neutrophils (**d**) were pre-treated with inhibitors targeting three endocytosis pathways (dynamin-dependent, macropinocytosis and phagocytosis) to assess inhibitor toxicity and efficacy. Neutrophils were retreated with macropinocytosis and phagocytosis inhibitors at 1:10 dilution due to the high toxicity for the cells at the original concentration. Monocytes (**b-c**) and neutrophils (**e-f**) pre-treated with endocytosis inhibitors were treated with pathway-specific fluorescently tagged cargoes (FITC-transferrin for dynamin-dependent endocytosis, FITC-dextran 70kDa for macropinocytosis and SYBR-stained *E. coli* JIE3454 for phagocytosis). Monocytes were labelled anti-human CD14-BV421 and neutrophils with anti-human CD66b-AF700 and viability dye (FVS700 or Aqua Zombie) before proceeding to flow cytometry. Comparative of Δ SYBR-MFI and percentage of SYBR-phage positive cells were calculated. Mean (black bar) and SEM were presented. Paired t-test, n =4, * p ≤0.05, ** p ≤0.01.


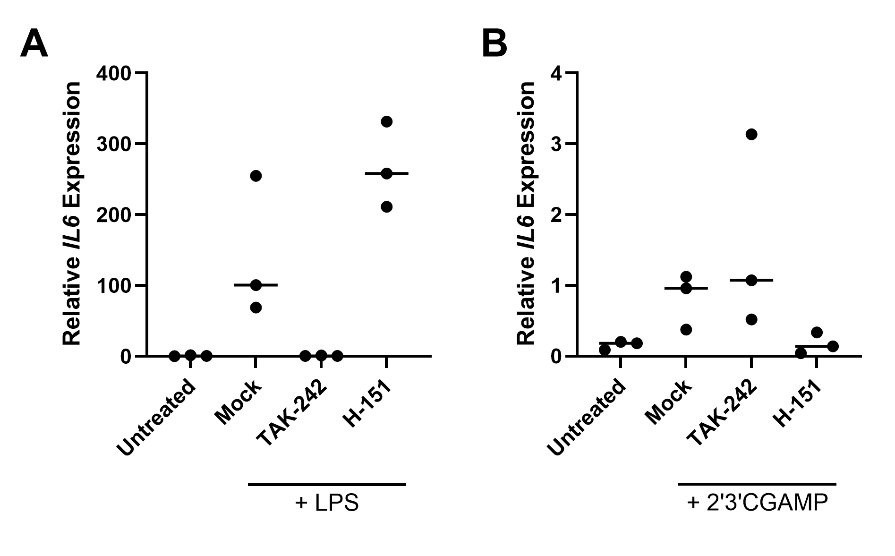


**Supplementary Figure 5: Confirmation of TLR4 and STING inhibitor specificity.**

PBMCs were treated with TLR4 and STING inhibitors TAK-242 and H-151, respectively for 30 minutes followed by the addition of **A.** TLR4 agonist LPS or **B** STING agonist 2’3’ CGAMP. Following 24 h incubation, *IL6* gene expression was measured by qPCR demonstrating inhibitor specificity.

**Supplementary Figure 6: Optimisation of DNA ejection temperatures.**

JIPh_Ec70 and JIPh_Kp127 phages were incubated at either 60, 70 or 80^o^C in the presence of 16 mM EDTA for 90 minutes to induce DNA ejection. Phage genomes remaining inside phage capsid were quantified with qPCR. JIPh_Ec70 genomic DNA after treatment at 80^o^C was not detected.


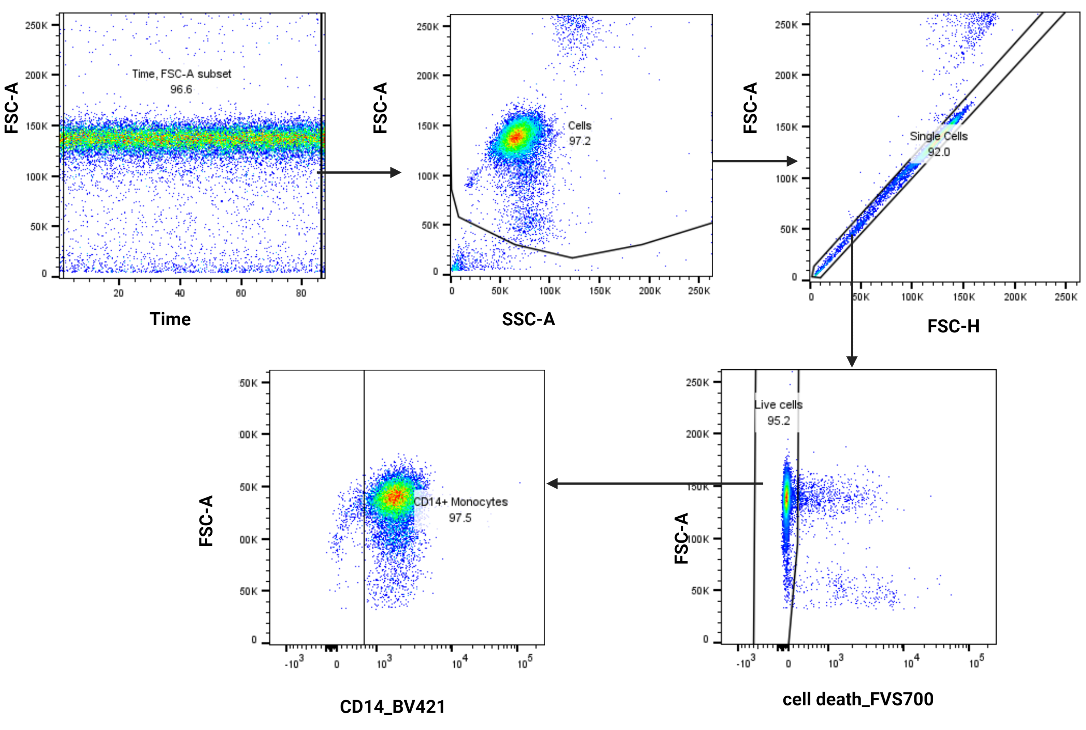


**Supplementary Figure 7: CD14+ monocyte gating strategy for purity assessment.** All events were plotted with Forward Scatter Area (FSC-A) over running time to ensure a stable cell flow. Cells were next gated using FSC and SSC to remove dead cells and debris, followed by single cells using the diagonal of FSC area and height. Live FVS700 unstained cells were then gated to select CD14+ monocytes.


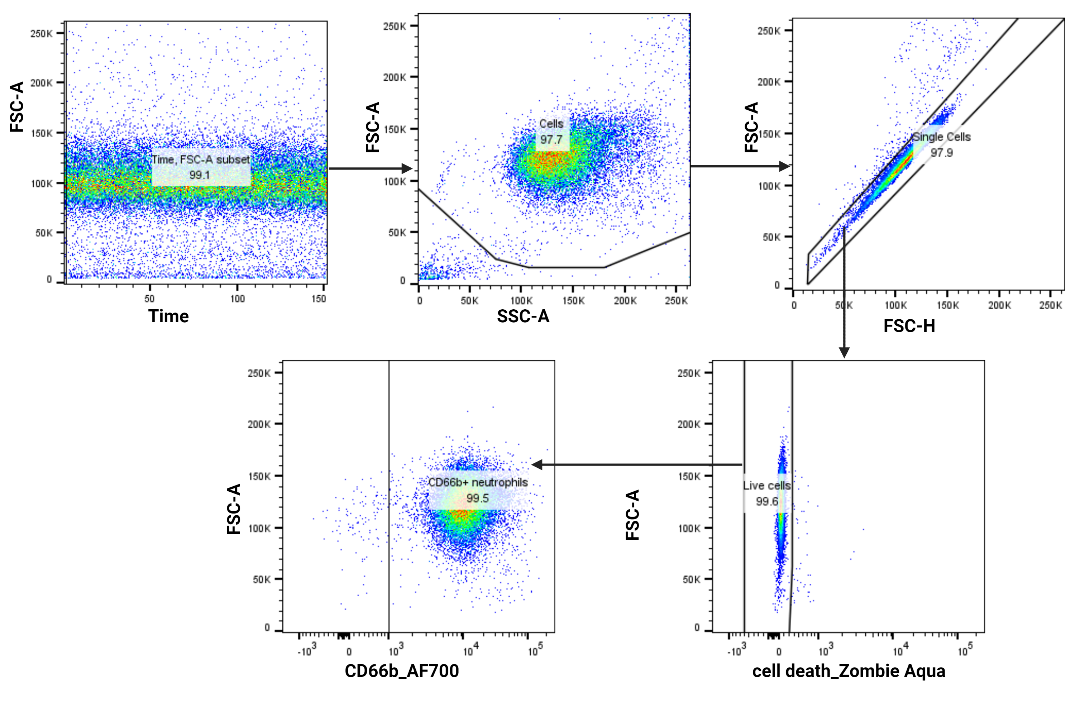


**Supplementary Figure 8: CD66b+ neutrophil gating strategy for purity assessment.** All events were plotted with Forward Scatter Area (FSC-A) over running time to ensure a stable cell flow. Cells were next gated using FSC and SSC to remove dead cells and debris, followed by single cells using the diagonal of FSC area and height. Live zombie Aqua unstained cells were then gated to select CD66b+ neutrophils.

B-Actin


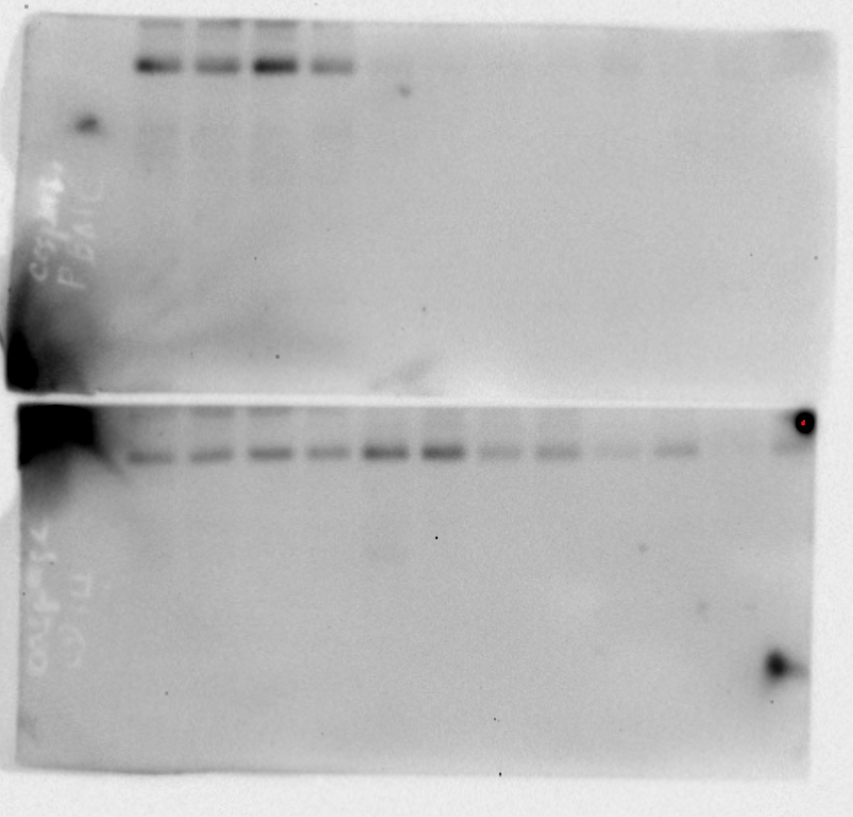


IRF3


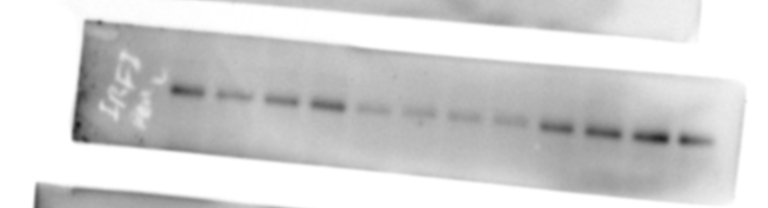


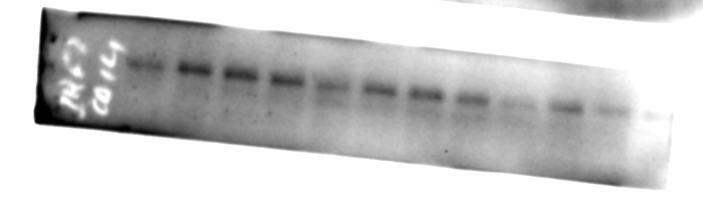


pIRF3


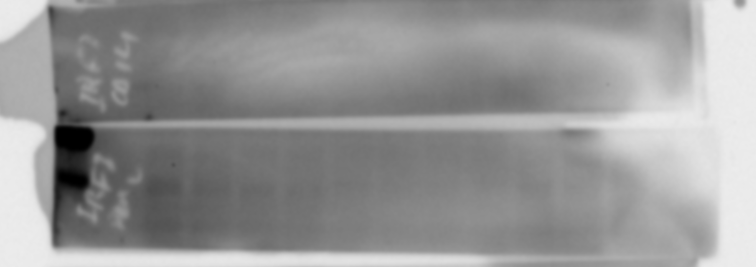


P65


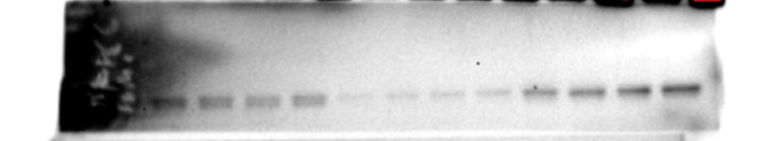


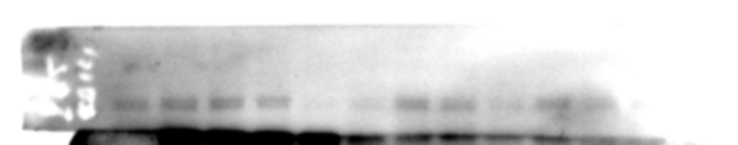


p-P65


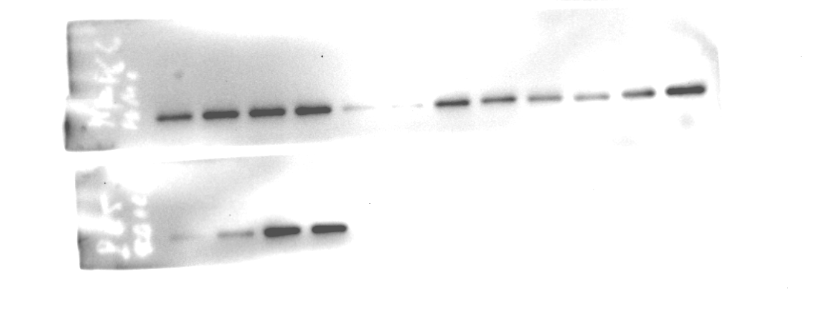


**Supplementary Figure 9. Full Western blots displayed in Figure 2c.** Protein indicated on blot does not necessarily match the protein listed, as most blots were stripped and re-treated with antibodies to make best use of limited protein.
