# Supplementary material for: Differences in Phage Recognition and Immunogenicity Contribute to Divergent Human Immune Responses to Escherichia coli and Klebsiella pneumoniae Phages
Source: Eur J Immunol. 2025 Mar 12;55(3):e202451543. doi: 10.1002/eji.202451543 (PMC11898580; doi:10.1002/eji.202451543)
Supplement: Supplementary file 2 — Supporting Information [file EJI-55-e202451543-s002.docx]

**Ec70-T4 Hoc gene (NC_000866.4) alignment**

T4Hoc --------------------------------------ATGACTTTTACAGTTGATATAA

Ec70 CAGAAATTAGTACAACTATCACAGTTCAAAATAAGACACAGACAACTACTTTAGCTGTAA

*** *** * * * ***

T4Hoc CTCCTAAAACACCTA---CAGGGGTTATTGATGAAACTAAGCAGTTTACTGCTACACCCA

Ec70 CTCCTGGTAGCCCTGATGCTGGAGTGATTGGAACTCCAATTGAATTTACCGCTGCCTTAG

***** * *** * ** ** **** * * * ***** *** *

T4Hoc GTGGTCAAACTGGAGGCGGAACTATTACATATGCTTGGAGCGTAGATAATGTTCCACAAG

Ec70 CTTCACAGCCATCAGGTGCAAACGTTACGTATCAATGGCACGTTGACGGTTCTCCTGTAG

* ** * *** * ** **** *** *** *** ** * *** **

T4Hoc ATGGAGC---TGAAGCAACTTTTAGTTATGTACTAAAAGGACCTGCCGGTCAAAAGACTA

Ec70 ACGAAGCAACTGACGCTACATTCAATTACACTCCTGACACAAGCGGAGTTAAAAAAATCA

* * *** *** ** ** ** * *** * * * * * * **** * *

T4Hoc TTAAAGTAGTTGCAACAAATACACTTTCTGAAGGAGGCCCGGAAACGGCTGAAGCGACAA

Ec70 AGTGTGTAGCTCAAGTAACTGCGGCAGATTATGATGCACTGAGCGTTACTTCTAATGAAG

**** * * ** * * * * * * * * ** *

T4Hoc CAACTATCACAGTTAAAAATAAGACACAGACGACTACCTTAGCCGTAACTCCTGCTAGTC

Ec70 TGTCACTGACTGTTAATAAAAAGACGCAGACAACTACCTTGGCAGTAACTCCTGATAGTC

* * ** ***** ** ***** ***** ******** ** ********** *****

T4Hoc CTGCGGCTGGAGTGATTGGAACCCCAGTTCAATTTACTGCTGCCTTAGCTTCTCAACCTG

Ec70 CTCCAGCAGGAGTTATTGGAACCCCAGTTCAATTTACTGCTGCCTTAGTTTCTCAACCCG

** * ** ***** ********************************** ********* *

T4Hoc ATGGAGCATCTGCTACGTATCAGTGGTATGTAGATGATTCACAAGTTGGTGGAGAAACTA

Ec70 TTGGAGCGTCTGCTACATATCAATGGTATGTAGATGATTCTCAAATTGGTGAAGAAACTA

****** ******** ***** ***************** *** ****** ********

T4Hoc ACTCTACATTTAGCTATACTCCAACTACAAGTGGAGTAAAAAGAATTAAATGCGTAGCCC

Ec70 ACTCTACATTTAACTATACTCCAACTACAAGTGGAGTAAAAAGAATTAAATGCGTAGCCC

************ ***********************************************

T4Hoc AAGTAACCGCGACAGATTATGATGCACTAAGCGTTACTTCTAATGAAGTATCATTAACGG

Ec70 AAGTAACAGCAACAGATTATGACGCAAAAACAGTTACTTCTAATGAAGTATCATTAACAG

******* ** *********** *** ** ************************** *

T4Hoc TTAATAAGAAGACAATGAATCCACAGGTTACATTGACTCCTCCTTCTATTAATGTTCAGC

Ec70 TTAATAAGAAGACAATGAATCCACAGGTTACATTGACTCCTCCTTCTATTAACGTTCAGC

**************************************************** *******

T4Hoc AAGATGCTTCGGCTACATTTACGGCTAATGTTACGGGTGCTCCAGAAGAAGCACAAATTA

Ec70 AAGATGCTTCGGCTACATTTACTGCTAATGTCACTGATGCTCCAGAAGAAGCACAAATCG

********************** ******** ** * *********************

T4Hoc CTTACTCATGGAAGAAAGATTCTTCTCCTGTAGAAGGGTCAACTAACGTATATACTGTCG

Ec70 AATACTCATGGAAGAAAGATTCTTCTCCTGTAGAAGGGTCAACTAATGTATATACCGTTG

******************************************** ******** ** *

T4Hoc ATACCTCATCTGTTGGAAGTCAAACTATTGAAGTTACTGCAACTGTTACTGCTGCAGATT

Ec70 ATACCTCATCTATTGGAAGTCAAACTATTGAAGTTACTGCAATTGTTACTGCTACTGATT

*********** ****************************** ********** * ****

T4Hoc ATAACCCTGTAACCGTTACCAAAACTGGTAATGTAACAGTCACGGCTAAAGTTGCTCCAG

Ec70 ATGATAGCAAAACTATTACAGCAGAAGGTCAAGTTCAGGTAACTGATAAAGTTGCTCCAG

** * *** **** * *** * ** ** ** * **************

T4Hoc AACCAGAAGGTGAATTACCTTATGTTCATCCTCTTCCACACCGTAGCTCAGCTTACATCT

Ec70 AACCAGAAGGTGAACTACCTTATGTTCATCCTCTTCCACATCGTACTTCAGCTTATATCT

************** ************************* **** ******** ****

T4Hoc GGTGCGGTTGGTGGGTTATGGATGAAATCCAAAAAATGACCGAAGAAGGTAAAGATTGGA

Ec70 GGTGCGGTTGGTGGGTTATGGATGAAATCCAAAAAATGACCGAAGAAGGTAAAGATTGGA

************************************************************

T4Hoc AAACTGACGACCCAGATAGTAAATATTACCTGCATCGTTACACTCTCCAGAAGATGATGA

Ec70 AAACTGACGACCCAGATAGTAAATATTACCTACATCGTTACACTCTTCAGAAGATGATGA

******************************* ************** *************

T4Hoc AAGACTATCCAGAAGTTGATGTCCAAGAATCGCGTAATGGATACATCATTCATAAAACTG

Ec70 AAGACTATCCAGAAGTTGATGTTCAAGAATCGCGTAATGGATACATCATTCATAAAACTG

********************** *************************************

T4Hoc CTTTAGAAACTGGTATCATCTATACCTATCCATAA-------------------------

Ec70 CTTTAGAAACTGGTATCATCTATACCTATCCATAATCATAAGGGGCTTCGGCCCCTTTCT

***********************************

**Ec70 Genome**

1GGCCGCAAGGGCCTTCATAGTTTTAGCGATTTGAGAAACTTCATCATCACTTAAAGAGTT

61GCGATAACCGATGAAGTCGGAAACAATTCGGAATTTCTTGGTAAACTCAGCAACCATTTT

121ATCACTGTTTTTTGAAGCATTATTTGATAATACATCAAAAAGATTAGTTACTGTCCAGAT

181GTCATGACCAATGGTATCTTTTCCACCATTAAAATATACACCGCGCAATGAACTAACTAT

241ATTAGCGAGTCGTGTATATTCTTCAGAAACTTCATCTGTACTGAAGTACTTCATCATAAA

301ATCTAACTCAGGATACTTGATAATTTTATCAATATATCGTTGAGCTGAACTTGAATAACC

361TACGTACTTATCATAATCTACATCATCAAAAGCATCTACATATAAATCACGCAAAGTTTC

421AAAAATACATTGGCACTGACCGAGTTCTTTTACCTTTTTCTGTAAAAGCGGACGAATAAC

481ATAAAATTCATTAATGCCAATAAGATTAGCCATACGAATCAAAATATTCATAGATGGATG

541ACAAAGAGATGTAGTACCATCCATAGAGAAAATATCAGAACGATGCATATACGCTACATA

601ACCAGTAATTTCATCTGCTTCTGATGTGAGCGTAAATAATTCCTCTTTTTCCCAGCGCCC

661GTCTTTAATTTCAAACTTAAATGCTGTAGCAGCTTTAGGACGAGGAGCTTTACTTTTAAC

721TACCTTTGGAATATAGCTTTTAACTAAAGCTTCGATTTCTGACAAATAATGAATGTTAAC

781TTCATCATTTTCAAACATTGCCATAATATCAGGGAGTAAATCAATCTGTGATTCTACTTC

841CGGATTAATAAACAGAAGACGCTCATTGTGATGAATATTTAAAGTGTTATTAAATTCACT

901ATCATCCAATGCACGTGCTAATCCACGGACAATATTAACACGATTTTTAATATTATCAAT

961AACGATATTAATTTTTGTTGTATTAATACCAAACAGACGATAACTTGATGCAACGGCTGA

1021AGTTTCATGACTTTGCTTAATGCGTTTCAGTCGAGGGTCAAGATTTACTTCATACACAAC

1081TCCCGCGTTGCATAACTTACTATCAGGTTCAAACATACTCTGCATCTTCTTATATGACAG

1141ATTTTTAGTCGTGAATTTGACTGAATTACTAATCATATAATCTCGAGCAGAATACCCCAT

1201CTTCATCAATTCACGATATGTGTGACGAGGAGATGTAGATTCTTTAAATCGTTTTACATC

1261TTCATTAAATGCTTTCTCACTGAGTTCTTTAACTCGTTCAATAATATTTTTACGAGTGCG

1321ATCATCCAGTGAAAGAGCCTCGCGAGATGGAGCAATATCAAGTGAACCCATTGGAAACTT

1381AATGTAATTCACTTCATTGCGAATGCTTAGCCAGTTACGGTCTCTAATAACACCATCGAT

1441AGGATAAACAATACCGCCATAGATAGCATATAATCCACCACGATCTGGCCAGTATCTTTC

1501TGGATTTACGCCATAATAATCATCAAAATCCGGAAAATAATCAATTTCGCGGTCAAGACC

1561ATTAATGATAGCCAAATCTTTGAACGGTCGCATGATATAAGAAACTTCATAAGCAAAGTT

1621TCTAAAGTCTTTTTCTTCAACTGGAACTACAATTTCAATACCAGTTTTATCGTCTGGACC

1681CATTTCCTTTACGAATGTAGGTTTAATCTGCGGACCATCACCATCCATGTAAGCTACATA

1741ACCACGAATTTCACCTTTATGATACGAAGTAATACTAAACGTATCAGTATAACTAAACGG

1801AGATTTAGAACCTAAACCAAATCCGCCAATAAAGTCATTAGATTCAGCTTTAGATGAACT

1861GAAGTATGAATTATACAACCCAGGAGAATTATCATCACCCTGAATATCAAAATCACTCAT

1921ACCCGGACCAAAATCTCGACAAACAAATCGCGGGTCTAATCGTCCTGGAACTTGAATGAT

1981AAATTTTTCAGGATTTCCATTGAGAGCATGGGCATCAATCATGTTAGTAATTAATTCACG

2041GACCACTGCGCGAATCTTGTTTGTATACAAATCAGATGATAGAATTTTAAATACTTTAGG

2101ATTTGCTGCGATGCTAAATGCTTTTGATTTAGAACCATTGCCAAGAATTGTTTCTTTTTC

2161AGTGGTGATAATCATAATTTCCTCATTAATTCATATTACGCTTAATAACTTCAGCAACTT

2221CTAGTAGTTCATCTTTAGTTGCGGTGTCGGATTGAATTTTATCTCTAATATCCTTAAAGC

2281GGTTTTTAAATTCTTCGGCTTCTCCCATATCGAAAAAGCGTTGAATGATTCTATATTCTC

2341GATGAACTGCTTTATCAAAAAGTTCTAAATTTACTTTATATGATTTCATTTCAATATCCT

2401CATTTGCCCAATTAATTATACCACATCCTTGTGGTAAAGTAAACTATTGGCTCATCCATT

2461CTTTACGAAGGTCAGCATTATCTCCCATGAGCATTTCAAAAAGCTCTTTCCAGTTCTCAG

2521GAAGTTTAACAACATCATATACTGGGTTTTGAATCATCTCACGATATTCAGATTTTTCCA

2581AAGAGCCAAGTCCTTTAATATAACGGATGCTATGTTTAGGTAGAGCATCTTTAGCACTCT

2641CATATTCAGCGACTGTATAAAACCATTCTTGTTTTTTACCGACCTGAGCGATGATTACAG

2701GAGTTTTGACAAAACGAATTCGTCCTTGCTCAAACAATTCTGGCCAATTACTAAAAAATC

2761CGAGCAGAGAAGGATAAATGCTTCCTAGACCATCATGGTCAGCATCAGTCATAATAGCGA

2821TATTATGATAATTCAAGTTTTCAGCTTTTTCACCGAGAACTAATCCAGTGATTGCGCAAA

2881TATCAAATAGTTCTTTGTTTTTAAGCATGTCAGCATATGACATACCCCAACTGTTGAGAA

2941CTTTACCACGTAATGGATAACCACCGTGAAGTTCTTTATCACGAACATCAATAAGATATC

3001CGATAGCCGAATCACCCTCAGTCAAGAAAAGAGTAGTATCAGCATCTTTACCACAAAGAT

3061TCGCTTTAATATGTTTATGAACCTTAGCTTTAGAAGCCTTTTTAGCTGCTTTAGTTTCTG

3121CTGCTTTTTCTGCCGCTAATTTACGAGCCAAAGCAGCTTCAATAATCGGCATTAGAATTG

3181CTTCATTATTTAGAATAGCACGTGAAATCTTTTTAGCATCAAGTTGAATATGACTACGGA

3241TTTCACCAAATGGAGAAGTCAAACGCTCTTTAGTTTGAGAATCAAATCGCATGTTTTTCA

3301TATCACGGACAAACATAACGATAGTCAAACATTCTTTAACGCGTGCTTTAGTCACATCAA

3361TTTTGAACTTACGTTTGATTTGTGGAATAAGGTCTTCACAAATATCATCCATAACACAGT

3421CAATGTGATGGCCACCATTCTTAGTGTGAATGTTATTCACATAAGTTAATTGACGAAAAC

3481CATCCGGTGAACGACCAACCGCAATAGAGCAATTTTCTTGTTCTTGAACGATAGCATGCT

3541CATCATATTGGCGTGCATATTTCTTAAAATTGCCCTGAACCTTTTTACCATTAAAGGTAA

3601ATTGAATATCAGGATAAACTACTGCAAGTGTCTGGAGACGATCTAGTGTAATGTCAAGAT

3661AAACTTGGGACAGCTCATTAGTTTCAAATGACATAAAATCAGGAATGAAAGTAACGCGAG

3721TTCCTTTCCATTTTCCTGGAATAGTTTCCCATGATTTATTTTCCATGCCATTTGAACAAC

3781GAACTACAATATTATTTTGACCATCACCAGTTTCACCGACAAACATCACAGAAAAAATGT

3841TTGTCAAACTAGAACCAACACCATTCATACCACCGGTGACGCGTTCTTTATCATCACCAA

3901AGTTACCACCTGCTTTTGGAATAGTCCACGCAGCAACTGGACCAGGAATTTCTTCACCGG

3961TAGGTGTTTTGACCATCGCTTGTGGAATACCACGACCGTTATCTTCAACTGTTACTTGAT

4021TGTTTTTAATAGTAACATTAATTTTATTTGCGAATTTAAACTTAGTACGAATACCTTCAT

4081CTACTGAGTTATCGATAATTTCATCAATAAGCTTAACAAGACCAGGTACATACTGAACAC

4141TTTCCCATTTACCAAACAGAAAGCGCTCATGCATTTCATTAGCGGAAGAGCCAATGTACA

4201TGCCGCTACGCTTTTTGATATGTTCAATATCGCTCAGAATTTTAATTTCATTCTTAATCA

4261TCACTTATCCTCGTTTGGTTTCGGGAATATTATACTCCGGTAATCATAAAGCTAAAGGCC

4321CGAAGGCCTTTTATTTAAAACGAATAGTTGAATCCTTAAAGAACAGCCCAGAACATACTG

4381TTCCTTCTACTTTCTGCCCAGTAGGTCCAATAGCACGAAATCCAGTATGTTGGAAATCAT

4441TTTCAGAACAACCGAACCAGTTATATCCAGTGATTTCAATATTAGTAAAACCGCTTGAAG

4501ACAAAACTTTGGTTGCATTATCAGCATCAGTACATCCTACTAAAGACACTGCTAATACTA

4561ATGCCGCGATAGAACGATTAATATATTTCATAATTTTCACTTAAATTTAATGGCTTGAAG

4621AAGACTAATAATTCTCAAGCGACTTCCTTCATCTTTAACTGTAAAAGAAAGAGGGTCACC

4681AGATTTCATAGTGATAGTGCATTCAAAAGCAAAACCTTCGGGAACTTCTTCGAAGAAGTC

4741AAATTCTTCATCATAAATTAGAACATTACTCTGAAAACTGTGAAGAATTTTTCCGTCATT

4801TCCAGATGCTGTACTAATCATTGTAACATTATTACCTTTCATATCTTCAACGATAAATTC

4861GCTTGTAGATATTACAGCATTAATAGAACCATTCCTATAAATAGCAGAAAATAGATATTT

4921CTTTTCTTCACCTTCGCGAATGCGATATTTCTTACCGATTTTAAACATAATTACCCTTTA

4981AGTAAGTCGTAAAATCCGCCATTCACATGCTTAGGGGCAGAGACTAACCGAATAGCAATC

5041CGATGACAATCAGGACATACATCAGTATCTCTTTCAGAAATTTTCTTGATTTTTTCGTAT

5101TCTTTTGCGCAGTCTTTTGATTGACATTTATAATCATAAAGCGGCATAATTATTCCTTAA

5161AGTGAGCTTTCAACATCTGATACAAGGACCATGCCTGTTCATCATTTTCAATAGTAACTT

5221TCATTACAGGGAATTTAGATTGATCAGTTATCGGTGCAGATTCTTCCTGCTCTTCAGCTG

5281CCTGGTATGGATTTTCAACTTCATTAAAGAATTCAGCTTCATTCGATGAAAGCCAAATAA

5341AATCTTCATCCAAAATATCTTTACCAGAAGATAGTACTAAATTTCCAGCAAATGACATAA

5401TTTTAATAGGACGCCCAAGAGCATCCACATCTAAAATTTTAAAAGGATGCATACCTAAAC

5461GGCGTGCATAGATTCCGTTATCAGTATGGTCTTTAATAAAATTTTCTTGAGCTTGTTTAT

5521TTTTAAATTGATACCATTTATTAACTTCAAATTTAATAGCCATTAATAAATTTCCTTCCA

5581GTAAGTTGTACCATCTTCAGTGATTTCACGAAATACACCGTAAATTGGTTGTTTATCCCC

5641AACCTTTTCATACACATAAACCGAAGTCAAGTGAGTAAACTTAGCAGTGTGTTCCTTTTG

5701AACTACTACCAAATCTGGATCGAATAATACATCTTCAAATTCATCATTAGTGCAATTCTG

5761AACAATTTTACGTTTCATCATAATTTCCTCATTAATTGAACATTGGAGCGATGCGTTTCA

5821GAAGAGTAGCAGCACCTTTGGCGAATTATCCATTTTATTCTCCAAGTTGTTTTCTGTATC

5881AGTAGTTGATATTGATATAGTACCATAATCAACTACTGATGTATATAGTTTTATGAAAAA

5941ATTTTAAACTTTATGCATAGCGAGCTTTGCTGTAGTGTTTAATCCAACTTTCAGGAATGA

6001CTTTGTATGTTCCTAAAAATACCGCGTTGTATAACTTAACGCCATCTTCTACCCATTGAT

6061CAGTAATGTATGCACACATAGCGCGAGTACGCCGAGGAAGTGGTTGTCCACCTTCGATAA

6121ATTCAAACTCATAAGGAGCAATGAACTTAATAGCTTGACCGAGTTTCCACTTAAAGTCTA

6181CACCTACATGCGAAGTATCAATCGTTTCAATTCCTTTAGCGGGAACAGCTTTCAAAAACG

6241CAGATTCAAGAAATTTCGCGCGAACATAACCAAACTGAGGTTTAGACTTTCCATCTTTAG

6301GAATGATACGCACTTTTACTTCAGAATCTTCATCTTTAACGCCGTGTTTAAGCTGAATGC

6361TTACAATTTCGACCAATTTTCCTGCTGCTTTAGAACGGGATTTATCAGATACACGAGCAA

6421TTTCACCAATATTAATAATCATAGTTATCTCTCACTTGTTAAAAAGATTTTATACTCCAC

6481AGGACCATTATACTCTGGTCCCAAGAGTTTGTAAACTATTAATTCAAAATAGCTACCACC

6541GCACTACGAGGTACTACACTAAAATCTCCTGCATGGACAACGTTCAGAAGCTCAACACCA

6601TCTTCAATCCACTGGTCTGTTACCCAACCACAGATAGGATTATCAAAAGGACGACGGATA

6661GACACAGCAGCACACAATAGGTCTGTAGGGTCTTGTTCTTCAACTTCTTGGAACAGGATG

6721AACTCATCTTCATAAACCAGGTTTTTCTTTATTTTGTTATTATCTAACCGGCGACCGTGG

6781ATGACGTAAGATTTGTCAAACCACTCACTTTTGGCAAATTCTTCAACAATAAATTCGCCT

6841TCGCCAAAAACATCAGTCAGAGTTTTATGACCAGAAATCAAGGCATTAGTTTTAATTTTA

6901GGTTCAACCAGTTTGTAGGTTTTGCCGATTTCAATAGCGGTAGTCATAGTAGGTTCCTTA

6961ATTTCCAGTGGTTTAACAGGGCATACATAAGTGCTTACAACATCAAAATCAATCAGTTTA

7021GCTACCGGATTTGGTATGTATTCAGGATTATAATTAAATTTCATAATTATCTCATTTCAA

7081TAAAATCTACGAGTTCAGCATGCGATTTGCGGAACATTACTTGGTGCCCACCAATGATAA

7141CTTCATCTTCAGGAACTTCGTATACAGCGAGATAAAATCCTTTAGAAGATAATTCTTCCC

7201GCTCTTCTCGTGTGAACCATCTCATCATATCATACTCACTAGCGAAAGCAAAATGATAAA

7261GAGCTATAAACCATCCTGGAATATGATATTCTACTCCAACATAATCTTTCTTGAACTTAG

7321TATTAATTACTATATTAGCGTTTTTAACTAATAGTTTATCTTCGTGTGGTAAAGGAATTC

7381TTTTATTATTATCGCTATGATGCATAAAATTAGGTCTGTCATAACCTACGTGTAATAGCC

7441ACTCTTCGCTCCATGGATCTATTATACTCCTGTACGGCGTTATTTGAACACAAAGATTTC

7501GACGTATTGTTATAGCATCTTCATAATCAAGAATACTAAACGATGATTCAACACGATAAA

7561TTTTCATTTTATTATCCTCAGTAGCTATGGTGTTATAATACCACAACTAACCGAGGAAGT

7621AAACAACTTTTTATCGTTTTGTTGGAAGAGATAGAGGATCGCAATCTTCCTCTGATGGAG

7681CATCTTCAAGACCCATAGCATATCGCAAAGCGTACTTCATCATCAGGATGTCTTTCGCAC

7741AGTCATGAATAGAATCATGCGCAACGAATCCATCTAAAGTTCCTTTTGGAAGAGGACACG

7801TGGTCATATCACGAACAAGCAGAAGTGCTTCAATTCTGGTACGAATATCACGCTGATTCC

7861AGAATTTACATGGTTCTAACTTAAATGTGTCAAGTTCATTCTCAGAAACGCCGTTAAGAC

7921GTTGAATATCGCGAATGAGATCGACTAAAATTGGAAAATCAAACGACATTCCACGGCACC

7981AGCCTTGAGATTTCCAAGGATCGATATTATGTGCATTGATGTAATCATTGAATTTTGCAA

8041TACCATCAATGGTGCTTACATCTTCATCTGATGGTGCAATATTTTTTCTAGCTTCAGGCG

8101ATTGATTTTTCCACCATTCGATAGTGCTTTTAGTAAAAAGACGATGTCCTTTTTGGCTTT

8161TTAAATCAAATTTGATTTTAATGCCACGTGAAACTAATTCATCAAATGTTTCAACTACTT

8221CTGGGTTAGGGTCAAAAGCAATTACAGCTAAATCAATAACAGCTGCTTTTTCACCACTTC

8281CCATTGTTTCAAAATCTATAATAAAATCAAACATTAAATTTTCCTCGCTAAATCACGAAT

8341TTGACCTACAGTATAGTCTTGAATATAAACTTTATTGATAGGCTCATCAATAAATTTTGC

8401CATAGATTCAATATCTTTTTGTATTTCTTCAAGACTGTATACTATCTTTAAAGCTTTTTC

8461GCGAATAGTAATATTTTCAGGACCAGAATTTTTCTCAATGACAGCTTTAACATTTGTCAT

8521AAGAGATTTAAACTGACACCAACTTAATTCAATCATTAATAATCGCCTTATAAAGATAGC

8581TAATTTCACCTAAAACATAATCACTGATTGTGACAGTTTTCACTTCACCACAAAAGAATT

8641CTAACGCAATTAAATCTCGTTCAATTTCTTCTAATTGAAGCATCAACTTACTAGATTCAA

8701TTTTTACAGTTTCACGATTTTTACTATAAGCTATTTCATAAATTTCGCTCACTTTATCTT

8761GAAGCAGATAAAACTGGTCTTTAGTTATTTCCACGAATAGCTTCCTCAAATTTAATTAGT

8821AATTTACAGACACTTTCATCTAGTGTAATTACCGTATCTGTATTACTGTGATATGATTGT

8881TTTTCAATTCGTTTAATTGCTAATATAGTATCACTTATAAGATAAAGCTTATGAAGGAGC

8941TCAGCTCCTGTTACCATAACATCGAGATTATCTTGCTTATTAGTATGCTTCCTTATACCA

9001TCTATTAGACGATTTAAATATTTTGAATTGATGTTAATCATATATATCCTCACACATAAA

9061ATACGTCATAACGACCACGGGTAACACCAACATAAAGAAGTTGTTGAGCCAATTCAGCAT

9121CTGCATAATGAATACAAGGTGTATAGATGAAAGCACGGTCTACAGACATACCCTGCGCTT

9181TATGGAATGTTGATGCAGGAAGTGCTTTCACTTTACTAAACTGTGATTTAGCATCCCAAA

9241AATCACTCCATGGAGCTTTTCCACCCTTATTCCAGTTTTTATAAGTTTCTGCTGTTTTAC

9301CTAAAAATAGGTTAAACTTATATAGCTCTTCATCAGATGAAATTATTTTAATCTTTTCAC

9361GATAATATTCATCATCGCCGTAAGTTTCTACTGTTAAATCCCAATGACGAATTAAGTATT

9421CTCCAGAAACACCACGAGCTTTAACAAATGTTGATGTATACTCTGCTTCTATAATACGAA

9481CTAATTGTCCGTTATTAAAAATAATTTCTGACACAGGCTTTCCATCAATTTTATATGTTT

9541TAATTAATGGTTCCTGCATTACAATAATTTCACCGACAATAAAATCTTTATCAGTTTCAA

9601AAATCTTTTTACGAATAATGCTATTTAACTTGTCAACAGATTTATTCGTAAATGCCATTA

9661CGCGATTTTCAAACAAATCATCTAGAGATTTGACGATTGAAAAATAATTTACCATAAAAT

9721CGCGTAAAGCGGTATCACCAGTAAATCCACGTACTCCATGCCCGTCAACAACTTTATCAT

9781AAATCCACTTACCGTTACGAACGTCAGTAGCTACATCAATAATAGGAGCATTACTGCGTT

9841TAACTTCAGTGAGTTCACACTGATAAAAATCTTTATGTGTAAAGAATGGACTGATATAAG

9901CAGTATTTTCTCCTGGATCAACAGGTCTAATTTGCTTATTATCACCTATTCCAATTATAG

9961TACACCAAGGTGGAATAGTTGAAAGCAGAATTTTAAATAGCTTTCTATCATACATTGACA

10021CTTCGTCGCAGATTAATACCCTGCATTTAGCTAAATCTGGTACTTCCTTCTGTTCAAAAA

10081GAACGTTTTCTTCATACGTAACTGGGTTAATTTTAAGAATACTGTGAATAGTACTCGCTT

10141CTTTCCCCGACAGTTTTGAAAGAATCTTTTTAGCTGCATGTGTAGGAGCTGCTAAAATAA

10201TACCAGTTTCACCCGTAGATATTAAAGCTTCAATGATGAACTTAGTAAGAGTAGTCTTAC

10261CAGTACCAGCAGGTCCATTAATAGTTACATGATGTTTCTTTTCTTTAATAGCCCTCATAA

10321CGATGTTAAAGGCATTTTTCTGGCCTTCTGTCAAATCATCAAATGTCATCGTAAATTCCC

10381TGCAATTGGTATACTAACAATACGCCCAGTATCTAAAATTCGCTGATATAATCTTTGCGT

10441GTCTACGTCAGGCTTAACATGTTTAACTTCTATTTTATTAAACCAAAACTTGCGTGGAGT

10501CTCAACTAATCTTGGAATTCCCTTACCTAAAGCTAGTCGATACTGCTCTTTAAGAGTAGT

10561AAATACTTTATCAGCAATATTCCATTCAAAAAATACAGCAGGACGATGTTCATCAAGCGG

10621AACTGGCGCCGTAAATCCGTCTTTGTCTCGGTAAACTATCGCATATACATAAACCATATT

10681ATCCTCGGATAAGTTTAAAAATTGAACAATTTAGCGGGTATCCTCTTTTCAGTTTAAGTT

10741TATCAATAAAAGACAAATTTTGATACCGCTTTACACCTTGAATAATTTTATCACACATAT

10801CATATTGCATTTCTGCTTCTGACAACTTTTTCACAATTTTCCAATCCGAACCTTTAAGAA

10861GAACGTTCAGTTTAACAACTTCAGCGCCTTCTGCTATGCGAGAACCATCAATACGAGCTT

10921TAAGTGCTATAATCCTCAGCTTAATGTCAGATGTCTGTTTTGATTTAGAAAGCTGAGAAA

10981TGTGTTCAATTCGGTTTTCACGTTTTTTCTGTATAGCTTTAATTTGATTATAAGTCTTTT

11041TGATTTTAGCCCATTTCTTTTCATCTAAATTTAGTTTATGAACTTTTTTCGCAGATGAAC

11101GACCAATTCGCAAAGCAAATAAATCACGCTTTTCAATCAACTCTTCTAAAGTATAATCAG

11161AACGAAATGTATTATACTTTTTCTTTACTGCAATAACATTCCCTTTAATGTATCCAACGT

11221TATTATCAAAACGTTCTAATGATAATTTCTCTCCTTCAATACGATTATCAAAAGGTTCTC

11281CCGAGTAAGCACAAACTTTTTGATCTAAAATGTTCTTAATGTAATTGAAGTCTAAGTTAA

11341AATCTTTAGAACGTCTTTTTGCAGATGCCTGAGTATGCTCTAGACGACGTTTAATTTTAC

11401GAATTTGGTTATTAGACAGCTTCATATTTTTCTCACATCTTACGGACGGTTAACTACTTA

11461TACTATAACATTTTTACCTTAACTTGTAAACAACTTTATGAAAAATGCTTTAAAACTTTC

11521ATGGTATAATGAATCTAAGTCCTTCCATTATAGATTAAATCCTTCAAAATCAAGAGTATA

11581GATAGTGTATGTTGAATACTTTTTATACTCGTATCTATCCGCAATTCTAAATACACTTCC

11641AGCTGGTATCATTACTTCTTGTTCATCTGAAACTAATTCCATATTACGATAGCGATGGCT

11701ATCCGGAAACTTAAAACTCGGACTATATTCTTTACAACGTAAAGCTTTTATAGCATACTC

11761CTGGAAATTAAACACCATAGGAGCTTTGAATTCAAAAATAACTTGTGTGTTGTATTCTAA

11821TCCAGAAGCAAAATGTAGAGCTATATTTTTATCATATGAAGCTGATACTACTTTATCAAA

11881TGTAATAATATCAATTCCTTGATTTAACACCTGTTTAGTCTCAACTGGAACACCTCTCCA

11941AAGAGGTTTATCGTTTGGAACCAAACGAGATTTGACTATTTCATTTAACCAAGAATGATC

12001GTCTGGTTTATTAGTAATACAATGAATTAAAAGTTCAATTTCAGATAAATTAAACCCTTC

12061AGAAAGTAATTCTTCACGAATAGAAGCACGCACCGATGCATCCATTGATTTTATTTTAAA

12121ATCTTTTAGTTGCATTACTGAGTATTTCATTCAACTACCTCAATATCATAAACTTTAAAT

12181GTTCCAAATGAATCGTGTAATTTTTCTTTTGAAATAGAAGTTATTTTATACTTTCCAATT

12241GGAATCATCCATTCTTGTTCACGTACAATCATCATTAAGTTATCAGTACGTTCTGAATCT

12301AATCCATCAGTATCTTCATATGTATACTTAAATTCAGTATTAGGAGAAGAAAGTATAATA

12361TCGCTGATATGGTCAGAATAATTAAAAGCTTTATCAGTTTTTAAGCGAAATATTATTTCA

12421GTGAAATACTCAACATAAGAAAAACCACATGCTGTGTGCAAACTAGTAGTAAATGAATCT

12481ACTCTGTTCGTTGAAAACACTTCTCCGACTTGTAAATCTTTAATGAGTTCTTTTGTCGAT

12541TTTGATATACCACGATATAGCTGATAAGGCGATTTAGTTAAATGCTTTTTAACGATTTTA

12601TTCAACTGCCGATGAAGAGCTTCATTCTTTTTGGCTTCCATACATTGCCAAAGAATTGAC

12661TGTTCAAAGTCAGTAAATTTTTCACAGACCTTTTTATACATATCATATTGAAAATCAACG

12721CTTTCAGCTTTTATAGATAACTGTTCAACATCTGCAAGATTAATAATCATGATAGCCTCC

12781GTATACTTCAGAAGCTATCATATCATCGTTAGGAAGGAAAGTAAACAACTTTTTGAATTA

12841TTTTGCCCAGGGAGCCCAGGGCGGAGGGTCAAGATGGTATGAAGCTAGTTCTTCTAGAAG

12901AGCATCTGGGGCTTCAATTCCATAATTCTGTAATACTATACGGTACTCTTTCTTATAATC

12961ACTAGAATCATTCTGGATATTCGTAGAATGATTATCTTCTAACATCTCAAATAAATCCAT

13021ATTAATTCCTAGCGATAAAAACCAAAATAACGATTAGCTTCAATAATCTTTCTTTCTTCT

13081TCGGACATTCTCCAGCGTAGTCCAACATCAAAATGAGCCCAGACCATCTTAACAAATGCA

13141TTTATATCTTTAATATCTTCAATAATGAATGATTTACTTTTAGAAGGTTGACTCGCTAAT

13201TTAACTTTATCGTCTTCAAACAGGTCAAAAAGACCATATTCATGGGCTCTTTTATAGCCT

13261TTAATAGTTAAAGCTTCAGAAGAAGAAAAAGGTGAATCAGTATTCTGCAAAATATCTTCA

13321GTATGCTTTATAATTAGAATAACATTTTCTGGATATTTTCTTTCTTTTATATCTTTAATT

13381AAAATATCCGGATTTTCTGCTAAAGGAATAATTAATGAGCATTTTTTACCAAGAGTATTT

13441ACTGATTTACCTTCTTTAGACATAAATTCTATTGAATAATGTTTTGCTACTTCAATCATG

13501CGATTTCCTTTTGCCTACTAATGGACCGTCAGGAATTTTGTTTTCCTGGATATATTTCTC

13561ATTTTCTTCCATCATTTTACTACCGATTTTAAGAAGCAAATCCATTGCTTCATTTGCTTT

13621AGCTTTAGCCTCTTCTAATATCATATCTCTGTTCACGATTTATCCCCATAGATGTCTCTC

13681ATCAATTTAAGCGCTGAGCGTTCTAATTTCTTTTCTTTCTCAGCACTAATCATTGATTTC

13741ATCCATTCTTCCGATTCGTTCTGCATTTCTTTATTTGCTTGTTCAACCCAACCATCATCA

13801ATATACATCGAGTTTGGTCTATTGAACCATTCAAGCATCTTCTTCAGAGCTTTCATTTGT

13861TTTACCTAAAACAACAGTAGGAGCATCATCAAATTCATGAATTTTGAGCAAATTTGGATG

13921TAAACTATTCCATACAGAAGAAATAAAGTATGATAAAGCATCTTCACTCGCAATTATAAA

13981TTTATTTCCTTTATCTATTTTCCAACTATATACTGAATCGTACGAATAAAAAGATAAAGG

14041TTTATTATTATATTGTTCTGCGCTAACATTAATGTATTCTGATTTAGTAAATGCTTCAAT

14101TGCTCTAAACGGAATATTTGGTACAGTTTCAATAATACCAATCTTTTTAAGTTTTATTTC

14161AGCATCTTCTGGAACTGGCATTGAAACGAAATTTTCTACGAGATACATTGGGTCACAATA

14221ACCCTTATCAATCATTACTGCTACGGTAATTGGAACGTCTTTAGCAAACGACATACTATT

14281ACTGCTAATATACATTTCAAACAAAATCGCTTCCATAATTTTCCTCAATCACAAGATGTA

14341GATGAACAACTAGAATCACAAGAACTTCCACATGAATCGCCCGTCCATACATGAACAGGA

14401ACATTAGTATCATATGAATCAGAACTAGACTGTGTATTCTGCGTATTAGATGATGTAGGT

14461GTTGACCAGCGCCAAGGATTTTTATAATATTCTTGGGCTTCTTCATAAGTCATAGTAACT

14521GCTTCTACTGTTCCATCTCCCATATAAACATACTCAACTACAGTTAAAGGAAGATAGTCA

14581TTTGAAATAGGAACTACACCTTCTCCGGGAGTTGTAGAGAAAAAATCCGTAAAGAAACTT

14641TTAAACCAATTAAAGATAAACATTACAAAAAGCCTCTCTTGAATTCGACTTGCTTTTCAC

14701CATAATCATATCGAATCTCTGCATTAAATTCGACAGAACCATCTGCGTACATCATAAATG

14761AATGCACAACAACTTCTGTAGACCATGGCTGTAGTTCATATTTCTTCATTACATGCCGTG

14821AAATGATAATATCTAAATCTTCATTTGGTTTAATCCAACGATTTAACATAGTGCTCTCCT

14881CTATAAGATAATTCTATTATACCATACTCATTTTGGAAAGTAAACCGTTTAAATGAAAAA

14941AGGACTCCCGAAGGAGTCCTTGAATTATTAACCACCTATTTCTGTTGGCGTAAACATTGC

15001AGCATTCTTAGTTTTCCAGTCAGCTGCATCAGAAACTACAGTAGAATATGCTGCCTTTTC

15061AGAAGGAAAGGTCTGATAATGAGAATCTGCAATACGATGCTCGTTAGAATAGATTTCAAA

15121CGGAACAGAAACTTCCATACCTTTAACTTCTTTGCCTTCACCTGCTGGATGAGTAAAGGT

15181CTTGATGTTAACGAAACCACCCATAATAAACTCCTTTATTGTTTAATTACAGGTGTATTT

15241ATATCATCAATAACCTCGGTCTTGACGTGCGAAATTTTCGGCATTTTTCAAATAATAAAG

15301TTTAAAGATTTCTTCAGCTGACATTCCAAGAGCAATAAATTTATTCATGAAAAAGTGAAA

15361TTGGTCAATCAGTTCAAATTTAATTTCAAGCTGATCTTCGGGAGATAAATCTTGAATTTT

15421CTTGGATTGCATTTCAGAATAACGTTTCTTCCAAGGTTTCCATACAGCAGAAGCTTCTTT

15481TTCACCATTACTCATACCACCAAGAGAAGTATATAGCTCGCGAGTTTCGTCTGCGATATA

15541ATCGTCTTGGTTTCGCAGCCAAGCAAGAACTTCTCCTGCAGTTTCAAGTGAATCAGGATG

15601ACGATTTGTTTCTGGTTTATCATTAGCCAAACGAATCTGTAAATGCCGCTGCATATCAAG

15661CATAACCTGCAGTGGGTCAATATTGTGCATAATATTCTCAGCATATGCACGATTTGCTTT

15721ATCAACACCTTCGATCAAATGAGCACATTCATTAAAGTAAGCCATTATTTTTCCTTTCAA

15781TTCATGGGTTAGTAGATTAATTATACAATAAATATATAAAGCAATAAGGAGGACATATGG

15841TACAAAAATTAATGGCACTTGTTAATGCCATCAAAGGTAATAAAAAGCGTATAGCTTTTA

15901CTATTTCTGCTATGGTAGGAATTTTACTCTGGAACTTTATTCTATCACCTGTTGCAATTG

15961CACATGGTATTAATATTCCAGTAGTTACTCTTGATACATTCGTAGATTTAGCATTTGCTT

16021TAGTTGGGTTAATTTAAATCTTGGCATATTTAGATAGCCGCATTTTAGCCATCAACCCCT

16081GTGCAATATTATTTTTCATATATTCCATAATTTGTTCAGGGGTTGCACCTTCCTTTCTAA

16141TCATATCATTAACATCTTTTGATTTCCAGGGAGATTTATCCCAAAACATAACCCTTTCTC

16201CTGCATCAACTAATTTAGTCATTCGTTTAATAGTGTCAGGGTGACGAGGTTCATTATCTA

16261AGACCCACACACGTCTATCTTTAAATGGAACAACTTCTAGGTCTAATTGACCACCCGTAA

16321TAGCTATACCATTTTCAATAAAAAGTGAATCTATAGGTCCTTCTAGAACATATACATCAC

16381CATCTTTAACTCGTTCGACTCCATAGATTTTTGTTGCCTCAGGATAAGCTTTGATGGTGA

16441TATATTTTTGAGGAGCATCTTTCTTTAATGCACGCCCTTGAAACGACTCGGCTTTTCCGT

16501TAGCATTATAAATTGGAATAACAAGACGAGGCTCAGAAATTTCCTTTTTGTATGTTCCCG

16561GTGCTATGCTATTAACTAATTTAGGCCATTCGGTTGTAAACCAAAGATATTTCCATTTAT

16621CCTTTGGAATACAACGAGCTTTTACATATTTTATAATTGGATGATCTTCCGCCAGTTTAT

16681CTAATCTAACACATGACGGAAGAGATTTAATTATTTTCTTCTCAGGTTGTTTAGGAAGTT

16741CTTTAGGTTTTTCTACTGGACGACTTTTACCTTTTTCTTTTCTTATTTCAAAGATATACT

16801CACGATATAAATCAGGTTCAAACTCCTTTAAATATATTCCGATTGGTGCATGATAGTTAC

16861AGTTATAACAATGAATATTTCCTTCATTATTATCGCCATAATACCATCCACGAGCTTTAT

16921TCTGGTCGGTTTTTGAATCTCCACAAACAGGGCATCTAAACCGCAATTTAAAAGTTGAAC

16981TATTATTTACTTGTGTGAATTTAGGTAAATGAGCTAATGCACGGTATGCAAACTCATTAT

17041CAATCCAAGGTATTGATGACATTTTTACTCTTCTTTTTCTTTAGATTCCTCTTTTTTCTT

17101TTTAGGAATCTGTTCAGGACCTTTATTTACTACAGCGCCTGATGTTGTTCCAGTAGAGAT

17161ATTTTCAGGATTACCACCCGAATCTCCAGCTACCATATCTTCTTTGATAAATTCTTTATA

17221TGTTTTCATATTAACCTCTATTCATAAAAGCATTAAAAATTTGGTCATCAATAGATGGAA

17281TAGCAACCTTTTCGGCATCATTTAGCAAATCATTAATTTCATCAGATAGCATAACGCATG

17341TACCTTTATTTCCTGTAATTGTTACTGCGCAATTACGAACGTTTTCTTTATCAAATTTTA

17401GGTTAATATTTTCGTAGTGAAAATCCTTTAATGCCGAATATATTTTTAAAATGAAATTGT

17461CTTTTACTACAATAAACCCAACCTCACTATTAAAACGTGGTTCATGGAAACTGTAATAAA

17521CCTTTTCATCTATAAAATCGTATGAAATTTTTTCCATTTTTAAATATGAAAATTCAGAAC

17581TATACATACTAACCTCTAATTACTCACCTACCACTTCAGCGATGATATTTTTATTATTAA

17641AGTTTTTATCACAATACAGAACGTAATTGTATTGCATTACACCACCAGACTTAAGCTGTT

17701TTTGCACTTCAGCTTTCATTTCAGGGCGATCGCGCTCAACGATATTCATAATATCTGCTT

17761CAATTTGAGTTTCAACCTCAGTCTGATCAGCAGTCATAGACCACTCACACAAATCTTTAT

17821CATAACCTGCCATAGCAGGCTGAGCAGCACAAGAAGCTAAAGCAAAAATTGTAGCAAAGA

17881TAAATTTTTTCATGATAATCTCCTCAGTAGTTTATGTTTATATAGTATCTCAATTTCCAA

17941CAAAAGTAAACAGTTATTTTAAATTTCTGCGTAATCACATGTTACAAACTGTTTCTCTAG

18001CTTAACGATCTTACGAAAATACCTTTTACACTGACGAATCTGTCGCTTCGTAGGACGTAC

18061AGCAAACTTAATAAATTCCACTCGACCAAATGGAGGACTTTCTTCTGCTGGAATATCTAA

18121CACCAATTCCCACGTATCCGCAATAAGTGCTTTGAATTGAGTATTTTTCCTGACGTTATA

18181CGGAGTAGGTTTAAATAAAACAATATGCATATTATCCTCGGCAATCTACTTCACATACTT

18241TCTTGTCATCAATGAAAGCTTTAACTAATGCTTTATTAACTTCAGCATACTGAGTAGTAG

18301CCCATTGAACGTCATCTTTCATCATTGTGGTTTCTTTAGTAAACATGCTTTCATTCTTAA

18361ACCACCCCATAAAAACTACCTTTACCAATTCCATAACAATCTCCTCATTTAACCAACAGG

18421ACTACTATACCATAGTCTTGTCAGTTTGTAAACTAAAATTTTAATTCATTCGCCAAAGCA

18481TCTAACTGAGCTCGAGTGGATTCATTTCTTTGATAGCGGTTCTGTTCAGCCTGAATCTGC

18541TGTGAACCTGCTACTTCGTTCACTTCAGTTGGAGTAGAATCTTGTTCAATTTCTACCCAT

18601TTCTGATTTCCTTTTTGAACACCCATCAAAAACTTATTCCATTTATTCTTATCACCATAT

18661CGTGATTTGATTTGCTTAATTAGTTGTTGTTCAGCAGCTGCTAACTCCTCGGTTTCAATG

18721ACCGCAAGCATAAAATCGGCTGTTGCTGGAAGACCGGCAGATTCTGCAATATCGCTCATG

18781TTAACATCAGAAGAATCCCAAGCTTGTTTACCAACCTGTGCTGCAGTCCAAAGAACAGTT

18841TCGGTTTCAACAGCAAGAGCACGCAATTCCTCTGCAATGGCTTTAACAGTTGTGTAACTA

18901TTTTCTGAGTAAACTCTAATGCGGCAAGATTTACAAATACCCAGATAGTCGACAATAATG

18961ATTGTTGGAACAAAATTCTTTTTGAGCTTCAATTCATTCAAGAGAGACCTGAATGTGTTA

19021GCATCTGCCCCACCAGTAGGGTACTGTTTAACAATTAAACGACCGAGAGTAGATTTCTCA

19081CGCCATTTTTCCATTTTTCCTTTATATTCAGCGTAAGAAATATGCCCATCATCAATGTCA

19141TCAAGAGAAACATCAAGCATATTAGCATCAATACGTTTAGCGCAGACTTCTTCTGCCATT

19201TCCATGGAGATATAAAGAACGTTATGACCAAGCTGCAAATAATCTGCTGCCAATGAACAC

19261AGACCTAATGACTTACCAACGTTAACACCAGCCATTAAAACGTTCAGTGTTCCAGTCTCA

19321GCTCCGCCTTTAGTAATTTTATTTAGAATTTTGAGTTTAAATGGAACCTTACGAGCTTTA

19381TTCATATAAGATAGCCAACGTGCTTCGTAGTCATCCATCCAATCATGACCAACGTAGCTA

19441TCAAATGAAATTGATAATGCTTGGCGCATGATGTCAGGAATAGCACCTACATCTGGCATT

19501TTCTTATTTCGTTTTTCCGGAGGAAGCTCAGCATTAGTTTGAATTTCGATTATTTTAGAC

19561GTAGCATTAAACATCGCCCTTTGCTGAACATATTTTTCTGTTTCTTTTACTAACCAGCTG

19621TGGTCTTCTGGAGAATCAGCTAGTTTTGAAATAAGTGTTTTTACACCAGAATATTCTGTT

19681TCAGTAAATGAACTATTTTCTAATGCAACATTTAACGCATTAATAGATGGAACGCTATGG

19741TACTCATTAACATGAGATTTAATTAATTTGAATGTATTTTTAGCTGGACCACTTTCAAAA

19801TATTCTGAATCCATATATGGCCAAACTTTTGAAAAATAAGCTTGATCAAATATAAGATGA

19861GAAAGAATAATTTCTACCACACTTACTCCTTAAAAGAATTTAAATTTTTTCTTTGACCTT

19921TTATTAAATGCATCTTGTAGTTGCATTGTAATACATTTTTCTACATGAGGAGCTAACTCA

19981GCTTTTCTTTCTTGGTCAAGAACAGCAAAGTCCATTACAACCTTTCCATTAACCCAATCT

20041AATTTTGTCACATACACAATGTGTGTAGAACCATCTTCTAGTTTAATGACAATCTCCTGG

20101ATAACATTTTCCATGGCAGATTTAATTATCTTAAGAGACTCATTAAAAAGACGTTCTTTT

20161CTTTCTTCTTCCCCCTCCGAAGAGGGGGATTCATCGATAATTTCTAGATCTAAATCTAAA

20221TCATCTTTATTCATTAAATTCTTCCATATCACTTAACTGTTCTAGGTCAGTTTCTAAATC

20281AGCTGCTGATTTACTTTTACTTTCTGGAGATTTAAATTTTTCAACCTTTGAGTTAATCAA

20341TTCATCAACTTCAGCTTCAACAATTTCATTACTATCAATAGCACCTAACTGATAAGCACG

20401TTTAATAGCATCTCGGAATGGTTGATGCTTAAATAAAGGACCCCAGAATGTAGTGCAGTT

20461GGTATCTTTTGCACGCCAAGATTTTTCTTCGCGAATCATCTCGCCGGTTTCTTCATCAAG

20521AAATTCACGAGCATACCAGCCATTTTTAGGTTTTACCACGAATCCTAATTCTAGAGCCAT

20581ATCTAACAATCCAGAATAAGGATCGATACCACCGTCAAATTTAACATCAATAAAGAATTT

20641ACTTTTTTCTTTAACGGTACGAGATTTTTCTACATTTAGAACAAATTGATACCCCTGAAG

20701ATCAGAACCATCTTTAATCTGACGCTTACCGATAATGAATACGGTATCAGCCGAATACAT

20761TACGCCTGTACCACCTGTCATCACGGTTTTACTAAACATTTCAATTGTTTCAATTGTATG

20821GTTAACCGCAACGCACGGAATATTTTTAATGCTAAAATAAGGAGTAACAATACGGAATAA

20881TGACTTCAGTGATTTAGCACGAGTCATATCTGCCACAGATTTTTCATTCAAGGCATCTTC

20941CGTTTCTTTCTTAGAAGCCATATTACCGATTGAGTCGATGAATACAATAACCTTTTCACC

21001ACGCTCAATAGCTTCAAGCTGATTCACCATATCAATTTTCAGCTGTTCAACTGACTGGAT

21061TGGCGTATGAATTACTCGTTCCGGGTCAACTCCCATGGATCGCAAATAAGCTGGAGTAAT

21121ACCAAATTCGCTATCATAGAATAAACAAACCGCGTCAGGATATTTGTTCAAATACGCCGC

21181AACCATAGTCAAAGACATATTTGATTTAAAGTGTTTAGAAGGCCCTGCGAAAATAGTTAA

21241ACCAGACTGCATACCGCCATCAATCGCACCAGAAATAGCAATATTAAGCATTGGAATTTT

21301TGTACGAATTACATCCTTTTCATTAAAGAATTTAGATGTAGTCAGTTCAGCAGTCATTTT

21361AGAAGTGGAAGCTTTAATCAAACGGGATTTTAAATCTGCAATAGACATTCATTTTTTCCA

21421TAGGCATCATTATATTTTCCTCACTGGTTAAAGATAGAGTAATTATAACATAATAAATTT

21481AGGCATTAATCAACTGCTATTGGATGAATAGCATTAAACTTATGAAATGCTTCTGATTTT

21541TCTTTACGCGAAACGCACATGCGAAGAACCTTTAATGGTTCGTCTTCTTCACCCAACGAT

21601TTTCGTTTTTCAATATTAGAAGTTTTCCAACGAGCTTGCTCTGGAAACTCTCTATTGATT

21661TGCTCAAGCGCTCTATTATGTTTAGAATTACTACGAATTGAACTGCACCCGCCAGGAGCT

21721TGTGCTTTTCCAGATACAACCAGATATTTGAACAAGGCCAAATGCGGATAACCTTGATTA

21781ATTAAATTGAGAAATGCATACATATCTTCGCACAAATCAATTTTTCCATACCCAATTTGT

21841TCTGTCGTAAGTTTTCCAAGGTCATACCATGTATTCGTGAATCCATATGAATTTTCACGA

21901TAATTACCCCAAGATGAAGTAATTTTAAAAATTGGTAGACGAGCATGACCGTGATAATAC

21961CCGCAATCCATAGCATCTTCAACGTATTGAATCAATTCGTAGAATTGTTCACGGGTTAAT

22021TGATTGACTTTATCTACACAGCGACGATCATCTCTTTTTCGCATTGAGCTCATACGAATC

22081GTAGTATCATCGTCAATCATCCAGATTCGTTGACCTGCATACATATCAGTAATTGCTTTA

22141CGAGTACCGGCAATTCCATTTACGTCATCAGGAATAGTTATAATTTTAGCTCTAGACCCA

22201TAGGCATCATAATAAGCTTTTTCTTCATGTTCACGCACTACAATATGGGGTTCATAATCA

22261GTCGGAAACATATCAAGGGCAGAAACTGCCCCGACCCGCTGATAACTTGGAATTACGAAT

22321TGAATCATTTCCACTCACCGTTATAATCTTTTTTCACAATATGAGTTTCGCCAGTATTCC

22381ACCAATGATCAACCAAGTAAAAATGACGAGAATATACATGAAGACTTCCAACATTCCATA

22441TAATAGAACCTGCTTTATACTGACGAGTTGAATCGCCGGCATTCAAATCAGATACTAATT

22501TATCTAATACGTATTTTTGCCATGCATAATCATTACGGAATCCGAAGACCACGTCATTTG

22561AGCGCATGCTTACTACTGCATTGACTTTCTTATCACGAATCAGGTACTGTACTGTATTAG

22621TACACATGAAATCTGACATACCATCTTTATTGTAGTCAAACTGCATAGATGGACGAGTAT

22681AAATCATGATACCACGTCGAGAATCAGGATTTTGACCAAGTTCAGCTAAACACATATCAT

22741ACTGGGCATAGTTATCTTCTGACCAGATAGCCCAACCATAATTCGAGTTAATTTCACCTT

22801TAGAAGATGCTACTTGTTGCCAAATCTTTGGTGTTTCACCTGGAATATCTTTAACGAACA

22861AGCTTTTAGATTTATACCATTCAAGTTCACGCTGAATGTATTCATCATTAAGAGCGCCAA

22921AAATAAACGGTTCATCTGCTACAAATGATGCGCCAATAATTTCAATAGTTTTAACACCAG

22981TTTTATCAACTACGAAATCTTTTTCTTTTAATGCAAGCCCCAAATGAAGACGGATTTCTT

23041CAACTGTCATAGAGTCACTAATCATTTAAACCTCAATTGATACATTCATATTTAACTTGT

23101AACAGTAATAAACCCCAACCTAAAATAATAGTTGGAATCATAAGAGGAACCGTTACACTA

23161TAGTATATACTTATTATAATCATCAAGATTAAAAGCAACACTGCTATAATTTTGCTTTTC

23221ATTCCTTCTCTCTGATGATAATTACCTGATTTGGTTGTGCAGACTTTTTAGTTTCACCCG

23281CAATTGACCAAATAAATGTAATAAACCAACCAATAATTGACCAGTTAAACAGTAAAGATG

23341TGAAAAAGATTCCTACTGTCGATTTTGACCCACGCATCAAAGCAATAAACCATGGAAGCA

23401TGTATATAATAATAGCCAACACACCTGAAACTAAAACCATAAAAATTGAACCTGCTACTA

23461AAGTTTCCATGTTTTCCTCACTTAGTCAAATTTTTTACACATGAATTATAAGAATTCACT

23521ACATACTCCATCGGAGCATTTTTACCGGTACGCCATTGGTAATTATTAGCCCAATTTGCC

23581CAAAGCTCGGCGCAGTAGTTTTCAATTTTTTCTTCGCGTGTAATTACATCAGAATTACGA

23641TATGCTTGAGCAGATTCATCTGGACGAATAGCCTCGTCAAAATTCGCCTGCATTTGTTCG

23701ACTGTTTGCTTTGGAGCTTCTTTATAACACTTGACATTAGGATTATAAAATTTGCTTGAA

23761CAGTTTACAATTTTTCCTACATCAGACTGATTTACTACCGGTCCTTGAGCTACACAACCA

23821GTAAGACCTAATGCAATAACCAAAATAGCGATTTTCATAATAATTTCCTCAAATGCAAGT

23881AGTAATTACTCCAGTAGTGCTTATGCAGGTATTACCCATTTGCACACCTAAAGATCCATT

23941TGTATTCACATGAAGATTATCATCAATTTTAACTGATATTTTACCATTAGTGTGAATAAC

24001GGTGCTAGATTCAATCACAGGTTGAGTATCATTTTCAACACTAACAAATGGAACTGCTGC

24061TAATGCAACTAAACAAACTGCTAAGCATCCCATAACAATTTTCATTTCATTCTCCAAATC

24121CGTATCAGTAGTTGATAGTTGTATAGTACCATGGAAGAACATTCTTGTAAACAGTTTTGT

24181GAAAATTTTTTAGGGAATTCTAAATGTCCAGAATCATCTGTTTTTCATAAGTATAGATTT

24241ATATTACTTGTATGAAAAAGGGACCCGGAGGTCCCTAGATTTATTCTATCAGCCAAACAG

24301GAAGTCTAACGAAGCTTTTTCTTCATAGTCCATACCAGCCGATTCACACATGCCCGCAAG

24361CGGTTTAACAAACGATTTTTGGAACAAAGTTGAGTAGTCAATCCAAGACAGTACGTCAGA

24421ACGAATTTCTTTTGGAAGTTCTGTACCCGACGGCCAAGCAATGCATTTGTCACCAAATGG

24481ATTTCCTTCACGCAATGGAAGAACCATTACTTTATTTCCATCCAAAATTGGAGCTACACC

24541TAAACCGCTAACAGCTCGACGATAAGTTAGCACACCACGAATATGGAACGGACATTTAAA

24601TCCTGGCCAACCTTTATCATCATATTTCGCTATATCGTTCGCAGTTTTTACTTCAGCAAT

24661AACTTTATAGTCGAGTTGACGATATTCTTTCTCGAAGTTCTTGTAATATTCTTGGACAGA

24721CTCTTCACCTTCCTGAAGAATACGACGAATACTTTCTTCGAGAGCTTCTTGTACTGCTTT

24781TGGTGTTGAACTCTGCTGAGTTTCCATACCCATGATTTTTAGATGCGGTTCAGCAAATCG

24841TTTATCTTCCATATCATAAACGTTCAGAGCATAACGCTTTTTCGCTTTCCAAAATCCACC

24901AATACCCTTTGAACCAAGTGGAGGGCAAGAAATAGCTTCACGGTCCATATGCATCAGATG

24961CTCGCGGTTATTCATATAATCACATAACTCACGATATGCAACATCAATCATAGGTTCCAT

25021CTTTTTCTTACCGAACTGATTCATAAATTCAACCAAATCGTTCTGCTCTTTGAATCGGTC

25081AAGACCAACTTTTTCAATAACTTTATCTACACAAACATATACCGAATCAGTATCACCTGC

25141TGCAATGAAATCTTCGCCATTAGTTCCGCATACTTTATTCAGATATTCATTAATTTTACG

25201AGCAATCCACTGAATACCAACTTGGCCGAAAATTGTGATAGCAGTAGCATTTCGCAAATC

25261ATAGTAACGGAAATGAATATTACCAAGAGCACCATAAAGACTGTTAATAAGAATTTTACG

25321GTTCAGCTGATTTGTATTAGCAAGTGTAGCTGCTTTTTCACATTCTTCAATCAGACTATT

25381GAGAACAGATTCAGTATAATTCGACAGTTCATTTAAGAAATCATCACTGAACTTAACATA

25441TCGTTCAACTTCTGGTTTAGTTGAACAAGACCCTGCGCCTTTCATAATAATCTTTTTAAT

25501AGCTTCGGCATTCATTTCTTCAGCGAACATTTTCTTTTTCCAGTCTTTACGCTGGAAAAA

25561TACTTTAGCGATTTCCTTTGGAATGATACCTTCTTGATGCTTATCATACATCCATCCATT

25621CGGAGAACAAGAATATTCTTCACTTGGCTTAGGAGCTGTTCCTGCGATATATTCATGAAT

25681TGGATGAACTTTAAATTGTCCACGAATAGTCTCAGGACTAATGTTAACCTGACGAATAAT

25741GCTCGGATACAGAGACGTCAAGTCAAAACTCATAATATATCGACGAGCAATTGGTTTAGG

25801TTCAAACACAAATGCACCCGGAAAACTCTGTTTAACGTGCGAACCTTGTTGAGGAATAAC

25861CTTATGTTCACCTTTCAATGAGTTAAAAATAATAGCATCCCAAGTTTTAATAGGACTCAT

25921TACACCAGAAAAAGGCATTTTAGCATAATAAGACATACTTAAAACTAGATCGATAAACCC

25981GCGAATTTTATCAATTGCTTGAACTGATTCTACGTCAATGATGTTATAACTAATGTATCG

26041TTGATGATTAGTCTCACGGAGTTTATTAATAGGACCGTCGTATGGTAATTTACCTTTTTT

26101GGTTTCATGTTGAGCAACTGATTCCAAAGAGAATGACGGCAAATTAGTAAAAGCGAATTT

26161CTTGTACAAATCTAAATAATCAAGAATAGATACGCCATCAATAGAATAAATTTCTTTGCT

26221ACCGTACATATTTTGAATTAGTTTAGATTTTACCCGACCGATTGGAGAGAAACGTTTCAT

26281ACTACGTTCACCTAGAATCATTTTAACGCGATTCATGATATACGGAACGTCAAATCCCTC

26341AATATTCCAACCAGTAAAAATAGCAGGTCGTTTCTGTTCCCAGAGATTGATATATTCCAT

26401GAGCATATCACGCTCATTATCGAATGGCATATAAATTACTCGGTCAAGAATTTCTTGAGG

26461AACTTCATCACCACCTTCACAGTCAAGCTTAGCAGCTAACTTTGCATCCCATTTTGATAC

26521TGAACCGTACATTGAATTCAAAAGGTCGAAAACATAAAAACGATCATCAATTGAATCGTA

26581ATGAGTGATAGCATCAATTTCATATTCAGCTTTCATTGGGTCAGGAAATTTATCACCAGT

26641AACCTCAATGTCACAGTTAGCTACACGAACAAATTTTCGGTCATAAACAATTTCTGAACC

26701GTATGTATCACTGATATAAGCGAGTTTAAAATCGTTCATACCGAGAGCTTCGAGACCGAT

26761GTCTTCCATTCGTTTCATCCAATCTCGAGCATCTTTCATTGATGGAAATTTTTGAGGAGC

26821ACAATTTTTACCATAGATGTCTTTGTACTTTGACTCTTCCTTACAATGCCTAAACATAGT

26881CGGAAGATATTCTACTTCACGAGTACGTTCCTTTCCGTTTTCATCAATATAACGTTCAAC

26941GATGTTATTTCCGACTGTTTCGATAGAGATATAAAATTCTTTCATAGATATTCCTTAGTT

27001TATAGCCCGAGTTATTAGGCTCTTGATATATTATACTCCAAATAAGGGGCCGAAGCCCCT

27061TGCTTAATTACCAATCGTATATTTAGGAACGAGTTTCCATTCATGTTTTTGTTTAAAAGA

27121AATAACTCGGAAGTTATTAGTTAAATCTTTCATAAAAGTTCTTTGACCAGGAACGATTTC

27181AATCAGTCCCCAATCTTCTAACAGCCATGCAATCGAATCACGACGAACTTCATCTTCTTC

27241TGTCATTTCAACTTGACGGCCATCCATACGAAGCATTTCTTTAAAATGAACGATATAGTA

27301TAGTCCTTTTTTCTGAAGAATATGACAGGACTGATACAGAACTTTATCTTTATTATTAGC

27361AATTCCCATACGAGTCAAAGTTTCTTTTACTTTCAGAAAATCTTCAGGTTTTTTAAGAGT

27421AATTTCAATCATTTTACCATTCCAATGCTAGTTTTTTGAGTTGTTTCTGTTCTTTTACGT

27481TCTTAGTCACTTCTTTCAAAAAATCATCCGTGACTAAACCTTTTAGTTCTTTTAATACTA

27541AAGGAAGTTTTCCATTTTTAGTAAGAATTGATTTATAGTTAATTGCATCATTTGTATTAA

27601CTTGATACCGCTTAGCAAGTAACTTAATAATCAATACTTCGGTGGAATCTTCAACCAGTT

27661TTGCCCATTTACCATATCTTTTACCACGAGGAACTGCAGCCATTAGATAATTAAAATGAG

27721CTTCATCACTTAATCCAGAACCGATTAAATTCATAGCATATACAGCTGGCATGCACTCTG

27781GAAATTGTGATAATGCATTTTCAACCATGAATTTTGAATAATCTTTTTGAGCAATAGAGC

27841ATTTAGTTTTATTATTAATAGCTCCAATTATTTCAAAAAATTCATTTTCAGCTTTTTCTT

27901TAAAAGAATCAGCAGCGGATTGGACAGCTGTCCAATCTTTTGAATACCAAGCAACTTGAT

27961GCTCGTTTAATTGAATATCATCTTCAAATAAGCTCATATCACTTCCACTGCATTTCGCAT

28021GCTAATTGAATGAAAAGATAAGCTAAATGCAATTCAGTATTAGCTGCAATACCATGATAC

28081TGATTATTTTCGCCTACAATTTCGTACATACGAATAATACTCTGCGGAGTTACACGTGAA

28141TAGATTTCTTCGGCAAGTTTACCAACGAACCACGAATAATCAGCTGCATATTTTGGTGCT

28201AAAGCTCTGAGTTGTTTAACATCTTTATTTTTGAGAGACTCAAGAACATCATCAATAGCA

28261CCACGATCGTTAGTAACCAGTGATAAAATACCAGCATCCAAAACACCTTTAGATGAATAA

28321CTATCGAGCTCGCCAATAGTTTTACGAAAATCAGGAAAATTCTTTTTAACCAAAGCTGCT

28381ACAACTTTCATATCAGCTATAGCAATTCCTTCATGCTTACAGATTTCAGTCAATCGACGA

28441ATCATCTGCTTCATCATTTCAATTTTATCTTCATCGGTCGGTTGACCGAACGTAATAACT

28501CGGCAGCGTGACTGAAGTGGTTTAATAATACCATCAATATTATTAGCAGTAATAATAATA

28561CTACAGTTTGAACTATAAGCTTCCATAAAGGAACGAAGATGTCGCTGAGACTCTGCTAAT

28621CCTGAGCGGTCAAATTCATCAATAACGATTACTTTTTGACGACCATCAAATGAAGCGGCG

28681CTGGCAAAATTAGTCAAAGGACCGCGAACGAAATCAATTTTACAATCTGATCCATTCACA

28741AACATCATATCAGCATTTACATCATGACACAATGCTTTTGCTACAGTTGTTTTACCTGTT

28801CCTGGAGAAGGAGAATGAAGAATAATATGTGGAATTTTACCTTTACTTGTAATAGATTTA

28861AAGGTTTCTTTATCAAAGGCGGGAAGAATACATTCATCGATAGTAGATGGACGATATTTC

28921TGTTCAAGAATGTGTTCTTTTTCATTTACGGTAATCATAATTTCCTCATTCAAGTTTTAG

28981TGTAAATTATAAAGGGCCGAAGCCCTTTATTAAAAATCGTGAGTAGAATCAGCTTCAAGA

29041GCTACTACATAATTCGCATGTTCACCTTCAAATTTAGCAGCACCTTGTTTACCTTTTGCC

29101CAGAGCAGAAGTTTATAATTTCCTGGTTGCATTTTCATATTTGCCATATTGATAATGAAA

29161TTAAATGTATTTTCACCATCATAATCACCAAGAGTCAAAGAATATTTAACACGGGTCAGA

29221GCAGAATCTTCTACTTTATTAAAACCGTTAATTACGATTTTACCTTCTTTTACCGTGATA

29281GCAATTGTATCAATTTGCAGACCACGAGATACACGCAACAGTTGTTGAAGGTCTTCAGCT

29341TTAATTTCAGTAACAACAGATGCTACCGGGAATGGAATTGGTTTATTAGGAGCAACTACT

29401GTACTCGGATCGGCTGCTGGCCAAAAAATTGTTGAGCGGGCATCAGCAATTTTAATATTT

29461CCATCTTCTGACTGGGAAATTTCTGCATCATCATTAACTAGAGACAGAATACCGAGAAAA

29521CCGTTCAAATCGTAAATTGCTACATCAAAATCAATAACGTCAGAAATATTTGCTTCCGCA

29581TAAGTTGTACCATTAACTGCGCGAGTCATAATAAATTGACCGGATTTAAGCATAATACCA

29641GAATTAATAGTAGCGAAATTTTTAAGCAGAGCAGTAGTATCTTTAGACAGTTTCATGTAA

29701TTTCCTTCAATTCAAATGAGATTTAATTTTATAACTAATTTAATAAAGCAATTAACGATT

29761AAAATCAGTTGCGATTGTTTCGGCAACAATTTGAGCGGCAACAATCAGACGTTCATCTGC

29821ATTGCCACAATATTCATCTTCAAGGCGTTCACCACATGAAGTCATAATAAATTTAGCACC

29881AGCGTTTAGGGATTCTGTAGTATGTTTGCGCATTAGTTCAATCCATTTATTACTTACTTC

29941ACGATCGATAGCTTCATAATATGCATGACGAGCAGGTGCAGATTTAATTTTGTTCTGAAT

30001AACTTCCATTGCGTTATCAGAAAGAGACAAAACCCATGCTCGACGAATTTTATTTTGGTT

30061TTGTGGATTTGATTCAGAACGCACGTGTTTTGGCTGAATATCTTTTACATCAACAGTATA

30121ATTCACAGTAATTTTAGTCATAATACACCTTTAGTCATAATAATCAGTAACAGTCCAAGC

30181TTCATTTCTATTGGACATTATTTTTGTATATTCTGCTTTAAATGCATTCCTAAGCATAGA

30241TTCAGTAACTATATGCTCTTCATTAGAAAAATTATTTCTCAGAATATATCGTTTTATTTC

30301AGGAATAGTTAATAGATGCTGTCCGGTTGAATATTCCATGTTTTTCCTCCATAGAGATTA

30361TACTCTAATAAATTAAAGCATAATCTCTTATAAATTAAACCATTACAGTAAATCGACCAA

30421CTTTCTTCATTTGAAGATGCTGACCGTATGCTTGCGGGTCATGGTCTCTATGCGAAATAA

30481CAAAAACGTTAGTATTTTTTAAACTATCTAAAATAGTTGAAATAGCTTTTACACCTTCAA

30541CGTCGGTCGCTGAATCAAAAACTTCATCAAGAATTAGTGTGTTTATTTTAACACCTGAAA

30601CTTTTTCAGCAATATCACGCCAAGTAAATAAAAGAGCAATATCAATTCGTGCTTTTTCAC

30661CTTGACTGAATGAAGCATAACTAAAATCTTCACGACCACGGGATTTAATTGTCTCATTAA

30721ATTCTTCATCTAATGTAAACACATAATCCGCTTCCATTATTTTAAGATAATGGTTAATCT

30781GCTTATTAAATAATGGAATGTACTTTTTAATAATAGCACCTTTAATACCAGAATCTTTGA

30841GCATATCAGTCAAAATTCCTCGGTGGTATTTTTCCATTACTAAATTAGTTTTTGTCTTAA

30901CAATTTTATCAAGTTCTTCTTGAAGCAGTGCTATTTCATCAGCATGGTCAATAAACTCAG

30961AAGATGCTTTTTCTATAGCTGCTTTAACTTTTTTGGCTTTATCTACCGTCGTGATTAGAG

31021ATTGCTTTTTATTGCGAATATCATTTGCCAACGACTGTTGGGTTTTAATATTATCTCGGT

31081ATTCATCAACAAGAACTTTTAAATTATCACGATGTGTTGAAAGCTGTTCAAACGAATGCG

31141TACATTCAGAAACTTTATCTTTAATTTTAGAAACAACTTTATCACCGGAACTTAATTGTG

31201ACAAGCAGGTTGGACATAATCCACCTTCGTGATACATATTAATGACTTTATTATACGAGT

31261CAATTTTTGATTTAATTAAAACTGCTTCTTGACCGATTTTATTAAATGCATCAGTCGGGT

31321CTTCGTCTAAAACAATATTAACTAATCTTTCATTAGCTTCTTCTATTTCCGATTTTAGCG

31381TTCTAGCTTCTTTTGCCAAATCATCATACATATTTTGCAGACGAGTAAGGTTGTCACCCG

31441TTAATTTTTTCTGGCGTTCAACGTTATCATTATATATTTTAATTTGTTGGATAATACTAT

31501CTTTTTTAACATCAAGCACTTGGTTTTGTGAATTTAATTCGCGTATTAGTGCTTTATTAA

31561GCTTATCCATTTCAGCTAATGTTCCTACCTCAAGCAAGTCTTCCACAAGCTTTCTTCGCG

31621CAGGGGTCGACAAACCCATGAAAGGGGTATACCCTGCTGTACCAAGGACAACAATCTGCT

31681TGAAACTGGCATATGACATTCCGATAAGCTGTTCAAATTCTGCTTGGAAATCTTTACTGC

31741TGGCAGATTCATTAAGACGTGTACCGTTAACGGTGATTTCGAAAACGTTCGGTTTTTGTC

31801CTCTTTTGATATAGTACTTTTTCTCATCATATTCCATCCACAGTTCAACTAAAAGTTCTT

31861TCTTATTTGTGCTGTTTATTAATTGACCTTTCTTTACATCACGAAATGGCTTGCCAAAAA

31921GCCCAAATGTGATGGCTTCTAACATAGTAGACTTACCACCACCATTTCGTCCAGTAATAA

31981GAGTTTTTTGAACCTTATCTAATTGAATGTCAATAGGATTTCCACCTACTGACATTATAT

32041TTTGGTACCTAACTCGGTTTAGTTTAAAATTCTTCACAAAAGATTCCTTTTAATGTATCT

32101TTTAGACCATTCTATCATATCATCATAATCTAAAAAGTATTCATCAAATTCAGCCATGCA

32161AACAACACCTTGTGCTGTTTTTGATGTAATATAAATTATTCCAACATATCTAGAATCTTC

32221TTCGGTATAATCAATGTTTGCTATGAATTCATCATTAATGTCAAATGTCGAAAACTTCAC

32281AGTATGCATCCTTAATACAAGATACGGCCATATCTCGCAATGATTTAGGTGTGTCATTAT

32341CTAAAATGTTGAAGTTAAAAGATACAGCCCAATCTGTACAGACCGTAACTTTTTTAATAC

32401AATTATCTTCAACCTCTAGCGGTTCAAACCAATATTCAATAATTTCTTTATGCCCAATAT

32461CATCTTTTACTTCACATTCAAAATGCTGACTCATCATAACATTTTTAAATTCATCAAAAG

32521TCATTGTGTTGCCTCTACATATAACTGATTTGCATATTGAATAAGTGCTTCACGGTCAGA

32581ATCAGTGATGTCTGGAATTGCATTAATGTATTCTTCCATCAACGTCTGAAGAGATTGAAC

32641TTCAACTTCTTCACTGTCATCTGACTCGACAGAGTTATCAATCTTTGACACAACTCGTAA

32701TGAATGCACAACTTTTTCTAGTTCAGATTCGAACTTCGTCAGATTTTTGTCTACTTCAGT

32761TACTATAACACGTACTGATAGATTTGTAAAATCTTTATAGTCAATTTTTCCTTTAAATGG

32821ATAATGAATTCTACGATGCCAGGTAGTATTGTTTGGAATAAATTCCATTCGTTCTGTTTC

32881TGTATCAAACATCCAGAACCCACGAGGGTCATTCTCGTCACCTGCAGTTAGTGTCCATGG

32941TGTCCCAATATATCTGACGTTAGCAGCCTCAGAGATAGTATGGAAGTGACCAGACCACAC

33001CTCTTTATAAGTCTTAAGGAAATCAGGTTCAAGACCGTGAGATTTCATTCCTTTATAAAA

33061ATAAAATCCATTCAGTTCCCAGTGACCAACACAAAAAGAAGCAGATGAAGTTTTGATGTG

33121CTCAAGAATTTCACCAGTATTTTCTTCACACATCCAAGGAATTAAATCAATTAAACATCC

33181GTCAAAATCTACTGTAGTAGGCTTATCATATACTTTAACATTAGGATATTTAGCCAAAAG

33241CTCAGTAGAGGCATTTGGAGTTAAAGTATTTTTAAAGTGCATATCATGGTTTCCTACAAC

33301AGTATGTAGGGTAATACCAGCATCATCAAGCATTTGAACTATTTCACGAGCGAACTCCAT

33361AGTTTTGTGTGTGATTGCTTTTCGCACATCAAAAATATCACCGTATTGAATCCAGGTAGT

33421AATTCCATTTTTCTTAGAATATTCTATTGCTTGTTTAATTCCATCCAACTGAATGGATTG

33481AACCCACTCATCATCGGCTTTAACACCTAAATGCCAATCACCTAAATTTAAAATTTTCAT

33541ATATCAAGAACCGTCATTGAAATGCAAAATAAAATTATTGAAATAAACCCATCTGGCGTG

33601CTAAAGAACCCAATCCAACATGCTCTAGTGAATAGATAAAATGCAAGAAAAAGTATCACA

33661TATCCAAGAAATATCATTATATCAAACTCCGTATAAAGCTAAAGGGCCGAAGCCCTTTAT

33721TTTGTAATAATGTCAAACTGTTCTTTAAAGCAGAAGCTTGAATCTTGATGCTGATACAAA

33781AATTCATAAGCTTTTTCGCGTTCACGGTCATAAAGAGTTCGGTCAGATGACAGTTCTTTA

33841ATACGTTCAAATGTTGATTCCATGTCATTTTCATCAAACCAAATGATACCGCTATCATGC

33901GAGGTCAAAGGAGTATTATCAACACGGAATTTTAAATTTTCGCCAGTAGATTTCCAAAAT

33961ACCGGAATTGTTCCACATGCACCAAGCTCGAGATGAGTATATTCTAAAGAACGTTGTAGA

34021TATTTTTTGTCCAACTTACTCAGCTGATAACCAAATCCGGATTTACTCATGCGTTCAAGC

34081ATTTCGCTATTTACATAACGGTCAAGAATTTGCGTTGGCAAATTAGGAGCAATTTTAATT

34141TGGTCTACTTGATGAAGACGATAATACTCGTATGGAATTCCTTTTTCTTTAATAGGAATG

34201AACGCTGGAGAACGTTCCAGACCTTCCATAATAGTACTTAGTCCTGCAGGTTTAAGATGT

34261TTTTCATGAAAATCAAACATCTGATAAAAACCTTTCCATGTAGTCGTACGACCAATCCAA

34321CGGTTGATATTCATGTTAATTTCAGAAACATCTTTCCAGTAGGTTGACCGAACCTTCGCA

34381ATATCCATAGGAGGCTGAAAGTTATATACGGTCGGTGCTTCTTCAATATCATCAAACAGA

34441GAAACTGTTTCTGGATACCATTCTTTCATCAGAACTTTATTAAAATCACCATTATCAGAA

34501TGGCTAAAAATAACATCAGCTCGACGAACAGTTTCTTCTAATCCCAAATTTCGGCGCAAA

34561GAAAGAGAAGAATGGTCATGTTGATAAACTACAACACGAACAGAAGGTTTAATGTTATCA

34621ATAATTTTTTTATAGTTATTAATAGTGTCTTCTTCAACCGAAGTAGCAGGAACTGAATTG

34681ATAATTAGAATATCACAATCATTTACCAGCTTAAGTGTTTTATCGTATTCTTTTGCTAAT

34741AAAACCGGAATTGAAAATGATTTATAATCATGCGCACAATTACGAGTAAATGATTTATCT

34801TTAGCATAAACCAAAGTTACTTCATGACCATTTTTAATAAACCAATCACGTTGCTCGAGT

34861GAGAATTTTGTTACACCACAACCTTCAAGACCTCGAGCCATAAAAATACATACTTTCATT

34921TAATATCCTCTTTGTTTTGGTTTATTTTACCAAAAATTTATAAAGCAATATAGGAGCCGA

34981AGCTCCTATCCACATAATACGCCATACAGAGGCTCGTTAGAACTTTTAAATTTTATGCGC

35041TTATATTTTATAGTTCCTTCTGCTTTAGCTTTATCATGAGACTCTTTAAAGCGTCTCATC

35101ATTTCCTCTTTAGAGGAACGAATTTTATTATAATCTATTTCAGAAGTCTGGGTGTTCATC

35161TTTCATAACTGCCACCATTTTTTGACTTGATAAGAATCAACCCACACTTTCATATTGGGG

35221TCTGCTCTTACAATAGGAGGATTAACTTCTTTAATTGAACTATCATGGTATTCTTTTTCA

35281GAAATTTTCACACAAAGATGACCATTCAAAGATTCAGTAAATCCTGCATTAATTTTAAGA

35341CGCTTGAATTTTACGTGTGTAAGCATCAATCATATCCTCAATCTGCGATCTAGTAGTCTT

35401CCAAAGAATACTGATGAGTTCATCGTTATATGGCTGTTTCAGAATATCCCGACTTTTCTT

35461GATAGCATATTCGTATTGAGCAAAATTATTGTTTTCAGCTGCGATCTGAGCGTGCTTATA

35521AAGACGATTCAGTTCGCGTTTGTTTTTAGACAATAACTTATTTGCTTTTCTTTCTGCTTC

35581AAGACGATTCTTTTCTTCAATAGAAGAAATAAGCTTTTCCACTTCATCATTAATTTCGGG

35641TTTATCAGTCATATTATTTCTCTAATATAAAATAAAAATCATCATCTGTTAAATGATACC

35701GATAGTTTAATTCTACACCATTAGATTTAAAAGCGGTATCATACGGATTTTCTGGATCAA

35761TATCAATGTCAAGAGCTAAAACTTCCCTGAAATACATTTTAAGTAAATAGGGAATAGCTT

35821CAACTTCAGGTATTTCTTCCAAGAATCCGGAGAGGTTAATCGTTAGCCTCATATAAAAAA

35881TCCAAACTAGGAGAATCGTCTACAACACTTTTCTTTTCAGCCCCCGGTGTTCTATAGGTT

35941GATTCTTCGTAATGCGTCATTTTATCGTAGATGTCTTGAATAAAAGTTTCATCTACTAAC

36001GCAACCATATCGTCGTCACGACTGTCATAGACATTGTGAACGAAGTAACTATATTTCTTT

36061GCAACTTCCTTACGTTCTTTTTTAATACGTTGGACAAATGCATTAAAACAAGCTTGAGTT

36121ATATACGCATGTGGGTTTTTATATTTCGTTTCATCAAAATTGTGAAGCCCCTTAATAGAA

36181GCTTCTATACCATCTGCAATCATTTCTTGTTTCCAAGACTGGGTGTATCCTGAAAAGTTG

36241AAACGTTTAGATAAGCCTTCTGCAATAAGCATAATGGCTAATCCGATAGTATCATTCTGA

36301CGAACTACTTTATTTGGGTCTTTATTATTTGCTAATTCTGTTTTCCAATCAATAATAGCT

36361TGTAAAAGCTCTTTATTGTTTACGTAATTATATTTAGGCTTAGTTTCTGACATTTTCACC

36421TCTTAGCTCAATTCATAGATCTATTATATCATAATATTTGAAGATCTATCTTAAAGCATA

36481GAGGATATCAGTTATCTAAGTAAGCAGTATGCTTGGAACACTTTCTCCATCATCTTTTTA

36541AATTCATGAATATCTATGTAATGATCATTAATATCAGCGCGATATAAAAAGTCTAAAAAC

36601TTTTTATGGGTATTAGAATATTTACTTCCAGTTAATTCAGAAACATGGCTTTCCCAATAT

36661CCAACACCAGTTTTATTTGGGAAAAATGAAATTTTCATGTATTGGTCAACATAGAAAATA

36721CCAAAATTTTCAATAGCAATAGAAATATTAATTAATTTCATACATTCATCAACTTAGCCG

36781CTTCAAGAGCTGCATCTAGTGAATCAAATTGGTCAACATATTCAATCAATTCGCCGTAAT

36841TGGCATATAACCACCATTGGCTAAATTCATACTCAAGGATGAATCCGTTTCCTTCAATTT

36901GAGTTAAACCAATGCCATTTGTATTTACTTCATATCCAGCAAGACGCAAATCGTTAATAA

36961GAGCTTCGTTCATAATTATACCTTAGTAATTTTCAGGTCTGCAAATTTTTTCTTACGTTG

37021ATTTTTCATGCGACGAATAGTTTTATCGGAAATTTCATGCTTTTGATAAGCTTTAGATTC

37081TACACCAAAAGCTTTAACATCAAATTCTGACAAGATATATTGAACCAACAATTCACGGAC

37141AGTATTGCGTCCAATCTTCTGATCATTCTGTTTCATCGTCTGATGAAGCTCTTTTTCCCA

37201TTTATCCAAAATTTGAGGAGTTACAATATCGCCTTTTTCTAGCAAAGAAACTACTTTATC

37261ATATGCGTAAGAATTGATGGCGTTTTTAATTTGAATAGTCATACATTATCCTCAATTACG

37321TTAAAATTTTATTATCCAAAAAGGGCCGAAGCCCTTAGCTAAACTTTTTGGCACCCTTCC

37381AGCCTTCGTACATCATTGCGACTGACAATGACAAAGCTCCTTCACATGCTGCATACTTAT

37441TATTCCAGAACCAATTTAGAAAAACTTCATCCTCAATACCATTGTGTTTCATGTTTTGGA

37501AAAATTTGGCGCGTTCTTCAGCAATTCGCTGCGATAATTTAGATTCAGGATTCATTTAAA

37561TTTCCCAATTACCATTTTCATCAATAAATTTAATCCAGTCATTTACTGACCACTTAGTTG

37621TATCACCTTTTGGAGTAACATTTAAAATATATAGCCCTTGCTTAAAAAGCATTCGTTTGA

37681TATTCATATTTTCCTCAGCTGTAACGATAGCACTCGTTTGATTTACGTTTAGCAACTCGT

37741TGAGAAATATTATAATCAAAATCATCGTCAATGTAAACTGATTTTTTCAACTTTCTTACT

37801TCACCACGTAATTGACGATTCAATTCATTCTTAACCTCTGAATCAACACCTCGCATACGT

37861CGCCATTTATCTGAGCGAAAAATGTTTTCAACCATATCTTTATGACTTACACCATCAGGA

37921GCTTTACGCTTTCCGAAATAGTCATAATCACGCACTTTTAAGTCTTTACGACGATACGTT

37981TTACCCATGGAGTTTAATTTCCTTAGCAACTGAACTAAATGCAGCACGATCGCAAATCAT

38041GCGTTTATGTAACTTGAGTATAATATAGCAAACTTCAAAACTATTTACATGAGTAACACG

38101AACAGCATTACCATTAATGAGATAATACTGTCCCTTTTTAATTTCTTTATCAACCATAAC

38161CATATCAATTCCTCAAAGGCAATTCATATGTTAATAATACCACAGTTTGAACTTGTTGTA

38221AACAACTTTGTGAAAATATTTTAGGGAATGATAAGAAGGGAACGATAGCTTAGAATGGTA

38281ATATACAGAATGTGAGAAAGAAGGACCCAGAGGGCCCGTCTTAGTCTTCTATGATATCTC

38341TATCATATCCAAGTGAAATGAGAGTTTCTTTGAAGTGTTTAATGTTCTTTTGTCTAGAAT

38401CATTAATGAAAATGACTGGATAACGAATGTTAAGAGATGTGAATCCAGCGCGTTTAGCAA

38461GAGATACAATCAGCGGACGATCATACTCAATCTTACCGTTATTTGTAAGAACTTTATAGA

38521AAGTAAAAGGAGCATTGAGCTCCTTTAGAAGTTTTGTAACTGATTGACATCCAGGACAAC

38581GACCTACTTCTTCGGGGATGCCATAGATTTCAATCTTATTCTTTAAGTTCGAGTTTTGTT

38641CCACGAGAAATAATTCCTTGATAAGCCCAGTATGGCGGGTTAACAGAATCCTCACCAGAG

38701TTTTCTTCAGGAAAATAAACCTGGATAACGAAGCCGGATTCATCAAAGGTGAAATAAGTA

38761GTAGGCAGTTCACCTAACAAATCGGCACGAGCGTTGATTTCTTCGAGGTCTTGTTCACGT

38821CCAGCCCAGAAGCTTGGGTTCAGACTACATTCATAGAAATAATAGTCATTACCGAACATA

38881TCAAAACCATTCCAACTCTCTACTACATACCGCTCAAAACTAATCATAATTAAGCCTTTT

38941TATCAAGAACAGCATTCAGCTTGTTAGTAATTTTATCCAGACGCTCATTAAACTCACTAG

39001AAGATAATCCCTTTTCTGGAGAAATTAAACTAATCACGAAAAATATAGCAATAAAAGGAA

39061TAAGGAAAATAGCTCCAACTGCCATAAACAGAAAGAATGTTACAGTTGTAAGAAAATCAG

39121CTAAACCTTTACGAAATTTATACATATTTTACCCTTAATTAATTAACCAAGCATTGATAA

39181GCACTAAACTATATTGGGAATAAAATTCTGGACCAAAATGAAAAATCATATCATTTATAG

39241TATCCATAATGTAATTCAGTTTAATCATGTTTCCACACCCCATCGGTATTTGACCAAAGT

39301CGCTGATTATCTGATCCTCGCCACAGCTTTTTAGTCGGAAGATTTTTCTCATACTTCCCA

39361TCAATAATAACATCAACATATTTAAGCATTTCTAGCTGTTTAATATCTTCAAACTTATAT

39421CCTGTCCACAACCAAATGCTTTTATTGGGATAAAGATTTTTAATAGTTTGAACCACAGAG

39481TGAATCACGTCTCTGTTATCAGGATAAAGAGGGTCTCCTCCAGTTATAGTCAATCCTTCT

39541ATATAATCATTATTCAAACATTCAATCAATTGTTCTAGTGTTTCACCAGTGAATGGAATA

39601CCATTTCTAGCATTCCATGTTGATTTATTATAACACCCTTCACATTTATGCAAACAACCT

39661GTAACGAAAAGAACGACCCTGCATCCAGGGCCATTCACAAAATCGCAAGGATAAAATCTA

39721TCATAATTCATTGGTGTTTAACCCTATGCATGATTTCTTTATTTTTGCCGAGATTAAATC

39781CACGTTCGTTCGGATTTCCCAAATAACCACAAGTTCTTCTTATTGTGTTCATCTTTTTAG

39841GATCAGTTTCTCCACAAATAGAACAAACAAACCCGTTTTCAGTAGGAGTCATTTCATGGG

39901TACTTCCACACGTAAAACATTTATCTACTGGCATGTTAACACCAAAATAATCTAAATGTT

39961GTGCAGCATAATCCCAGACAGCCTCAAGACCTTTTAGGTTATTTTTCATATCAGGAAGTT

40021CAACATAAGAAATGTGACCACCTGTCGCAATGAAATGATATGGCGCTTCACGAGAAATTT

40081TTTCAAACGGAGTAATATTTTCTTCTACTGAAACGTGGAAACTATTAGTGTACCAGCCTT

40141TATCGGTAACATCTTTTACACTTCCATATTTTTCTGTATCAAGTTTACAGAAGCGATAAC

40201AAAGGTTTTCAGCAGGAGTCGAATATAAACTAAAAGCAAATCCAGTTCTTTCAGTCCACT

40261GTTTAAGATGAGCATTCATTTTAGTTAAAATTTCTTGTCCAATATCACGACCGACAAGAA

40321TATTCAATTCGTGAATACCAATGTATCCTAGGGACACTGAACTTCTACCATTTTTAAATA

40381ACTCAATTATATCGTCATCAGGTTTAAGACGAACCCCGAATGCACCTTCTTGGTAAAGAA

40441TAGGAGCAACAGTCGCTTTAACTCCTTTTAAGGAACTAATTCTACACATCAAGGCTTCAA

40501AGCACAGATCCATTCGTTCATTAAATAGCTCAACAAATTTCTGTTCATTGAACTGTGTTC

40561CAATATAAGAATCTAATGCGATACGAGGAAGATTCAGTGTTACAACACCGAGATTATTAC

40621GTCCATCAAGAATTTCATTACCAGTCGAATCTTTCCATACGCTCAAGAAACTACGGCAGC

40681CCATCGGAGAAACAGGAATAGATGAACCAGTGATAGCTTTATTGTTCTTAGCTGAAATAA

40741TATCAGGATACATCCTTTTGCTTGCACACTCTAGAGCAAGCTGCTTAATATCATAGTTCG

40801GATCGTCTTTATAAAGATTAACACCTTCTTCAACGAACATAACAAGCTTAGGGAAAATAG

40861GAGTTATCCCATCACGACCGAGACCTTTAATACGATTTTTCAGAATTGCTTTTTGAATCA

40921TTCGTTCAGTCCAGTCAGTTCCCGTACCAAATGTAATTGTTACAAAAGGTGTTTGCCCGT

40981TTGAACTAAAGAGAGTATTTACTTCATATTCATAAGCTTGAAATGCATCGTATACGTCTT

41041TTTCTGTTTTAGATTGAGCATAATTCAACGCATCGGCGATTTGCCATTTTTCTGCATCCT

41101CAATATGTTTTGCATAGGTGCGTTTAACATAAGGAGAAAGTACTTTATCTACATTCGCAA

41161AAGTCGTTCCGCCGTATTGGTGAGAAGCAACCTGTGCAGTAATTTGTGCCATAATTGCAG

41221TAGCAACGCCAATTGATTTAGGAGTTTCAATCTGTGCATTACCAAGCTTAAATCCGTTCT

41281CAAGCATTCCTTTTAAATCGACTAAACAGCAATTGGTAAAAGGCAATGATATACTGTAAT

41341CTAAATCATGAAAATGAATTAACCCGGCATTATGAGCATTTACAATATGGCTTGGAAGAA

41401TACTGCAAGCGATATGTTTAGAAACAATACCGGCCATTAAATCTCTTTTTGTTGGAAAGA

41461CACGAGAATCTTTGTTGGCGTTTTCGGTATTTAATTCTTTATCTTTATTTGAAATAAAAT

41521TTAAAACATCTTTCTCTAAAGACATAATTCTAATTTATCCTTTTGAAATTCTTCGTCTAA

41581ATATGGGTTCCAACCCATTTTAAACCTTTTGATGAAATTATCAATTCTAAATTTCGGTAA

41641GTTTAAATCCGCAAAAATAGATGATTTACTTTTAAAATCTGGACCTAAATAATCATATAA

41701AATTTCTGCGTATTTTAACCATAATATATAAGTACCAGATTTAATTGCTCTCGGAGTTTT

41761CCAAGGTTTTACACCATACATCGGATTTCCACTTCCTTTATGCCTGACAGAAGATGAAAT

41821TCCAATTTTAATTTTAGCTTCCTCTGTATGCGAAAATCCATGATGTGGATTTTCTTTCCA

41881ATATTTTTTAGAAGCTTCTGATATTTTCTTTCTGGCTAAATTAGTATGATGCTTTCCATA

41941AAATCCATGATTTTTACCTTTATTACGATTTCTTTTAATCATGGTTATTGATTGCTTTTT

42001TCTAGTTTCTTTAGAAGGAGAAACACCGTACGTTGTAAATTGACCATTAGCATATGCTAA

42061ATTAAAATACTCTTTATTCCATATATCTTTTTCTATATTCTTTTGAAATTCAGCTTCAAT

42121GCTAGTTATTTGCTCATCTCCGACTAATTGAAAATATAATATTTCAACTTTCGGCGTTTC

42181TTTCAACAGGGCTTTTTGAAATTCGGAAGATGACGAAGACGAATAATAAAATTTACCATT

42241TTTATCAATAATATTCGTTCCGTCAAAAGTGCAATTAGTTTTACTTCCAATGTAATAATA

42301TGGCGGGGTTTTAGCATTAATTCTATTAATAAATGATAGTTTATATACTACATTCATTTT

42361AAACTCTTTCTAAGCTGCTTCTTAAATGAAGCTATTAATTGTGTTTTGGTGTCAGATTCA

42421TTATATTCAAATCCTCTTTGAAGCATCTCGGCCATCATTTCCTCTTTTCCTAAACGAGAA

42481AATTCCTTTGATTTATCTCCAACAAAGTTAGGGTGAATATTATTTTGGGTGTAATCGGAT

42541TTTAAATAAGTAAGTAAATTTTCTAACCATTCAAGATAATCAACACCTTGTCCCTTTAAG

42601CCAGAACGATTGAATTTATGCTTCATTTGACCTTCTGCAGCATTACAGAGATTACATAGC

42661AACCCACGTACCTTTCCTGCTTTTGGTCCATTTAATTCATGGTCATGGTCAAGGTGATTA

42721GCTTGAACATCAGGATTTAGTTCTCGTTGGCAAATTAAGCATTTACCGTTTTGTGCATTA

42781TAAAATTTTTGTTTTTCTTCTTTGTATAATTTGCCAGTCAATAACATAATAAAACCCTTA

42841CCTTGAATAGATAAGGGTATTTATTATTTTCAAGTATTGTAAAACATTCGATGCAATCGC

42901TTATATTGCCGAATCTTTTGGTCAGAAAAAGAAATTTGGGTTTCAAGCCATTCAATATAT

42961TCTGCCGCAGCTTGCATCAAATTTCCTTCATAGCCATCGTTATTTTCTTGTGCAGCTAAT

43021TTAGCTAATGCGTATGAAATACGTTCACCTTGAAAATCAGCTTTAGGCTTCTGAACAACT

43081TGATTAGTTCTCTCTACAACTTCTTCAATTTCGCCATTTTCTACTGATTCAGTATTCCAC

43141AAGCACCAATACGTAATTGGCTTATCGTAGATGTTAATAATCTTTCCATCAGAAAGTTCA

43201ATTTCAATAATTCCAGTATCAGGCTCTATATCATCTTCACATTCTTTTGCAAGTTCACGA

43261ACTTTAAAGACAGTACCTGCACTAAGTTCCGGCCAGTAATTACACAGCCCTTTATCAGCA

43321CGATTAATTCTAAACCACTTATCTACTGTAATCATGTCCCATCTCCATATCAATTATGTC

43381ATTTATTGTAGGTTCATTATATACTGTTTCTTCATCAGTGTAAACCGGTTCTTCCGGCTC

43441TGGCTCTACTGTTTCCCATCTAGCCGCCCACCAGGGTTTAACCCCGAAGCTTATTAAGTT

43501CTTCATCATCAATCCAGGATTCTAGTCCTGATGGCAGGTCACACTCAATTTCCCAAAGAA

43561GTTCTTCAATTTTCTGAAGACGATCTAGTTCTTCGGCTGGAATAGTAACCATTGACGGAG

43621CTCGTGATACATTAATATCGTAAATCATATTTACCCCAATTTAGCCATACAATCGCCGTA

43681TTTCCACTTAGAAATAGACTTTTCACCATTAGAATAATAAACTTCGAGTTTAGCACGATT

43741ATTTTTAATTTGAATAACCTTGGCTGTCATCAAAGTTCCATACCCATAATAAACTGCAAC

43801TTCATCACCTACATATACCGCGCTTCCGCGGTAATCATGAATATAGTCAGTTCCTTCGAG

43861CATCATTTAAAATATTCTCGCAGTTGGTCAAATCCACCAATATGACTTCCATCAGGAGCA

43921AATACCTGAGGCATTGTTAAGCCGATTTGAGTATCACGACCTAGTTTAGTCAGAAGCTCA

43981GCGATTTTCTCATCATCAAAAACACCTTTTTCCGGCATAATGTTGATAAATTCAAACGGC

44041TGTTTCTTCACGGTCAAAAGACGTTTTGCATTATCGCAATACACGCATTTGTGGATGTTG

44101CTATCATAACCATATACTTTAAACATATTATTCCTTAATTCCTAATACTTGTTTAAAAGT

44161CTCGTCGTAATCAAGACTTTGGCCTGTTTGTTCTTTATGTTTGTATATAATATCACTTAC

44221TTCTGATAGCATATTTTTATATGAACGAGTTAAAGCAGATTTAAGCACGCTGTATCTATC

44281AGGAAATTTACCATGTTCATTATAATACGCTATTGCTAATTCACGGACTGCCTTTTCAGC

44341AATCTCCATATATTCTTTACGCTTGGTCATTTTCTTCTCGGTCAAATCGGTGTTTACAAT

44401GGCGACATTTATAACGAAGATTACTAGTCTGCCAATGGACCAATTGCACCTGTTCAGTTC

44461CACATTCAGGGCAATTAGGAACGTTTTTAGAAGCACGTTCGCGGCGTTCAACCATGACCA

44521TTACAGAATCCCAATTAATAGGGCTGCTATAATCATCACAACCATGAATCTTTCCACGCA

44581TTTCCAAATCGTCTTCTTCACCGGCCATAATAATTTTAATTAAACTGGAATTACTTGAAG

44641CTGCAATGTCTTCTAATAGACGCTTTTTCATTTCAATACCTCAATAGCATTACGTAAACC

44701ATTTGCTTTTGCATTAAGAGCTTTTAACAATTTGGTGTGTTCTGCAATTTGGGCTTCAAC

44761TTCAATCAAACGAGCATTGAGATATTCGCGTTCTTCACTCAGATTATCAATCTTTTCTAC

44821TTTAGGCTCTTTTACAACTGTCTTATATGTATTAGGGTTCTTCAGTTTAATTAGAATGCG

44881TGAACTGTATAACATTGAACCATTATCTTCGGTTTCGTCATACATTTCTACTACGTCCAT

44941GATTTTTAACATCATGCGGAGTTTATAAGACAACGAACGAATAGTTTTGTTGTGCTTATT

45001AATTTCTCTAGAGTTCATGGTGCCGAGATTACGTGAGTAATAATAAGGAACCTGGATATT

45061AATTAGCACTGATGAACCACCTGGCTTATGTGTTACTTCATCAGCAATAACCCAATCCTT

45121AACATGAACTGTATTTGTACGGTCATCGCCCGCGGTAAATAGTTTACGAGCATGCTTAAA

45181AAGCATATTCACTTGGTCATTGAATTGACAAGCGAATTTATCAGCAAATTTAGTTTCGGG

45241AATGGTAGATAACCAATCAGCCAAATGGGTCTTTTTAACATCGTTAACAATAGTAAATTC

45301AACCAAATCGGTTATAGATGTCGCAGTTTTAATGAATCTCGGCTTCATAAAGTTTTTAAT

45361GTTATCGCGAGCTACAGTAGAAGATAACATACGAGAACGTTTAAACCATTCAAGTAAATT

45421GCCTAACGAATGAGAATAAGCGTTCTGAGTATCTTTACAGATATGATTTTCCTGGCCAAC

45481ATACTGAAGGAAATCACGAAGAATATTATTGATTACTGCTCTGTGTCCGATCTGATAATT

45541ACGGTGTTTAGTAATAAGGCTGTCAAAATAATCAATATAATGTTTACGAGTTTTCATGTT

45601CTTCTCACTTGGTTAATGATTTATACTCCGAGCCATCCTTGGCTTTAAATTACTTAATTA

45661ACTGTAAAGCTTGTTCAAGACGATCCAGGCGATTAACAGATTCTTCCCAAATCCTTTTAG

45721CCTGCTCATATTCTTTCTGCGCTTTATTAGAAATTTCTAGAACTTCTTTATAAGCTTTTT

45781CTAGTGCAATAACTTCTGGACGAATTTCTACTGGTTCAGGCGAATCGTCAAGACATTCCA

45841TTAGTTCCTCAAGGGTAGTTTCTTCTTTAGGAGTATTCACAATTTCATCACATTTTTGTT

45901GGTAAATTTCTTTATCAGTCGGTGAGTACGCACACTTTACTTCACGGAGACTAACTACAA

45961TTTTATATCCTTCCCAACCGCATATATTCTTAAAAGGATAAGTATGAATATTTTCTACTG

46021TTTCCATACAAAGTAATGCGGTCTCTAACTGACGAGTTATATCGCTAGCAATCGAGAAAA

46081ATTTATTAATGTTCTTTTGATTAGCTTCTGGTGTAAAAAATTCATAATTCACAAAAATAG

46141CTGCTTTATTTTTATCAAGCTCATATTCTTTTATGATAATCATATCAGAAGCCCAAGGAT

46201GGATTTGACGATAATCACCATAGCATAATTTAGAAGCTGATTTTAGAATTTGCTTCTTGA

46261AAAGTCTAAAATTACTAATCCAACGACGAGTAAAAATATTCTCAGGGTCTTCTTTATTAT

46321TAAGATGATAAGAATTAACATCACCGAACCAACTGTATCCTACTACATCTTTAGTTCGTT

46381TAATTTCTTTCCCAAATTTACCAAGAATATCTTGATTAACTAAATATGAAAGAAGACGAG

46441AACCTTTAAAAGTTTCCTTAATATCATCTTTATATTCGCGACTAGAAATTTTATGAACTT

46501CATGAATAAATTTGCTGGAAGAAACAATACTTGCATCAGTATTATTGAGCCATTGATTGA

46561TAATGGAAATAACACTATTCTGACTAAACACAGGTTTAACTTTATCATCAATAACGCTAT

46621TGAATGATTTGATATATTCGTTACGAGTCATATTAATCTCCTCAGTAGAAAGTAAGAACA

46681TTATACCACATCCTTGTGGCAAAGTAAACTAGTTTAGTGCATTTAGTGCATTGTTCAGTT

46741TAGAACGTTGCTTCGTAAGATTTTTGACTTTTTCTTGTGCTTTTTCTAGCATCTTTTCAG

46801CTTCTAGCACTTCATTGGTCGCTTTAACGAGTTCATCATCTACTGTCTTAAGAGACTTCT

46861CAATCGCATCCGCGTGCCATTTTTCAACAGGTTTAAGACTCGGATTTTCAATAGGAAGAA

46921AATTCACTTTATTAAATTTCCATGCATCTTTATTACCTGAGCTATACATCCAAAATTTAG

46981GATCGTTTGATGAGCAATTAGATAAATTTAAGTTATCTTGCACTTCCTGCTTTTTCTCTT

47041GCGGGACTTCGTCCTTTCTCAAAAAATCGTATTTAAATGAAACGATCATATCAAGTTCAT

47101ACGTTGTTGTATGTTCCCAATTTCGCTCAAAATGAGGTAAAATCTTAGATTGAACAGCGG

47161CAACAACATCCATATACTTAAATGCTTCTGTCAACTGTGATTTAAGGCATTCACAAATTG

47221AAAGAGAATTTTTTGTATTAGGTTTAAAGCTAATTCGTGCAGTTCTGTTATTTTCTTTTA

47281ATGGTCTTACTTCCATCTGAAGAGTATAACCATCAAATTTCAGGTTCTTTAAGTTAATAT

47341CAGAACCTTTTAATCGACTAGCCGTCGACAAAATTTGCTTTAATTGTTTACGGAATAGAG

47401CAATAAATCTCCATTCAATACGATAATGATTTGGATTAAAAAATCCAGATGATAAATCGA

47461CCTTACTTTGACCAACGCCTTGAACAAATAGAGGATTTTTATAATCAATAGTTTTACAGA

47521AATCGGTGAGCTGCTCACGAGCAGTCATTTGTGTAATATATCCTGCTTCGCTAAAATTAC

47581GCACCCATTCGCTGCTGTTCAAATGTTTAAAGGCATGAATTGAACAATTAACTTTACCAA

47641GGTCTAAATTATTTTCACACAAAAATGTCATAGCATCACGAGTATAGCTGGCATTACGAA

47701CCATATCTTCAATTTGAGAACGAGTTTTCATAGTGTTCCTTAAGATTTAAGTAAATCAAC

47761AATTTTAATTAACTTTTCACGCTCAGATTTAGCTTTACTACTCAGTCCAGATAGTCTGAA

47821AATTTCATCATCATATTGCTGAATAGAAATATTCAGCTCTTCAATCTGCTTATTAAAATA

47881ATCAATTTGTTCAGAATGTTTTTCGTTACTACGAACTGGTACAGGTTTTGTAGGTAATTT

47941AGATGAACTGGATTCATCCTGGCGATAAATTAAAATACAACTTGAACCAATTGGACATTT

48001TGCACCAGATGAATATTCTAATGTTCCAGCTTCTTTTAAAACTTCAAAAGCTAAACAGAG

48061ATGATGCCCCATATTGACATAATCTGTAGAGCGAGCTCTGACGTAATAATCATCTCCGCG

48121AATACTAAATTTAAAGCATTTAAGGTCTTCTGTATTATTGGTCTTAAAAATCATTTGTTT

48181ATCTAGACCCTTAGCTAATCGAGCGCCTAATGCTAATAATCGTCGTTGATTTTCCCACAA

48241ATCCTCAATCATTTGATCGAATGATAATTTTGGCATTAGTCGACCATAAAGGTCATATCC

48301TTTATTATAATTACGAAGGATTTCACTCGCGTTAATACGAGACAACGCTCCAATTTTATT

48361AAGGGCTTTCATGATGCGATTAGATAAACCCATTCCAGACCCGTTTGTTCGTTGTAAATG

48421TTTTTCTAATCCAAACCCAATATCAACTTTAAATTTATCTAAAATTTCAGCATGAACGTC

48481TCTGTCAATAACATTCAAATCCAAAGTTGGGTTAAATCTATGAAAAAATTTATCTGGCTC

48541TCCACGACGAAGTACAGTCCATTCATTCAATTTTTTATTAACTAAAGATTTAATTACTGC

48601ATTGACATTATTAATTACTACTGACATATTTTCCTCACTCAATTTCAATTTTACTAAATA

48661TACAGAATAAGATAGAACAGACTATATAAGCACCACACACAGATTGAACTAATACCATTC

48721CAAAGAACCAAACAATATTATCAAACAATGTCTGTTTTACGTCGAAAGGACGTAAACTTA

48781CAGTAATATTATCGCCTTTTTCTATTGATGAATACATCTCTGGTGAAATATATTCGCTAA

48841ATCTATAACCGTCTTTGAGTTCATATACGGCAATAAACGATAAACTAGACCCCTTTCCTT

48901GAGTTCCTGTAAGGGTATTAACTACAGTAACATCATAATCTTTATAATGCATATAATCAT

48961TAATAGCATAATAACCATATGCAACTACTACACATAAACAGCATATCAATAAATTCAATC

49021TTTTAATTATCAACTGTTTCATAATAATCTCAATTAAAAGGGCTTAGAACCATTATACCA

49081TCCTTGGTATAAAGCGGTTATGCGAGTACCGTCTTTAACCGTTCTTCAAACTTCCGAAGA

49141GTATTCTGGCGTTCAGCTCTTTGCTTTTTGTAAGTTTCAATACGCTCTGAAATGAGAGTG

49201TATCGTTCATTTACTGATTCTTTCATAAAATCAGGAATTTCACGAGAAGCTTTAATCTCG

49261TCAAATTTATCAATAACAGCTTGCTCTTCAGCAATTAAGTTATCATACATCAAAATATCT

49321TTCTTGATGAACTCAATATCTTCTTGAGTTACACGAGATAATTTAGATGCTTTATCCTTT

49381TTGTACTGTTCGTTAGTATCACGAGACCAGTGTAATGTACGATTTTTATTCGTATTCTTA

49441TAAATTTCTACAATACCAATCTCATCAATAATAACGATCCAATTCCAACGGGATTTGTAA

49501ATTTGTCCTCCATCAACCGTGATTTCACCTCCGATTGAGATGTCATTAAAGAACTTGCTT

49561TGTGCTTCAGATTTAAATTTACCATCGTTGTAATTTACCAGGTTGAAAATATCTTTAGCG

49621TTCATTTTGTGTTCCTCCGTAGTTGATAGTTGTATAGTACCACAGAGGAACGGTCTTGTA

49681AACAACTAAAAGAAACTTCTTTCACAATTTTTTCCACTGAACCACGCACTCACTGCTTTC

49741TTAGTCTCAGGAGCAGTGTTATCCATAAACCATTCAAAGGCAGCCTTTTTATGATTCTGG

49801AGGGCTTCTCGGGCTTTAATCTGCTCACGGTCTATTAACACTAACATATGAGCCTTTCTT

49861GTCACCAAGGGCTTCTTATGATTCTTGGAGTATTCCCAGTCATTTGTCCAACGCATTGTC

49921GTTGCGAACTGAAATACTGCTTCTTTAATTTTAGTTTCGTAAATTTCACGAGCCTTTGAG

49981TATAACATCACTACCTCCATTTACCAGTTTAATTCTAATCATCTTTTTAATGGCAGTCCA

50041TATAATCTATTTCTGAACTGCCTTTTTGTCTTAGAAGTCCTCTTATGAATTTATTTCAGA

50101AGAGTAACCGTAGCAATTTCTTCCCAACCGTTTTTGTCGGTCATAATAAAGTCAGCAAGA

50161TAAAGAGCAGTACGCAGTGAAACATTGCGTAAACGATTAACATTGACTTTCATCCATGAT

50221AATGCTTTATAAGTTTCTTCATCAGAAAGACCGCGTTTTTGCATCATGTCAGTTGAAAGA

50281ATAACATCTTCAACCCTGACCATAATTTCTTCATTAGTGTGAACACCCAAATCCAAATAA

50341ACTGAGCGGGACACTAATGCTTGTAAATGTGGAGCAAGTTTAGTACCACGGTCTAATTCG

50401CGGTCAATATCAACGTTTGTGATAAAAACAATCGTTCCTTTAAATTCAAACTCACGCTCA

50461ATGCCTTTTTCTTCTAAGTAAGAAGATGCAGTGCTCCAGCAGACTTTACGGGTCTCTCCA

50521GTGTCCAGAGCAGCTTTCAGAAGATTAAGAATGTCCATATCAGAGAAAACATCCACATCA

50581TCAATCAAAAGGACAGAATTCTCTTCACGATTATTCCAAAGTTGTTCATAAAGACCGATA

50641CCGGAGATTTTACCGTTAATGCTTTTATATTCAATGTATCCATTATCATTTGCTTTATTC

50701AAAGCTTTATCTAAAGAATACGTTTTACCAATACCCGCCGCACCAGAGATAATTAATGAA

50761CGAATGTTTCCGTTAATAATACCATTCGTCATCATTCCCATAACATTAAATCTTTTATTA

50821ATGCGGGTTTTCATATCTTCATATGATTCTTTAACTTCTTCAACTTTTACACCATCATAT

50881GAAATGTCTGATTTGTAAACCCAAACACCGCGACGCTTACCGTCAATTTCAACAAAAACT

50941TTACCATCTCCTTGTGCATCTACCGGAGCATTATCTGGGAACCATTCACCTAAGAGCTCA

51001AAAGTTCCAGAGATTTCTTTACCGAAGTAGATACCCTTATTGATAGTTACAGTTTTCATT

51061TTATTCTCCAATCTCACATTTGTTTTGATAGTTGTATAGTACCATAAAGCTTTATGCTTG

51121TAAACAATTTTGTGAAAAATTTTTGAAATAAAAAGGGAGCCCGAAGGCTCCCTATCATTT

51181ATAATAACTTCGGTGGTTTTCAAGATAAACCCTCTCAAGGAAGTCATCCCAAAAACCCAT

51241GTCTACTTTTTGCTGCATACCGTTCTTAGAAGCTTCAGTAGATGCTGCTTCTACTTGATC

51301GACCACATCTTCCAAAAACTCTTGAACCGTTTTAAATGGATGTTTACCCAACTTCACGTC

51361GAGAATAAATGGAGCATCTTGGAGTGGATAAACCAAGTCACCAGTTTTGTAAATTTCCAA

51421TAGTTGGAGTCCACCACGGCAAGCATGACTCAGAGCTTTCCAGTCAATACCTTCATTGGC

51481TTCGGCCTTACGAGCACGTTCACCGTATTCAGCATCTAATTTGTTCAGTGACTGCTTAAG

51541CTCAATAAGAGAAAGCGTTGTCTGATATTTACGACCCAACACTGTGTAGAACGTCTGTGG

51601GCCTGTTTTCTCATGATTATGGAACACCCATTCACAGAATTCGTTTTCTGGAAGACGATG

51661CTTAATATCTTCAACTTTAGTACGACGCTGCTTAATAGAACCATCTTCTTGGTAATCAAC

51721CCATTGCTCAGGGATTTGATTAACTACTTTCAATACATCACGTAATGCAGCCAAACGAGA

51781ACCCTTAACGCCGTATTTAGAAGCTTGCTTACGGACATATCCTAAATAGGATTTCATGTT

51841AGTCGTATAAAAACGAGAACGGTTGTCTTGAATAAACTTCCACACATCAGGCAAATCAGA

51901TTTAACCACTAACTCAGGTGGAGTGTGAAGCATATCTAACGCTACAGTTTCACCATCTGC

51961TGCTAATTTAAAGAAATATTTAAGACTGTATAGTTCATGGTCAATATCATCTTTAGTGTT

52021TTTAGATGATGTGTTGTTAGTGTTTTTACTCATGTGCTCTTTAACATTTCCAATAAGAAT

52081ATCACGAGCAGGAGGAACAAAGATTTCTTTAAAATCTACATCAGATTCTGGGGTAGAAGT

52141TCCATAAAGATGACTACCAAAATAGCTTTTCATTACTGTTTTCATCATTCAGCCTTATAT

52201TCAATAACAGGACATACTTTAGCTTTACGCGCTTTTAAAAATTCGACGATAATAGATTTC

52261TTGGGTTGAATAGGAGGTAAACCTTTATACGCCCTATCAATATTTTCTACATGTAAAGCA

52321TAATCCTTTTTCCATTTATAATCTTTATATTTTTCACGCAAATGTGCAGGAATAACAAAT

52381GAACCGATAATAAGTAAAACAACCAAAAGTGCAAGTATAAATGCCGATGGAAGAAGAGTC

52441CATAAACTACCAAAAATAAGCCATTTCTGTATGATTTCTGCACAGATAATAGTTAGACCT

52501GCGAGTATAGTACATCCGAAAAAAGAAACGAAAAATGTAGGGATAACAATAGACCAAAAG

52561TATGCACACAGTGTCTTAGGTCGTTTCCATTCGTCGTTAAACAGTTTGAATAATTTATAG

52621TGCCAAGAGTTTTCATTAATAATCATAATTATCCTTTCATTGAAGGTGTAACAGTTGTTA

52681AATACTTAATCATAGCTTCAGCTTCTGATTTTGATAATGAAATTTCTTCATTGTTTTGAC

52741GAATAGAAATAAAATCCGGATGACGGTCACCACCAGCTTTTAAAACACACATATAAGTAT

52801CAGTTTCATCTTCAATATCTGAAATGATTGAAGCAGTTCCACATGAATGTGGTTTTTGAT

52861AAATCAATACAGCTTTTCTTCCACCGTTTGACTGTTCATAAACTTTTAACACATCAAACA

52921AATCGCTTCTTTTATCAATAGCTAAGATGTTGTTTTCATCTTCAATATTAAATCCTTCGG

52981ATACTTCATAAAGTGTCACCCATAAATCACCAATATCAATAACAGCGTTCACTTTTCCAT

53041GACTTTCAAGAGCATTACAAATTTTCTTAATTTCATCATAACTCATTTTAATAATATCAT

53101CTCCTTGAATAAGGAAGATGTCATTTTTAAATTCAACACGAATTAAACCACTTCCACTTG

53161AAATAAACATATTTCCTCACTTTGAAATCATAGTTGGAATGACAGAATCGAGATAAGTCT

53221TTAGCGCAATAGCTTCGTCTTTGGTAAATGTGACAATATGTGATCGAAAATCGTCGATTT

53281GGCGAATAGTTAAGACATCTCCCTCTTCATAAGAAGTCTTGACATCCAAAGTCGTAGTAT

53341AATTTGCAGCTACTGGATTAATAATGCTAGCACCGCGATCTTTAGCCCATTGAAGTTTAG

53401CCTTTTCGGGTGTTTCGACTGTTTCAGATTCCAAGAATTCTTTCACCTTGGCAACGAATG

53461TTGCCCAATTATTGATAGAAAGACACATAAATTCAGTTTCAATATTACCGCCGTAAAGGC

53521CACGATAAAAACGAACTTGGAGGTCGTCTGTAATATTAGTTACACCGGTAACTCCTGAGG

53581GAGCATAAGATAAAATCGCAGCCAGTTCCATAGGAAGAATAGTAATACTATCGTCTTCCT

53641GCGTCAGAACGTATACATTATCACGTTTTTCGATAACTAGGTCACCGAACAAATCAGTTG

53701TTTCAATTACAGCTTTCATATTATTTACCAATTAAAGTTTCAATATAAGAATCGAGATTT

53761ACTTTAGCTTCTTCCATAAGAGTTTTGTATTTAATCTTGTTGGCATGTCCGGAAAAAGCT

53821GATTCATCAATAATTGTTGATGTATGAACAGCTTTACCATACGTCTCAATTAATTCTCTT

53881AATTTAAAAGCCTGCTCACTTGTCATTACATACTCCTTTGAATAATATCAATAATGTTGT

53941TCACCAGATTATAAGTAAACATTGGGTAATTATATTGAATCATCACATACACAACAAACA

54001AAACTTTCATTCTCTTCTCCTCAGCAGTTGACAAGATTACTATACCATAACCTTGCCAAC

54061TTGTAAACCATTAAATGACGTTTTCAATAAAATTCTGAAGCTTTGTATGAGCATCAACCA

54121TGATTTTCAATTCTTCCTTTGCGAAAGCTATACCTCTTTCTCTAGCAGAATACTCATAGT

54181CAGAAACTGCATTAGCATATTCTTCAATTAGCTTCATTAAAAACATCTGCTTTTCAGTTT

54241TCATTATTCCACCTAATCATTTCAAGATATTGAACTAACTTAGCTTTAGATTTATCCAAA

54301TCCTTTTTAGCTGCTTCTATACCATCGTATGAATATCCTTCACAATGCTCAACTGCTAAT

54361TGATATGAATCTATTTCAATATCACGTGCTAATTTAATGATTTTTTCAAACTGTTCGCGT

54421GTTAGCATACTTACACTCTCGTATTATGATCGATAATTTCATCAAGAAACATATCTAACG

54481CTTCTAAAGCATTATCAACTTTAGTTTCAAAGTCTTTAATGCCTTTATTCGAAATACCCG

54541ATGAATACCAAGCAAAGTCAATCAGCTCTTCATCTGCTTTGCGAATTGAAGCCACTAATT

54601CCTTAATTTTATCTGCTTGTTCAATGCTAGTCATTATTCCACCACATATGAAAGAGAGAA

54661TATTGCACACGCCATATGAGTTGCAGCTTCATCACACATATCATAACGTTTCTTAAGAAG

54721TTCTACAAGTTCTTCACTAGTAACTTCATCCATGTCGACGAAAAAATCACCATTAATGAT

54781GACGTAGATATTTCCTTCTTGATTGAGTGCTTCAATTTTCATGATGTTCTCCTCTTTATC

54841CGATGGTTGTATAGTATCACAGCTCAAATTGAAAGTAAACTGGTAAAATGAAAAAAGTCT

54901CCCGAAGGAGACTAATGTTATTCGAGGGAAAGAAGATATTTACTCTGGTAAAACATCCCA

54961GTAATATCATCTATCGTGCTTTGGATGGCTGGAGGCATTTCTTTATAAATGCTGTTAGAT

55021TGGTCTAGTATGCGATCAATCATTTTAATTGTGTCGGTAGGAAGTTTACTGGCATCTGGA

55081ATTGAAGGTGTGTATTTTCTACCAGAATACCCCAAATATTGCTCACCAAATTTATCAATC

55141AAATCTGGCAACTCAGAGAAAATAAAATCGTATGCTTTGTGTCTAGCATAACTTTTAGTT

55201TCAAAATGTGCAGAATGAAAATAAGCTTGTGCAGCCATTAATAAACCTAAGTATTCATCT

55261GCCTTTGAAGGTTTTCCACTTTGTGAAAAGTCGCTGAATTTCATTCAGTCTCCAATTTAA

55321TGTTCATAATTCTAGCGTATGATTGTGCCATCTCCGCGCCTCGCTCTATACATTCAAAAT

55381CGGAAGAGCACGGGTCATTTTTATAGGTCGTTCGCATAAAACTATAGAACTGTTCAGATG

55441ATTCTACGCTTTTATTTTCAAAAAGCATATAAACATGTCTAATGCCAGATTCCATAAATT

55501TATCAAAATGAGGATCGACATTCGCTTCAATCGGTGGAGATAAAGCAAACGCTAATCCTA

55561GCATGGCAAATAGTGCCGTTGCTTTTAAGGCCATAAAGGCCTCCTATCATTTTTGTCCTG

55621TATTTACTTTATGCCGATGCACGGCCTTAACTTTATCAAGGTATTTTTCAAAATTTCGCA

55681ATCTAGTATAGTCTGCCGGAGATTGGTTGAGTGATACTTCTCGACGCAAAGCTGAAATGA

55741TATTTCCAACTTCCCTACGAATTTCATCTAATTGAAGAACAGTAAGATTGCGAAGTTGCT

55801TTTCAGTTAATTGTAACATATATACCCCTTTAGTTAGATAAACCTATTTATAACTTTTGC

55861ACTAACCGAGCTTTTTAGTTAATTCATTCCAATGTTTTCTGCACAAAGAAACATAAATTT

55921CATCACCAATACAAATTTGATTACCTTCTTTAACTGGTGTTCCATCTTCCATTAATCGAG

55981CTGTCATAATAGCTTTTTTACCACAATGACAAACTGCTTTTAGTTCAATAAGTTTATCTG

56041CAATCGCCAAAAGTTCTTTAGAACCTTCAAATAATTTTCCAGCGAAATCAGTCCTTAGCC

56101CATAAGCCATAACAGGAACATTATATGTATCAACAATTCGACTTAATTGATGCACCTGTT

56161CAGTTTTTAAAAACTGAGCTTCATCTACAAATACACAATGAATATCTTTTTGTGCTTCAG

56221CCCATTTATAGAACTCAAAAATATCCATATCATCCGTAATAATATTCGCTTCCTGCTTAA

56281TTCCAATGCGAGAAACGACTTCACAGACAGAATCGCGAGTATCAATAGCAGGCTTAAGAA

56341CTAATACACCCATTCCGCGTTCTTTATAATTATGTGCAGCAGTCAAAAGAGAAGCAGATT

56401TTCCAGCATTCATTGCTGCGTAAGTAAAAATTAAACTCGCCATATCACCTTCTTAAAGCA

56461TATTCACATAAGCTATTAATTCGTTTTCTTTCTTATCAAACCGATCAGCAAATTCTTCTT

56521GCTGTTCGGGAGATAGCGGGCCGTATTCATCATAAAAGGTGTTTGCTTCTGTATGCTCAT

56581CTAGAAGTTCATGGATAAGCTCAAACAATTTATCTTTTTGTTCTTTACTCAGACTCATAT

56641ATTAATTTCCAGGAGTTTTAAAATAATTCGCTTTTGCTTAAATATTTCCATAGTTAAATG

56701ACCAGTATCTTTTTGACCGATTCCAATGGCAAAACCTTTATTGACAGCGAGTTCAATTAA

56761ATTATCAAGCTCACGTAACACTTCATACTGTAACTCAAAACTATTCATTTCGGTTTACCT

56821TCTTTAACGAAATCTGGATAAGGATAAAATCCCGAATAAAATCCTTCACCTTTATGATTG

56881ATGCATCCTTTATTAGAACAATAGCACCAATAATCGAAATCACAAGCCATTTCATCGTTG

56941CATAAAGCAAAAACAACTGGCCATTTACACTTTTCACAGTGGGCATTTTTAAGAATACTA

57001GTTTGGCTCATAACCATGTAACCTTTAAGCAATATTCTTCTACATGTTGTTTACGACCTT

57061TCTTATCAATAAAGGTATATTCAACGAATGTTCCAATGTAGTCTTTATCTACATCATGTG

57121GACTATTAATTGGACATTTAGTACGGCAAATGCGTTCCCATTGACGAATAATTACTGCCT

57181TATTCTTTGGGTCATATGGATGTGGATAATGTATATTCATAATAACGGTTCCCAATCAAC

57241AATCACAATTTCTAATTTAGAGGAATATGTATCTAAAATCCCCTCAATAATATCCCAGTT

57301CCCTTTACCTATGCCTGCACCAATCCTAGGCATATAGATTGTAGGTTTAATCAGTTTATT

57361TTCACCAAACTCATTTAATTCTAACATACAATTCATTAAAGCGGAATACTCAAAATTTGG

57421CCCTGGTTGAAATTGAGTATAAAGGTTGAAGCAATACGCTTTATGAGTCCTAAAGTATTT

57481TTCATAGACTGAGTAAGAACCGAGTTTAGTTACATCACCCCATTCAGTCTGTAATTTATC

57541AGCTTCCAAAATTTTAGGGAAAGCTTTGGTTAATTGACCCGCTACGCCTGAACCCATAGT

57601ATGAAAACAATTGCATCCATGCGCAATATTTTTACCTTCAGCGAAAAGGGCGACAATATC

57661GCCCTTGATATATTTTACAATCATCTAGTACTCAATCCTCGATTATAAGAATCTACCAAA

57721CGGTCAACCATTGAATGACAAGCGGCTTTATCTTTCTCCTCCGCAACTGAACATTCTAAG

57781GTATTCCACTTTTTAGCATATCGTTTTAATAATGTATCGTTTTTGTATCTGCTTGATTTA

57841TCTCTTTCTCCGTCTTTATATGCATATATTAATTTCTGCGCAAATTCAGCTTGGCATGCT

57901TTATTTTTCCCACAATAATCCGCGGCAGTACGGTTTACATACTCTCTAATTTCAGTATAT

57961GATGTATCTGCTAACGCAGAAAATGAAATTAATCCTATACATAAAACCAAAATTTTAGTC

58021ATTTACTATTTCCAAAAGTTTATTATTTTTAAGGTAATTAGCCTTTTCTAGGACTTCAGA

58081AGCATATTTAGAACCAGCTTTAACATTCCATCCCGAATTATAAGAGGATATTGCTTTTCT

58141TATATCGCCCTTATGTATATTTAACCAATAAGAAAGTTCAATGTACGCCCAGGAAGCTGA

58201ATTGGACCGTTTATTCAACATTCTTTTTATTTCAGCATCGGTCATATTATAACCAAGTTC

58261CTTGACTCTCGCTCGCATAGTAGGCAAATAATTTTGGAACATTCCATAGGCATGATGCTT

58321TGGTTTAGATTTTAAATTAACTCCGCCAGAGCTTTCTTGCCATAAAATAGCAGCCATTAT

58381ATGACCTAATCCGCTCTTATGGATATTTTTGTGTGTTTTATATTTTCCATCCTTAGAAAA

58441TTGTTCCCCGAATTGATACGCGTAACGCATGTTATCGAGTTGGACATTACTGAAAGTATG

58501CTCGGAGCTATGTGCCATCATTGAAATGGCCAATAGACCAGCGAGTAGTGCTTTTCTCAT

58561GCTTACCTCATTGAGTTTTAATTACTGCTTTAGAAGCCTTTCCTGGTAAACGACGACTAT

58621TGATAATTGCCATCCTGCATTGAAGCGACGGGTCTTTGAACTTCGCATTAGGTTTACAAA

58681CTGTAAATCCAAGCCAAAGATTTCCATCTGTGATTTCTAAACGTCCAGGACGGTATTCAA

58741CCCCATCAATAAAATCCTCATCAATGTCAGGACGCGGAGGCATACTCAGGAATTCATTAA

58801CTTCTAAAACATGGTCTTTTATTTTGTGGAATAATTCAAAAACATATGTCTCATCAATCT

58861CCCGTTGAATTGCACGATCAAGAAGATGCTGAGAATATTTTAGATGAAACGATGAGACCC

58921CTGCTGCTTTTGATGCCTCACGAATCTCATTGTTAATTTGACGAAACTCCGACTCAAAGT

58981GACGACGAAGCTTATTTCGACGGATAAAAACTTCTGTATTGATAGTCATGTTGTTCTCCT

59041CTTAACTGATAGAAAAATTATACCACAGTCAAGAGGAAAAGTAAACAGTTATTCTTTAAA

59101TCTAATCAATTTATTCATAGACTTTGAAACTTCTGCACGAACCTCATGTAGATTTTTGAG

59161CTGTTCAAGACGCTGCTCATAGTAAGCAATTTCATCTTCTTCGAGACAGTCCTGTGAATC

59221TTCTTTAAGATACCGTGCATAGTCCTGGAAAGCGTTACGGACTACTTCCTGGAAGTCATC

59281AAGACTTTGAATTTTCTTAGGAGCAGCAGACACACGACGAGGGGCAGTATAATACTCATA

59341ACCAAACCCTGCGCTTAATTGAGCCATTAATATTTTTCCTCTGGTTGGAACACAGCACGA

59401CAAGCCCACATACTAGCTTCTTTGAGTTTCGTTTTAGCAATAGCTAACTGATCGAGGCTT

59461TCAGCATAATTCTTCGCGAGTTCAAAGTCTTCGCAATTATCTAGTGCTTCCCAGAATTCA

59521TCATATAAAGCATCAAAGATAAGTCCTAAACGAACTTCAGCGTCTTTAATAGCATTTACT

59581TTACCGATTTTTTCGTCGGTATGTGGCTTATAACCTTTAATATCTTCAATCATATTTGAC

59641TTCCTCACCAGTACATAAATCGTATTCAACTAAACGAATAGGTTCATGAATGCCATATCC

59701CTGAACAGAAATTTCTGTCGTAGGATAAATTCCACTAATATCACCCATATTCCACGCTTC

59761ATTAAATTGCTGTTCGCCTGAATTACTAAACCATTCTGCGAAAGCATTTAACACATCTTT

59821AGAACCTTCAATAATTATCTTTGCCATTACAAGCTCTCAGTAAAGGTACGAGCGATAACG

59881TCGCGCTGCTGTTCCGGAGTCAGAGAGTTAAAGCGAACTGCATAACCGGATACACGGATG

59941GTCAGCTGCGGATATTTTTCCGGATGCTTAACTGCATCTTCCAGAGTTTCACGACGCAGA

60001ACGTTAACGTTCAGGTGTTGACCACCTTCAATTTTAACTGTAGGCTGTTGCTCAATTTCA

60061ATTTCACGGGCATGCAAACCATAGAAAATTTCTGGGTCTACAAAAGAGTCCTCTTTAAAG

60121GTTTTAGAGACAATAATTCGTGCTTGAATACCATCTTCAAAATAAATAGTACCTTTATGT

60181GTGCCTTCAAGAATTTGATATGCTTTCATATAACCTCAATTAGAAAATAATTTTATCCAA

60241GATTGTTCTTTAATTAAAAATGGCTCAGAATCATATGCCATTAAACTTTGAGTAATTAAT

60301CCTTTAAATGGTCCATCAATAAATTCCATGGTAAAATATGGAATTTTATTCATTAATCTT

60361GCATTAGGAGCAGTGCACAAAACTCTGCATCCTTTGAATACACCTTTTTGTAATTTGTAT

60421TGCTTGGGATAAAATTCGCTCAAGACGTTGTTATTTGCCAAAATTTCAAAATGATTCACC

60481AATTTATTTTTAATAGTTTTTGGCGAAAAATAAAGATATTCGAAAAGTTGCGTATCTGTC

60541ATCATTGCATTCCAATTACGAAAAACTGCGGACGAGTAATACCGCCAATGCAGCATTTAC

60601TATTGCAGCAGTGTTGCACAGTTTCAATATGAATATCGTAAATCTTATCCATATCAGGAG

60661ATTTGACAGGCTCATCAATTATATACAAAATTCGCGAAAGCGATAAACCTCTGAACTTGC

60721TTCCTTTATTACCAATAAAACTGCGTACAGAATCAGTAAATAAACGAAAACGTATATCAT

60781CATTAGAATAACGTGAAAATTCCTTTTTAATGTTATTTGCAGAAATTTTAGCGTAAGCTG

60841AAGTATTAGAAAGAACAATAACTGTTCCGCCATCATACAACCAATTAGCAGCAAAGTTAG

60901TCACAGCAATTGATTTACCAGATTGACGTCCACCGTCTAGTCGAAGTGTGCAATACTGTT

60961TAAGCAAGTCTTCAAATGGCAGGATATATTCGTTTTTACAAATTTCTTCTACTCTAGCAT

61021CAGAATGGTGTGTAAAAGCATTCATCAGGGATAGATAAGGACCAGTTAAAAATGTTCTCA

61081TTTTATTCTCTCTAAATTTGGGCCATTCCATGGCGCATGAATTGTCCATTTCTGTATTTA

61141CCCATTACCGCACTTGGGCTCGACCTTATTACAGGTTGGTGGGAATCCCTCACAGAATCA

61201TGAGGTCCAGGTTATTCCCATGTTATTTAAATGTAAATATTTTTGCCGTAATACTTATAC

61261CAGTGTGGCTTCATTAAAATTTTTTCATCGAGTCGTTGTTGACTCAACTTAATAGCTGAT

61321TTACATGGATTATAATCATTTTTCCATTCTACTGGAATATCGCTGATGTCAGGAACTTCA

61381GTATCTTTTATACTGAACCCGCGTTTTAAGCATTCCGTTATAATGTCCGATTGACGCTTT

61441CGCAAAAATTCTAACTTATCGTAAAAGAAAGTAACATGACCAGAACCTAAAATAAATTTA

61501GAAGATATTTTAAAATCTTTAACGCGCTTACCGTTTGCCACATGCTTACGAACTATACCA

61561AAAACACGCGGCAATTCACGGTATTCTGCGATTAAATGTTGATCAGCAAGTTCAGATACT

61621AAAGTCAAATTAATACGAGTCATTTTATCCCTCCAAGTAACTGTGAATATACTATCACAA

61681TTCTAGGAGAAAGTAAACAACTTTATAGATTTTTATACGCGTCCCAAGTGCCAGTTCTAA

61741ACGTTGCAATGACTCGTTTTGCGCGATTAGGTGTTTGATTATACCATCTACTTTTAGCTA

61801AGTTAACTGCTGCTTCATCCCAGCGTTTTTGTTGAAGCATACGCAAAGAGTTAGTGAATC

61861CAGCTACGCCAGTTTCTCCCATTTGGAAAACCATATTAATTAATGCACAGCGACGAACAG

61921CATCAAGAGAATCATAAACTGGTTTTAATTTAGCATTTCTCAAAATTCCACGAACAGCAG

61981CATCAACATCCTGATTAAAGAGTTTTTCAGCCTCATCTTTTGTAATTACACCATTACAAT

62041TACGCCCAATAGCTTTATCTAATTCAGATTTAGCAACACTTAGTGATGGACTTTTAGTAA

62101GCAAATGACCAATGCCAATAGTGTAATAACCTTCTGTGTCTTTATAGATTTTAAGTCTAA

62161GACCTTCATCTATACGTAACATTTCAAATATATTCATAATACCTCCTAAGTATTTATAGA

62221AGGTATTTATAAATTAAAAGAGGCTGTTCATTATTCGGTAAAGTGAAGGACCCATCACAT

62281ATTGCCACTGAGTACGAGGAATAAGAGCAAAAGCATCCATCTCTGGAATCATAACGCCAT

62341CTTTATTTTCAAAATAAGACTCGCAATGGCAATTTCTAAACATCTCATGCTCTACTGGAA

62401TCGTATAATAAAATAACTGTAAGTCTTTATTACTAGAATATTTAAATACACCTAAGTCTT

62461CTAGAAGGTCTGGATTATAATCGATAAAACCAGTCTCTTCTGAGCATTCTCTTTTTGCAG

62521CTTCCAGTGCATTCAAATCAGAACTTTCTACACGCCCCTTTGGAATATCCCAGCGATGTG

62581CAATCATTCCAGGTTTACGAGAACCGGTAACTCGTCCCATAAATAAATCTTTATCTTCTG

62641TCATAAAGATAATACCAGCTGATAATGTTTTCATTTTAATTTCCTGCATTCAGTGATAAA

62701GCTATTTAAATTTTGGGTATATTTCTTTTCATCAAAAATCTTTTGTTGTCTGCGTAAACG

62761CCATGGCATTTCAATGAACGTGTACCATATCCCAGATAATATTGCTGCTGTAAAAATATT

62821AACAAGTATGGTTAAAATAATCCAATCTCCCGTTCTGTCTACTGGACTTTCTCCAAAGAA

62881GCAAAATACAAATGATGTAACAGGAAAACTAATAATGTACCACAGAATCATAATTTTATC

62941TGTGAACCAATTAGCGTTCGTTAACTTAGCGCGACCATTATGAATACATACGAACTTATC

63001ATCTGTTATAGTAGATGGCTTAACTGGTTGATATCCCATTCTAAACTCCCTAATCAACAA

63061ATTTTTCATATCTTCGGAACAACCACTCCAGTCAACTCTATCAACTGAAATGCCATCATC

63121CCCATCATCTAAATCATACCAGCGAGTTTTTAAAATCATTTAATTTTCCTACAATCACTC

63181ACAAACTCTTCCATTGATTCATTTTCAATATAAGACATGTAGTTATTATATTCTTTTAAT

63241TGTATTTTGTAATCCTTTTTTCTTTGCCAATTTATTTTAAAATTATCATAATGAAAATAT

63301AACATGGTACCAAAGAATGAAAATAATGAAATCGCTATAGTATAACGAAGTTCACTCCAA

63361ACCTCTGTTATAATTACTGTGCCGTCGATGTTTAAAATAAAACAGTCAATTAACAATCCA

63421ATAAGACTACCTGCAAGAGCTGCAAATGCTGATACGGCAAGAATTAATAGTGCCTCAGGA

63481AACGAATATTTAACTTTATTTAGTTTTGGCTTTTGCATCGTGATTCCTTAACAAATTTCA

63541TAATTTCATCAAATTCATACGCAGCAAGTTTAAGCTGGTGTTCCTTTTTAATCTTTTTAC

63601ACTGTGCTTTCCAATCACGTACGCGTTTACGATAATGTCTTCCTTGATACCAGTATCCTA

63661CCCAATTTACAGGTACTAATAAAAGCGGAACTACCAATGGAAGAATTAGTGTTGCTCCAA

63721ATATTGCACCAGATTCAATATCAGTCATAACGTCTATAACTATTCCAGCAATCAACAGAA

63781TCACAAATGATACGGCTACCACAGGACCTATTAATACATCAGTAGAAATTAGCTGGCGCT

63841TTAATTCATACTTCAAAGGTTTACTTGGAAGGTATAGTGATGGTTTTGACATATTCTCTA

63901CATTCCTTAACAAATTTTTCTAGTAATAAATCGCTTTCAAAATTGGGATTTTCTACGAAT

63961TTATCAAAAAGATCATCAACAATAGTTAAGATATTCTTTTTACTAAGAATACGTTTATTT

64021TCATGCTTCGTTTCAGAATCAACTATAAGAGTAAAGAAATATTTCTTGCCCTGAAATTTT

64081ACAGTAGTATCAATATAGAATAAATTTGACTTTTGTAAATTACGTTTAAACCATGCATCA

64141CTTAAACTATAAACGCCAAGATAATCATAATCGTCGTTTAAATAACATACTGTCCATTCA

64201GGAGAAATGAAATCAGTAAATTCAACATCAAAATCACATGTTAATGAATGAATTGATTCA

64261ATACTGTTAATAAGTATTCCAGGACGTATTAAAGACTTTTTACCTTTATAAAATCCAGAA

64321AGACTTTCATCAGTTTCATATGAAGAACCCCAATAATAATTACGTCCTTCTGCCATACGT

64381TTAAGAGCATTTAATAATTGGTCTGGAACATCAACCTGTCTTTGGAATTCTTCAAACATT

64441GAATTGAAATCACTTTGCATTTTCATTCCTACTTACTCCAAGTAATAGGGGCCGAAGCCC

64501CTTATCATTATTTCAGAGAATTAATGTATTCCTGAACATCGGCAGAGGTAGTTTCAACCC

64561CAGAAATATTGCCATTAAAGGTTTCAACTCGAGCAAGAGTATCTTCAATATCAACCTTAG

64621TCAGTGCTGCAATTTCAACTACATCATCAGCAGTACTAATTCCGAGGGCATTCGCGGCGC

64681GAGTTTCACGGATATATTCCAATTTAACTGCAAGTTCTTGGCGAGCATCATCTAACTCAA

64741CTACTTTCTTAGCGATTTCAATTCGCATTTCAGCATAACCATCAGCTTTAGTAGTTAACT

64801GCTCAGCTGTTCGACGATATAGTAAACCGAGTTTAGCATGCATTGTTACATCTTGACCTT

64861CAGAAAGAAGCTTGCGAATTTCACGCTCTTTTGATTCGGCCTGTCGATTCTTTTCGACAA

64921TAAGTTCACGAATACGCTTTTCTTCATTAATAGATTTAACAGAAGCAGTTTTTAGATCTT

64981TAATTTTATCAAGCAATTTTGCTGCTGCGGCAGTATACTGCTCTTCAACAGATAGATTTT

65041TAGCCATAGCAGAACCAAGTTTAGTGCGAATAAACTCAACAATTTTCTTCAGCATGTTCA

65101TAGCATTTCCTTTAGTTAATTAAGGGTTTTATAATCCGTGGGAGTATTATACTCTACTCC

65161CGAGAGTTTGTAAACATGTTATTTCATTTTTGCGAGGCAGATTTCATTGGCTTCCCATTG

65221AGCTTGCTTAATAATAGCTTTGGATACAGGAACCTTTTGACCATAAGTTTTTGACGTTGA

65281GAGAACAATATATTTGTTTGCCTCATTATGCCAACCCCATGATTCTTCGCCATGAGTACC

65341TTTAACTACAGCTGACACCTCAACAGAACATCCATCATCACCAATCTTGGTGTCTACAAT

65401CCATTCATTTCTCTCGACAGCTTCATTTTCCGATCTCATTTTTTCTTCATACACGGCGAC

65461CCAATCAGTTTTTGTCAAGCTTAAACTAAGAGCTTTCTCTAATTCTTTAACAGATTTTTG

65521TTGTTCGGGTGTTAATGAATTATATAACTCTCGCATTTCTTTATCTTCAATATTCATTAT

65581GTTCTCCTCAAAGTTATAGGCTCATAATATCTCAATCATGAGCCTGTGTAAACTTATTTC

65641ATATTATTGAAATATTCTTCAGCAATTTCATCATTATCATGATAAACTTTAGAAGACAGT

65701TTAACATAACTTTCAGCAGTGAACATGTTAATCACAACCTTTACAGTATACCACTGACCG

65761TCTTCATTACCCATTACTGCATAAGTTTCAAACATCGGATGATCAGGACCGATAACTTTA

65821ATATCATTCACTGTACGACCAAAATCTTCTGAAACACATTTCATAAAGAAGTTGAACAGT

65881TCGCCGTAATTATCCATTTCATTCTCCAAGTTGTTTTCTGTATCAGTAGTTGATAGTTGT

65941ATAGTACCATGGAAGGGTAAGGATGTAAACCGTTTTGTGAAAAAATTTTTTAAAAAGTTT

66001TGGGGAATTCTAGGGCAGGGAGGGGAAATCAAAGGATAGGATAATATATTATAAAGGGTA

66061GAAACTAAATGATGTCTAGAGAGGCCTGGAAAGGCCTAGATACCAAAAAGCCCCAACCTT

66121TCGGCTGGGGCTAAGAATGTTATTTGATTTGTTTAGCAGACCAAATGCGGTCTTTAATAA

66181TTTTTTGGATGTCTTCAACATACTCTAGGTCATGAGCATGTGGGTTATCTTTGAAACTAT

66241AAGCACGAGCCAGTTTTTGGCCTTCAGTTTTGATTACTAAAAGCTCTTTAAGGATAGCTT

66301CATATTTGGAGATAATTCCTTTAACAATATTTTTTTCTTGTGTAGCTTTAGGATCTGCTT

66361TAGGAGCAGGTTTACCCGAAGCCTTTGCAAAAGCAGCGCCAGTAGCAACTAAACTTTTCC

66421AGGCCATACTAACTGCATTCCCTGTAAATCCTTCGGCTTTCATATCACGAGCAAATTGGA

66481ATCGTGATTCATCGGAAGCATCTTTATAGGAATATTTACCCGCGGCAATGGCTGCTTTTG

66541CGACAGCTTGAATTTCGGTGCTAGATGCTTCATTTAACACCGCTTCATTTAAAAATTGAG

66601CATATGATTTCATCTTATTTCCTGTTTTAATTCGTGGTTTTAATATACTTATTTATACCA

66661AAAAGCCCCAACCTTTCGGTCGGGGCTAAGCCTTGCGGCAACCTTGTCGGGGTTCCACCT

66721GCTAAGGCAAGTGTTTGTACGAAACGCCGGGATTCGAACCCGGTTATTAAGCAGTTGACG

66781CTACTCAATATTTTTAAAAGGCCATATCTCGACCATATCCGAACGTTCCGTCAAAAACGC

66841TACTCGGCTTACGGCAAAGATATTTCCTCGAATCGATAATTCGGTGCGCCGTTTCTGCTG

66901TGATATAAGAGGGCATTAACAAAACATAAAGATTTATTAATGCCAGTCCTTAAACAGGGA

66961ACATCAGTCCGACGACTTACCGGTAGCGACCCGGTTTCTTAATATTCTTTTAAAGCATCA

67021ATTTGTTCACGGCGTAAACGCCATAAATCAATTGCTTCATAAGCAGATTCAGCATTTCGA

67081ATTAGCCGACATTTATGTAAAGACTTATGATTTTGATATTGTGTAGATAACGGAATTTTA

67141TCTAAAAGGTCTTTACGTTCAAAATATTCGTAACCATCTAAACCAAATGAACGATTTTTG

67201AGAAATTGCCAATGACCAAATTCGTTTTCGACATAAACATAATCTGCATGCTGATAAAGC

67261AAATTAATTACACGTTCAGAAATAACAGTATCATTATGATTAAAATAGAATGTTAAATCA

67321TGAACTACTAAATAAACATTACCTTTCATATTTTCCTCACTTATAATTGGTCGAGACAGA

67381AGGATTCTAACCTTCAACCTACGGATTAGAAGTCCGTTGCTCTATACAATTGAGCTATGC

67441CTCGTATTTGGCGGACGTGATAGGATTCGAACCTATAACCAATCGCTTAACAGGCGATCG

67501CTCTGCCATTGAGCTACACATCCAAATTGGTGGGGAGTGGTGGAGTCGAACCACCCGAGT

67561CGCAATGACAATGAATTTACAGTCCACACCGCTACCTCTACGGGATAACTCCCCAAATTA

67621ATTTGGTGGCCCTGGGTGGAATTGAACCACCATCTGGCGATTATGAGTCGCTTGCTTGAA

67681CCTTCCAGCTACAGGGCCTTGGTGCTGATTGACGGAATCGAACCGCCGACATCCTCATTA

67741CAAGTGAGGTGCTCTACCTACTGAGCTAAATCAGCAAAACTGGCGGAGGCGATAGGATTT

67801GAACCTATGAGTCGCCGGAGCGACTGCCGGTTTTCAAGACCGGTGCATTAAACCACTCTG

67861CCACGCCTCCAGTCTCCATACAAGGATTTGAACCTTGGACCTCCTGATCCCAAATCAGGC

67921GCTCTACCAAACTGAGCTACACGGAGTAAATTAAATTGGAGCGGATAATGAGAATCGAAC

67981TCACATCATCAGATTGGAAGTCTGAGGTAATACCATTATACGATATCCGCAAATTTGGTG

68041CGAGAAGTGGGACTCGAACCCACAAGGAAATCATTCCGCAGCATTTTAAGTGCTGTGCCT

68101TTACCAATTTGACCATTCTCGCGCTGGGAATAAAGGACTCGAACCTTTGCATCCTGGAAT

68161CAAAATCCAGTGCCTTACCAACTTGGCTAATTCCCAATTATTAACAAAGGCTCTTAAGCA

68221AGAACCCTTGATGATAGAGGGTATTAATCAGTGCGATATGAGTTAATAATAACAAATAAT

68281TCTTAAAGCATATTTACCATTTATAATAATACATATTTACGATGCATTCAAGACCCAAAG

68341GATTCTTGAAAATATCATATTCAAGAGGACCTTTTTCTGTTTCAATAAAGAAATCAAAAT

68401TTACTGTATTAAATTTACGTTCTTCCTTCACTAATTTAACTTGAGAAGATGAACGGTCAA

68461TGTAAACCTTTTCAACTTCAAAACATGTTAAAATGCCATAATCATCAATCAAAGCTTTAG

68521CCGCGTCTTGATCATATTTATATCCATTTTCAATGGATGATACTTTAGCATAAAGAATCA

68581TTATCAGCCTTCATCAACAATAGTGTGAGTATTAGCATTTACGATTTGCCACCAATCAAA

68641GCGATTAGAATCCATCGGTTTGTTTTCATTTTCTTTGATAATGTCACGGAGTTCATCTTC

68701AGAGAATGCTTTAGCGATTAAATCGGTATACCCACCACGTGGATAATAATTATCACCTGC

68761AAACAAAAGGAAATTTACCTTCCCGGAAGGAACATACGCTTCCTTAGGATACTTGTTTCC

68821TGCGTGGTCAACCACTTCAATATAACGGTAAGGAATATCGGTTCTTTCAACCCATTGCCA

68881TGCCGCCGCAGGGGAATCGAAAGCATCTACACCTAAACGATTATCTTCATCTTTAGACGG

68941ATTATTTTCGTAATCCGCATATACATAATATTCAACGTTCATTATTCACCTTTAGAAATT

69001TTATCCATAACAATAGCAATTAAACCAATTAAAAATGCTACTACAAGTGAAAAAACATTT

69061TCTGCCGTAGTCAATAATCCGCATATAAATCCAACAAACATTGAAAAACTGAAAGCGGAA

69121GCAGAAATTGCAATAGCAACATTTCGAATTAATTCACAACGTTTCATTTTATTCTCCTCA

69181GTAGTAGATAGGGTAATAGTATCACTACCCTATCTAAAAGTAAACTTATTTTTTACGAAA

69241AATTGATTTATTTTCTGCTGCCCATTTTTCAATAACTGCAGCAGGACCAGTAACAACAAT

69301TTTATCACCGTAATCTTCTGCGGCGAGTTGACCGAAATTATCTAATGCATCACGAAGAAC

69361TCCATGAGGCATTTCACCCATTTTATCTTTCTTGCCAGTGTAAATAAACTCAACTTTTAC

69421ATCAGCAGTTTCAGCAATAAATTCTTGGTAAGTTTTCATTTTGATTTCCATTTGGTTTTG

69481TTTTGATAGGGTAATAGTATCACAACTAAAACCCTATGTAAACAACTTTGTGAAATTATT

69541TTAAATCTTCTAATCGTTTCTTCATCTTAGAACCATTTTTAGCAATTTCTCCTGCATCGG

69601AGCATAATGCTAAAAGTGCTTTAACTTTAGTTTGGTCTCCTTTAAAAGATGAAGGATCGA

69661CCGCAGCAGCAAGTTCTTTAATACGAGAGAAAAGACGCTCAGCTTCAGCTAAAGCCATTC

69721CAAGCTTTACTTCCATACCGTGGGAAGATTCAGTGATAGTAGTTTTTGTAGCAAACTCTT

69781TGAAAGTTTTCATTTTTATTTTCCTAATTAATTTTGATGAGGTAATAGTATCACTACCTC

69841ATCAGTATGTAAACAACTTTGTGAAATTATTTTAAATCATCTGCCCAATCGAGTTTAAGA

69901GGCTCTTTGTATTCACGGTCAAGTACAACCGGAATTTGTACATCACCGCTAAATGATAAG

69961GGCCCAACATTATAAGACAATGTTATATGCGGTGTGTAATCATCAAAATCGTGTGTAGCA

70021CCTAGTGCCCGCGCATACATGTGTCGACAGCGCAGATATTCAGAATCTAGCACAAGTACA

70081AGAGTCGATCCATCTTGTGTTTTCCACACTTCTAAATGTCCAGAAGAAGCTACTTCAAAA

70141CTTCCACTCGATGGAACATATGGAACATTTACTCTTGAATAACATATAGTCGAATGAATT

70201TTTTCTCTAGGAACTGGATTAGGAACACGTAAAGAGCGCTGGAGTTCTTCCAGCGCGTCA

70261AGTGTTAATTCTGAAAACTTAGCTGCTACATAAAGACCCGTTGAAAAGTCTTTAAATTCC

70321ATCATTCTTCATCTGCAGATTCAGCAGTAAGATTCTTGACAGCTTCAACGATTTCTTCAA

70381CTTTAATAGTATCGCCAGTGATACCTACTGCATGAGCAATTTCAGCCAAAGTTCCTTGCA

70441GAATTTTGGATTCTTCCATCAGACGAGCCGCTTGGTCCTGCGTATCAAGAATACGAGATT

70501TCAGAGTTACGATTTCAGCAGACAGTTTTTGTTCAATAGTTTGTTCAGACATTATAGTAC

70561CTTTAGTGTATTTTTAATTTTAGAAAAAAGTTCTTCAAGAGAACCATCGTTTGTAATTAC

70621TAAATCGCCATCACGAATTGGCAATCCAGCTTCTGTAATATGTGTATCATTGGATTTTTG

70681ACCAGGACGAACTACATGAATTACTGTAGCACCCATCGCCCTAGCCGCATCCATTTCATG

70741ATCTTGACGGGTATCAGGAACGATATAATAATCATAACCTGAGTTAAATTTATCAAGATA

70801ATCTAAAGCAAATAATTTTACCCAGTACATGCGGTCGAAGTTATTAACAATCAAATCCGT

70861ACCTAGGGCTTGCATCAGACGACGGACTGACCATTGATCTTCAATATTATTTATAACGTC

70921AATAATCTTGTTAAATGCTACGGAATTAACTGATTCTTGTCCTTCGTCATCAAAAACAAA

70981CACACCTTTAATTGGGCTTTTACCATTAAGATAGCAAAATGCTTGTTCCATAATCGTGAT

71041TACTTCTAATTTAGTTAGATTTAAATTAGTCTCACGATCATAGTCAATTCCTTCAAACTC

71101TTTACGAGTTAAGCAAGGATAGTCGGTATTTGCTGCAAATACTCCCCATGCATAAGCCAA

71161TGCATCCTTAATAGGACCAGCAAGTTGGTATTTAACTGCAGAATAATTGCTCATGATAAA

71221ATCAGCAGTAGTATCTTTTCCACTACGCTTTACACCGCTTAAAAAGATTAGTTTCATATG

71281TTTCTCCTCAAATTTAGTTAAGATTATAACACATGAAACTGAAGCATTAAACTTCCGCTA

71341TAATTTTTCCATCTTTTTCTACTTGAAAATACGTATAAGGAATCGTTGCTGTACATACTA

71401AAGCCGGGTCTGAATCTTCTGTGTAGCTAAATTCTACTTCAGACAGGTCAGAAACCCAAG

71461GCTTATAAAAATTTATTGACATCACGATTTCAGTTTTGCTATTATCTAAAATGTAAAGCG

71521TAATGTATTCAGGACCTGTTTTTTGGGCAGTATTTTCACCTGTAAGATAGTTGCTAGTTC

71581CTAGCATCCATTCATACATTCCTATCCATGACTTAAGTTCTTCATCAACTATAAATCTCA

71641CGATGAGTGGATCGTACTCAAATGTAGCACCTGGACGTTGTGCTCGGCCCAGTCCAAACG

71701GCCCAGTCACGGTATCAGTAACAGGTATTCTAATTCCTGGAATAGGAACTGACTGAGCAT

71761TTAAAGTAAAAGCAGATGTAGTATTACTATGTGGTATTGATACTACAAAGTTAGTTGTAT

71821TTGCTTGGTTAAAAATTTGTTGCAGTGCTTGCGACATATATTCCTCATAATGCTTTATAA

71881CTGTTGGTGGTATAATGGGTCTAAGTCCCTTCCATTCAATTCCATTTAGAACAAACAACA

71941GAAAAGAATGGAAGATAATAGAATTAGATATTTGACCAGACTTTGTTTGCAGAGAAACGT

72001TTTCCTTTTGAAACGAACTGCTGAAGTGGCATTAACACAACGTTCGCCCAGTCTTTCGGG

72061GCGATTTCAACAAGGCTACCCATAATATTACCAGGTATATATGCCTTAATCATTTGGTCT

72121GCACCCCTAAATCCTTTCACTTGACTCCAATCAATTTTTAATTTCGTTTTATTAGTAATA

72181GTAGGTGTATTTGAATATTGCTTTAAAAGCTCTTCTAGGAATTGCTGGCGAGCTTTAGGT

72241GGAATATAGTGCAAGTTCAATCCGTACATTAAATTATGCTTACCTAAACCAAGGTAAATT

72301ATCAAAGGAAATTTATCCCAGTAAGGAAGAGTTTCCTTGTGTTTAGCATCATAAGCAAAA

72361GCATATATTCGTCCCGGCTGCGGGCGAACAACTTTATGTCCTTTTACTTGCTTAATAGTT

72421TCAGCAAACCACTTTCTGGTTTTATTATTAATTGCTGCGCCTTCATTACGAATTTTATCG

72481CGCAATGTTTGTCTGAATGAATTTATCATAAGCAGTTGTCTTTCTTGCTTATTGAGTTTA

72541TTCATTGGTTTTGATTCAAGCTTTTGAATCTTTTCAGCCGTTTTAATTCCTGAAGCATAT

72601TTTGACATTGCTGAAGTAAACGTAGAGTATTTGATTCCTCTTTCTTCAGCAAATTGCTTT

72661CCGGTCATTCCTTTTGCTTTGGCCTTTTTATATTCAAGACCTATCTGAATCCATTTCTTT

72721TCGTTTAATGATTGCTTAACCTTTGGAACTTGGGGAGTGCTTTCATTAATTATTTGAAAA

72781ATAGCCATTATGCCCCCTTAAAGCCAAGAGCTCGTAATCCATCTTCTGTTAAAATTCTAA

72841ATTTTATTCCACGCTTTTCAGCTAAAGCTTGAGCTGCTTTCCATTTGTCAGTATTAACGG

72901AATATGTATAAATTTCATTCATAAATCTTTTCTTCGCTGCAGTTGTTAGATGTGCTGGTT

72961TAACTGGTGGTTGTGTTTCTTTTTTAGGTTTTATTTCAATAAAAAATTCTTGTCCAGAAG

73021AATCTTTCATCCAAATATCCATGAAGTATCTACGTTTTTTCCCTTCTGCATTACAAAAAT

73081AAGGAATTACTGCTGTTTCACTACCCCATGCAATAATTTCTGGATTTTTATCTAACCATT

73141CAAAAAAGAATTTTTCCCAATTTGATCTATACGTAATTTTTTTAGGGTCACCTCTATACT

73201TTGATATATTTTTAGGAACCCATTTTCCAGAATATGCCATTGGATTCTCCTTATAAATAG

73261ATAATATATTTATAAACAGGAGGGCCCATGCTCTTTACATTTTTTGATCCGATTGAATAT

73321GCGGCCAAAACGGTGAATAAAAACGCGCCGACTATTCCTATGACAGATATTTTTAGAAAC

73381TATAAAGACTATTTTAAACGCGCTCTTGCGGGATACCGCTTACGTACTTATTATATCAAA

73441GGTTCACCACGCCCGGAAGAATTAGCAAATACTATATATGGAAATCCACAGTTGTATTGG

73501GTTTTATTGATGTGTAATGATAATTATGACCCGTATTATGGATGGATTACTTCACAAGAA

73561GCAGCTTATCAAGCATCTATACAAAAATACAAAAATGTAGGTGGAGACCAAATAGTATAT

73621CATGTGAATGAGAACGGTGAAAAATTTTATAATTTAATATCATACGATGATAATCCATAT

73681GTTTGGTATGATAAAGGTGATAAAGCTAGAAAATATCCTCAATATGAAGGAGCACTTGCT

73741GCTGTTAATACGTATGAAGATGCTGTTCTTGAAAATGAAAAACTTCGTCAAATAAAAATA

73801ATAGCAAAATCAGACATTAATTCATTTATGAACGACCTTATACGTATAATGGAGAAATCT

73861TATGGAAATGATAAGTAATAACCTTAATTGGTTTGTTGGTGTTGTTGAAGATAGAATGGA

73921CCCATTAAAATTAGGTCGTGTTCGTGTTCGTGTGGTTGGTCTGCATCCACCTCAAAGAGC

73981ACAAGGCGATGTAATGGGTATTCCAACTGAAAAATTACCATGGATGTCAGTTATTCAACC

74041TATAACTTCTGCAGCAATGTCTGGAATTGGAGGTTCTGTTACTGGACCGGTAGAAGGAAC

74101TAGAGTTTATGGTCATTTTTTAGACAAATGGAAAACTAACGGAATTGTCCTTGGCACGTA

74161TGGTGGAATAGTTCGCGAAAAACCGAATAGACTTGAAGGATTTTCTGACCCAACTGGGCA

74221ATATCCTAGACGTTTAGGAAATGATACTAATGTATTAAACCAAGGCGGAGAAGTAGGATA

74281TGATTCGTCTTCTAACATTATCCAAGATAGTAACTTAGACACTGCAATAAATCCCGATGA

74341TAGACCACTATCAGAGATTCCAACCGATGATAATCCAAATATGTCAATGGCTGACATGCT

74401TCGCCGTGATGAAGGATTAAGACTAAAAGTTTATTGGGATACTGAAGGATATCCGACAAT

74461TGGTATTGGTCATCTTATCATGAAGCAGCCAGTTCGTGATATGGCTCAAATTAATAAAGT

74521TTTATCAAAACAAGTTGGTCGTGAAATTACTGGAAACCCAGGTTCTATTACGATGGAAGA

74581GGCGACGACTTTATTTGAACGTGATTTGGCTGATATGCAACGGGACATTAAATCACATTC

74641TAAAGTAGGACCAGTCTGGCAAGCTGTCAACCGTTCTCGTCAAATGGCGTTAGAAAATAT

74701GGCATTTCAAATGGGTGTTGGCGGTGTAGCTAAATTTAACACAATGTTAACTGCTATGTT

74761AGCCGGAGATTGGGAAAAAGCATATAAAGCCGGTCGTGATTCATTGTGGTATCAACAAAC

74821AAAAGGCCGTGCATCCCGTGTTACCATGATTATTCTTACGGGGAATTTGGAATCATATGG

74881TGTTGAAGTGAAAACCCCAGCTAGGTCTCTATCAGCAATGGCTGCTACTGTAGCTAAATC

74941TTCTGACCCGGCTGACCCTCCTATTCCAAATGACTCGAGAATTTTATTCAAAGAACCAGT

75001TTCTTCATATAAAGGTGAATATCCTTATGTGCATACAATGGAAACTGAAAGCGGACATAT

75061TCAGGAATTTGATGATACCCCTGGGCAAGAACGATACAGATTAGTTCATCCGACTGGAAC

75121TTATGAAGAAGTATCACCGTCAGGAAGAAGAACAAGAAAAACTGTCGATAATTTGTATGA

75181TATAACCAACGCTGATGGTAATTTTTTGGTAGCCGGTGATAAAAAGACTAACGTCGGTGG

75241ATCAGAAATTTATTACAACATGGATAATCGTCTTCACCAAATAGATGGAAGCAATACAAT

75301ATTTGTACGTGGCGATGAAACTAAGACAGTTGAAGGCAATGGAACTATCCTAGTTAAAGG

75361TAATGTTACTATTGTAGTTGAAGGTAATGCTGACATTACAGTTAAAGGAGATGCTACCAC

75421TTTAGTTGAAGGAAATCAAACTAACACAGTAAATGGAAATCTTTCTTGGAAAGTTGCTGG

75481GACAGTTGATTGGGACGTTGGTGGTGATTGGACAGAAAAAATGGCATCTATGAGTTCTAT

75541TTCATCTGGTCAATACACAATTGATGGATCGAGGATTGACATTGGCTAATATACTTCCAA

75601TGAGCGCTGATTTAGGAGAATCCATGGAAGGTTCTTCTATCGACGTCACCTTTACCGCTC

75661AATTAGAAACAGGTGAAACGTTAGTATCTATAAATATAACTAGTTACGAAGAAACTCCTG

75721GGGTTTTAGTAGAAGAAAATCGCTTATACGGAACATATGAATCTGTGTTTGGATTTGGAA

75781ATGACGCGTTGAAATATCGTTTAGGCGATGAATTTAAAACTGCTGCTTCATGGGAAGAAC

75841TTCCTACTGATTCTGATACTCAGTTGTATTTATGGAAAGCTCCTCAAAACCTCCAGAAGA

75901CATTCACTTACGAAGTAACATTAATATATGACTACCAAGAACAAAGTGAATCTGGAGGTT

75961CTGGCAGTAATTCTAGGTCATCCTCTGATACTACTGAACCGACGGATCCTCCTGCTCCAG

76021TAAGAAAAACTCTAGTTAAAAATTACACAAAAACTATAGTTGGAAATTGGAGTCGTTGGG

76081CTAATAAATTAAGAAGCTATGTGTATGAGAGGTCATAGATGTCAGGATTAAGTTATGATA

76141AGTGTGTTACTGCTGGCCATGAAGCATGGCCTCCAACAGTTGTGAATGCCACACAAAGTA

76201AAGTATTCACTGGAGGAATTGCTGTTCTCGTAGCAGGTGATCCAATTACAGAACATACAG

76261AAATTAAAAAGCCATATGAAACACATGGCGGAGTGACACAACCCAGAACTTCTAAGGTAT

76321ATGTCACTGGAAAGAAAGCTGTTCAAATGGCTGATCCAATATCATGCGGTGATACTGTGG

76381CTCAGGCATCATCTAAAGTATTCATTAAATAGGATTTAAAATGGCAAATACCCCTGTAAA

76441TTATCAATTAACAAGAACAGCAAATGCTATTCCCGAGATATTCGTCGGGGGTACGTTTGC

76501TGAAATAAAACAAAACCTCATTGAATGGCTTAATGGCCAAAATGAATTTTTGGATTATGA

76561TTTTGAAGGCTCAAGATTAAACGTTCTGTGTGACCTTTTGGCTTATAATACATTGTACAT

76621TCAGCAGTTTGGTAATGCTGCTGTGTATGAAAGCTTTATGCGTACTGCTAACTTACGAAG

76681TTCAGTTGTTCAAGCTGCACAAGATAACGGATATTTACCTACTTCAAAATCCGCTGCGCA

76741GACCGAAATTATGTTAACATGCACCGACGCATTGAATAGGAATTACATTACAATTCCTCG

76801CGGAACTCGCTTTTTAGCATATGCAAAAGATACTTCTGTTAATCCATATAACTTTGTATC

76861TACCGAAGATGTTATTGCTATTCGTGATAAAAATAACCAATATTTTCCACGATTAAAATT

76921GGCCCAAGGACGTATAGTAAGAACTGAAATCATTTATGATAAATTAACACCTATTATCAT

76981TTATGATAAAAATATTGATAGAAACCAGGTTAAATTATATGTTGATGGAGCGGAATGGAT

77041TAACTGGACGAGAAAGTCAATGGTTCATGCTGGTTCTACATCAACAATTTACTACATGCG

77101TGAAACTATTGATGGAAATACTGAATTTTATTTTGGTGAAGGTGAGATTTCCGTTAATGC

77161GGCAGAAGGAGCATTGACCGCTAATTATATTGGAGGTCTTAAACCTACCCAGAACTCTAC

77221GATTGTCATTGAGTACATCAGTACTAACGGTGCAGATGCGAACGGTGCAGTCGGATTTTC

77281ATATGCAGATACATTAACAAATATAACTGTCATCAATATTAATGAAAATCCAAACGACGA

77341CCCAGATTTTGTCGGAGCAGATGGCGGCGGTGATCCAGAAGATATTGAGCGTATTCGCGA

77401ATTGGGTACTATTAAACGCGAAACTCAGCAACGATGCGTAACTGCGACTGATTATGATAC

77461ATTCGTTTCAGAGAGATTTGGTTCTATTATTCAAGCTGTTCAGACTTTCACTGATTCTAC

77521TAAACCTGGGTATGCATTTATTGCTGCTAAACCTAAATCAGGATTGTATTTAACTACTGT

77581ACAGCGTGAAGATATTAAAAATTATCTCAAAGACTATAATTTAGCTCCTATTACGCCATC

77641AATTATTTCTCCTAATTATCTTTTTATTAAGACTAATTTAAAAGTCACATACGCTTTAAA

77701TAAGCTGCAAGAATCCGAACAGTGGCTCGAAGGTCAAATAATTGATAAAATTGATCGCTA

77761TTATACCGAAGATGTAGAAATTTTTAACTCGTCTTTTGCCAAGTCTAAGATGTTGACATA

77821TGTAGATGATGCAGATCATTCTATCATTGGTTCATCAGCGACAATTCAAATTGTTCGTGA

77881AGTACAAAACTTCTATAAAACGCCTGAAGCAGGTATTAAATATAATAATCAAATAAAAGA

77941CCGTTCTATGGAATCTAATACGTTTTCATTTAATTCTGGACGAAAAGTTGTAAATCCTGA

78001TACTGGTTTAGAAGAAGATGTATTATATGACGTTCGTATAGTATCAACAGACCGAGATTC

78061TAAAGGAATTGGTAAAGTTATTATTGGTCCATTTGCTTCTGGCGATGTTACAGAAAATGA

78121AAACATTCAGCCGTATACAGGCAACGATTTTAACAAATTAGCAAATTCTGATGGACGCGA

78181CAAATACTATGTTATCGGTGAAATAAATTATCCAGCTGACATGATTTATTGGAATATCGC

78241TAAAATTAATTTAACATCTGAAAAATTTGAAGTCCAGACTATTGAATTATATTCTGACCC

78301AACCGATGATGTTATCTTTACTCGCGATGGTTCACTGATTGTATTTGAAAATGACTTACG

78361TCCACAATACTTAACTATCGATTTGGAGCCTATATCACAATGACAGTAAAAGCACCTTCA

78421GTCACTAGTCTCAGAATTTCCAAGTTATCCGCTAATCAGGTGCAAGTACGCTGGGATGAC

78481GTTGGTGCTAATTTCTACTACTTTGTAGAAATCGCTGAGACAAAATCAAACTCGGGAGAA

78541AATCTCCCGAGTAATCAATATCGTTGGATTAATTTAGGATATACAGCAAATAATAGTTTC

78601TTTTTTGATGATGCTGATCCATTGACATCATACATTATCAGAGTAGCTACAGCTGCGCAA

78661GATTTTGAGCAGTCTGATTGGATTTATACAGAAGAGTTTGAAACTTTTGCTACAAATGCT

78721TATACATTTCAAAACATGATTGAAATGCAATTAGCTAATAAATTCATTCAGGAAAAATTT

78781ACTCTTAATAATTCTGATTATGTTAATTTTAATAATGACACTATAATGGCTGCATTGATG

78841AATGAATCATTTCAATTCAGCCCATCGTATGTTGATGTCTCATCAATCAGTAATTTTATT

78901ATTGGTGAAAACGAGTATCATGAAATACAAGGTTCTATTCAGCAAGTATGTAAGGATATT

78961AACCGAGTTTATTTGATGGAATCAGAAGGGATTCTATATCTTTTTGAGCGTTATCAACCC

79021GTAGTTAAAGTATCAAATGATAAAGGACAAACTTGGAAAGCTGTAAAGCTCTTCAATGAC

79081CGTGTAGGATATCCTTTATCTAAGACTGTATATTACCAATCTGCAAACACAACATACGTT

79141CTAGGATACGACAAGATTTTCTATGGTCGCAAATCTACTGATGTTAGATGGTCAGCAGAT

79201GATGTCAGATTCAGTTCGCAGGATATAACATTTGCTAAATTAGGAGACCAACTTCATTTA

79261GGATTCGATGTTGAAATCTTCGCTACTTATGCGACTTTACCAGCGAATGTATATCGTATA

79321GCTGAAGCCATTACTTGCACGGATGATTATATCTACGTTGTTGCCAGAGATAAAGTTAGA

79381TATATAAAAACGAGTAATGCACCTATAGATTCTGACCCGTTATCACCAACATACTCGGAA

79441AGACTTTTTGAACCTGATACAATGACTATAACTGGAAATCCTAAAGCTGTATGCTATAAA

79501ATGGATTCTATCGGTGATAAAGTTTTTGCTCTTATTATTGGTGAAGTTGAAACATTAAAT

79561GCCAATCCGAGAACTTCAAAAATAATTGATTCTGCTGATAAAGGAATATATGTTTTAAAT

79621CATGACGAAAAAACATGGAAAAGAGTTTTCGGTAATACCGAAGAAGAAAGAAGACGTATT

79681CAACCTGGATACGCAAACATGTCAACTGACGGTAAATTAGTTTCTCTGTCTTCGAGTAAT

79741TTTAAATTTTTAAGTGATAATGTTGTTAATGACCCTGAAACTGTAGCAAAATATCAGTTA

79801ATCGGTGCCGTTAAATATGAATTTCCTCGTGAATGGTTAGCTGATAAGCATTATCATATG

79861ATGGCATTTATAGCGGATGAAAAATCTGATTGGGAGACTTTTACTCCTCAACCAATGAAA

79921TACTACGCAGAACCGTTCTTTAATTGGTCTAAAAAATCTAACACACGCTGTTGGATAAAC

79981AACTCTAATAGAGCTGTAGTAGTTTATGCTGATTTAAAATACACTAAAGTTATAGAAAAT

80041ATTCCGGAAACATCACCAGATAGATTAGTTCATGAATACTGGGATGATGGTGATTGTACG

80101ATAGTAATGCCAAATGTTAAATTCACGGGATTTAAAAAATATGCGTCTGGGATGCTTTTT

80161TATAAATCTTCTGGTGAAATAATTTCTTACTATGATTTTAACTATCGTGTGAGAGATACA

80221GTAGAAATTATTTGGAAGCCGACTGGAGTATTTTTAAAAGCATTTTTACAAAACCAAGAG

80281CATGAGACTCCTTGGTCACCAGAAGAAGAGCATGGATTAGCTGATCCTGATTTAAGACCA

80341TTAATTGGCACAATGATGCCTGATTCTTATTTGTTGCAGGATTCGAATTTTGAGGCATTT

80401TGCGAAGCATATATTCAGTATCTTTCTGATGGATATGGAACTCAGTATAACAATTTACGA

80461AATTTAATTCGTAACCAATATCCACGAGAAGAGCATGCGTGGGAATATTTGTGGTCAGAG

80521ATATATAAAAGAAACATTTATTTAAATGCTGATAAACGTGATGCTGTTGCGAGATTCTTT

80581GAATCACGTAGCTATGATTTTTATTCTACTAAAGGAATTGAAGCATCATACAAGTTTCTT

80641TTTAAAGTTCTTTATAATGAAGAAGTTGAAATTGAAATTGAATCTGGGGCCGGTACTGAA

80701TATGACATAATCGTTCAATCTGATTCTTTGACTGAAGATTTAGTAGGACAAACGATTTAT

80761ACGGCAACAGGAAGATGTAATGTTACTTATATAGAAAGAAGTTATTCTAATGGTAAATTG

80821CAATGGACCGTAACTATTCATAATCTTTTGGGAAGATTAATTGCTGGTCAAGAAGTTAAA

80881GCAGAAAGACTCCCTAGTTTTGAAGGCGAAATTATTCGTGGAGTTAAAGGAAAGGATTTG

80941CTTCAAAACAATATAGACTATATTAATAGAAGTAGATCATACTATGTAATGAAAATTAAA

81001TCCAATTTACCTTCTTCCCGCTGGAAATCTGACGTTATTCGTTTTGTTCATCCAGTAGGA

81061TTTGGATTTATAGCAATTACCCTTTTAACGATGTTTATTAATGTTGGTTTAACTCTTAAA

81121CACACAGAGACAATAATTAATAAATATAAAAACTATAAATGGGATTCTGGATTGCCTACT

81181GAATATGCTGATAGAGTAGCTAAATTAACTCCAACCGGTGAAATTGAGCATGATTCAGTA

81241ACAGGCGAAGCAATTTATGAGCCTGGCCCAATGGCTGGTGTAAAATATCCTCTTCCTGAT

81301GACTATAATGCTGAAAATAGTAATTCAATATTTCAAGGTCAATTGCCTTCTGAACGACGC

81361AAGTTAATGAGTCCTCTATTTGACGCATCTGGAACAACATTTGCACAGTTTAGGGATTTA

81421GTTAATAAACGTCTAAAAGATAATATAGGAAATCCAAGAGACCCTGAAAATCCAACACAG

81481GTTAAAATAGATGAATGATTCAAGTGTTATCTATCGTGCGATAGTTACTTCAAAATTTAG

81541AACAGAAAAAATGTTGAATTTTTATAATTCAATTGGGAGTGGTCCGGATAAAAACACTAT

81601CTTTATCACATTTGGAAGATCAGAACCGTGGTCATCAAATGAAAATGAGGTGGGCTTTGC

81661TCCACCTTATCCAACTGATTCTGTATTGGGTGTAACTGACATGTGGACTCATATGATGGG

81721AACCGTAAAGGTTCTTCCATCTATGCTTGACGCAGTTATTCCTCGTCGAGATTGGGGAGA

81781TACTAGATATCCGGATCCATACACATTTAGAATTAACGATATTGTAGTGTGTAACTCAGC

81841TCCTTACAACGCTACTGAATCAGGCGCAGGCTGGTTAGTATATCGTTGTTTAGATGTTCC

81901TGATACTGGAATGTGTTCAATTGCATCTTTAACTGATAAGGATGAATGCCTTAAATTAGG

81961CGGAAAATGGACACCTTCTGTTAGGTCAATGACTCCTCCTGAAGGTCGAGGAGATGCTGA

82021AGGAACAATTGAACCTGGGGACGGGTATGTATGGGAATATCTTTTTGAGATTCCACCCGA

82081TGTATCTATAAATAGATGCACGAATGAATATATTGTGGTTCCTTGGCCTGAGGAATTAAA

82141AGAAGACCCGACTAGATGGGGATATGAAGATAATCTCACTTGGCAACAAGATGATTTTGG

82201ATTAATTTACCGGGTTAAGGCAAATACTATCCGTTTTAAAGCATATTTAGATTCAGTTTA

82261TTTTCCTGATGCTGCATTGCCAGGAAATAAAGGATTTAGACAAATATCAATAATCACGAA

82321TCCTCTTGAAGCTAAAGCTCATCCAAATGACCCAAACGTTAAAGCTGAAAAGGATTATTA

82381TGACCCAGAAGATTTAATGAGGCATTCGGGCGAAATGATTTATATGGAAAATAGGCCACC

82441TATTATTATGGCAATGGATCAAACAGAAGAAATCAATATTCTGTTTACATTTTAAATTAA

82501GGGAGCCCATGGGCTCCCTTTTTCTTTATAAATACTATAAACTCATAAGGAAACCGCTAT

82561GTTCATTCAAGAACCAAAGAAATTGATTGATACCGGCGAAATTGGTAACGCTTCTACTGG

82621TGATATCTTATTCGACGGTGGTAATAAAATTAATAGTGATTTTAACGCAATTTATAATGC

82681GTTTGGCGATCAGCGTAAAATGGCAGTAGCAAATGGCACTGGAGCAGATGGTCAAATTAT

82741CCATGCTACTGGGTATTATCAAAAACACTCTATTACAGAGTACGCAACTCCAGTGAAAGT

82801TGGCACTAGACATGATATTGATACCTCTACTGTAGGTGTTAAAGTTATTATTGAAAGAGG

82861CGAACTCGGCGATTGTGTTGAATTCATTAACTCTAATGGATCAATATCAGTTACTAATCC

82921TTTAACAATTCAAGCTATTGATTCAATTAAAGGTGTTTCAGGTAATTTAGTAGTAACTAG

82981CCCATATAGTAAAGTAACTTTACGCTGTATTTCATCTGATAATTCTACATCGGTTTGGAA

83041TTATTCTATTGAAAGTATGTTTGGACAAAAGGAATCACCAGCTGAAGGTACATGGAATAT

83101TTCTACATCCGGATCAGTTGATATTCCACTATTTCATCGCACTGAATACAATATGGCTAA

83161ATTGTTAGTTACGTGCCAATCGGTAGATGGAAGAAAAATTAAAACGGCAGAAATAAATAT

83221TCTTGTAGATGCTGTTAATTCAGAAGTCATTTCTTCTGAATATGCTGTCATGCGGGTTGG

83281AAATGAAACAGAAGAAGACGAAATCGCTAATATTGCATTTAGTATTAAAGAAAATTATGT

83341AACGGCAACTATAAGTTCTTCAACTGTCGGTATGAGAGCAGCAGTTAAAGTTATCGCTAC

83401GCAGAAAATCGGGGTGGCTCAATAATGAAACAAAATATTAATATCGGTAATGTTGTAGAT

83461GATGGTACCGGTGACTACCTGCGTAAAGGTGGTATAAAAATAAATGAAAACTTTGATGAG

83521CTTTATTACGAGCTTGGTGATGGAGATGTTCCATATTCAGCCGGTGCCTGGAAAACTTAT

83581AATGCTTCATCAGGACAAACATTAACAGCAGAATGGGGAAAATCATATGCTATTAACACT

83641TCATCTGGAAGAGTAACTCTACAACTTCCTAAAGGAACCGTTAATGATTATAATAAAGTA

83701ATTAAAGCTAGGGATGTATTTGCAACATGGAATGTTAATCCAGTTACTCTAGTAGCCGCT

83761TCTGGTGATACTATTAAAGGATCATCGTCATCAGTTGAAATTAATGTTCAATTTAGCGAT

83821TTAGAGTTAGTTTATTGTGCTCCAGGACGTTGGGAATATGTCAAAAACAAACAAATTGAC

83881AAAATTATTAGTTCAGATATTAGTAATGTAGCACGTAAAGAATTTTTAGTCGAAGTTCAA

83941GGACAAACAGACTTTTTAGATGTTTTCCGCGGAACTAGCTATAATGTTAATAACATCCGA

84001GTAAAACACCGTGGTAATGAATTATATTACGGCGATGTATTTAGTGAAAACAGTGACTTT

84061GGCTCTCCCGGAGAAAATGAAGGAGAATTGATTCCTCTTGATGGATTTAATATTAGGTTA

84121AGACAGCCTTGTAATATTGGTGACACTGTTCAAATTGAAACATTTATGGATGGTGTATCA

84181CAATGGAGAAGTTCATATACAAGACGCCAAATAAAAGTACTGGATTCAAAACTAACGTCA

84241AAAACGTCTTTAGAAGGAAGTATTTACGTTACTGATTTATCAACAATGAAATCAATTCCA

84301TTTTCTGCATTTGGATTAATTCCTGGAGAACCTATTAATCCTAATTCTCTTGAAGTTCGT

84361TTTAATGGAATTTTACAACAACAAGCTGGAACAGCAGGATATCCTCTGTTTTTGTGTGAA

84421GGTGCTAACTCAGATACGCAAGCAGGATGTATATCTCTTGGTGGTGAATGGAAAGAATCG

84481AACACAGATTATTCTATAGAATATGAAGATGGAAAACCTGTCAGTCTTTTATTTGATAGA

84541AAATTTGAATCAGGTGATATAATCGTTATAACATGGTTTAATAATGATTTAGGAACTCTT

84601TTAGAAAAAGATGACATTATTGAATTAACTGATGACCGTTATGTTAGTAAAGGATCATCT

84661ACTGAAGTTACTGGGGATGTAGCCTTAACAGATTTTGATAAAATCGGTTGGCCAAATGTT

84721GAAAAAGTTGATTCTTATACTAGAACGTATAATTCAATATCATCTATTTTCGACAGCATT

84781TATCCTGTTGGTTCAATTTATGAAAATGCTATAAACCCAAATAATCCGGTGACTTATATG

84841GGATTTGGTTCATGGAAATTATTTGGTAAAGGACAAGTTTTAGTAGGATGGAATGATGAT

84901GTTACGGATCCAAACTTTGCTTTAAACAATAATGATTTAGATTCTAGCGGAAATCCTTCA

84961CATACTGCCGGTGGAACAGTTGGTACAACATCAGTAACGCTTGAAAACGCAAATCTTCCT

85021GCGACTAAAACTGATGAAAGAGTTTTAATTGAAGACGAAAATGGATCAGTTATTATTGGA

85081GGATGTCAATATGACCCAGACGAAACTGGTCCTATATATACAAAATATCGTGAAGACTAC

85141GCAACAACAAACTCTTCACATACTCCTCCTACTAATATTAGTAATATTCAACCGTCTATT

85201ACTGTATACCGTTGGATAAGGATTGCATAATGAGTTTACTTAATAATAAAGCGGGAGTTA

85261TTTCCCGCTTAGCAGATTTTCTTGGTTTTAGAACTAAGAAAAATGATATTTCGGTTATGA

85321ATAATCAGCCGGTAGGCGCTGTAACAATTTCACAAATAGCAAAAGGTTTTTATGACTCAA

85381ATGTAGAATCTGCTATCAATGATGTTAGAAATATGGCAGAGCAACAGGTTGGAGCTGTAT

85441TAATTAATATAAGCGGCGTATCTCCTACTGGCGTGCAACAAACCGACTATTGGTCATTTG

85501AAGGAACTGTTACTGATACTTCAGCCAAACCAGGTGACCCAGTTATTGTTAACATGTTTG

85561GTATTCCAGTTAAAGCTACTAATGGAATGACTTCAATTGAATTTACTAGTGCTGTTAGAA

85621CAGCTCTACAGGAAATGGTTGTAAAATTTATTGCAATTGATTCATTTGAAGACCATCCTA

85681CCATAGGGAATAAAATACAAGTTAAGTATTTAGATAACCAAGAACATATCTTAGAACAAT

85741ACTCTGATAAAGGAATTACTTTCAAACAAGAAATAATTTCTCCTTCTAAACCCGGGTATG

85801GAACTTGGCAATTATTAGGTGCGCAAACTGTTACGCTAGATAGTCACACACAACCTACAG

85861TATTTTATTATTTTGAGAGAATAGCATGAGTAACAATACATATCAGCACGTATCAAATGA

85921ATCAAAATATGTTAAATTCGATCCAGTAGGATCGAATTTTCCTGGCACTGTTACGACAGT

85981ACAGTCTGCATTATCAAAAATAAGTAATATCGGCGTAAATGGTATTCCTGATGCAACTAT

86041GGAAGTTAAAGGAATAGCAATGATTGCATCAGAGCAAGAGGTTTTAGATGGAACAAATAA

86101TTCTAAAATCGTTACACCAGCTACATTAGCAACAAGATTATTGTATCCAAATGCTACTGA

86161AACTAAATATGGTTTAACGCGCTATTCAACAAATGAAGAAACATTAGAAGGCTCAGATAA

86221TAATTCATCAATAACGCCGCAAAAACTGAAATACCATACTGATGATGTGTTCCGAAATAG

86281ATATTCATCTGAGTCATCAAACGGGGTTATTAAAATATCATCTACGCCTGCGGCTTTGGC

86341TGGTGTTGATGATACTACGGCGATGACTCCGCTAAAAACCCAAAAACTCGCAATAAAATT

86401AATTTCACAAATAGCTCCTTCCGAAGATACTGCATCAGAATCTGTGAGAGGTGTGGTTCA

86461ATTATCAACTGTTGCGCAAACTCGTCAAGGAACTCTCCGCGAAGGATATGCAATTTCTCC

86521GTATACCTTTATGAATTCTGTTGCAACACAAGAATATAAGGGTGTTATACGTTTAGGAAC

86581ACAATCGGAAATTAACAGTAATTTAGGAGATGTTGCAGTAACAGGTGAAACACTAAATGG

86641TCGAGGAGCTACTGGTTCTATGCGTGGAGTAGTTAAATTAACGACGCAAGCCGGTATTGC

86701CCCTGAAGGTGATAGTTCTGGAGCATTAGCGTGGAACGCAGATGTAATTAATACGCGTGG

86761TGGACAAACTATTAATGGTTCTTTAAATTTAGACCATCTTACAGCAAATGGAATTTGGTC

86821ACGCGGTGGAATGTGGAAAAATGGCGATCAACCTGTTGCCACTGAAAGATATGCGTCCGA

86881ACGAGTTCCGGTTGGAACTATTATGATGTTTGCGGGTGATTCAGCACCTCCAGGTTGGAT

86941TATGTGTCATGGTGGAACCGTGTCAGGTGACCAATTTCCTGATTATAGAAACGTAGTTGG

87001AACAAGATTTGGTGGCGATTGGAATAATCCTGGCGTTCCTGATATGCGGGGTCTTTTCGT

87061CAGAGGAGCTGGCACAGGCGGTCATATTTTAAATCAACGAGGACAAGATGGTTATGGAAA

87121GGATAGACTTGGCGTAGGATGTGACGGAATGCATGTTGGTGGTGTTCAAGCACAACAAAT

87181GTCATACCATAAACACGCCGGTGGTTGGGGGGAATATAACAGAAGTGAAGGCCCATTTGG

87241CGCGTCTGTTTATCAAGGATATCTTGGAACTAGAAAATATGCCGACTGGGATAATGCTTC

87301ATACTTCACTAATGATGGATTTGAATTAGGCGGACCGAGAGATGCCCTTGGTACACTTAA

87361TCGTGAAGGATTAATTGGTTATGAAACTAGACCATGGAATATATCATTAAACTATATTAT

87421TAAAGTTCATTACTAAGGATTACAAATGATTGAATTAAAAGATTTACCTTTTGTTGATAG

87481CGTTCCTGATGAAGGTCAGGAACGTATTTCATGGATTAAAAATGGTGAAGAAATACTAGG

87541AGCTAGCACTAAGTATGGAAACGATGGATCAATGAATAGACCAATTGTTTCCGTGTTTAA

87601AAATGTTGAGGTTCTTGACGAAAATATAGGAATTCTTAAAACTGCCATTGAAACATCGCA

87661GAAAGATATTAAAACAATTCAAGGAGTTTTAGATGTATCAGGAGATATAGAAGCACTATC

87721GCAGATCAGTGTTAATAAAAATGACATATCAAATTTAAAGACACTTACTAATGAACACAC

87781TGATATATTAACTGGAACTAATAATACAGTTGACAAAATTATTGCTGATATAGGTCCATT

87841TAATGATGAAGAAAACTCTGTTTATAGAACAATTAGAAATGATTTATTGTGGATTAAACA

87901AGAACTAGGGCAATATTCTGGCCAAGACATTAACGGTCTTCCTGTTGTTGGTAATGCTAG

87961CACAGGAATGAAACATAGAATAATAACAAATAGTACATTATTATCTTCACAAGGCATTCG

88021TTTAAGTGAATTAGAAAATAAATTTACTGAATCTGACGTAGGATCATTGACGGTTGAAGT

88081TGGTAAATTACGCGATGAACTTGGTAATAAACCAGTAGATTTTGGACCAAATATTTATAA

88141TAGATTAAACACTATAGATGATAAACAAACTTTAATAAATTCTGATATAGCAGAAATTAA

88201ATCTTCTATTGGATACCCAGAAAATGTTTCTATAATAACAGAAATTAATAATAACAAAAG

88261TAGCATTGAATCTATTAATAATGAATTAAATCAGAGTGAAGGTGTTAAACAGCGCTTAAC

88321GGCTATTGAAACTTCTATAGGTTCAGATGATATTCCTTCTAGTATTAAAGGAAAAATTAA

88381AAATCACACAACTTCTATTGAGTCTTTAAACGGAATTGTTGGTGAAAACACTTCGTCTGG

88441TTTAAGAGCGAATGTTTCGTGGTTAAATCAAATTGTTGGAACTGACTCCAGCGGAGGACA

88501ACCGTCTCCTTCTGGATCTCTATTAAATAAAGTTTCAGTGCTTGAAGGAGAAGTTTCAGT

88561TTTAAACAATAATGTTCAAAATATACAAGTTGAAATAGGAAATAATAGAACAGGAATTAA

88621AGGTCAAGTTATTGAACTTACTTCACTTATAAATGGAAATAATCCTGACGGATCAACTGT

88681TGAAGAACGAGGATTAACTAATTCTATAAAAACGAATGAAACTAACATTGCGGCAGTCAC

88741GCATGAAGTAAATACAGCTAAAGACAATATATCCTCTTTACAGAGCAGCGTTCAAGCTCT

88801ACAAGAAGCGGGTTATATTCCTGAAGCTCCAAAAGATGGCCAAGCTTACGTTCGTAAAGA

88861CGGCGAATGGGTACTACTTTCTACCTTTTTATCACCAGCATAACATGGGGCCGCAAGGCC

88921CCAAAGGATTTTAAATGTCAGGATATAATTCTCAGAATCCAAAGGAACTCAAAGATGTCA

88981TTCTAAGACGTTTAGGGGCTCCAATTATTAATGTTGAGTTAACACCCGATCAAATTTATG

89041ATTGTATCCAGCGTGCCCTAGAATTATACGGTGAATACCATTTTGATGGACTTAACAAAG

89101GTTTTCATGTATTTTATGTAGGAGATGACGAAGAAAAATATAAAACTGGAGTCTTCGATT

89161TAAGAGGTTCAAATGTATTCGCAGTAACTCGCATTTTGCGAACAAATATTGGATCAATAA

89221CGTCAATGGACGGAAACGCTACATATCCATGGTTTACTGATTTTCTTTTAGGAATGGCTG

89281GTATTAATGGCGGTATGGGGACTTCTTGTAATAGATTTTATGGACCAAATGCCTTTGGAG

89341CTGATTTAGGATATTTTACCCAGCTTACCAGTTACATGGGAATGATGCAAGATATGCTCT

89401CTCCTATTCCAGACTTTTGGTTTAATTCAGCAAATGAACAGCTCAAAGTCATGGGAAACT

89461TCCAAAAATATGATTTAATTATCGTAGAAAGCTGGACTAAATCATACATTGATACTAATA

89521AAATGGTTGGAAATACAGTAGGATATGGAACAGTCGGTCTACAAGATAGCTGGTCATTAT

89581CTGAACGATATAATAACCCAGACCACAATTTAGTTGGTCGTGTTGTTGGTCAAGACCCAA

89641ATGTTAAACAGGGTGCTTATAATAATCGTTGGGTGAAAGACTATGCTACTGCTTTAGCTA

89701AAGAATTGAATGGTCAAATTTTAGCACGCCACCAAGGTATGATGCTTCCTGGCGGTGTTA

89761CGATTGATGGACAACGCTTAATAGAAGAAGCTCGATTAGAAAAAGAAGCATTACGTGAAG

89821AATTATACTTGCTCGATCCTCCATTTGGAATTTTGGTAGGTTAATATGGCTACTTATGAT

89881AAAAATCTTTTTGCTAAATTGGAAAACCGCACTGGTTATTCTCAGACCAATGAAACTGAA

89941ATACTAAATCCTTATGTAAATTTCAATCATTATAAAAACAGTCAAATATTAGCTGATGTA

90001TTAGTAGCTGAAAGCATTCAAATGCGAGGTGTAGAATGCTATTATGTTCCAAGAGAGTAT

90061GTTTCCCCTGATTTGATATTTGGCGAAGACTTAAAAAATAAATTTACTAAAGCTTGGAAA

90121TTCGCTGCATATTTAAATTCATTTGAAGGATATGAAGGAGCTAAATCGTTCTTTAGTAAC

90181TTTGGTATGCAAGTACAGGATGAAGTTACTTTGTCCATTAATCCAAACTTGTTTAAACAC

90241CAAGTTAATGGAAAAGAACCGAAAGAAGGCGATTTGATATATTTTCCTATGGATAACAGC

90301TTATTTGAAATTAACTGGGTTGAACCATATGATCCATTTTATCAATTAGGTCAAAACGCT

90361ATTCGTAAAATTACAGCAGGTAAATTCATTTATTCTGGAGAAGAAATTAATCCAGTTCTA

90421CAGAAAAATGAAGGAATTAACATTCCAGAATTTAGTGAATTAGAACTAAATCCTGTTCGC

90481AATCTTAACGGTATTCATGATATTAATATTGATCAATATGCTGAAGTAGATCAAATTAAT

90541TCTGAAGCTAAAGAATATGTTGAACCTTATGTTGTTGTCAATAACAGAGGCAAATCTTTT

90601GAATCTAGCCCATTTGATAATGATTTCATGGATTAATAAATATTATAAACTAATTAAAGC

90661CCAGATTAGGAGAAATCATGTTTGGTTATTTTTATAATTCGTCTTTTAGACGATATGCTA

90721CCTTGATGGGCGATTTGTTTTCAAATATCCAAATCAAACGTCAGTTAGAATCTGGTGATA

90781AGTTTATACGTGTTCCTATTACGTATGCATCAAAGGAACACTTTATGATGAAATTGAATA

90841AATGGACATCGATAAATTCACAAGAAGATGTGGCCAAGGTTGAAACTATTCTACCTCGTA

90901TAAATTTGCATTTAGTTGATTTTAGCTATAACGCTCCATTTAAAACAAACATTTTAAATC

90961AGAATTTACTGCAAAAAGGTACAACTTCTGTAGTATCGCAGTATAATCCATCTCCTATTA

91021AAATGATTTATGAATTGAGTATCTTTACTCGTTACGAAGACGATATGTTTCAAATAGTTG

91081AACAGATTCTTCCATATTTTCAACCTCATTTTAATACAACTATGTACGAACAGTTTGGAA

91141ATGATATTCCATTTAAAAGGGATATTAAAATTGTACTGATGTCTGCTGCTATAGATGAAG

91201CTATAGATGGAGATAATTTATCTCGTCGTAGAATCGAATGGTCACTAACATTTGAAGTAA

91261ACGGATGGATGTATCCTCCAGTAGATGATGCAGAAGGATTAATTCGTACTACTTATACAG

91321ATTTTCACGCCAATACAAGAGATTTGCCTGATGGCGAAGGTGTTTTTGAATCTGTTGATA

91381GCGAAGTTGTTCCTCGAGATATTAACCCAGAGGACTGGGATGGAACAGTAAAACAAACTT

91441TCACTAGCAATGTAAATAGACCAACACCGCCAGAACCTCCTGGCCCAAGAACATAGAGGT

91501TATTATGGAAGGTCTTGATATAAACAAACTTTTAGATATTTCTGACCTCCCCGGAATCGA

91561CGGGGAGGAAATTAAAGTATATGAACCTCTACAATTAGTAGAAGTTAAAAGCAATCCTCA

91621AAACCGTACTCCTGACTTAGAAGATGATTATGGAGTAGTTCGTCGAAATATGCATTTTCA

91681ACAACAAATGCTAATGGACGCAGCCAAGATTTTTCTTGAGACGGCAAAGAATGCTGATTC

91741TCCTCGTCACATGGAAGTATTTGCAACTCTTATGGGGCAAATGACTACGACGAACAGAGA

91801AATACTGAAGCTTCATAAAGATATGAAAGATATTACATCTGAGCAGGTTGGCACTAAAGG

91861CGCTGTTCCTACAGGTCAAATGAATATTCAGAACGCGACAGTATTCATGGGTTCACCAAC

91921AGAATTAATGGACGAAATTGGTGATGCTTACGAGGCTCAAGAAGCTCGTGAGAAGGTGAT

91981AAATGGAACAACCGATTAATGCATTAAATGATTTCCATCCATTAAATGAAGCTGGAAAAA

92041TTTTAATAAAACACCCAAGCTTAGCAGAAAGAAAAGATGAAGATGGAATTCATTGGATAA

92101AATCACAGTGGGATGGAAAATGGTATCCTGAAAAATTCAGTGATTATCTTCGTTTACACA

92161AAATAGTAAAAATCCCAAATAATTCTGATAAGCCTGAATTATTTCAAACTTATAAAGATA

92221AGAATAATAAAAGATCTCGGTATATGGGTCTTCCTAACTTAAAACGAGCTAATATTAAAA

92281CACAATGGACTCGTGAAATGGTTGAGGAATGGAAAAAATGCCGAGATGATATTGTATATT

92341TTGCAGAAACATACTGCGCCATTACTCATATCGACTATGGTGTCATAAAGGTTCAATTAC

92401GTGACTATCAGCGTGATATGCTCAAAATAATGTCATCTAAACGTATGACTGTTTGTAATC

92461TATCACGTCAGCTTGGTAAAACAACTGTAGTAGCTATTTTTCTTGCACACTTTGTATGTT

92521TTAACAAGGATAAAGCTGTAGGTATTCTTGCGCATAAAGGCTCAATGTCCGCGGAAGTTT

92581TAGACCGTACTAAGCAAGCAATTGAACTGCTTCCTGACTTTTTGCAGCCTGGTATAGTTG

92641AATGGAATAAGGGTTCAATTGAACTAGATAATGGTTCCTCAATCGGAGCCTATGCTTCCT

92701CTCCAGACGCAGTTCGTGGTAACTCGTTCGCAATGATTTATATTGACGAATGCGCATTTA

92761TTCCAAACTTCCATGATTCCTGGCTTGCTATTCAACCAGTTATTTCATCTGGTCGTCGTT

92821CAAAAATTATTATTACTACGACTCCTAATGGATTAAATCATTTTTATGATATTTGGACTG

92881CTGCTGTTGAAGGTAAATCAGGATTTGAACCATACACTGCTATTTGGAATTCAGTTAAAG

92941AACGTCTTTATAATGATGAAGATATTTTTGACGATGGATGGCAATGGAGTATACAAACCA

93001TTAATGGTTCTACTTTAGCTCAATTTCGTCAAGAACACACCGCAGCATTTGAAGGGACTT

93061CCGGTACATTAATTTCAGGAATGAAATTAGCTGTTATGGATTTCATTGAAGTAACTCCAG

93121ATGATCACGGTTTTCATCGATTTAAAAGCCCTGAACCAGATAGAAAATATATTGCAACTC

93181TAGACTGCTCAGAAGGTCGTGGGCAAGACTATCATGCTTTACATATAATTGATGTTACCG

93241ATGATGTGTGGGAACAGGTTGGTGTTTTGCACTCAAACACTATTTCTCATTTAATTCTAC

93301CTGACATCGTTATGCGTTATTTAGTAGAATATAATGAATGCCCAGTTTATATTGAATTAA

93361ATAGTACTGGTGTGTCAGTTGCTAAATCACTTTATATGGATTTAGAATACGAAGGCGTTA

93421TTTGCGATTCATATACTGATTTAGGAATGAAGCAAACTAAACGAACGAAAGCAGTCGGAT

93481GCTCTACATTAAAAGACCTTATTGAAAAAGATAAGCTTATTATTCATCACCGTGCAACTA

93541TTCAAGAATTTAGAACGTTTAGTGAAAAAGGCGTGTCTTGGGCGGCTGAAGAAGGTTATC

93601ATGACGATTTAGTAATGTCTTTAGTAATTTTTGGATGGTTATCAACACAATCAAAATTTA

93661TTGATTATGCCGACAAAGATGACATGCGATTAGCATCTGAAGTGTTTTCAAAAGAGCTTC

93721AAGATATGGGGGATGAATACGCTCCAGTTATATTTGTTGATTCGGTTCATTCTGCTGAGT

93781ATGTTCCAGTATCTCATGGTATGTCAATGGTATAAATATATTAAAGCATATTAAAGAGGA

93841TTAAAAATGACTTTATTATCTCCGGGCATTGAGCTCAAAGAAACTACGGTTCAAAGCACC

93901GTGGTTAATAACTCTACTGGTACAGCAGCTTTGGCCGGTAAATTCCAGTGGGGTCCTGCT

93961TTTCAGATTAAACAGGTTACAAACGAAGTAGATTTAGTTAATACTTTTGGTCAACCTACA

94021GCTGAAACTGCTGACTATTTTATGTCTGCAATGAATTTCTTACAATATGGAAATGATTTG

94081CGAGTTGTGCGCGCTGTCGATAGAGATACCGCTAAAAACTCATCTCCGATTGCTGGTAAT

94141ATTGAATACACAATTTCTACCCCAGGTAGTAACTACGCGGTTGGAGATAAAATCACGGTC

94201AAATATGTTTCAGAAGATGTTGAAACAGAAGGTAAAATTACCGAAGTAGATGCTGATGGA

94261AAAATTAAGAAAATTAATATTCCTACTGCAAAAATTATTGCTAAAGCTAAAGAAGTTGGC

94321GAATACCCAACATTAGGTTCTAACTGGACTGCAGAAATTTCTTCATCTTCTTCCGGTTTA

94381GCTGCAGTAATAACTCTTGGAAAAATTATTACTGATTCTGGTATTTTATTAGCTGAAATT

94441GAAAATGCTGAAGCTGCTATGACAGCGGTTGACTTTCAAGCAAATCTCGAAAAATACGGA

94501ATTCCAGGAGTAGTAGCTCTTTATCCAGGCGAATTAGGCGATAAAATTGAAATTGAAATC

94561GTATCTAAAGCTGACTATGCAAAAGGAGCTTCTGCATTACTCCCAATTTATCCGGGCGGT

94621GGTACTCGCGCATCTACTGCTAAAGCAGTGTTTGGATATGGACCACAAACTGATTCACAG

94681TACGCTATTATAGTTCGTCGTAATGATGCTATTGTTCAAAGCGTTGTTCTTTCAACTAAG

94741CGTGGTGAAAAAGATATTTACGATAGTAACATCTATATCGATGACTTTTTCGCAAAAGGT

94801GGTTCAGAATATATTTTTGCAACTGCACAAAACTGGCCAGAAGGATTCTCTGGAATTTTA

94861ACTCTGTCTGGTGGATTATCATCAAATGCTGAAGTAACAGCAGGAGATTTGATGGAAGCT

94921TGGGACTTCTTTGCTGACCGCGAATCTGTTGACGTTCAGTTGTTTATTGCGGGTTCTTGT

94981GCCGGTGAATCTTTAGAAACAGCATCTACTGTCCAAAAACACGTCGTTTCAATTGGGGAT

95041GCTCGTCAAGATTGCTTAGTATTGTGCTCACCTCCGCGTGAAACTGTAGTTGGAATTCCT

95101GTAACTCGTGCAGTAGATAATTTAGTTAACTGGAGAACTGCAGCAGGTTCATACACTGAT

95161AATAACTTTAATATCAGTTCAACCTATGCAGCAATCGATGGCAACTACAAATATCAGTAT

95221GACAAATATAATGATGTGAATCGTTGGGTTCCATTAGCAGCTGATATTGCTGGTTTATGC

95281GCGAGAACCGATAACGTTTCTCAGACTTGGATGTCTCCAGCTGGTTATAATCGTGGCCAG

95341ATTCTTAACGTTATTAAACTTGCTATTGAAACTCGCCAGGCTCAGCGCGACCGTTTATAC

95401CAAGAAGCTATCAACCCGGTAACCGGTACAGGTGGTGATGGTTACGTATTGTATGGTGAT

95461AAAACAGCTACTTCTGTTCCTTCTCCGTTTGATCGTATTAACGTTCGTCGTCTGTTTAAT

95521ATGTTGAAAACGAATATCGGACGTAGTTCAAAATATCGTTTGTTCGAATTAAACAATGCG

95581TTTACTCGTTCATCATTCCGCACAGAAACTGCCCAGTACTTACAGGGAATTAAAGCTCTC

95641GGTGGAATTTATGAATATCGTGTAGTTTGCGATACAACAAATAACACTCCGTCAGTAATT

95701GATAGAAATGAGTTTGTTGCAACATTCTACATCCAACCGGCGCGCAGTATAAATTATATT

95761ACTTTGAATTTCGTCGCAACTGCTACTGGTGCAGATTTCGATGAGTTAACTGGTCTTGCA

95821GGTTAATTGATATGGGGCTTCGGCCCCTATTCTCGAGGAATCAAGTGATTATTTCTAAAT

95881TTATTAAAGTGAAAATTGTACCGTCAAATTTTAAACATTATCAATCAAAGGGTTATAAAT

95941TAACACCTCTTGGAGCGGTTAAACATGGATTTAGGTATTATGAAATTGATGTTAAAGTTG

96001AAGACATTAAAGGTAGTACCGCGTATGTAAAATGCCGTTGCGATAAATGTGGAATTGAAT

96061ATAACCAACGCATTGGAAGAAACACTGATATTTGTCGTGATTGCCGTCATAAAGATAAAA

96121TGCGCAATAATTCATATGGTTCCGCGAATAAAGGAAAAACAGTAGAATGTATGCATGGCG

96181TAAATCATCCAAGATGGAACCCAAATAAATCTGAAATGCGTGAATATATTTCATTGGTAT

96241ATAAAGAAACTCGTAAGCATAAGAAAATTTGGTCAAAATGGCCAAATGCTGATAAAATAG

96301GAGTGTGTGGTACAAAAGGTGCTTACCAACTTGATCATAAGGTATCTATCAAATACGGTT

96361TTGATAATTTAATTCCTGCTAAAATAATTGGCGGTATTAATAATTTAGAAATAATCACTT

96421GGGAATCTAACCGAGAAAAATCAAAAAGAAATAGTGTAGATCTTTGGGATTTACTAGAAT

96481GATTCTAAAGGCCTGTTTCGGCAGGCCATATAAATACACTATATCCTTAATTCTTTAATT

96541CTATATGCCCTAGGTTAAACATAGGGATATAAATACTACAGAGGCTAATATGTTTGTAGA

96601TGATGTAACACGAGCGTTTGAATCTGGTGATTTTGCTCGACCTAACTTATTCCAAGTAGA

96661AATTTCTTATCTTGGACAAAATTTTACATTCCAATGTAAAGCCACTGCTTTACCAGCTGG

96721TATTGTAGAAAAAATTCCAGTCGGATTTATGAACCGTAAAATTAACGTAGCAGGCGATCG

96781TACATTCGATGACTGGACTGTTACAGTAATGAACGATGAAGCTCATGATGCTCGCCAGAA

96841GTTTGTTGATTGGCAAAGCATTGCTGCTGGTCAAGGAAACGAAATTACTGGTGGAAAACC

96901TGCAGAGTATAAAAAGAGCGCTATTGTTCGTCAATATGCTCGTGATGCTAAAACAGTAAC

96961AAAAGAAATTGAAATTAAAGGTCTGTGGCCTACTAACGTGGGTGAACTTCAATTAGATTG

97021GGATTCAAACAATGAAATCCAAACCTTTGAAGTAACTCTTGCTCTCGATTATTGGGAATA

97081AAATGAATGGGGAGAAATCCCCATCCTGCTTAAAGCAGAGAAGTCCATTATAAATATAAC

97141TATAATTCCCATTTGGAGAATACAATGAAATTTAATGTATTAAGTTTGTTTGCTCCATGG

97201GCTAAAATGGACGAACGAAATTTTAAAGACCAAGAAAAAGAAGATCTTGTTTCCATTACA

97261GCCCCAAAGCTTGATGATGGAGCAAGAGAATTTGAAGTAAGCTCGAATGAAGCTGCTTCT

97321CCTTATAATGCTGCATTCCAAACAATTTTTGGTTCATATGAACCAGGAATGAAAACTACT

97381CGTGAGCTTATTGATACATATCGTAATCTCATGAATAACTATGAAGTAGATAATGCAGTT

97441TCAGAAATCGTTTCAGATGCTATCGTCTACGAAGATGATACTGAAGTCGTAGCGTTAAAT

97501TTGGATAAATCTAAATTTAGTCCAAAAATTAAAAATATGATGTTAGATGAATTTAGTGAT

97561GTATTAAATCATCTATCGTTTCAACGAAAAGGTTCTGATCATTTTAGACGTTGGTATGTT

97621GATTCAAGAATTTTCTTTCATAAAATCATTGATCCAAAACGTCCAAAAGAAGGCATAAAA

97681GAATTACGTAGATTAGACCCTCGCCAAGTTCAGTATGTTCGCGAAATTATAACAGAAACT

97741GAAGCTGGCACAAAAATAGTTAAAGGTTACAAAGAATATTTTATATATGATACTGCCCAT

97801GAGTCATATGCATGTGATGGTAGAATGTATGAAGCTGGCACAAAAATAAAAATTCCAAAA

97861GCTGCTGTTGTTTATGCCCATTCTGGATTAGTCGATTGCTGTGGCAAAAATATCATCGGG

97921TATTTACATCGTGCTGTTAAACCTGCTAACCAATTAAAATTATTAGAAGATGCTGTAGTC

97981ATTTATCGCATTACTCGTGCTCCTGACCGTCGTGTTTGGTATGTAGACACAGGTAATATG

98041CCTGCTCGTAAAGCTGCTGAGCACATGCAACATGTTATGAACACGATGAAAAACCGTGTA

98101GTATATGATGCATCAACAGGTAAAATAAAAAATCAACAACATAATATGTCTATGACCGAA

98161GACTATTGGTTGCAGCGCCGTGATGGTAAAGCTGTGACAGAAGTTGATACTCTTCCTGGT

98221GCTGATAATACTGGCAATATGGAAGATATTCGTTGGTTTAGACAAGCTCTTTACATGGCA

98281TTACGTGTTCCTCTTTCACGCATTCCACAAGACCAACAAGGCGGTGTGATGTTTGATTCT

98341GGAACTAGCATTACACGTGATGAATTAACGTTTGCTAAATTTATTCGTGAGTTACAGCAC

98401AAGTTTGAAGAAGTTTTCCTAGATCCACTTAAAACAAATCTTTTGCTTAAAGGTATAATC

98461ACAGAAGATGAGTGGAATGATGAAATAAATAATATTAAGATAGAATTTCATCGGGATAGC

98521TACTTTGCTGAGCTCAAAGAAGCAGAAATTTTGGAACGAAGAATTAATATGCTAACCATG

98581GCAGAACCATTTATTGGTAAATATATTTCTCACAGAACTGCTATGAAAGATATTTTGCAG

98641ATGACTGATGAAGAAATAGAACAAGAAGCCAAGCAAATTGAAGAAGAGTCTAAAGAGGCT

98701CGTTTCCAAGACCCCGACCAAGAACAAGAGGATTTTTAATGGAAGGTTTAATTGAAGCTA

98761TTAAATCAAACGACCTCGTAGCCGCTCGTAAATTATTTGCTGAAGCCATGGCTGCAAGAA

98821CGACTGATTTAATTAAAGAAGAAAAAATCGCTATCGCTCGTAATTTCTTAATCGAAGGTG

98881AAGAACCTGATGACGAGGATGATGACGAAGACGAAGATAGTGATGATAAAGACGACAAAA

98941AAGACAAAGACTCTGACGAAGACGAGGATGATGAATAATGCTTCTGATCCCTGAAACTCA

99001TGAATTAGTTCTCGAGAATGTCGAAGCACTTATTCCTGAAGCACAGGGTCGCTTTGACGA

99061ATTGTCTTCTGCTTTAAATAAAGACGATATAAATACAATTGTCGAGAATATGCTTGATGA

99121TGAAACTGATTTAGCGGTTGCATTAGCTTCTATTAATGAAAATATGCCGTTAAATGAATT

99181TATCGTTAAACATGTTTCTGCCCGTGGTGAAATTACTCGCACTAAAGATCGCAAAACCCG

99241CGAACGTAATGCATTTCAAACTACCGGGTTGTCTAAAGCAAAACGTAGACAAATTGCTCG

99301TAAAGCTACCAAAACGAAGATTGCCAATCCAGCAGGTCAATCTCGTGCTCAGCGTAAGCG

99361TAAAAAAGCTCTTAAACGCCGTAAAGCATTAGGATTAAGCTAATGAATGAACCCCAATTA

99421CTAATTGAAACTTGGGGTCAACCTGGCGAAATTATTGATGGCGTACCAATGCTTGAATCT

99481CATGATGGAAAAGACTTAGGTTTAAAACCGGGTTTATACATCGAAGGAATATTCATGCAA

99541GCGGAAGTCGTCAATAGAAATAAACGTCTTTATCCAAAACGTATATTAGAAAAAGCGGTA

99601AAAGACTATATTAATGAGCAAGTTTTAACTAAACAAGCTCTCGGAGAATTAAATCATCCT

99661CCACGCGCTAATGTTGACCCGATGCAAGCCGCTATCATTATAGAAGATATGTGGTGGAAA

99721GGAAATGACGTATACGGACGAGCTCGTGTTATTGAAGGTGACCACGGTCCTGGAGATAAA

99781TTAGCAGCTAATATTCGTGCCGGATGGATTCCAGGAGTTTCTTCTCGTGGATTAGGTTCA

99841TTGACTGACACAAATAAAGGTTATCGTATCGTAAACGAAGGATTCAAATTAACTGTAGGT

99901GTTGATGCAGTATGGGGTCCAAGTGCTCCAGATGCATGGGTAACTCCTAAGGAAATTACC

99961GAATCACAGACGGCGGAAGCCGATACAAGTGCCGATGACGCCTATATGGCTCTCGCAGAG

100021GCCATGAAAAAAGCGTTATAAATATTATTATCTAAACAACAGGACTACAAAATGCTTAAA

100081GAACAACTGATTGCCGAAGCGCAGAAAATTGATGCTTCCGTTGCTCTTGATAGTATTTTC

100141GAATCAGTTAATATTTCTCCGGAAGCAAAAGAAACTTTCGGCACTGTATTCGAAGCTACC

100201GTCAAGCAGCACGCCGTTAAATTAGCTGAATCTCATATCGCTAAAATTGCTGAAAAAGCA

100261GAAGAAGAAGTAGAAAAAAATAAAGAAGAAGCAGAAGAAAAAGCTGAGAAGAAAATCGCT

100321GAGCAAGCTTCTAAATTCCTTGACCATCTTGCAAAAGAATGGCTCACTGAAAATAAATTA

100381GCAGTAGATAAAGGTATCAAAGCCGAACTGTTTGAATCCATGCTTGGTGGATTGAAAGAG

100441CTCTTCGTTGAACACAACGTTGTTGTTCCAGAAGAATCAGTTGATGTTGTAGCTGAAATG

100501GAAGAAGAACTGCAAGAACATAAAGAAGAATCAGCTCGTCTGTTCGAAGAACTCAATAAG

100561CGTGACGCATATATCAATTATGTGCAGCGTGAAGTGGCATTGAGCGAAAGTACTAAAGAT

100621CTGACTGAGTCTCAAAAAGAAAAAGTCTCTGCTCTGGTCGAAGGTATGGATTATTCAGAT

100681GCATTCTCAAGTAAATTGAGTGCAATCGTAGAAATGGTGAAGAAATCTAATAAAGATGAA

100741AGCACTATTACTGAGAGTATAAATACTCCTGATACTGAAGCAGCCGGACTGAATTTCGTC

100801ACTGAAGCTGTAGAAGATAAATCTGCACAAGGTGCAGAAGATATTGTAAGTGTATATGCG

100861AAAGTCGCATCTCGTTTCTAATTTTAAAGGTTAACACAAATGACTATCAAAACTAAAGCT

100921GAACTTTTGAACAAATGGAAGCCATTACTGGAAGGTGAAGGTTTACCGGAAATTGCTAAT

100981AGCAAACAAGCGATTATCGCTAAAATCTTTGAAAACCAGGAAAAAGATTTCCAGACAGCT

101041CCGGAATATAAAGACGAAAAAATTGCTCAGGCATTCGGTTCTTTCTTAACAGAAGCTGAA

101101ATCGGTGGTGACCACGGTTACAATGCTACCAACATCGCCGCAGGTCAGACTTCTGGCGCA

101161GTAACTCAGATTGGCCCAGCTGTTATGGGTATGGTACGTCGTGCTATTCCTAACCTGATT

101221GCTTTCGATATTTGTGGTGTTCAGCCAATGAACAGCCCGACTGGCCAGGTATTCGCGCTG

101281CGCGCAGTATATGGTAAAGATCCAATCGCTTCCGGCGCTAAAGAAGCATTCCACCCAATG

101341TATGGTCCAGATGCAATGTTCTCTGGTCAGGGTGCTGCTAAGAAATTCGCTGCTTTGAAA

101401GCAAGTGATACTCTTGAAGTCGGAACTATTTACACTCACTTCTTCCAGGAAACTGGTACA

101461GTATATCTGCAAGCTACAGCAGCTAAACAAATTGATTCAGGCGCATCTGACGCAGACAAA

101521TTAGATGCTGAAATTAAGAAACAAATGGAAGCTGGTGTACTGGTAGAAATCGCTGAAGGT

101581ATGGCTACTTCTATCGCTGAACTTCAGGAAGGTTTCAACGGTTCTACCGATAACCCATGG

101641AATGAAATGGGCTTCCGTATCGATAAACAAGTTATCGAAGCTAAATCTCGTCAGCTGAAA

101701GCTGCTTACTCTATTGAATTAGCACAAGACCTTCGCGCTGTTCACGGTATGGATGCTGAT

101761GCTGAACTGTCTGGTATTCTGGCTACCGAAATTATGCTGGAAATCAACCGTGAAGTTGTT

101821GATTGGATTAACTACTCAGCTCAGGTTGGTAAATCTGGTATGACCCTGACTCCGGGTTCT

101881AAAGCTGGTGTATTTGACTTCCAGGACCCAATCGATATTCGTGGTGCTCGCTGGGCAGGT

101941GAATCCTTTAAAGCTCTGTTGTTCCAGATTGACAAAGAAGCAGTTGAAATTGCTCGTCAG

102001ACTGGTCGCGGCGAAGGTAACTTCATTATCGCTTCCCGTAACGTAGTTAACGTTCTGGCT

102061TCAGTTGATACCGGCATTTCTTATGCTGCACAAGGTCTGGCTACTGGCTTTAACACTGAT

102121ACTACCAAGTCAGTATTTGCTGGTGTTCTGGGTGGTAAATACCGTGTATATATCGACCAG

102181TATGCTAAACAGGATTATTTCACTGTAGGTTATAAAGGTCCGAACGAAATGGATGCTGGT

102241ATTTACTACGCTCCATATGTAGCTCTGACTCCGCTGCGTGGTTCCGATCCGAAGAACTTC

102301CAACCAGTAATGGGATTCAAAACTCGTTACGGTATCGGTATCAACCCATTTGCAGAATCC

102361GCTGCTCAGGCTCCGGCTTCTCGCATCCAGAGCGGTATGCCTTCTATTCTGAATAGCCTT

102421GGTAAAAACGCTTACTTCCGCCGTGTATATGTTAAAGGTATCTAATCTTTGACGATTTAA

102481GGGACCTTCGGGTCCCTTTAGTTGTTTTTATGGTATAAATAAATCATATAAACTGAAAGG

102541AAAGCGCAATGGCTAAAATCAACGAACTTCTGCGCGAATCAACCACAACGAATAGCAACT

102601CAATCGGTCGCCCAAATCTCGTTGCTTTGACTCGCGCTACCACTAAATTAATATATTCTG

102661ACATTGTAGCAACGCAAAGAACTAATCAACCTGTTGCTGCTTTTTATGGTATCAAATACC

102721TTAACCCAGACAATGAATTTACATTTAAAACTGGTGCTACTTATGCTGGCGAAGCTGGAT

102781ATGTAGACCGAGAACAAATCACAGAATTAACAGAAGAGTCTAAATTAACTCTCAATAAGG

102841GCGATTTATTCAAATATAATAATATCGTTTATAAAGTATTAGAAGATACACCATTTGCTG

102901ATATTGAAGAAAGCGATTTAGAATTAGCTCTTCAGATTGCAATTGTTCTTTTAAAGGTTC

102961GTCTATTTTCTGACGCAGCGTCAACAAGCAAATTTGAAAGCTCTGATAGTGAAATTGCGG

103021ATGCTAGATTCCAGATTAATAAATGGCAAACCGCAGTTAAATCTCGTAAACTTAAAACTG

103081GTATCACAGTTGAATTAGCACAAGATTTAGAAGCAAATGGATTCGATGCTCCTAATTTCT

103141TGGAAGATTTGCTTGCAACTGAAATGGCAGATGAAATCAATAAAGATATTCTGCAGTCTT

103201TGATTACAGTGTCAAAACGCTATAAAGTTACAGGAATTACTGATACTGGATTCATCGATT

103261TGAGTTATGCATCTGCACCTGAAGCTGGTCGTTCATTATACCGAATGGTATGTGAAATGG

103321TTTCGCATATCCAAAAAGAATCAACTTATACAGCAACGTTCTGTGTTGCTTCAGCTCGTG

103381CTGCTGCGATTCTTGCTGCATCAGGTTGGTTAAAACATAAACCAGAAGATGACAAATATC

103441TTTCACAAAATGCCTACGGGTTCTTAGCTAATGGTTTACCGCTTTATTGCGATACCAACA

103501GTCCATTAGATTATGTAATCGTTGGCGTAGTAGAAAATATTGGTGAAAAAGAAATTGTTG

103561GATCAATTTTCTATGCTCCGTATACAGAAGGTCTTGACTTAGATGACCCTGAACATGTAG

103621GTGCATTTAAAGTTATAGTTGATCCAGAAAGTTTGCAGCCATCTATCGGTTTATTAGTTA

103681GATATGCTTTATCTGCAAATCCTTACACTGTAGCAAAAGATGAAAAAGAAGCAAGAGTAA

103741TTGATGGTGGAGATATGGATAAAATGGCGGGTCGTTCAGATTTGTCTGTTTTATTAGGTG

103801TTAAGCTACCAAAAATTATCATTGATGAATAAAACAAAGGGACCGAAAGGTCCCTTTTTA

103861TTTAACTTACCAATTCAATCCAAGCCGGACGAAGTACATCTTGTACCATTTTAACTAATT

103921CCTTCTTAATCAAAGAAGGATTATCCGCTTGAGTTAGAGTAATACCTTCACGAGAAGTTT

103981CTTCCAAAATATCTTGAACAGTTAGCCCCATCACCTTTCCAAAATCTTTTGGACCAATTT

104041CACCAATTTTAGAAATAACGTTATTTACTCGGTTCAGTGTAACGTAACAAGCTAAAATTC

104101CAACCAATTTGTTATCAGCTTCTGACAGTTCAACTTTAGCTTTAATAGGCTTATCAGACT

104161TTTTCTTTTCACTAAATTTAGAGTTCTTGCATTTGATAGCTACGCGATTGCCATTAGGCA

104221TCCAAGAAGGATAACAAGGTTTCAATACATATCCTTCAGCGGTAAATACTTCGCCTTTTA

104281CTTCAGCATTCCAAACGCATTTATTTGCATCAACTAATCCAGCATGGTCTACCGTAAAAT

104341TATAATCCTGGACGACAGAATCTAAATCATTTGGCAATTTAATAAGTTCTTCAAATTTAC

104401CACGACCTAAAAGTGGAGCCATTTTAAATTTAAATGTATTACAAAATGATTCCATCATAT

104461AATCATCGACATAAGTCACATCACCACTTTCTGTAGTGACAATAATGTCAAATACATAAA

104521AATCTTTATCGCCATAATCGACATTCTTCTGAATGCCAGGTCCAGCGAATTCGCCAAAGA

104581CTTGATAAGATACAACCGCTGAGGTTTCCATAATATCTTGTACTGCTTTAATAGAATCTT

104641CGTAATTCTTTAGAATAATTTCATACCCAAAGAAATCTTCAGCAGGAAGAATCGGTCCAG

104701TACGTTTAGCGCAAGTTACTTTATCACGCTCAATAATCAATGAGAAATTTGTGCCGTGAA

104761TCTTTTCACGAGCTACCCACTCCCCACCAGTCAATCCCAAGCTATAAAGTTTTTCAATAA

104821ATTTAGAGTTGTAATGATTTTCAAGACTGCTATACTTTTTAAACATAATTAATCCTCAAA

104881ATGTAATTTCTAACCAATCACCATCACGCTGATCACTATTGACTTTAAAGCTGAATCCTT

104941CTTTTCTCAGCCAATCACCAATTTCTTCAGTAATCAATTTATCACGAGCAATACAATAAT

105001AACTAAAATGTGTTTTACCTTGTTCAGCTGCTTTATTAGCAAGTTCCGAAAAATCTTTAA

105061TAAAACACTCTAGCTTAAACTGTTTACTTTTAAGTGCTTTTTCGCGCAATTGATTAGCAA

105121AAGATTCATTTTCATAAAGATCATACTGTTCCATTTTTCACCTTTTTATTGATATGTCTT

105181TTTCTATAGACGACTTTTTCTCGAGTTTCAAATTTATTTTTAGCAGTCATAGCTCGAGCC

105241CATAATACAGCCACTTCTTTTGCCTGTAAGTTTAATTCACGAGCAATTTCAATGAATGAC

105301TTTCCAGACTCATGAAGAGTAAACACCACAACCTCAGTTCTCATAATCAATCTCATGTTA

105361TCGAGTTGGTGCCATTATATACATCATTTTCTGATTGTGTTTTGTGTGCTTTCAAAATGA

105421AGAAAGGGGCCGAAGCCCCTTATGATTATGGATAGGTATAGATGATACCAGTTTCTAAAG

105481CAGTTTTATGAATGATGTATCCATTACGCGATTCTTGAACATCAACTTCTGGATAGTCTT

105541TCATCATCTTCTGAAGAGTGTAACGATGTAGGTAATATTTACTATCTGGGTCGTCAGTTT

105601TCCAATCTTTACCTTCTTCGGTCATTTTTTGGATTTCATCCATAACCCACCAACCGCACC

105661AGATATAAGCTGAAGTACGATGTGGAAGAGGATGAACATAAGGTAGTTCACCTTCTGGTT

105721CTGGAGCAACTTTATCAGTTACCTGAACTTGACCTTCTGCTGTAATAGTTTTGCTATCAT

105781AATCAGTAGCAGTAACAATTGCAGTAACTTCAATAGTTTGACTTCCAATAGATGAGGTAT

105841CAACGGTATATACATTAGTTGACCCTTCTACAGGAGAAGAATCTTTCTTCCATGAGTATT

105901CGATTTGTGCTTCTTCTGGAGCATCAGTGACATTAGCAGTAAATGTAGCCGAAGCATCTT

105961GCTGAACGTTAATAGAAGGAGGAGTCAATGTAACCTGTGGATTCATTGTCTTCTTATTAA

106021CTGTTAATGATACTTCATTAGAAGTAACTGTTTTTGCGTCATAATCTGTTGCTGTTACTT

106081GGGCTACGCATTTAATTCTTTTTACTCCACTTGTAGTTGGAGTATAGTTAAATGTAGAGT

106141TAGTTTCTTCACCAATTTGAGAATCATCTACATACCATTGATATGTAGCAGACGCTCCAA

106201CGGGTTGAGAAACTAAGGCAGCAGTAAATTGAACTGGGGTTCCAATAACTCCTGCTGGAG

106261GACTATCAGGAGTTACTGCCAAGGTAGTTGTCTGCGTCTTTTTATTAACAGTCAGTGACA

106321CTTCATTAGAAGTAACGCTCAGTGCATCATAATCTGCCGCAGTTACTTGAGCTACACACT

106381TGATTTTTTTAACTCCGCTTGTGTCAGGAGTGTAATTGAATGTAGCGTCAGTTGCTTCGT

106441CTACAGGAGAACCGTCAACGTGCCATTGATACGTAACGTTTGCACCTGATGGCTGTGAAG

106501CTAAGGCAGCGGTAAATTCAATTGGAGTTCCAATCACTCCAGCATCAGGGCTACCAGGAG

106561TTACAGCTAAAGTAGTTGTCTGTGTCTTATTTTGAACTGTGATAGTTGTACTAATTTCTG

106621CTGTTTCAGGTTCAGATTCTGCAACTTGATTGGTTGCAACTACTTTAATAGTCTTTTGAC

106681CGGCAGGTCCTTTTAATACATAATCAAAAGTTGCTGATGATCCTTCTTGGGGAGCATCAT

106741CTACGCTCCAAGCATAGGTAATAGTTCCACCTCCAGTTTCACCACTGGGTGTAGCAGTAA

106801ACTGCTTGGTTTCATCAATAACCCCTGTTGGTGTTTTAGGAGTTATATCAACTGTAAAAG

106861TCATAAGTTATCCTTATTTTAATGTTACGAAAGAAGAATTGCGTGTTTCACGAATTAAAA

106921CTGATCCGTCACGGTTAATGTAATAAATTAAGCTAAATAAAGTTTGGTGTGCCGACGCGT

106981GTTCAAAACTAGTTGGGTGAGATTTCCAATCCGGAGTTTCAGCAATCCACTGGTAAATCC

107041ACCAAGGAACAGTACAGAATCCTGGATTTTTTCCAATTAACTGAAGATTCGGACTAAAGT

107101TTTCCGGAAGAGTAAATACAGACGGCTTTTCAGATTCAATAATCTCAGCTACAGCCTGTT

107161CAAATTTTTCTTCAACAAAAGGAGTATCTTCAATCAAAACATCGGTATTTTCAGGAATTT

107221TATCCGTTTCTACTACTTCAATTTTAATGTCAGATTTAACCGGAGAATCAATCAAAAGTG

107281CTGCTTCTGGATTGACTTCTTCATCGTCATATTTTAACCCCTCTGCAGCATCAGCAGCAT

107341CAATTAAGTCTTTAATAGATAACCCATCAGTCTCTGGCATAGGTTCACTAGCGAGCTTCT

107401GGAGGGCTTCTTCAATATCAACAACGATATTATCAAAAGATTTATTCTTTTTGACCTTTA

107461TACCAAACTGTTCAGCATATTCAGCTAATTTAGCTTTAGCTTCTTTGTTATCATCAAGAG

107521CCTTCAGCTCTGCAATATAATCTTTATCTATCATAATATTTCCTCAGTATAAATATAGAT

107581ATATTTATTACATGGTATTTAGACATGACTGACATTAAAGTACATTTTTATGATTTTAGT

107641CACGTGAGAATTGATTGTGAAGAAAGTACATTTCATGAACTCCGTGATTTCTTTAGCTTT

107701GAAGCTGATGGGTATCGTTTTAATCCGAAATACAAATATGGCCACTGGGATGGACGAATC

107761CGTCTTTTAGATTATAATCGTCTTCTTCCATTCGGCTTAGTCGGGCAAATTAAAAAGTTC

107821TGTGATAACTTTGGCTATAAAGCCTGGATTGACCCACAAATTAACGAAAAAGAAGAATTA

107881TCAAGAAAAGATTTTGATGAATGGCTTTCTAAATTAGAAATCTATTCAGGAAATAAAAGA

107941ATTGAACCGCACTGGTATCAAAAAGATGCAGTGTTCGAAGGATTAGTTAATCGTCGTAGA

108001ATTCTTAATCTTCCAACATCCGCTGGTAAATCGTTAATTCAAGCTCTGCTTGCTCGGTAT

108061TATTTGGAAAATTATGAAGGTAAAATTCTTATCATTGTTCCAACAACTGCTCTGACAACT

108121CAGATGGCTGATGACTTCGTCGACTATCGTCTATTCAGTCATGCAATGATAAAGAAAATT

108181GGTGGCGGAGCATCAAAAGATGATAAATATAAAAATGATGCACCAGTCGTTGTTGGTACA

108241TGGCAAACTGTAGTAAAACAACCGAAAGAATGGTTCTCACAGTTTGGAATGATGATGAAT

108301GATGAATGTCATCTTGCTACAGGAAAAAGTATTTCATCCATCATATCAGGTTTAAATAAC

108361TGCATGTTCAAATTCGGTTTGTCTGGTTCATTACGTGATGGCAAAGCCAATATCATGCAG

108421TATGTTGGAATGTTTGGTGAAATATTTAAGCCAGTAACGACTTCTAAATTAATGGAAGAT

108481GGACAAGTAACTGAGCTAAAAATTAATAGTATTTTTCTTCGCTATCCCGATGAGTTCACT

108541ACTAAATTAAAGGGAAAAACTTATCAAGAAGAAATAAAAATTATTACGGGGCTTAGTAAA

108601AGAAATAAATGGATCGCTAAATTAGCTATTAAGCTTGCGCAAAAAGATGAAAACGCTTTT

108661GTCATGTTTAAACATGTATCGCATGGTAAAGCTATTTTCGATTTAATTAAAAATGAATAC

108721GATAAAGTTTATTACGTATCAGGGGAAGTTGATACCGAAACCCGCAATATAATGAAAACC

108781TTAGCTGAAAATGGTAAAGGAATAATTATAGTAGCTAGTTATGGTGTATTTTCTACTGGT

108841ATTTCAGTTAAAAATCTGCATCATGTTGTTTTAGCGCACGGTGTTAAATCAAAAATTATT

108901GTATTACAAACAATCGGTCGTGTATTACGTAAGCATGGTTCTAAAACAATCGCAACAGTC

108961TGGGATCTCATAGATGACGCGGGCGTCAAGCCAAAATCTGCTAATACGAAAAAGAAATAT

109021GTTCATTTGAACTATCTTTTAAAACACGGCATTGATCGTATTCAGCGCTACGCAGATGAA

109081AAATTTAATTACGTAATGAAAACAGTTAATTTATAAGGGCTTCGGCCCTTTGGAGAAAAA

109141GATGTTACTAGAATTTAAACAATTTCTTTATGAAGCTTCTATTGATGAATTTATGGGTAA

109201AATTGCCTCTTGCCAAACATTAGAAGGTCTAGAAGAACTTGAAGCTTATTATAAGAAAAG

109261AGTCAAAGAAACTGAATTAAAAGATACTGATGACATCTCAGTCAGAGATGCTTTGGCAGG

109321AAAAAGAGCTGAATTAGAAGATTCAGACGATGAAGTAGAAGAAAGCTTTTAAATTAAAAA

109381AGGCCCAACCAAAAAGGAAGGGCCAAAACTATAGACTAAAGGTCACACTATAGCAAAAGT

109441TGTGTTTCATTTAATTGTTCTTCCGAACTTTCTGAAACTGGTAGTTCTTTAATGTAATTA

109501TAGCAAGGCCCAGGATGTACAGGACCTTTGTCTGTTTCAACAACCAATGCAGAATCGATT

109561GGAGTTTTACAGACAACACAAATCTTATCTGACATGATTGTCTCCTCTGAATTATATATC

109621TATTTATACAACTCTCATATGCATATCAATGCCCATATCTTTAGAATAAAAATATTCATC

109681AAGATATCCGGCAAATTTTCCTTTAATATAAAGGACATCTTCACCACACGGGTGGTCGGC

109741CAGGATACGAATATCCTGACGCTTAAGATTATGCTTTTTCATTAAGAATTGAATTTCCGT

109801TTCAAATTCTTCTTCATAATTAAAAGCATCATCAATACTATATTTCATTATTTTCCAGCC

109861TCAAATGCTCGCATGTCTTGAATATGCTTAATAGCAAATCCACGTGATTTGATAGCATCA

109921AGAGCTCCGCTACAGAAATCTAATAAAATCCCCCAATACTGTAACGAGGTATCAACCTTT

109981AAAACATCCTTATCAGCTGATAGAACTGTCTTCATTTCTGATTTCTCGTAACGATCCATA

110041CTAAATTCATCACCATCTCCTCGTCCCGAGTAGTAGTCTAATTTAGCTTTAAGAGCAACT

110101TTTTTCTGTGCATCAATTCTAAGCATTTCCTTTTTAATACTTGAATGCTTATTAAGCCAT

110161TTACTATATAACATCACATTATTAGCTGCTTCATACTGTAATTTAGTCGAATCTATAAAC

110221ACATCTTTCTTCAATTCTTCTTGAAGATCTTCTAATCTCATATTGTTCTCTATTCAATTG

110281TTATTGGATGGACTTAGATTCATTATACCACGTTTATACGTGAAGCATTATACTCTATTA

110341CTGGAAGCCAGTTGTAGTTTTATCTGCTCAATATCATCAGGGTTATCGATGACCGAAAAG

110401CGTATTTCTACTATCAGAGTATAATCATCATAAACCGGTATTACATTAACTGCTAATTTA

110461TCAATACGTGGCTCATAGTTTCTTACTGCGCTTTCGATATTACGCTCAACCGTGTCAGCA

110521GTAAGAGGAGTCATATTCTCAAAAAGTTGGTCAGATAAATCGCATCCAAATTCAGGGTCA

110581AACGGTCTTGAACCTTTTCTTGTTGTAATAATTCCTAAAAGACTATTTTTAATTGACCTT

110641AATCCAAGTGATCTTGAAACGTCTTTGTTCCAATCCATTTTCATCTCTGGGTCAATATCA

110701GAATAAAGCTTATTAATATTTGCCATTACAATAGCTCAAAGAATTCTTTGAGTCCTCTTA

110761TTACATGAGCATGAGTTTTTCCACACTCTGGACATTTAATTGGAACAGCCAAATAAACAG

110821TAGGCTTTAAAAGCATATCTTTTATAGCTACAATATCTGACTCTGTGATGATAGAATATA

110881AATCTTCTAGTTCCTTTTCATTTAAGTCTTCAACTGGAATGCTTTCCCCGTTAGCATGAA

110941TCGTTTCTATACATGATACTATCATGTGGGCTATATTTTTATCATCAAAAATTTTAGGGT

111001ATCGGAATTTAATTTTAATGTCACCCAGTGTATACCAAAGGTCTTCTGGTGCATCTATTT

111061GTGTATGTAATAGATTTATATGGGTTGGTATTTCAGTTCCGCAGGTGCACTTCCAGGAGT

111121TTTCGTGATTAACTTCTCCAAGAGAATGCGCCCATAAATGAATCAACAATAGTTCTGATT

111181CTTGGCGATTTAAATCTTTTGCATTTGTGCAGTCTTTGATTAGCTTTTTAACAATCTCTT

111241CTACTGAACCATTCTTTTTAGCAGTAATAAGTTCTAGATATTCTTTAAGAGTAAATGCGC

111301GACAGTTGATTATTTTAGAACCAACTCTCACATCAAATTTGTATTCATACATATTTAGCT

111361CCTTTATTTATCATATTTATAAATAGAATAAAAGGAGCATCTATGGCAAACATTATTCGT

111421TGTAAATTACCAGATGGTGTTCATCGTTTTAAACCATTTACGGTAGAAGATTATCGAGAT

111481TTTTTGTTAGTTCGAAACGATATAGAACATCGGTCACCACAAGAACAAAAAGAAATAATT

111541ACTGATTTAATTGATGATTATTTTGGAGACTATCCGAAGACTTGGCAACCATTTATATTT

111601TTGCAGGTATTTGTAGGTTCAATAGGTAAAACTAAAGTACCAGTCACATTTGTATGTCCA

111661AAATGTAAAAAAGAAAAGACAGTTCCATTTGAAATATATCAAAAAGAATTAAAGGAACCT

111721ATTTTTGATGTAGCTAATGTTAAAATTAAATTAAATTTTCCTTCTGAGTTTTATGAAAAT

111781AAAGCAAAGATGATTACTGAAAATATTCATTCTGTTCAAGTAGATGAAATGTGGTATGAT

111841TGGAAGGAAATTAGTGAATCGAGTCAAATAGAACTTGTTGACGCCATCGAGATAGAAACA

111901TTAGAAAAAATTCTCGATGCAATGAATCCTATTAATTTAACATTGCACATGTCGTGTTGT

111961GATAAGTACATTAAAAAATACACTGATATAGTAGATGTGTTTAAGCTATTAGTTAACCCA

112021GATGAGATATTTACTTTTTATCAAATTAATCACACACTCGTAAAAAGTAATTATAGCTTA

112081AATTCAATAATGAAAATGATTCCTGCTGAGCGCGGATTCGTATTAAAACTGATTGAGAAG

112141GATAAACAATAATGAGTATGTTACAACGCCCTGGATATCCAAATCTCAGCGTTAAATTAT

112201TTGAGAGTTATGACGCTTGGAGTAATAATAGATTTGTTGAATTAGCTGCTACTATTACCA

112261CCTTAACTATGCGGGATTCTCTTTATGGACGAAATGAAGGAATGCTACAATTTTATGATT

112321CTAAAAACATTCATACAAAAATGGATGGAAATGAAATAATTCAGATTTCTGTAGCTAATG

112381CAAACGATATTAATAATGTTAAAACACGAATTTATGGATGCAAGCATTTTTCCGTGTCAG

112441TAGATTCAAAAGGCGATAACATCATTGCTATTGAATTAGGAACTATTCATTCTATAGAAA

112501ATCTTAAATTTGGTAGACCGTTTTTCCCTGATGCCGGTGAATCTATAAAGGAAATGCTTG

112561GCGTAATTTATCAAGATCGTACATTATTAACTCCAGCGATAAATGCTATTAATGCTTATG

112621TTCCTGATATTCCATGGACTAGCACATTTGAAAACTATTTGTCATATGTAAGAGAAGTTG

112681CTCTAGCGGTAGGAAGCGATAAGTTCGTATTTGTATGGCAAGACATCATGGGAGTTAACA

112741TGATGGACTATGATATGATGATAAATCAAGAACCATATCCAATGATTGTAGGTGAGCCAA

112801CTTTAATAGGTCAATTCGTCCAAGAATTGAAATATCCATTAGCATATGATTTTGTTTGGT

112861TGACTAAATCAAACCCGCATAAACGTGATCCAATGAAAAATGCTACTATCTATGCTCATT

112921CATTTTTAGATTCTTCATTACCAATGATTACTACAGGAAAGGGTGAAAACTCTATTGTAG

112981TGTCAAGGTCAGGTGCTTATTCTGAAATGACTTATAGAAATGGATATGAAGAAGCTATTC

113041GTCTTCAGACTATGGCGCAGTATGATGGTTACGCTAAATGTTCTACTGTCGGTAATTTTA

113101ACTTGACTCCTGGCGTTAAAATTATTTTTAATGATAGTAAAAACCAATTTAAAACAGAAT

113161TTTACGTTGATGAAGTTATTCACGAATTATCAAATAATAATTCCGTAACTCATCTTTATA

113221TGTTCACTAATGCAACGAAACTGGAAACAATCGACCCAGTTAAGGTTAAAAATGAATTTA

113281AAACTGATACTACCACTGAAGAAAGTAGTTCTTCCGATAAGTAATAAAGAAGTTTCTATT

113341CCTAAGATGGGTCTTAAACATTATAACATTTTAAAAGATGTTAAAGGTCCTGATGAAAAT

113401TTAAAGCTTCTTATTGATTCTATTTGTCCAAATTTATCACCGGCAGAAGTTGATTTCGTT

113461TCTATTCATTTGCTAGAATTTAATGGAAAGATTAAATCTCGTAAAGAAATAGATGGTTAT

113521ACTTATGATATTAATGACGTTTATGTATGTCAAAGATTGGAATTTCAATACCAAGGAAAT

113581ACATTTTATTTTAGACCTCCTGGAAAATTTGAACAATTTTTAACTGTAAGCGATATGTTA

113641TCCAAATGCTTGCTTAAGGTCAACGATGAAGTTAAAGAAATTAATTTTCTTGAGATGCCA

113701GCATTCGTTTTAAAATGGGCAAATGATATTTTTACAACTTTAGCAATTCCTGGCCCTAAT

113761GGTCCAATAACTGGAATTGGCAATATTATTGGATTATTTGAATGAAAAAGCCACAAGAAA

113821TGCAAACGATGCGTAGAAAAGTTATTTCAGATAATAAACCAACACAGGAAGCGGCTAAAT

113881CCGCTTCCAATACTTTATCTGGACTTAATGACATTTCTACAAAATTGGATGATGCTCAAG

113941CTGCTTCTGAATTAATAGCTCAAACCGTTGAAGAAAAATCGAATGAAATAATTGGAGCAA

114001TTGACAATGTAGAAAGCGCAGTGAGTGATACTACTGCTGGTTCTGAGTTAATTGCTGAAA

114061CTGTCGAAATTGGCAATAATATTAATAAAGAAATCGGTGAATCGCTCGGAAGTAAATTAG

114121ATAAATTAACGAGTTTACTCGAGCAAAAAATCCAGACAGCCGGAATTCAACAAACAGGAA

114181CTAGTTTAGCTACGGTTGAAAGCGCTATTCCTGTTAAAGTTGTTGAGGACGATACAGCTG

114241AATCCGTGGGTCCTTTATTACCAGCTCCTGAAGCAGTTAATAATGATCCTGACGCTGATT

114301TTTTCCCTGCCCCTCAACCAGTTGAACCTAAGCGAGAGTCACCGGAAGAAAAACAGAAAA

114361AAGAAGCATTTAATTTAAAATTATCTCAAGCTTTAGATAAATTAACAAAGACTGTTGATT

114421TTGGATTTAAAAAATCCATTTCAATTACTGATAAAATATCAAGCATGCTATTTAAGTATA

114481CTGTCAGTGCTGCTATTGAAGCTGCTAAAATGGTTGCATTAATAATGGCTGTTGTTATTG

114541GAATAGACCTACTGATGGTTCATTTTAAATATTGGTCAGATAAATTTTCAAAAGCCTGGG

114601ATTTGTTTAGCACTGATTTTAAAACTTTTTCAAGCGAAACCGGAACTTGGGGTCCTTTAT

114661TACAGAGCATCTTTGAATCTATTGATGAAATTAAAAAGTTCTGGGAAGCGGGAGATTGGG

114721GCGGTTTGACGGTAGCTATTGTAGAAGGGCTTGGAAAGGTTCTTTATAATTTAGGAGAAC

114781TTATTCAGCTTGGAATGGCTAAATTATCAGCAGCAATTCTTCGAGTTATTCCTGGTATGA

114841AAGATACTGCTGACGAAGTAGAAGGAAGAGCACTGGAAAATTTCCAAAATTCTACTGGAG

114901CATCTCTCAATAAAGAAGACCAGGAAAAAGTTGCGAATTATCAAGATAAACGAATGAATG

114961GAGACCTTGGTCCGATAGCAGAAGGACTAGACAAAATTGCGAACTGGAAAACTCGTGCAT

115021CTAACTGGATTCGTGGTGTTGATAATAAAGAAGCATTGACAACTGATGAAGAACGCGCAG

115081CAGAAGAAGAAAAATTAAAGCAGCTTTCACCAGAAGAAAGAAAAAATGCTTTAATGAAGG

115141CTAATGAAGCCCGTGCCGCGATGATTCGTTTTGAAAAATATGCTGATTCAGTTGATATGA

115201GTAAAGACTCAACGGTTAAATCAGTTGAAGCTGCCTATGAAGACCTTAAAAAACGAATGG

115261ATGACCCGGATTTAAATAATTCACCGGCAGTTAAAAAAGAACTTGCTGCTAGATTTTCTA

115321AAATTGATGCTACTTATCAAGAGCTCAAGAAAAATCAGCCTAATGCCAAACCTCAAACTT

115381CTGCTAAATCGCCAGAAGCAAAACAAGTCCAGGTAATAGAAAAGAACAAAGCACAGCAAG

115441CTCCTATTCAACAAGCATCTCCTTCAATCAATAATACTAATAATGTTATTAAGAAAAATA

115501CTGTCGTTCATAATATGACACCCGTAACGAGCACGACTGCTCCTGGTGTATTTGATGCGA

115561CTGGAGTTAATTAAGGAATAATATGGCAATTGTTAAAGAAATAACTGCTGATTTAATTAA

115621AAAGTCCGGTGAGAAAATTTCAGCCGGACAGAGTACTAAATCAGAAGTAGCAACTAAAAC

115681ATACACTGCCCAGTTTCCAACTGGGCGTGCTAGTGGTAATGACACTACAGGGGACTTCCA

115741GGTAACAGATCTATATAAGAATGGATTATTATTTACTGCATACAATATGTCATCTAGGGA

115801TTCTGGAAGTCTTAGAACGATGAGATCTAACTACTCTTCTTCATCTTCGAGTATTTTACG

115861TACAGCCAGAAACACTATCAGTAATACAGTATCAAAACTATCAAATGGACTAATATCAGA

115921TAATAATTCAGGAACAATAAGTAAAGTTCCTGTTGCAAATATTCTTTTACCTAGATCTAA

115981ATCTGACGTCGATACGTCATCACATAGATTTAATGATGTTCAAGATAGTCTTATTACAAA

116041AGGTGGAGGTACTGCTACTGGTGTGCTAAGCAATATGGCTTCAACCGCAGTATTTGGAGC

116101GCTGGAAAGTATAACACAAGGTATAATGGCTGATAATAATGAACAGATTTATACGACAGC

116161CAGAAGCATGTACGGCGGTGCTGAAAATAGAACTAAAGTGTTCACTTGGGATTTGACTCC

116221GCGCTCAACAGAAGATTTAATGGCTATTATTAATATCTATCAGTATTTTAACTATTTTTC

116281TTACGGTGAAACTGGTAAATCTCAATATGCTGCTGAAATAAAAGGATATCTAGATGAGTG

116341GTACCGTTCTACTTTTATTGAGCCGTTAACTCCAGAAGATGCAGTTAAAAATAAGACGTT

116401ATTTGAGAAAATGACATCGAGTTTAACTAACGTTCTTGTAGTTTCAAACCCGACAATTTG

116461GATGGTGAAAAACTTTGGTGCAACATCTAAGTTTGATGGAAAAACGGAAATATTCGGTCC

116521ATGTCAAATCCAGAGTATCAGATTTGATAAAACACCTAATGGTAACTTTAACGGATTAGC

116581TATTGCTCCAAATCTCCCTAGTACATTTACTCTCGAGATTACTATGAGAGAAATTATCAC

116641GTTAAACCGTGCTTCTTTATATGCGGGGACTTTTTAATGTATTCTTTAGAGGAATTTAAT

116701AATCAAGCAATAAACGCAGATTTCCAACGTAATAATATGTTCAGCTGTGTTTTTGCTACA

116761ACTCCATCAACTAAAAGCTCTTCGTTGATAAGTTCAATTAGCAACTTTTCTTATAATAAC

116821TTGGGCCTAAATTCAGATTGGTTAGGATTAACTCAAGGCGATATTAATCAGGGAATTACT

116881ACGCTAATTACAGCTGGCACACAAAAACTGATAAGAAAATCAGGGGTTAGTAAATATCTT

116941ATTGGTGCCATGAGTCAACGTACAGTTCAAAGTTTATTAGGCTCATTTACAGTTGGTACA

117001TATTTAATTGACTTCTTTAACATGGCATATAACTCATCTGGATTGATGATATACTCTGTA

117061AAAATGCCAGAGAATAGATTATCCTATGAAACTGACTGGAACTATAATTCTCCTAATATT

117121CGTATAACTGGGAGAGAATTAGACCCTTTGGTTATTTCATTCAGAATGGATTCAGAAGCT

117181TGTAACTATCGTGCAATGCAAGACTGGGTTAACGCTGTTCAAGACCCAGTAACTGGATTG

117241CGTGCTTTGCCGCAAGATGTCGAGGCTGATATTCAGGTTAATCTTCATTCTCGCAATGGA

117301TTACCACATACTGCGGTGATGTTCACTGGATGCATTCCAGTGTCCGTAAGCGCTCCTGAG

117361TTATCATATGATGGAGATAACCAAATAACTACATTTGATGTTACTTTTGCGTATAGAGTC

117421ATGCAGGCTGGAGCAGTTGATAGACAAGCTGCGCTTGAATGGCTTGAATCTGCTGCTATA

117481AATGGTATTCAAAGCGTTCTCGGAAATAGTGGAGGTGTCACTGGACTATCTAATTCGCTT

117541TCACGACTTAGTAGATTAGGGGGAACTGCTGGAAGCATTTCAAACATTAATACTATGACG

117601GGAATTGTCAACTCGCAAAGTAAAATATTAGGAGCAATATAACAATGGGGACCGAAAGGT

117661CCCCATATTTTTATTTACGGAATGAAATAAAAGCAGCAACTGAAGCAACAGAGGTTTCTT

117721CAATATAAACCTCAATTTTTACCGGAGCTTCTGACTCAAATTTACCTGTTACTACACCTT

117781GGAAAATACTTTCAGTCTGCTCTGGCTTTGAAAAATTTTCAGAAGGAAAAATTCCGAACT

117841TTTTATCGGTTCCGAAAACTTTAATAAATTCATCATAAACTGCTTCATTGAAAGCGTTAT

117901CAGCGGGAATAACGCCCTCAACGACGAGTTCTTGACCTAAAAAGCGCAAAGAAGATTTCA

117961TTTTGTGTTCCTCATGTTATGTTAGTAAGATTACTATAACACAACATGAAGGACTTGTAA

118021ACTACATCTTGAACTTTTTAGACATTCCGTTGATGTTAACCAAAGAAGCCAAGATTTGAG

118081AAACTTCGTTTTCCTCTTCTTTTGATTCTTCTACAAGGAATTGATTAAATCCAGCCATTG

118141GACGAACTTCACCAGTTTCCATTAGATGGTCGCCATCATATACAGATTCCTCTAATTGTT

118201CAGAAGGAGGAACAATCGTGCCTTCAACAAGATACTTATTATAGGCAGTTTCTTTCATAA

118261AGTACGTACTTACTTTATTAATCTTCAATAAAATTCCACGTGGTAGAATAACTTCAGCTT

118321CTGATGGAAATCCTGATAAATGACCCGGAACGATAACTTTAACTCGTTCAGCTCCTTTAA

118381TTACCATACCCACGCTAGTTTCAGCATCTTCATTAGCATATGTTGAATGACTACCCATAT

118441GCATTAAATCTTCGGCATCAACTGAACCTTCTCCTTCGCCAGAAAATACTGCACCGGAAT

118501CATCTAGAGCCATATAGTTTTTACCATGCTCACCGAAGATGTTTGGTTTAAGCGACGTGG

118561ATACAAAGTTCTTGAAATAGAACATTTTGTTTTCAATATTATGACGTAAAGTTTTAAAGG

118621TAACTTCTTGTCCACGGTAAAGTGTAGTTCCTGGAGGAAGAACTGAACCTTTAGCGAATG

118681CAGAATCCAGCGTCTCAATATGTTTAATAGCTCGTTCCATATATTCTTCACGGGTAGAAT

118741CTGGTTTACCCAAAAGGAACGAGTTCATTTCAACATATGAATCTGCACAATAATCTTCAA

118801TTACATCAGACTCAGCCTGAGTTAATCCTTTTGGAGTTACTTCGGTGCTTGCATATTCAT

118861ATGCCTGAGCAGCAGATTCATTAATCAATTTATAAATGCCATTCATGACAAATTGAATAA

118921TAGCTACTTTTTCGTCTGGCGTGGCTTTAGAACCTTTAACTAAATCATTGATAATTTTCT

118981TAATAACATCAAAGCTATTAGAAATATTAATGTCAGCGAGATCGCTTAAAATTTTATCAG

119041TTTTATAATCAAAAAATTCTTTATTCTTTAAATATTCTTGAGAACCTTGATATGTTATAT

119101CGCTCTGCAGTCTGATAGATGGATCACGTAAACGTGCATGGGCCAATGCAATATGATTTC

119161GGAAGCTTTCTAGTTCATAATCGTTAGATTTAAAACGAGAAGCACTTCTCATACCACCGA

119221TAGCTCCTTTGCCGAAGTTTGAACCAATCTGGATAACGCCAGAGTTCATTGGTCCTTCTG

119281CTTTATATACCGGAACTTTAGATTCAAACTCTTGATATTTCTTGGCAGCGAAGCTATCAC

119341CCTGAAGTGAAGCATCTACAGTAGAATATTGAGCTTGCGCTGCTAATCGACGACTAATTT

119401TAGTTTTCTGAATAACAGCTTGATCCGTGCGCTTATCATTTTCTAAGGCAATAGAAGCAG

119461CAACAGCAACAGCTTTAGGAACAGCATTACCTGTTTTAACATCTACATAAACATCACCAA

119521CCTTTGATTCAACTTTTGTGAATAACTCAGTTGAAATAGGAGGTACAGTTCCTTAAGAAG

119581GACGTATCTGCCACCTGAACGACTGCGGATGAGTCTATCAGCAATAAGTTGAATTTGTCG

119641AGCTTGACCAGCAGTTTTAGACTTAAGAATACGAAGCATGCAGGCATCAATTTTATACTG

119701ACGCATCGTCTGCATTGCAACAGTAAAAACTGAATTGATATAATTAATTGGGCTTGGACC

119761GAGACCTTTTAATTTAGCAATTGAACCTTTAGCAGTTAATGTAAAAGGAACAATATGCAT

119821CATTTTATCGCCCATCTTTAAATCGCGATTAGTATCACCTCCAGATGTATAGGTACATAA

119881ACGAAAGCCTGGTTGTTCAATTGCATCATCAACATGAACTGAAAAAATTTGTGGTATTTT

119941CTTCTTTGGATTTAAGTTTGTAATCGGAAGAGTAGTATCTTCGTCAAATAATTCTGTAAT

120001GAGTTCCATCATATCCTCTCTAGTGTTTATTCTATTTATAAAATTAAAGGCCCGAAGGCC

120061TTTAATAATCTATTGGTAAGAGAGTACGATATATTTCAAACTTTGGACCTTTTTCATAAG

120121CATCAAAAGTTTCTGTGAATTTATTATAAGCATATGCATCTATAAATTCAATCATGATTT

120181GTGATACAGAAGTAGAAACATCTCCACCTTCTTTTTGAGCCACGACAATTGTTTCTAAGT

120241AAGCTTTCATAAACCAGTTACCTCATGAAAATCGCCAAATACATCTTCGAACGTATTAGC

120301TTTAGTTTTATCTTCACGTAAACGAATCGCAATTGGAAGAAATAATTTAACGTAATCAGT

120361GCGGCCATCAGATTTTAACCAACCGTTGCATTCGCACTCTAGAATTTTTCCAATATAATA

120421ATTTTGGTTTTCCATAATGCGAGTACGGTCAAGTTCATGCGATTTTACACCTGCTTTATC

120481TTTTAAGCCTGAACCAGCATTTACTTTAATTTTTCCACACTCTGACTCAAGAATAAATCC

120541ACCCGCTTTAGTAGGGTCTTTACGGTGAGGATAAATTCCTACAATTTTTAAATCAACATC

120601AATTACTTCTTTAAATTTATAAAGATTTTTTGAACGAGCATTTTCCCATAATCCATCGAT

120661ATTTTTGAGAATAATACCTTCAAGACCTTGGTCAATATACTTTTTGTAAATTACCTTAGC

120721TTCATCTAGGTTATTTACTACCTGGTTTTCAATTAAAATTACTTTATCATAGCCTGATGT

120781CATTTGTTCTAGTTTAGAAAAACGTACATCATATTTTAAACGAAACGCCGGAAGACCGTA

120841TATTTCTACCAACGGGACATAATCCCAGACCTGAAACTTCATGCATTGAGCTTCTTTTTC

120901TGAAATAGTTCCCTTTAAAGATTTATTAGCGATTCCATTAGAAGCAGTACGTGATTCAGC

120961TACTTCGGCGAATTCTTTAGCTTTACTAATTTCAGGATAAGCATCAAAAAGAAAATCTAG

121021GCCTTCTGGCTCTTTTTTAACTTGCTCATGGTATACCAATTCGCCATCAATTAACACACC

121081TTCTGGATGAATCTGGCGGGCTTCAGCGGTCATTTTGATTAACTCTTCTTTGAGAAGGTC

121141TAATCCTAGATATTCATTACCAGCTCGTGATAAAAGACGAACATCATCTAATTCATCGCC

121201TCTGACTTCAGCAAAACACCGAGCTCCATCAGCTTTTAGCTGCGCAAAGGCTGGAAATTT

121261GATATTCTTATTAATGCCTTTTTCGTCATAAGAACTTGCAAGCATTTGAGGCTGTTCAGG

121321AATTAAACCTGGCCAAACTTTATTTGCAATAGATACAGAAGCACCGCATTCAAGGTCTCG

121381CATCATCACCCGACGCAAAACTTCAACATCATCTTTTTTACCGTCAGTAATATATCCAGT

121441TAATTCCTCAATTGCCGCATTTCCAGTCAATTTCCGAGTAGCTAACGTGAATTCAATGAA

121501GTCGAGCATATCGGTAAGAGTCAACATTCCAAAACTCTGGGTAGCAATACCCGGTTTAGG

121561CCATTTCTTGATATAATACTGTAACCCACGAGAATAAGTCAGACGATATACTCGTTTAAG

121621CAATTCATTATCTTTATTCTTTTCAAGAATTGCTTGCTTCTGCTTAGTTGAACCAATAGA

121681TGCTATTTCGTTCAAAATTTTAAGAATCATTGTTCATCCTTTAGAGTTTGGTTTACAGCT

121741CTATTATAAATCAATTCATCATTAAGCTCAGTCAAAGACCTGTGGTACGTGGTTCTAACT

121801TTATTTCCTTGCATCCAGTGCTTGACGTAAATGAAACCTTGCTCTACGCATTTTTTAAAA

121861ATTCGTTCGTCTTTTTGAGCTCGGAATTCTGGATTGCATCTAAAAAATTGATTTACGTGA

121921CCGTAATCACGTGTAGTATTACCTTCATTTTCATAAATAGTATGAACAACAAACATTAGA

121981ATGCTCCTTGGAAAATATTATCACCACTAGTAGGTCTATTATACAAATACTCTATGCCAC

122041CAGGTTTAATATAGTTCCATGTCTGAAACGGATGTGTCTGATATGGATGATATAGATGAT

122101TATAAGGATTAAATCCAGGAGTTCTCCAGGTAGTGTTCCAAGGAAAAGAATCGTTTTTAA

122161TCATCTTTTCAATTACATCTTTTATAGCTTTTTCACTATCAAAACTTTCTTTATTTTCCT

122221TCGATGGAAAAAGCTTATTCTCGACATCATTCCATGTATAAACTCGCTGTGCAGTTTTTG

122281GAATACTGTCACGCTCCCCTCGAGCCATCCAATAAACAGGAACATTTAATATTTCACTCG

122341CGTGATCGCAGTGGTGAGCGAGATCGTCAATATAACAAATTACGTTATATTTCTCTTTTG

122401CCTTTTTGAACAACTCTTCTTTTGAAGAATCATGACCACACATCAATACTTCTGAGAAGG

122461CACCAGGAAAAAGAGCATTCAAATTAAATTGACGATTCAACAGAGCGTCAATAGAATCAC

122521CCAGCGCTGTAACAGCTACAAAATTATAATCTTCTTTTAATTTGTTAATTACACACAGGG

122581CATCTTTATATGGAGACAAGTAACGAATAAAATCTGAACGATTGTATTTTTCAATTAGCT

122641TAACGCCAAGTTCTTCATCACAATTAAAAAGTTTACCAGGAGAAATAAATTTTTCATCTT

122701GGATCATTTTTAAAATATGTTCCAACGGAAGATTATATTTCTGAGCAAAATAAGGAAGGC

122761CTGATTGCCAGCTTAAACACACCCCGTCAATATCAGTTAAAATAGTAGGCTTCATAGAGA

122821ATCTCTTAATAGGTTTAACACATCAATAAATTCAGCTTCGGTTAGTATTGTATCATCTTT

122881TGTTAGACCACTAGCCATGCTGTGCTTCAAAACTTTTCCTTTCGAGGCTTGTAATGCATC

122941ACGAAAGCCCTTGTTTTGAATCGCTGCTTCAAAATATGCATTTGTGTATAATTCTTTCCA

123001CGCTGGAGAGTATCTTGAAAATGGAACTCCGAGCCAAAAGAGGGTCCCATGGTCCTGAGC

123061TCTAGAATAAGACCTTCCAGCTTGTTGGGCGGCAAGCCCGGATAACCCAAATATACGTCT

123121TTGCTGTTCAACATTTTTCACCTTACACCCTTGGAGGAATCCTTCGAGACCTCCAAATTG

123181AATGCCATCCATAACGAAAGGCCATTGGGCGAAATTACTTAATGCACATGATGGCCATTT

123241AAAATTGCTTCTTATCTCTAGCTCAGACATTTTCAATACTTATAATTTCAACATCAGCCC

123301AATGACCATAGCAAGGAAGACGAAATTCAACTGGCCAGTTAGGGTCCTTTTCTAATATAA

123361TAGACTCAACTCGTGGTTCTTCATGTTCATCAGTATATGGATTTTTATCTGTTACTTTAT

123421ATGTGACCTTAATGTACTGAATTCCAAAAATCTTATTAATTATATTCATACTAATTCCTT

123481TAATCCGTAAATAGGAGATAATTCATCACCCATACGAAGGTCTTCATTTCCATCTACCCA

123541GGAAACAATATAAGCCTCTTTTATTTGAACACCACTCCATTTAAATGGAGGTAGAACCTT

123601AGAAATTAATCCAGGTATACCAACTCCCTTTAATTCAACAGTTTGACCTAAAAAGAATTT

123661CATCAGAACCTCATCTGAAAGCCGTGTGATTTAACATTACCGCCGCCAATATCAGAAATA

123721TTAATTTCACGCGCAATTGAAGGGTCAATGTCAATTTCACGATTAAGTTTAGCGATAGCT

123781AAAGTATCACGCCCATTTACTGTACGGAATTCTAAAGGACAAACCACATCAACATAATTT

123841TTAATCGATTCACTGGTAGGTTGCAAATGAGCAGGAAGGTCTTTAGGCGCCTTAGAGAAA

123901ACAACTTCACAGAAATTTTTGCGGGTATCAAAATAAGTAGTCATAAACATAGTATTTTCC

123961TCAGTAAGGGGCCGAAGCCCCTGTTTTATTTTAAATATCAAATTCGTTAAGAACTACATC

124021AAAGATTGCTTCAAGATGCTCAGGTTTAGCTCTGTTACTCAAAATATGACGAATCCAAGT

124081TTTAACCAGAAGCTTACGATTAGCGCCATTCCAACAAGGATGGGTCCCTAAATCGCGTTG

124141ACGAAAATCATCATCCAGAGCGATTTTGAAGTTTGAACCTTCCATTGTGATTGAAACCGT

124201GATGCCATTTTCAAATCGCATATAAACGTAGTTAGGAGTCATATACTGTTCAATTTCGCA

124261TACTGATCCATTTTGATGTTTCCAAAGGCAAATAGTATCAATAGAACCTGCAATACCATT

124321AGAAACATATTTACGTTCAAAGTTGATGTAGTTCATTTTTATTCTCCGAGATGTTTAATT

124381GCGGTACAGGTATATAATATCATATCCTGTACCAAAGTAAACAATTATTTTACTACTTTC

124441CAGTGCTTCATGTCAAGTTTACCAACTTTTTTCATCTTCTCAATCAAACGTTCGGCACGT

124501TGGCGAGCTGTAACATAATACCATTCGCCTAATTCATTTTGTTCAATTTTTCCAACGATT

124561ACTGTATTCAATTCATAAATCCAACCAGTAAAGAAATTATGAACTTGAATTGTAAAGGTG

124621AAATCTGTTCCCATACCTTCTGTTGTTTCTACTTCAATAATATCACCTTCAACTGCCATT

124681AAGAACCACATAGTTTCATCATATTTACCATTGAAGCATTTAGTCTTAACTGCAGCGTTC

124741AGATTAATCGTTTTCATTTTATTCTCCTTTGTTTGTGTAAGATAATACTATCACAAAGGA

124801ACCATACTGTAAACAACTTTATGCAATCTTTGGAAAATAAAAAGGACTCCCGAAGGAGTC

124861CTTAACTTATGCTTTCTGCTTACCAAAACGAGAAGCATCATCTCGAAGAACCGCACGTGC

124921TCGGCGCATGATCTTCTCAACAGTTTGATTGATACGAGAGTTCGACCCACGCTTGTAGCC

124981AGCGCGTTTAGAATCACCAACTTTCTTTTCAACTGCTTTCTTTGCTTTAGCTTGTTTTGC

125041CATTATAAATTCTCTTTTAAATGAAAATGCAGGACTTATTGGCATTGCCTGCGCAAGCCC

125101TCAAGGGGAACATAGGTTTTGGATATTTAACGACAGGATAACCATAAACCCGTCATCATT

125161CACATTCAAGAGGTACACCATAAAACTGCCGGGGTCTTAAAACTATAATGATTCGCAAAT

125221CATTAATCAGACAGTTCGATGGCTCCTCGATTTTAGCTCACACTAAGGCAGTGAATCTCC

125281AATAAATTACTTCAGTGTTACCACAAAGTGACGAACTGCTTTTCGTGCAGCAGAAGCCAG

125341AGGCTTAGCATACTTAAGTTCATCTTTTTCCTGAAGCTCAGCAGCTAATGCAGTTTGTGC

125401AGGATTCAGATGTTTGAAATAACGCAGGATTTCAAGAGCTTCGGCTTCAACATCAATAGA

125461TGCGCCATAGTTTTCGTGACCATTATTCCATGCGTTTCGTTGCAGTTCAAGAGCGTGTTG

125521TAATTGTTTAATCATTTAAAAATTCTCGTTAGAGATTAAAACTCGGTAATCACGTTCTTC

125581TGAATTTCATCTTCTTTCGACAGATCTCTCAGTTGTAGACTACCACATAGAATTGTTCGG

125641TTAACTTATTATTCCGACACCCAATTCATATTATTATTTATATCACTTATAAAGACATGG

125701AATAGCTTTATAGTGACAGGTAACGAATTTTTGTTTAATTTCTTTTGGCTGTTTAAGACC

125761CAGAGCTACAAAAGGATGCGGAACATTTCGAATTTGACCAACTGGAAGAGAAGTCAAATC

125821ACCAACTTCGCAGAAACCTTCAGGAACATCAGGACCGACAGAGTGAACTACACACAATTC

125881AGGAACTTCACCTTGGACACGTTTACCGATAATAAGTCCTGATTCTGTAACTTCTTCATC

125941ACCAGCTTGTGCAGGTTCAGAAACTAAAATAACATATTCACCGACAGCACGAATTGGTAG

126001CTGTTGTACTTCAGACATCGTTTTTCCTTTTTGTTAACAGATGAATTAATAATAACAAAT

126061AATTCTTAAAGCATTTATTTACCAATAAATTGGAGTAAATGCTCAACTTTCATACCATTA

126121ACAGAAATCAATTTGTCAATAGAAAAACCTCGCCATGCACCAAGTTCAACATCAAATACT

126181GGAATCATGTCAGTAGATTCTTTCCGAGTCGATTCAGTCAATTTACCAGTTTGCATGGTT

126241GGCATAAAGTCTGCGTCACGAGTACCTTTCATAGTACGAATAGTACCATCAGACTTTTCA

126301AAAACTACGTTTGAAACACCCATGGACAGTTTAGTTTTCAAAATTTCACGAATTGCTACT

126361TTTTGTTCAGTCGTCAGTTTCATTTATTTACCTATCACAGTTTTAATATGAGTTGTTCCA

126421CGTTCTTTAAGAGTGGAAAGCAATTTTTGGCATTTTTCTAAATCAGATTTCCAACTATAT

126481GGTCTATCTATACTAGTCCAATCAGTTTTATAATACTGCTTCCATTTAGAGAAAAAATAT

126541TTCTTATACTCTACAGCAAATGAGATGTTCTCGTTGGAATAAGAACTAATTGCTGTGAGT

126601TGTACCAAACGAAATTTCATTATTCACCACAGAATTCGTTGATATTTTCCCAGTTTAACT

126661TATTCAAGTTTTTCTTAGGAACATTAAACACTTCAATACCTGCATTTCGCAGAATATCAT

126721CCCAGCCAGGTTTATTTTTATCGTATGTTTCGCAATAAACCAGCTTTTTAATACCAGATT

126781GAGCTATCGCTTTTGCGCAATCCGGACAAGGAGAAAGTGTTACATACATAGTAGCACCTT

126841CAATTGAAGAACCATTTCGTGCAGCAAACAAAATCGCATTTAGTTCAGCATGAATTTCAT

126901TTTTAGATGACCATTCCGAGTGAGCACTACGATGTTCTTTCGCCAAGACAAAGCGATCAG

126961TTGAACCAAATGATACACATTCAGGCTTATGACCTTGAATGATAGTATGTTTAGGTTTAT

127021TCAGCAACCAACCTTGCTCAGCAGCATAATCACAACAGTTCACACCCCCTGCAGGTGAAC

127081CATTATACCCAGTAGAAATAATACGTCCATTCTTTTCAATTACTGCTCCTACCTTCCAGG

127141AGCAACATTTTGATTCTTGTGATACTAAATATGCAATTTGAAGTACTGTACTCGCTTTCA

127201TTTCATAATCACCAGATAAGCAGATTTAGCAGTTTCAACACGATAAATTTCGTGACGAAG

127261TTTAGTTATACTTTTAATAACAGAACTAATTATATTCTGCCCATCTTTAAAGCGGTTTTT

127321CTTATCAATAAAAATTGCGCCAACCATCTTTTTGTGAAGCTCAACTGGATACTTCGTCAC

127381AATAATAGCATCATACACAGAAGGATGAATACTATTCACTAGAGTATCATTCATTAAAGT

127441TATTCTAATGAACTGTGCTGTTTCAGAATCAAGCGCTCTATGATCGCCAGTATCATTTTC

127501AAGACAATTATCAATTATATCAGTTAAATTCATCATAGTACGCCATACACCCTTTGTGCT

127561TCAACTAATCCATCAAAATCCAGTTTAAGATGCGATATTTGATCGCCATCACCTGGATTC

127621ACAATTACTAATACTGAACGAGGAGTTTCGGTAATAACACGAACCGATGTTTCTGGAAAT

127681CGTTCAGAAACCTTATTTACCAATTCCTGAGCAAATAGTTTAACTTTTTCTTGGAATTCT

127741TTAACAGTAATTGGGTTTTCACTTAGCATGTTGATCCTAGCTCCTTACTTTCAACGTTTT

127801TAACTAATTTGACAGAAACTAATTCAGCATCTTGAGTAATCATTACCGGACCTTCTTCAT

127861ACTCTTCAAGATATTCTAGAGCTTTCTGTTTAGCTTCTTCCTTCGTATCAGTTTGAGCAT

127921AGATATAGAACATACAACCAGTAATACCTACTACTTTCCACTTAATTTCTTTATCAGTTT

127981TAATTTCAAATTTACTAACAGAATCATCTTTTTGAACTTCTTCGAAAAAAGAATATTCTA

128041ACATATAAGCAATTGAAAATAACGGGCTAACTTTATAGGATTCACCCATTACATCAACAG

128101CTTTATACCAACAATCATCAATTTCGCGTTTAATGCTAATAACATTCTTTGCAGGGTCAA

128161TTTTTACTTTAAATGAATTTTCCCCAATAAACTGGGCGATTCGTTTATTAGCCCCGCAAG

128221TTTCATTAAAAATATCTTTATACCCAGTTTTAAAGCGATAAACCTTATCTTCTTCAAACC

128281ATTCGCTCATTTCAATTTCCTCATTTGTTTTGGTAGAGCTATAATATCACAACTCTACCG

128341TAAAGTAAACCATTAAATCGCTTTGAATTCCGCAGTTTGAGATTCAAAGCGAATGTCGCC

128401TTTGATAACTAGCTCAGCATCAAGACCGAATACAACAATGATATGCGCGGAACCTGGATA

128461CAACGTAATGGCAATAGAATCCACCTGGTCTGGAAGCAAAGTGTTCAATACATGAGTCAC

128521TTGAGCGTGGATTCGAAGCTCAGCTGCGTTATCAAGTTTTTCAAACATATTATTAGCGAT

128581AATTTGGCTAAACACTACTTCTACGATTTTAGAGTAAGTCGGAAACATATTTACCTCACA

128641TAATTTTCTTCGAGCCAATCAATAACATCCAAAGCGTTATCAAAAGTTGCGCCATCTACT

128701TTGTCTTCTGTTTCATAATCAAGAACATCTAGGCCTACTCTTCCGTCAACAATAGGCCAT

128761AGACAAAATAGATATTTCTTTTCTTTTTCAATTTTATCACAAAGACGATAAATCTTTTCT

128821AGGTTATTCATACGTTTTCCATGGTAAAGGCAGTTTAGTTTTCTTTACTACTAGTTCAAC

128881ATCAGGATTCTTTTCTCTTAATTTAAGGCATTCCTCCCATGCTCTATTTTCACTAGTAAA

128941TACACAAAATTGCCCATTACTAGTACCAACTAAACCGCTATTTACAATAACAATAGCCCA

129001AGTTTCATGGTGCCAAGCCATTAAAAATCTCCCGAAGCGACTTGCCAGCATTCAACACCG

129061ATGCGGCGCCACATTTCAACTACTTGAGTTCGGTCATCAATAGCTAATTTCACATCAAAA

129121TGTGGTGCGATGTGTTTCCAGAAAATTTCTTCTTTAACTACATCATCTTTACGGGTATCG

129181CCTTGTTCGCGTTGACACTGCATGACTAATGGAACACCAGCAATGTCCTCAACCCATTTA

129241CGGGTCATACGATAATATTTCGTTGGGTCTTCTTCGGTTCCACTTTCACGACCTGAAACG

129301ACTACGATTTGATAACCCATAAGAGCATACATCTTGGATAGTTCAACGACCATTGGATTA

129361ATAATATCGGTATCGCATTTTTCAAGGTCATAAGGACCACGACCATTCATTTTTGCTAAC

129421GTGCCATCAACATCAAAAATAACTGCTTTTGGTTTACCAGGAGTCCCTTTATATACCGGA

129481AGACCGAGATACTCTCGCATGCTTTTATACATTGAACGTAAAACATCAATTGGTACTGCT

129541TTAGTTCCGCGTTTTGAGTTGCGTTTAACCAATTCAGTCCAAGGAACATCAAACACTTTA

129601TATTCAATTTGATGCCCAAGCTCTTTGGCAAACTCTTCCCAGACCTTACGTCGTTCAGGA

129661TTCAGATTAGTATCTGAAACAATTACACCCTTCGTTGCGTCTTGGCAGAGAATCATGTTA

129721GCAACATCATGCTGCATGTAAGTTACGATACCTTCTTTCTTTTTGGTATACTTATACTCG

129781TCGCGTTCTTCATGACCCATGATGGATTGACGATAATCATCACGATTAATATTATAAAAC

129841CCTGGATTTTTAGCAATAAATTCGCGAGCCCAAGTACTCTTACCAGAACCAGGACAGCCA

129901ATAGTCAAAATAATCTTTTTCATCATTTAATTCCTAAGAAAACTTCAAGAATACGAATAT

129961TGTGCTCACGCCGTCCTTTGTACAATTCAGCTGTTGATTGATTCGCTGATTTACTCTGAA

130021CATTGGATGAAATAAATTTTATCATGTGATTTTTAAGCTGATACATATCAAGCCCACGAG

130081CTTTCGCTTCTTTTCGCAGGGCTTTACCAGCATCATCTAAAGCTTTAGCAGGGTCTTCAT

130141CATTTAATACTATTCCACGATCCAAATCCATGTATACTGCACCATGACATGCATCAATGT

130201AACGGTCTGAGCATTCAATATAACGTTCTAGCAGAGTTTTCATTTATTTTTCTCAACCAA

130261TGATTGAATATAATCATGCAGGTCTTTAGATGCTTTGCCCCACTTATTTTGGTATTCATT

130321TTTAAGATTAGCACGTGATTGAGCTAATAAAACATCATTAGTTGGAGGTAAAGATTCTAA

130381CCGCTGAATCTGGCGTCCATAAATCATTGCAGCCATCTCGGATTCATAAATCAATCCTTT

130441GAGATGTTCAAATTGATGCCATGAAATCATTTACATTTATCCTCTTTTAACTCTTGACGA

130501TAATAACATATCATAGTTTTTTGGTCATGTACATATCGTTTTACATCATTAAGCCAAATA

130561CGAAATTCCTGGGAATCTTCAAATGGCATACCGACCCAAGCTTTACCATCAATAACTTTA

130621ACTTGCCAAGATAGTTTAGCTTCATCATATGACTTTATCTGTACAGGCCAATTAGGATGA

130681ACTGTTTCTTTCTTTACTTCTAGAGGCTTTGTCGAACAACCAGCTAGAAGACCGATAGAT

130741AATATTACTGCTGATAGTCTAATCATTTAGAAAGGTCCTGGATGTCTTCTGCGAACTTGT

130801TGAAGGAGTTGTTGATTTGTTTTTCAACCAATCCTGGCTTACGAGCCACCACATCCGCCT

130861TCTTTGCATCTTTGCGCAGTTTTTCATTTTCACGCTCAATAGCAGCAATCGCCTCACGAT

130921TTTTATTATTCATCGCATCAATATAATTATACTGAATTCGCAAATTATTTAATGCTAAGG

130981CGTTTTCATTGGCTGTTTTTGTAATTTCAGTAACTGATGTTTCTAATCTTTCTACCTTAT

131041TTTTTAAAATAATAGATGTTCCGCCAAATGCTATTACAAGTAATAGCAATCCAGCTGTAA

131101AATTACTTAATTGCATAAAGTTTTAATAACCTCTATAATATCGTCTTGAGAAAGACCGTT

131161AATTAAAATATGATGTTCAGCCGGAGATTTAGAAATTTTAAAGCATGCCTCAACATCTTC

131221TGCCATATCCGATGCGCTACGATTTGGATTACTAATTCCAAGACGATGTTTTCCCGTTAA

131281AGGATTAACGATGATATAGCATTTACAGTTGTTAATATGAACATTAGGTTGAGTCTGATT

131341AATGAACACATCGCAATCATATTTCGCAAGCTGATTTTCTAAAAAGACTTTCATCTCCTC

131401AACCGCATCAGGAAGCATATCACGGGCTTGCTCAAGACGACGATTTCGATATTCTTTAAT

131461GGTCGTTTTCCGCTTGACTTGCTTAGCTAAATCTTTCTTAAGATCGGTAATATATCCAAC

131521TCGACGGTTTCCTTTAAATACAGAAATCCCATCTGTAGTATCACCGTATGCTTCAACGAC

131581CATTTCAGTAGTAATGAGCTGCAAATCCATCATAAAGTCCTCATGTTATGTCAGTAAGAC

131641TACTGTAACACAACACGAGGGACTTGTAAACAGCTTAGTATCCTTCTGGGATAAATTTTT

131701TATAATTTTTCAAAAAATTCTGTTCGATTTCACACATAACCTTCTCTTGACTATCATACC

131761CCTGGTATAAGCTCATGATGATACCGAACAAGTGATCCATTCCAGCACCTTTAGCAACAC

131821CTTGTGCTTCCATTGCATAAGTCTTTCTATCTTTACCACAATGCTTATTGTGACAGTCAA

131881GAACTAAAAACAGAGCTCGGTCTAAGTACTTCAGATAAGTCGTCTCAAATGCTTCAATTT

131941TTCTATATGAATATTCATCGTCAGCGTACATTGCTTTAAGATCATCTGATGCACCATCAA

132001TAATAGTCTTAAACAGTTTTTCTGGATTGTCTAATGAGCTTTTTGTACTATGAAGAGACA

132061CGTACCAGTCAGACTTAATTTTAAAATGAGAACCATCTTTCATCACAGCAACATAGCCTT

132121CGATGTTTTCTGCATTTTTAGCTTCTTCTACCCATTTAGGACTATCGATTTCGTATCGTT

132181CAACTAGATACGGACGAAGAATAGCATCTTTATAAATATCATCGTATGAAATGTATTCAC

132241CCGTTTCGTTTTCACGAACATTCAGTAAAATAATTTTCATTTCTTGGTAAGCAAGAACGA

132301TTCTATTCGTCGGAGCAACGAATTCGAAGTTAGCAGTAAATCCATCTTCAGCTAATTCTT

132361TAAGTCTATCGCGCAACCGATGGTGATTAATATTCATCAAAATCCCATTAGCCATTAAAG

132421CCTGCTCAGATTTGATTGAACCCTTTGATTTGAACAGAATTTCATCACCATCTAAATAAG

132481TTGATACCAAAGACCCGTCTTCTTTTGTTAGAATGTAATCAACATCATTTAAATCGATAT

132541TCATCGTGAATGGATTTTCATTCAAGTTAAAAAACTTTTCCATAGGACGAGAAGCGATTC

132601TTACCGGTTTTTCTCCATCCATTTCAAACATGATTCCACGACATTCCAATGCGTCTGGAA

132661GTAACCAATCAGAATAAGATGCATAATTATATGAGAAAATTCTATAAGTTCTTCCAGATG

132721CACTTACATCATCCGAGTAAAAAAACTTACGCTGTGAATCCTTACATAGTTCCATTAAAT

132781TGTTAAAAAGTTCTTGCATTGTGTATCCTCTTTTGTGTTTTGAATATAGTACCACACTCC

132841ATGTGGAAGCATCATTTTTTCTTATGTTGAATATTCCAAGGCGGGTTAAACAGTTTAATG

132901AATAGCGGCTCCTCTAGGTCAATCGTTGCGATTGTCATTGTACCTAACTCATTTGTCATA

132961GAAAGATTAAAACATTGGCGGGCGTAAAATTCAACTTTGCTTCCTTCCTTTAGCGCAGAA

133021TGAATTAATGCAGATTTAGTAGAATCAGACGTTTTGTCTTTACGGTTAATAGCAGTTCTA

133081TAATAATTTATTCTTTTACGTAAATTTTTAGTTTTTCCAATATAAACAAGCTCATCATTT

133141ATAGCAATAGCATAAATTACGTTATACTTGTTTGGAATAGATAATTGTTTTATACTTCCG

133201TTGTCGTCTAATTCTAGCTCAGTATATTTAATAAATGAATATTCTGTTGCAATTTCTTTC

133261ATAATAAAATGGGCCTTGCGGCCCACTCCTTAAAAGTATTTTTTAAAACTCATCATAACT

133321TTATCATCAACATCATTATCAATCTGTGCAACAAGGTAAGATGACAGTTCTACTTCTTGT

133381GGCGCGGATTGAACATTATCAGAATTAAGATATTCACGAATCCAAGGATATGGATGTTTA

133441ACCGGAGCATCGGTAATTGGGCATGGAAGACCACACTGTTTCATACGAGATACAGTTAAG

133501TAATCAATAAAGCTCCACATGCTATTTGTATTTAATCCAGGAACATCGCCATCTTTAAAT

133561AAATGAACTGCCCAATCTTTTTCTTGGCGGTTAACTTCCATGAAAATATCAACTGCTTCT

133621TGCTCGCACTCTTGAGCAATTTTAACCCATTCATCGCCATCAGTACCAGATTGAAGTTGA

133681CGAATAATATATTGTGTACCTTTAAGATGAAGCTGCTCATCACGTGCAATGAACTTCATA

133741ATCTTGGCATTACCTTCCATGATTTCCATGTTTTTATGGAAGTTAAAGGTACACGCAAAG

133801GATACATAAAAACGAATAGCTTCTAATGCATTGATTACATGCAAACAGAGGTAAAGAGAT

133861TTCATTAGCTCACGCTTCCAATATGTCTCATGCTCAATAGCATCTTCAATAACGTCTTCG

133921TCTGCATTAATTTCTTTTTGGTATTCGATATCAGCTTTAGCGTTTTCCCAATAACGAGTT

133981TTAATCAGAACATCATCATAATAACGTCCAATGGATTCAGCACGTTTCATAATAGCTTCA

134041TCTAATACAATTTCATCAAATACCTTCGATGGATCAGTATAAAGATTTCGCATGATATGT

134101GTATATGAACGACTGTGAATAGTTTCACTAAAAGTCCATGTAGCAACCCATGTATCAAGA

134161CTTGGGTCTGAAATTAACGACATAAGTACAGCAGATGGTGCACGACCCTGAATGCTATCC

134221AAAAGTGATTGATACTTCAAGTTGTTAGTAAAAATATCTTGCTGATATTGAGGAAGCTTG

134281TTAAATTGCGCAGCATCCATCATTAAGTTTACTTCTTCAGGACGCCAGAAAAATGACAAC

134341TGCTTTTCGGTTAAATCTTCAAAAACTTTATGACGTTGAATATCATAACGCGCAATACCA

134401AGACCTGAACCAAAAAACATAGGTTCTTTTAAAACATCAACTGGATTTGTATTAAAAACT

134461GTGCTCATAAATTTTCCGCTTAGTTAATAGTTAGTGACTCGTCCGTGAGTCAAATTATAT

134521CATAATTTACAGGATGAACAATCTTCAGCTTTTGGAGTTTCTATTTCATAATCATCAGTA

134581CCAGAACCATCACGGGTATTATGATAATAGAAATTTTTAATGCCATAATACCATCCGTAT

134641AGCATATCATCAATCATTATTGACATTGGAACCTTGCCTTTAGGGAAGATTTGGGGGTCA

134701TAATATGTATTCGCTGAAGCTGATTGACATACCCATTTCAGCATAATAGCTACCTGCGTA

134761AGATAAGGTTTATTACCTTTCTTAGCTAATTTCCATGTATAGTCGTAAAGGTCTATGTTA

134821TGCTCAATATTGGGCACGACTTGATTAAAGGAGCCCTCTTTTGATTCTTTAACAGAGACT

134881GGTCCACGTGGAGGCTCGATACCGTTTGTACTGTTAGAAACTTGGGAAGATGACTCACAT

134941GGCATAAGTGCTGATAATGTGCTATTACGGATGCCAAAGAGCTTAAGGTCTTCCCGCAGC

135001GACGACCAGTCACAAACGTATTTTGGAGCTGCGATTTGGTCAATCTTTTTATTGTACCAG

135061TCGATAGGTAATTCGCCTCGAGACCAACGAGTGTCTGAATAATATTCGCAAGGTCCTTTT

135121TCTTTGGCGAGCTTAATGGATGCTTTAATGAGTCCATACTGTAGTCTCTCAAATAGTTCA

135181TGTGTTAAATCGTTAGCATCTTCATAAGAAGCAAAATTACTTGCCAGCCAAGCTGCATAG

135241TTAGTAACACCTACGCCAAGGTTTCGACGTTTTTTAGCTTTTTCTGCTTCAGGAACTGGA

135301TATCCTTGGTAATCCAAAAGATTATCAAGAGCACGAACTTGGACTTCTGCCAATTCATTA

135361ATTTTATCTTGGTCTTGCCAATCGAAGTTATCTAACACAAATGCAGATAACGTACACAAT

135421CCAATTTCAGCATCAGGACTATTCACATCATTTGTTGGAATAGCAATTTCACAACACAAG

135481TTGCTCTGACGAATAGGTGCCTTTTCACGAATAAACGGAGTATAGTTATTCGTATTATCT

135541ATGAACTGTACATAAATCCTTGCTGTTCCTGAGCGTTCAGTCATGAGTAATTCAAACAGT

135601TCACGGGCTTTAATACGCTTTTTACGAATATTAGGGTCTTTTTCTGCGGCTTCGTATAAT

135661TCACGGAAACGGTCTTGGTCTTTAAAATAAGAATAATAAAGCTCGCCACCCATTTCATGC

135721GGACTGAACAAAGTAATGTAATCGTTCTTTCCAAAACGTTCCATCATCAAATCATTCAGC

135781TGAACACCATAATCCATATGACGAATGCGGTTTTCTTCTACGCCTTTGTTATTTTTCAAA

135841ACGAGAAGATTTTCAACTTCCAAATGCCAAATAGGATAATAAGCAGTAGCAGCGCCGCCA

135901CGAATTCCGCCCTGTGAACATGATTTAACAGCAGTCTGAAAATGTTTCCAAAAAGGAATA

135961ACACCAGTATGGCGTACTTCACCCATGCCAATCTTAGAACCTTCAGCACGAATCATACCA

136021ACGTTAATACCAATTCCAGCACGTTTGGAGATATATTCAACAATTGAAGCAGAAGCCTTA

136081TTAATAGACTTTAATGAATCACCTGCCTCAATAACAACGCATGAACTAAACTGCCGAGTT

136141GGAGTACGACAACCAGCCATAATAGGAGTTGGTAATGAAATCTGTCGAGTAGATACTGCT

136201TCATAAAAACGAATAACATGTTTTAATCTATCAACAGGTTCATCTTGATGCAATGCCATT

136261CCAATAGTCATAAATGCAAACTGTGGAGTTTCATAAATTTGACCAGTGGTTTTATCTTTA

136321ACTAAATATTTTTCTTTTAATTGCATCGCCCCGGAATAAGTAAATTCCATATCCCGCTCG

136381TGCTTAATTTTTGATTCTAAAAATGTAATTTCTTCTGCTGAATATTTTGACAACAGTTCA

136441GGGTCGTATTTACCTTCATTTACACAGTAAGAAATATGGTCAATAAATGAACGCGGTTCA

136501TACTGCCCATAAACATGCTTACGAAGAGCGAACATTAGACAACGTGCAGCTACATATTGA

136561TAATCAGGCTCTTCAACCGAAATAGAATTCGCAGCGGCCTTAATGACAATAGTCTGAATA

136621TCATCAGTTGTCATTCCATCACGGAGATAAGATTTAATATTTTCATATAATTCATAAGGA

136681TCTACTGATGTTCCCTCAGCTGCCCAAGATAAAACTTTAATAATTTTTTGTGGGTCAAAG

136741CTCTGAGAAACACCACTACTTTTGATAACATTAATTAATTGCATAAGTCCTCAACTTGAA

136801AATCGTCTTTAAACAATCGGTTAACTATATGAGCTATTATATCACCATGACACGGCTTTG

136861GTTTACATGTGCATCCTAGCCTCATTCCACGTAAAGGCTCTAAATGTGCTTTAGTTATTT

136921CTCCGGATTTAATTCGACGTATAAAATCTTTTTTGAATAATTCAATGGCAGCCTCCCGGC

136981TGCCAGCATCTTTACCGACGTAATTTCCCCAAAATGTACCACGGTGAATATTAACATCAA

137041AGTCGGATTTGTATTTATTCACTACCCGACATAGACGGCCCGCGCGGTGATAATTCGGCA

137101TATTGTTTTTCCGTTAAAACAGTAATATCGTAGTAACAGTCAGAAGAAGTTTTAACTGTG

137161GAAATTTTATTATCAAAATACTCACGAGTCATTTTATGAGTATAATATTTTTTGCCATAA

137221ATGATAATAGGCTGATTTGGTCCTGGAACTTCTAGCTCACTTGGATTAGGAAGTGTAAAA

137281AGAACTACACCAGAAGTATCTTTAAATCGTAAAATCATATTGCCATTTTCCCTTTAATTG

137341CTGGATGCGATTCATAATTTTCAAGGATAAAATCTTTCGGTTTCATGTATTCAGTTAACC

137401AGTAAAGCTGTGTTTCGGTGTCACAATTTCCAAACTCTATAGGCCAATCAATTTTAAGTT

137461CACATAGTTCTTTAGGCTCACGACGTAAAATTTCTTTACATTGCTCAGTATGATTCATAT

137521AGATGTGAGTATTACCACCAGAAAATATCAAATCTCCTGGGATAAGATTACACATCTTAG

137581CCACAATATGCGTCAAGGCAGCGTACGAGGCAATGTTGAATGGCAATCCCAAGAAAACAT

137641CTACACTTCTTTGGTACCATTGTAAATCAAGATAACCATTACGCACATTAAACTGATAGA

137701ACATATGACAGGGCGGTAATGCCATTTGATTGATTTCAGCTGGATTCCATGCTGAAACGA

137761TTTGACGTCGATCAGTTGGTAATTTTTTAATACGATCAATAGTTTCAACTAATTGATCCA

137821CACCGCCGAAATCACGCCATTGTTTACCGTATATAGGACCAAGTTCACCACTATGATATC

137881CTAGGTCTTTAGCCTGGTTTTCATAGTTATCGTCCCAAATAGTTTTACCTTGAATAAGAC

137941TACCATGTGTGCGAAGACGTAAATCATTAACATTGGTAGAACCGGAAATAAACCATAGAA

138001GCTCAGCAATGCATGCATTCCACGCAAGCTTCTTAGTTGTTACCGCAGGAAAACCTTTAG

138061TTAAATCCCAACGTAATTTAGTACCGAACAAAGCAATTGTTCCTGTGCCTGTTCGGTCAT

138121CGGTTTCATAGCCATTTTCAAAAATGTCTTTAATTAAATCTTGGTATTGTTTCATTTAGT

138181ACCTTATATAAATTATAATACATTTTATGAAGCTAAATAATCAATACGGTTTAGCATCAA

138241ATTTTCTCTCAATTTCAGGCGTGAAATATAATTCTTTACCGCGTTTGGAAAAGCGACCAA

138301TAATATGATAATTATTCTCATCAAACGTGATATTACCAATATCCATAGCTTTCAACCAAA

138361TATCGATATTTCTATCCAGCTTTATGTCAAAATTCCAATACGGAGAACTATTACCGAAAA

138421TACTGCAAGCGGATTCAGTGAAACAACTGCTATAAATCTTGTCTTTAGGAACTACTAATT

138481GATAGACACCTTTAATAAACTGAGGACCCTGCCACTTTGGCTGTTTCGTGTCTTTTTCTA

138541ATAGAATAGGGTAATAATTAAAATTCATATTTCCGCCACGCGTTATTTCATTTATATACT

138601GATTCCGTAAGGGTTGTTACTTCATCTATTTTATACCAATGCGTTTCAACCATTTCACGC

138661TTGCTTATATCATCAAGAAAACTTGCATCTAATTGAACCGTTGAATTAACACGATGCCTT

138721TTAACAATGCGGGAAACAACTACTTCATCCGCATAAGGTAACGCAGCATATAACAGAGCA

138781GGCCCGCCAATTACGCTTACTTTAGAATTCTGATCAAGCATAGTCTCGAATGGCGCGTTA

138841TGACTTGAAACTTGAATTTCGCCACCAGAAATGTAAGATATATATTGCTCCCAAGTAATA

138901TAGAAATGTGCTAAATCGCCGTCTTTAGTTACAGGATAATCACGCGCAAGGTCACACACC

138961ACAATATGACTACGACCAGGAAGTAATGTAGATAATGACTGGAACGTTTTAGCACCCATA

139021ATCATAATTGTACCTTCGGTACGAGTTTTAAAATTCTGGAGGTCCTTTTTAACTCGTCCC

139081CATGGTAAACCATCACCTAAACCGAATGCTAATTCATTAAAGCCTTCGACGGTTTTAGTT

139141GGAGAATAAGCGAATACCAATTTAATCATTACGCAAAGCCCCTTTCAATAAACCATTCAG

139201TGGCTTTATTAGCGTCAAAGAATAATTCTTCATATGTCTTTTCTTTGTTTTCAAAGACTG

139261TCACACAAACACGTTGACATTCTCCACAGTATTCTTCTGACATACTCAAAGCGTCAGAAA

139321ACATTTCATTAAATTCATTTAAATCAGGATTATGCAATGCGTTAAAAATTGCATAATCGA

139381ATTCATCATTCATAAATTCAAATACAAAAATCATATTATTTTCCTTAAACAGACTTTTTC

139441ACAATTTTCCAATCAGCTTTAAACTGATCAACATCAGAATGATAAATCCAGAATCCTGCG

139501CTTTCTCCGTCTTCATAAAGAGGACATCCATCACATTCATCTTCCCATCCCATGTCACGT

139561AAAAGATGTTCAGCTTTTTCAACAAGTTCAGAATCTTTACCGATGATATTAAAATACCAT

139621TTACCTCTAACTTCTGAATCTTTGATGCTCTGGCGTTGTAATCTCATTTTATTCTCCTTA

139681GCAAGCTTTAATCAAAAGATATAAACAGACCAACATAACTGCTGCCATAATATAAGGTGC

139741GAACATTTTCTTTTCTCCATTAGTTTTGATAGGGTAATAGTATCACACTACTACCCTTCT

139801GTAAACTACTTTTTGAAAGTTTTTCGCAAAAGTTCAATGATTTCATCTACATTGTTTTCG

139861TCAACAATGCAGTGAATTTTTGTTACGCCAGAAACTTTGTCTTTAACTTCATCTTCTTCA

139921GAAGTCGGTTCTTTATACTCACGAAAACAATGAAATTCGTCTTCACAAACGTTAAAGTAA

139981AAATGCTTTCCATTTGCGCATTCAATGTGTTTTATTACTCTAAATCCATCAACAAAGAAA

140041GCTTCTTTAACTTCAAACCATCCGCCATTTTCTTGAATAATTTTAACTATTGCTGAATTA

140101GAACGTGGGCGATAATTAATAAATGTATCAATAAGTCTTGGAACAAGTTCATACTTTTTA

140161CCGATATACATTATGTTTTCCTCATTTTAACGGGGCTGTAATAGCCCCTTGATAATTATT

140221GTTCAATCAATCCCATGTAAAATTCTGCGTCTTCAGAATCCATACCATCACAATATTCAT

140281TAGCCATAAAGCGGGTGAGGTCTTCAAGAGGACCTTCAATAACGATTTGAATACTCCAAA

140341ACTTAGAATCTTGCACGCTTGTGATACTAAGTTCAGGATAACGATTACGAATAATTTCTT

140401CGATATATTCAAAATCAACGATGTCAATATCAACTTTAGCCATATTATTTTCCTCTTTAA

140461TCATCAGCAGTATTGCCGATAGTTGTATAGTACCATGGAAGGACAAGGATGTAAACCGTT

140521TTGTGAAAAAATTTTTGAAATAAAAAGGGGACCTCTAGGGTCCCCAATTAATTAGTAATA

140581TAATCTATTAAAGGTCATTCAAAAGGTCATCTAGGTCCGTGTCATCAGCACTAGATGAAC

140641TGCCAGAGCTTGAGCTCATAAAATCATCTTCAGTTTTTGTATTGAAGTCATCAACATTGA

140701ATGCATCCAAATCATCAGCAACTTTATCAGCTTTCTTAGCAGCAGTTGCAGCAGCACCGC

140761CCATCACAGCAGTTCCCATAACTTGACCGAATTTAGTGCTCAGTTCTTCAAACGATTTGA

140821ATTTATCTTTAGAAGTCATTTCAGAAAGGTCAACCATTTGTTCGAACAGTTCTTTCTGGA

140881AAGATTCATCGTCAATGTTTGGAATCGCAGATTGATTCAGGAATTTAGATTCGTCGTAGT

140941TACTAAATCCAGAAACCTGTTTAACTTTCAGTACAAAGTTAGCACCTTCCCACGGACAAG

141001TTACATCAACCGGAGTTTCACCCATTTCAACATCAACCGCAATCATCGCGTTGATTTTAT

141061CCCAGATTTTCTTACCGAAACGATATTTAAATACTTTACCTTCATTTTCTGGAGCAGCTG

141121GGTCTTTTACAACAAGAATATTAGCCCAGTAAGAAGTTTTACGTTTAACAAGACTGTACT

141181CTTTATTGTCAGTGTTGTACAGATCATTTTTACTGATGTACTGACATACTGGACAAGAAT

141241CGTAATCACCGTGGGTAGATGAACAGTTTTCAATATACCATTTACCATTTTTCTTGAAAC

141301CGTGATTTACAAGAATTGCGAACGGTGCTTGTTCATCATTTTTAGACGGAAGAAAACGAA

141361TTACTGCTTGACCGTTACCCGCATTGTCGAGTTTCAGTTTCCACTCGCCTTTATCTTCAG

141421AAGAAAAACCACCTTTATTTCCAGCCAGTTTAGCCATTTGTGCAGCGAGTTCAGCAGTAG

141481ATTTACGTTTAAACATTTTTATTTCCTTTTTAATTTAATTAACAGTTGGTGCTATGACGA

141541TGTATGACCTCATAGCTGGTCAGTGAGATAATTATAATCTATTTATAATAAGCAATTAAT

141601ACTTGCAAGATTTCACAGTTTCAATGAAAACACTTTTAGCTTTCTGTGAATCAATATTTA

141661AAATTTTTCTATAAGCCTTTAACTTTATAGAATAATTATTCCAGACTAAATTATCAGTCT

141721GTTCATCATGTTTATCAATTATATTTAAAAACGAATCAAGCAAGATAAACGTCTCAAACG

141781AAATTATGTTCGATTGAAGTAGTTTAAAAATATAACTTGATTGAACTTTTGGATTATATT

141841CAAAAATTTCTTTAAAAGCAGAAACTTCAACTTTTTTACTGAAATAGTAAATGTTGCGAA

141901TATCTTCTTCAAACTTAAATTTAATTTGCTTCAAGCGTCCGATATATTCACGATAAAACA

141961CAAGTGCATCAGCGTCAGAGATGTCACCAATCCAAGCATCTTGGTTAGCTACAAGATTGC

142021TAATAAAAATTAAAGTTAATTCTTTTAATTTATACTTTTCTGATAGCTTTTGGAAAAAAT

142081ACTTATCCCTTCGCTTTTGATAAGCGGCATCAGACACCCGCATGCACCAATTATACTTAA

142141TTACATCATACTTTCCATTCATATGTTGTTTTATCATTAAGTATAATTTATAAACTGATT

142201TACCATCAATGTATCTTTCACCACCAGCAGGCATGCGGAGTTTAATCATAGTAGAAAATC

142261TAATGTATTAGTTTTTTCACAACGAACAACAGAAGGACGCAAAAGATTTTCGTCGATAGC

142321TTCTGACTGAATTTTTTCAATTATACCCGAAGGAATAAATTTAGCAAATTGAGTTTCAGG

142381AATAGAGTTTTCTTCTAAGAATGCTGTTGTAGCTTCAAGATAACTCATTCCAAACTCTTC

142441TACCATTTTTTCAATAATAAATCCATTTTCTTGACGATCAAGAAGCTTTGCTATTTCATC

142501CTTTTCTTTCTTGATTGAAAGTTCTTTTTCTGAAAGACCGGTCTCATCGACCGGACGAAT

142561GTCATTTAGAGAAAACTGTGTCATAAAGTTCAACTACCTCTTCAGTTTCAGCTTCAAACA

142621CATCACGGTTATCTTTATGATACAAAGCTAATAGACGATTAAACATCTTACCATCAACGC

142681CAAGTTCATCTTTGGCACGAATTCGAATATCTTTAATCAGTTCATTATAACCGGAAATTT

142741TCAGTTTATGATCAGATGCTTCTTTAATAAATTTAGCCAAGTCTTCGCCATGGATAGCTT

142801CATCAAATTCAACCATTTCTTTTTTAGCCATTATTCACCTCAAAATTCATTAATGCTATT

142861AGTTAATTTAGAAAGACCCGCTTTTACAAAATATGAATAAATTTTGCCACGCGGTGGTAA

142921TTTATATGAATTATAGTAATTCACAATGTTTGAAGCAATATTATCAGGAATATAATCAAA

142981ATCAATTAGAACTAAATTTTCTTTATAACGATTATATTCAGATTCTGTGAGAAGCACCTT

143041AGCTTGCTCACGGTCATTAGCAATAGCTTCAACGATTGAAGTTTTCATTGAAGGGGTTCG

143101TTCACCTTCAACTCTGGTAAACCAAAAGTCAGATCGTACTTTAACTGAAGCAACGTTATC

143161CTTTTTGTCGCCTTTAAGGATTTTAGTCATACAGTCAATTTCAGCAGAACCGCTTTTAAT

143221TTTAACCCATTTCTTATGCATCGGTGACCATTGCTTAACATTTGGATATTTGTGAAGCTG

143281TGTAAAGTCACCGTCCGATGAAATGATTAAAATCTTATGTCCTTCTAAAGAGAACTTTTT

143341AACAAGAACAGCAATGTGGTCATCTGCTTCATACTTATCAATATCCATAACAATGTATGG

143401CATATAAGCTTTCAATTCATCTATAACTTTATGACTGGATTCAAAATAACCTTCCCAGTC

143461CCAAGTAGATTCTTCTCGTGCTTTTCCACGGTTTTTCTTGTAATAGTAAGCGAAATCACG

143521ACGCCAGTATCCAGATTTCGCGTTATCAATACATAGTACAATTTTAGTGTATCCAAGCGT

143581TTTTGCTTTTTTGACATTAAACTTAATTGAGTTCAATATCAAATGACGAACCATCGATAA

143641ATTAATTTTTTCTTTATCTGGGAAGTTTACCAGAGCAGTTGAAAGAGCAATTTGACTAAA

143701GTCAATAAAGCAAATTCCTTCTTTGTAATCTTCATCCAGCATCATTTCTAAATCCATATG

143761AACCTCGTTCAATTAGTGAGATTTCTATTATATACTATCTAAATCTTAAAGTAAACAGGT

143821ATAAATACTTATTATTGAAAACACAATAGGAGCCCGGGAGAATGGCCGAGATTAAAAGAA

143881AGTTCAGAGCAGAAGATGGTCTGGACGCAGGTGGCGATAAAATAATCAACGTAGCTTTAG

143941CTGACCGTACCGTAGGAACTGACGGTGTTAACGTTGATTACTTAATTCAAGAAAACACAG

144001TTCAGCAATATGATCCAACTCGTGGATATTTAAAAGATTTTGTAATCATTTATAATAATC

144061GTTTTTGGGCAGCAACGGATAATATTCCAAAACCTGCTGGAAATTTTAATAGAATTCGTT

144121GGAAAGCATTACGTACTGATGCCGTATATACAACCGTATCATCTGGACCATATCAATTAA

144181AATCCGGAGAAGCAATTTCAGTAGATACATCAGTTGGCAATGACATTGAGTTTACTTTAC

144241CACCTTCTCCGCTTGATGGAGAAACCGTAATAATTCAAGATATCGGTGGAAAACCTGGCA

144301TAAATCAGGTTAAAATAAATTCTTCAAATCAGAGTATTGTCAATTTTAGAGGTGAACAGG

144361TACGTTCAGTTTTAATGACTCATCCAAAGTCACAGATGATATTCATTTTTAATAATCGTT

144421TGTGGCAAATGTATGTTGCTGATTATAGCAGAGAAGCTGCGATTGTTACTCCATCGACTG

144481CGTATCAAGCACAATCTAATGATTTTATCGTACGTAGATTTACTTCTGCTGCACCAATTA

144541ATGTTAAACTTCCGAGATTTGCTAATCATGGCGATATTATTAATTTCGTTGATTTAGATA

144601AACTGAATCCACTTTATCATACAATTGTTACTACATACGATGAAACGACTTCAGTACAAG

144661AAGTTGGAACTCATTCCATTGAAGGCCGTACATCGATTGACGGTTTCTTGATGTTTGACG

144721ATAATGAGAAATTGTGGAGATTGTTTGACGGGGATAGTAAAGCACGTTTACGCATTATAA

144781CAACTAATTCAAATATTCGTCCAAATGAAGAAGTCATGGTATTTGGCGCGAATAATGGAA

144841CAACCCAAACAATTGAACTTCAGCTTCCGACTGATATTTCTGTTGGTGATACTGTTAAAA

144901TTTCCATGAATTACATGAGAAAAGGACAAACAGTTAAAATCAAAGCTGCTGGTGAAGATA

144961AAATTGCTTCTTCAGTTCAATTGCTGCAATTCCCAAAACGTTCAGAATATCCGCCTGAAG

145021CTGAATGGGTAACAGTTCAAGAATTAGTTTTTAATGGTGAAACTAATTATGTACCAGTTT

145081TGCAACTTGCTTATATAGAAGATTCTGATGGAAAATACTGGGTTGTACAGCAAAACGTTC

145141CAACAGTTGAAAGAGTCGATTCTTTAAATAATTCTACTAGAGCAAGATTAGGCGTAATTG

145201CTTTAGCTACACAAGCTCAAGCAAATGCTGATTTAGAAAATTCTCCACAAAAAGAATTAG

145261CAATTACTCCAGAAACGTTAGCTAATCGTACTGCTACAGAAACTCGCAGAGGTATTGCAA

145321GAATAGCAACTACTGCTCAAGTAAATCAGAACACTACATTCTCTTTTGCAGATGACCTTA

145381TCATCACTCCTAAAAAGCTGAATGAAAGAACTGCTACAGAAACTCGCAGAGGTGTTGCTG

145441AAATTGCTACGCAGCAGGAAACTAATACAGGTATCGATGATACTACAATCATCACTCCTA

145501AAAAGCTTCAAGCTCGTCAAGGTTCTGAATCATTATCTGGTATTGTAACTTTTGTATCTA

145561CCGCAGGAGCTACTCCAGCTTCTAGTCGTGAATTAAATGGTACGAATGTTTATAATAAAA

145621ACACTAATAATTTAGTTGTTTCACCTAAAGCTTTGGATCAGTATAAAGCTACTCCAACGC

145681AACAAGGCGCAGTAATTTTAGCAGTTGAAAGTGAAGTAATTGCTGGACAAAGCCAAGAAG

145741GATGGGCAAATGCGGTTGTAACGCCAGAAACGTTACATAAAAAGACATCAACTGATGCAA

145801GAATTGGTTTAATTGAAATTGCTACACAAAGTGAAGTTAATACAGGAACTGATTATACTC

145861GTGCAGTCACTCCTAAAACTTTAAATGACCGTAGAGCAACTGAAAGTTTAAGTGGTATAG

145921CTGAAATTGCTACACAGGTTGAATTCGACGCAGGCGTCGACGATACTCGTATCTCTACAC

145981CATTAAAAATTAAAACCAGATTTAATAGTACTGATCGTACTTCTGTTGTTGCTCTATCTG

146041GATTAGTTGAATCAGGAACTCTCTGGGACCATTATACCCTTAATATTCTTGAAGCAAATG

146101AGACACAGCGTGGTACACTTCGTGTAGCTACACAAGTTGAAGCTGCTGCAGGAACATTAG

146161ATAATGTTTTAATAACTCCTAAAAAGCTTTTAGGTACTAAATCTACCGAATCGCAAGAAG

146221GTGTTATTAAAGTTGCAACTCAGTCTGAAACTGTGACTGGAACGTCAGCAAATACTGCTG

146281TATCTCCAAAAAATTTAAAATGGATTGTGCAGAGTGAACCAACTTGGGCAGCAACTACTC

146341TGATAAGAGGGTTTGTTAAAACTTCTTCTGGTTCATTAACGTTTGTTGGTAATGATACGG

146401TAGGTTCAACACAGCCATTAGAATCATATGAGAAAAATGGTTATGCAGTATCACCATATG

146461AATTAAATCGCGTATTAGCAAATTATTTGCCATTAAAAGCAAAAGCTGTAGATAGTAATT

146521TATTAGATGGTCTAGATTCGCTCCAGTTCATTCGTAGGGACATTGCACAAACAGTTAATG

146581GTTCACTAACCTTAACCCAACAAACGAATCTGGGTGCCCCTCTTGTATCATCTAGTACTG

146641CTACATTCGGTGGATCAGTTTCAGCAAATAGTACATTAACTATTTCTAATACTGGAACGG

146701CAACTCGTCTGATTTTTGAGAAAGGACCTCAAACTGGAACAAACCCGGCTCAAACGATGA

146761CAGTCAGAGTGTGGGGAAATCAATTTAGCGGGGAATCAGACACAACACGTTCTACCGTAT

146821TTGAAGTTAGTGATGAAACGTCTAGTCATTTTTATTCTCAGCGTAATAAAGCTGGAAATA

146881TAACATTTAATATCAACGGTACAGTAACACCGATAAATGTTAATGCTTCAGGAACATTGA

146941ATGCAAATGGTGTAGCAACATTTGGTAATTCAGTCACTGCAACTGGTGAAATTATTTCTC

147001GAAGCGCAAATGCTTTCCGTGCTATTAACGGAAATTATGGTTTCATTGTTCGCAATGATG

147061GATCAGTAACGAATTTTATGCTTACTGCATCGGGTGATCAGACTGGTGGATTTAATGGAT

147121TACGTCCTTTAGCTATTAATAATGCATCTGGCCAAGTAACGATTGGTGAAAGCTTAATCA

147181TTGCCAAAGGTGCTACTATAAATTCAGGCGGTTTGACTGTTAACTCGAGAATTCGTTCTC

147241AGGGCACTAAAACATCTGATTTATACACCCGCGCTCCAACATCTGATACTGTAGGATTCT

147301GGTCAATTGATATTAACGATTCAGCCACTTATAACCAGTTCCCGGGTTATTTTAAAATGG

147361TTGAAAAAACTAATGAAGTGACTGGGCTTCCATACTTAGAACGTGGCGAAGAAGTTAAAT

147421CTCCTGGTACATTGACTCAGTTTGGTAACACACTTGATTCGCTTTACCAAGATTGGATTA

147481CTTATCCAACGACCCCAGAAGCACGTACCACTCGCTGGACGCGTACATGGCAGAAAACTA

147541AAAATTCTTGGTCAAGTTTTGTTCAGGTATTTGATGGAGGTAACCCTCCTCAACCTTCGG

147601ATATAGGAGCAATCCCATCTGATAATGGAATAATAGGTAATCTTACTATTCGTGATTTCT

147661TACGAATTGGTAATGTTCGCATTATTCCTGACCCAGTGAATAAAACTGTTAAATTTGAAT

147721GGGTTGAATAAGAGGTATTATGGAAAAATTTATGGCAGAGTTTGGACAAGGATACGTCCA

147781AACGCCATTTTTATCGGAAAGTAATTCAGTAAGATACAAAATAAGCATAGCGGGTTCTTG

147841CCCGCTTTCTACTGCGGGGCCATATGTTAAATTTCAGGATAATCCCGTTGGAAATCAAAC

147901ATTTAGTGCAGGTCTTCATTTAAGAGTTTTTGACCCTTCTACGGGAGCATTAGTTGATAG

147961CAAGTCATATGCTTTTTCTGCTTCAAACAATACAACATCTGCCGCTTTTGTCAGTTTCAT

148021GAATTCTTTGTCAAACAATAGACTTGTTGCTATATTAACTAGCGGAAAGGTTAATTTTCC

148081TCCTGAAGTGGTATCTTGGTTAAGGGGAGCAGGAACTTCAGTTTTTCCATCAGATTCAGT

148141ATTGTCAAGATTTGACGTATCATATGCTGCTTTTTATACTTCTTCTAAAAGAGCTATTGC

148201ATTAGAGCATGTTAAACTAAGTAATAGAAAAAGCACAGATGATTATCAAACTATTTTAGA

148261TGTTGTATTTGATAGTTTAGAAGACGTCGGAGCGACAGGATTTCCAAAAAGAACTTATGA

148321AAGTGTAGAGCAATTCATGTCAGCAGTTGGTGGAACTAATAACGAAATTGCGCGATTGCC

148381AACTTCAGCTGCTATAAGTAAACTTTCTGACTATAATTTAATTCCTGGTGATGTTCTTTA

148441TCTTAAAGCACAGTTATATGCTGATGCTGATTTACTTGATCTTGGAACTACAAACATATC

148501CATTCGTTTTTATGATGCATCAAATGGATATATTTCCTCGACCCAAGCTGAGTTTACTGG

148561GCAAGCTGGGTCTTGGGAATTAAAAGAAGACTATGTAGTTGTTCCAGAAAACGCCGTAGG

148621ATTTACGATATATGCACAAAGAACTGCTCAAGCAGGTCAAGGCGGCATGAGAAATTTAAG

148681TTTTTCTGAAGTATCAAGAAATGGCGGCATTTCAAAACCTGCCGAATTTGGCGTCAACGG

148741TATTCGCGTTAATTATGTCTGTGAATCGGCTTCACCTCCAGATATAATGGTACTTCCTAC

148801ACAAGCCTCTTCTAAAACTGGCAAAGTGTTTGGACAGGAATTTAGAGAAGTTTAAACTGA

148861GGGAGCCTTCGGGTTCCCTTTTTCTTTATAAATAATATTAAAATAAAGGGGCATATAATG

148921GCTGATTTAAAAGTAGGTTCAACTGTGGGTGGATCTGTCATTTGGCATCAAGGAAATTTT

148981CCATTGAATTCAGCCGGTGACGATGTACTCTACAAATCATTTAAAATATATTCAGAATAT

149041AATAAACCACAGGCAGCTGATAACGATTTCGTTTCTAAAGCTAATGGTGGTACTTACGCC

149101GGTCCAATTACTATTAATTACGGGGTAAATAGTTATCTTCAATTAAGTAATAATGAAACC

149161CCTATCCGAATTCGTTCTGGTGGCGGCACTGGTAATACTCTTGTAGTTGGCGGCTCTTCC

149221GGTGGTATTAGTTTTAGACCTGCAGGTAGTGAAATCACTACTGGACAAATTACTATTACG

149281CCAGAAGGTTTGACAATATTTACCAGGGCTGTAACGGCTCCATCGGTAACTGTTACATCT

149341ACTCCTTCCGCAGCATCTGATGTTACTCGCAAAGATTATGTTGATGGAGCAATAAATACT

149401GTTACAGCAAATGCAAACTCTAGGGTATTACGCTCTGGAGACACTATGATAGGAAATTTA

149461ACTGCGCCAAACCTTTTTTCACAGAATCCTGCATCTCAACCTTCACACGTTCCACGATTT

149521GACCAAATCGTAATTAAGGATTCTGTTCAAGATTTCGGCTATTATTAAGAGGACTTATGG

149581CTACTTTAAAACAAATACAATTTAAAAGAAGTAAAACTGCAGGTCAACGTCCTGCTGCTT

149641CAGTATTAGCCGAAGGTGAATTGGCTATAAACTTAAAAGATAAAACAATTTTCACAAAAG

149701ATGACTCAGGTAGTGTTATAGAATTAGGTTTAAAATATGGAGGAACAATTGATGGTTCTT

149761TAACTGTTAATGGAAACATAATTGGAAATTTAACAGGTAACGCTGCAACTGCAACGAAAT

149821TAAAAACAGCACGAAAAATTAATGGTATATCCTTTGATGGGTCAAAAGATATCACGCTAA

149881CTCCATCTGACATAAATGTCAATAGCACAACGTTTATAAAAAATAACGGCGAATTACCTG

149941TTGATGCTAATTTAGATGAGTATGGGCCTGTTGAAGAATATCTTGGAGTTTGGTCGAAAG

150001CAACTTCAACCAACGCTCAACCAGCAAATAAATTTCCAGAAGAAAATGCTGTAGGTGTTC

150061TAGAAGTATTTGTGGCCGGTCAATTTGCTGGTACTCAGAGATATACAACTAGATACGGAA

150121ATGTTTATATTCGTTCCTTGACTGCTACATGGAACGGAGTAAACGGTCCGTGGAGTGCGT

150181GGCGAAATATTCAATCTGGTACTCGTCCACTGTCAACAACGATTGATCTTAATGATCTAG

150241GAGGCGCTGAACACCTTGGTTTGTGGCGAAATAGTTCAAATTCCATTGCTACCTTTGACA

150301GAAATTTCCCAGAAGAAGGATCGTCCGCTCAAGGACTTTTAGAAGTATATGAAGGTGGAA

150361ACTATTCTCGCACACAGAGATATACGACCAGATTTGGTGTTGTTTATACTCGTTGTCTTG

150421CTGCTGCGTGGGATGCTTCTGCGCCTAAATGGGGACCTTGGCAACAAGTCGGTAATGTCA

150481CACCGGCGACTTTCTATGACGGAGATCTGAATGATTTTAAAACTCCTGGGTTATATAATA

150541TTTTAGGCACTGATGCCGTTATTAACTGTCCTACCGGTGAAGGTTTACCAGCCGTTATTG

150601TTGGTTTGCTGGAAGTTAAACAGCGTGCTTCTGGCGGTGCTATTTTCCAAAAATTTACTA

150661CTGCCGGAACGGGTGCAACTACTCGCGATCGTATTTTTGAGCGTGCATATACTGGTGGTG

150721TGTGGGGTGCATGGAACGAAGTATATACATCTTATTCCCTGCCAATTACTTTGGGTATGG

150781GTGGTATTAAAACTCAATTAGCGGAGTTAGATTGGCAAACCTTTGATTTTGTCCCTGGTA

150841GTATGTTTAGTGTTCCTTTGAACAAAATAAAGAATATGCCAGCAAATATGGATTGGGGCA

150901CGATTGATGGAAACCTGGTTATGTTTTCCGTTGGTCCTAGCGAACACACTAGCACAGGAC

150961GTACTGTTCAGATTTGGCGCGGCACCGTATCCCAGACAAACTACCGTTATTTTGTCGTTC

151021GTGTGTTCGGTAATTCTGGAAATAGAACTTGCACAGTTCGTCGTGTTGTTCTTGAAGACG

151081GATCACATACTTGGACTGCTAAACAAGATTTTAATGGCGCAGTTAACTTTAACTCTACTG

151141TTAACCTTAATAACACTACCACATTTAAAACAGAAGTTAAATTTCGCTCATTGAATGCAT

151201TCCGTATGTATGGCGGAAAATTTGGTACATTTTTACGTAATGATGGAGAGAGTCTTTATA

151261TTCTTTCCACCGACGAAGATGATCAAGATGGAAACTTTAATACAAATAGACCTTTCCGTT

151321ATGAATTAAGAACTGGTGATGTTACTTTGGGTGGTGCTAGTGGTGCTAACGTTTTAAAAT

151381TAAAACGTGATTCTCTCACCGCATTTTTTGGCGGTGATATTAACATTAAAGGCATGATGA

151441CTTTTGACGCCGGACGTTTAGGATCACGAGATTATTTTAAATTTAACCATTGGGGTGATA

151501GTAATAATGCGCGTGATAACATTATTCAGTTAGAAGACAGCAAAGGCGCTCATTTTTCCA

151561CTGAACGTACTTTAGCGACTGGTGCAATTAAGACTAAATTTTTTGGTGAAATTGAATCCG

151621ATGGTAAATTGGTTATTAAACGTCCGGGTGATTCTATTGTATTATCAACAACTGCTAGTA

151681ATTCTTTGCATATTCGCGGTGATATAGACGGGACTGGTAACTGGTATATTGGTAAAGGTG

151741GTGCTGATAATGGATTAGCGTTCTATAGTTATGCTACTAATGCTGGTGTATACATTACAA

151801ACGCAGGAGATATCTCGCTAAGTCCAAAGGGTGCCGAAATGGCTCAGGTCAATAACGTTC

151861GATTATATGTTCATGGTGAACGTTGGACCGCTAGTCAACCAGGTGATTGGGGCAGTCAGT

151921GGCAAGTGGAAGCGCCAATATTCGTCGATCATGGTTATGTTTCACAGGATTGTTATTATC

151981CAATTATTAAAGGAAGAAGTGTAATCACCAATCAAGGGTTTGTAACTGCCGTCGATCTTG

152041GTATTCGTCGTGTCCCTAACAATTGGGGGCAAGCAATTATTCGTGTTGGATCTGCAGAGG

152101CATCGCCAGCGGCTGGACACCCTAACGCGATATTTGAATTTCATTACGACGGTACTTTCT

152161ATTCTCCTGGTAATGGTAACTTTAACGATGTGTATATTCGTTCCGATGGTCGTCTTAAGA

152221TTAATAAAAAAGAGCTAGAAAACGGAGCACTTGAAAAAGTATGCCGACTGAAAGTTTATA

152281CATACGATAAGGTTAAGTCTATTAAAGATCGTAGTGTTATTAAACGTGAAGTTGGTATTA

152341TTGCTCAGGATCTTGAAAAAGAATTACCGGAAGCTGTGTCTAAAGTTGAAGTTGATGGAT

152401CTGATGTTCTGACAATTTCTAACTCCGCTGTGAATGCTCTTTTAATTAAGGCTATCCAAG

152461AAATGAGCGAAGAAATTAAAGAATTAAAAACGCCTTTCTTCACTAAAATTGCTCGCAAAA

152521TTAGTAAATATTTTAAATTCTAACAACAAGGGGCAACGCCCCCTTTGGAGATAAATTATG

152581GCAGTAGTTGGTGTTCCTGGTTGGATTGGTAGTTCATCCGTAAATGAAACAGGACAACGA

152641TGGATGAGTCAAGCGGCTGGTCAATTAAGATTGGGTGTTCCTTGCTGGATGAGTCAATTT

152701GCAGGTCGCTCACGAGAAATTATTCATACACTTGGAGCAGACCATAACTTCAATGGTCAA

152761TGGTTCCGAGATAGATGTTTTGAGGCCGGTAGTGCACCTATAGTGTTTAATATTACAGGT

152821GATTTAGTATCATATTCTAAAGATGTTCCTTTATTCTTCATGTACGGGGATACGCCTAAT

152881GAATATGTTGTTCTTAATATTCATGGTGGTGTTCATATGTGGGGTCGTGGTGGTAATGGT

152941GGATACACTCACTCGGGAGGCGACGGTAACGGTACACAAGGCGGTCATGTTATTCAAAAT

153001GATATCGGTGGACGGCTTCGTATTTGGAACTACGGTGTTATAGCTGCTGGCGGTGGCGGC

153061GGTGGTGGTATTGCATATCGTCCACACTCAGGGGCAAACTGGCAAGATATCGGTGGCGGT

153121GGTGGTCGACCTTTCGGTGGCGCTGGCGGTGGCGGTTATTCCGGTGGTGCTGCTTCGTAT

153181GAAGGTCCGGGTGGTGGTTATGACTATGGTAACGCACACTCCGGCGCAGGTGGTAATGCT

153241GGTGCTGCTGGTCAGAATGCATGGTCTGACGGCGGTAAAGTTCTTAAAGTTGGTGTTGGT

153301GGTGCGTCTGGTCATGCAGTGTTTGGGTCTTCTCCAACTTGGGGTGCTGTTGGAACAATT

153361TACGGACCAAGAGTATAATTTGAATAAATATCCTTAAAAGGAGGGTCTATGGCAGCACCT

153421AGAATATCATTTTCGCCCTCTGATATTCTGTTTGGTGTTCTAGATCGCTTGTTCAAAGAT

153481AACGCTACCGGGAAGGTTCTTGCTTCCCGAGTAGCTGTCGTAATTCTTTTATTTATGATG

153541GCGATTGTTTGGTATAGGGGAGATAGTTTCTTTGAGTACTATAAGCAATCAAAGTATGAA

153601ACATACAGTGAAATTATTGAAAAGGAAAGAAATGCACGCTTTGAATCTGTCGCCCTGGAA

153661CAACTCCAGATAGTTCATATATCATCTGAGGCAGACTTTAGTGCGGTGTATTCTTTCCGC

153721CCTAAAAACTTAAACTATTTTGTTGATATTATAGCATATGAAGGAAAATTACCTTCAACA

153781ATAAGTGAAAAATCACTTGGAGGATATCCTGTTGATAAAACTATGGATGAATATACAGTT

153841CATTTAAATGGACGTCATTATTATTCCGACTCAAAATTTGCTTTTTTACCAACTAAAAAG

153901CCTACTCCCGAAATAAACTACATGTACAGTTGTCCATATTTTAATTTGGATAATATCTAT

153961GCTGGAACAATAACCATGTACTGGTATAGAAATGATCATATAAGTAATGACCGCCTTGAA

154021TCAATATGTGCTCAGGCGGCCAGAATATTAGGAAGGGCTAAATAATTATTTGTTCGTATA

154081CATCTCTAGATATCGATATACACCCTCAAAACCCTCGTTGAATTCGTCGATGAGGGTTTT

154141CTTATCTTCTTGAGTTAATTCAGAAACAATTTTACGGAATGAATTCTGATTTAACTTTCT

154201ACCTTCATGTGTTACTCCAATCTCATTCAGAAATGCAATAAAATTAGCACGATTTTCAAC

154261AATATCTTCTCTGGAAAATTTAATCAAAATAGACGCAACAGTAATAATTTCACGAACTGT

154321ATCAATGTTTTTATTCATTAACTATACCACTCAATTAGTTGACTTTGTTATAATATCATC

154381AGACACTTGATTTGTAAACTGGTCTGTGTTATTTTCTTCAAAAATTTTTTCTACGAATTC

154441CTTGAACGACTCGCGTTCCTGAGCTACATTATGCTCGATTACCATTTCAAGATTATGACT

154501CATTCGAAATAATCTTCAATTTCGTAATCATGGACATAAATCATTATAGTTTTTAATACA

154561TCATCAATACTTTTTCCTGGAGCTGGAATTACGTAAAAATATCCTGCTTTTGAGAGGTCT

154621TTATAAGTTCCAACCAAGAAATCATTATTCTCGAGATGTAATTCTTCAACTAATTCATTA

154681ACAATTGAATGGTATAAATTTGGTAGAAACTTATATAGCTTTTCTAGAATATCAATTTTG

154741ATTGTATATTGAACCACAGATTGAGAATCAATAATCATAGACCTTCCCCTTATGTTTCTG

154801TTTGCGATTAGATTCTTTAAACGCTTTCTTCTTATCCTTATGAACAGAAGCTTTATTAAA

154861ATTATGTTTTGCGACTAAATTGTTCATAGTGCTGAATTACCTCTCTCAAACATTTGCATG

154921TGAATGAAAACTTTTTAGCTACACCACATTCAAATATATATTCTCTTAAATCACGTGTGT

154981CAGTATATCCCATCTCAACAATAAAATGTCGTATTAGATTTTTATCTTTATCATTGAGAG

155041AATTAAAATAATCGGATTTTGAATTAATTTCCCTGGCCAAATTGAATCACCTTCAGTTGA

155101CGTTTTAGTTCTTTTATCATTTCTTCATTCATCGCAATATAAAGATCGCGTAAAGCGGGT

155161TTTAACATTCCATTTACTGGAGAACTAAATGGACATACATAATCTTTTCCTACGAGCTTT

155221TTAGTGAATTCCATATTACAGAACTGAAATGCCGGTTCATTAGCATAAATTCCCCAATTG

155281GTTGACATCATTTTATTGGCATATTCCAATGCTTGGATTTGATTAGTAATTCCATCAATT

155341TGAAACTTTTAATATTCATTAGTAAAGGTCCTCGGAGTAAAGTTCTTTTTCACTACCACC

155401ACGTTCAATACGCACTTGTCCAGCGTAAGTTGCAATAATCATTGCTTCTTCACGTGTCCA

155461GTAATTGCTATACTGGTCAATAAATCCTTGGTCTTCACCACAAACATGGTCTGATACAAG

155521TTTATCACTTACCTGGTCAAGAACTTCAGCCATATCTTTAGAATAATGACGAGCACCAGG

155581AATAACCAGAGTCCCACCATCTTTTAACTTAAAGCGGTTGGCTGCACACACAATTCGACG

155641TTGATATTTTTCATTATTGTTCCAATGAGCTACTTGCCAACAGATTTCAGGAACCTCTTC

155701TAAAACATCCTCTTCCGTATATTCGGTGTAGTCACCATAGGCCTGTAATTTAGCTGCTAG

155761ACTTTCTGGAGTTTCACGTAATAAAGCCAGGTCTAATAATTCAAGACGCTCTTTGAAGGT

155821TTTCATTTGGTTTCCTCAACACTTTTAATTTTTATAGCTTGTTTAGAACTTTCAAAGCAT

155881TGACAATATACTCTTACCGCATCAAACTGGTTGGCCGCTTTAAGATGGACTACACCTTCG

155941CCGTTATAAAATTCTACGACAATTTTAAATGTTTTCATTTAAACCATCCTTTAATACGTT

156001GCCATAAAGTTTTCTGTTGAGCTTTGTTAACACCAATTGAGCGAATAACTGGTTGAGATT

156061CATGGAATTCTTTATAATCAGCAAGGTAAATTTCGTAAGCTGAATCCATAAAGGAACTTA

156121TAGCTGTCATGAAATTATTGCGAATACCTACTGGAGCATCTTTACTTTCACGAATAATCA

156181TGTATTTACCAGTCTTAATCTTTACGATAGTTCCAAGATAAGCTCCATGGTACCAGATGT

156241CCCAACCCTCTTGAGTAGGTTCTACACAACGACGAAGTTCATTGACAATTTCTAACTTGT

156301TCATTATTTATTCCTCACAGTTCAGATGCTACAGTGATTACAGCTTCAATGTTTTCTGCC

156361GAGCGTTTAATGTCAAGATACACATTACCGTTTTTAGCGATTTTACATGACATTCCGATA

156421TCAGTAAATTTCTGAATATGATGTTCCATCATTTTGTATCCAAAAATTCGCATATTTCCA

156481TTATTGTTAATTTCAAAATTACGAATTCCGTGAGTGCGTTTTTCTAAAATGGCAAGATAA

156541TTACTACGATAAGTTTCAACCTTTTTAAGAACAAATCCATTTTCATCTAAAAGTTTTAAC

156601ATGAGGTCTTTATCTTCTTCCATATCAGAAGTAATCTCACGAGCTTTACGAGTTGCTCGT

156661TTTTTCAGTAGTTCCGGAGCATTTTCCTGCGCGTATAAAGTTGCTGCGTTTGAAATAATA

156721TCTTGAGCTTCACCAGTAATGATTAATCCATCACCAGATTTCTCCACCAGGCCTTTTTTA

156781ATCAATACCCCAATATTACTATTAACTACTGCGTTACCTAAATCTGGATGCACCTCACGA

156841ACTTCTGCGGCTGTAATGAAATCTTTCTTAGCAATGGTAATTAAAATCGCAGCAGTTTTT

156901TCATTCAGAACATCGTTAGAAGCTTTGATGATGTAAGTTACTTTAGACATTTTAATCTCC

156961GTTTCAGTTCATTTATTTGATAGGTCTATAGTATCATGTTTAAAGCAGAAGTAAACACTT

157021TTTTGCACTCTTTTTCAAGAGCTCTAATAAAGTACTTACTTAATGTTCAGATTACGAACT

157081TCGATTTCGTCTTCATGGGCAAAATCAAACTGTTTGAATTCTGTGTCACCTTTAAATTTA

157141AAAATCATTGTGAAGAAAGTCAGAACACCAGGTTTTTCTTCACGAACTCCACAGATATCA

157201TATTCAATACCATCTACAACCAGTTTGTTAGCAGTCAGAGCATTTTTAGCTTTAACGATT

157261TCGTTTTCGATTTTAAATTTCATTTTTTGTATCTCCGGGTTGTTTGTTTCAGTTCATTTA

157321TTTGATAGTTGTATAGTACCACAGTATGCTTTGGTTGTAAACCGTTTTGTGAAAAAAATT

157381TTTGAAATAAAAAGGGAGAGCCGAGGCTCTCCCTAAAATTACTGCATGACTGTGATAACT

157441GTCATGATAACACGTTGAATTCCGAACGCAAGAAGACCTCCTGCTACGGCAGGAACAACA

157501CCTAAACCCGCCAGTAAAATGCCACCAGATACTAATGCAGCGCTTGTGATACCAATGAAT

157561GGACTCATTTGATTTCCTCTAAATCTTTGGTGTATTCTGTAACTACATCAGTAGTTTTCC

157621AATATTCGTTTTCTTCTTTTTTGGCTTTAGCTTCTTCAGCAAGTTTCTTTGCTTCGTCGG

157681AAGTCATATGAAAAATGTTCATTCCAACTAGTTTATCAACATAAGAAGAATACATATCAA

157741TTTTAGAAAGTTCTTCGGTCAGTTCTTTGCGAGTTTTACCTTGTACAACAATTTCACCTG

157801AAATTACTTTCTTAATGAAATGTGCCTTGGCAAAAGCTAAACGAAATGCTGATTCAGTTT

157861CTTTGATTTTGTTATCAATTCGTTTTTGGACATAAGTTTTACGAACTTCAACGAAGTCTT

157921TGATTAAATCAACTACATTATCGTAAACTTGCAGCTTTCCTTTCTCATTAATGACGGTAA

157981TATTCTGGGAACGACGCTCAATCAACCCGAAGTCTTTCATAATTTTTGCATGGCGTTCTT

158041CTTCATTATCGCTCAAAGAGTATTCTTTTCGGAATTTAACTTTGAATCCAAAACCATGCT

158101CACCACAAGCATCATCCCATGTAATGAAGCCTTTATCTTCAAGCGGGTCTAAGATTTTAC

158161TCACATAAGTTTCACGATCATACTTATATGGAATCTCAGTGATATGCATTTGAGTTCGTG

158221AAGTAAACTTATATGTTCCACGAATTTCATATTGCCCATCAATTTCAACGACTTCACCAC

158281GAAATTCTGGGAATTCTACCTTCGGTTTAGTTACTTTCTTTCCTTGAAGAGCTTGCAGTA

158341CAGCTTTCTTGACAGAAGAAACACTATGAGGAAGAATGTAAGTTGCATAACCAGTTGCAA

158401TACCGGAAACGCCATTAAGAAGAACAGTAGGAATAATAGGCAAATAGAAAGCAGGCGGAA

158461TGTGTTCTTTATCTTGATGTACTGGAGCATATTCAGTATCTTTATATACGTTATAGAAAT

158521TTTTACTTACACGAGCAAAAATATAACGACTTGCTGCTGCCTTTTGGACAGTACGAGAAC

158581CAAAGTTTCCTTGACCGTCTAACAGAGGAAAGTTATTATTCCAAGTGTTAGCCATCAAAG

158641CACCTGCGTCTTGCGCAGAGTTTTCACCATGATGATATCCAAGGTCCGCTACACCGCCTG

158701CGATAGAAGCGAGTTTGTGAAACTTATCTTTATTTCCTCGTGCCAAATCAAGAGCTCGAG

158761CAATAACAAATCGTTGAACTGGCTTAAATCCGTCAATCATATTTGGAATGGCACGATTTT

158821CAACCGTGTACATAGCATAAGCCAATGCTTCATTATCAATGATACTTTTTAAATCACGAT

158881TATTCAGTTGCATAAATTTACCATACTAGTGAATGTAGTGCCATAATAACATCAGAAATG

158941AAAAGCACGACTTGAATTAATCCGAACATTATTCCGTAATATAATGCTATCAATAAAATA

159001GCAAGGGCTAATGAATAGCCCAAGATTTTCTTAATCATTAGATAACAACACAAATGTTAA

159061ATATGCACACATACCCTGAGCTAAAGCTTGCGAAAACACACTGCTGACATCAATACAGAT

159121AGTTAAAACACATGCTACTATCCAACAAATAAATGAAATAACTCCTAATAATTTTGCGAT

159181ATTCATATTTTCCTCACTGGCGTCCGAAGACGCCTTTAGTTTTAAGATTATTACGATAGA

159241ACTGCATCACGTGTTCGTTGTGGAAATTACTCATTAATATGCCTGCAAAACGAATTTAAA

159301ATTATCAGCCAACATACGGTTCATTTCTTCAAGTGTTTGATACTCAGAATGATGATTACG

159361AGTAAACGCTAAAGCTAACTGTCCTTTTCCAAATCCCGTCGTCAGAGGTTTCATCTTAGA

159421AGCAGGCAGATAAAATACTGCGTATGGAACATTGTTATTTGCAATAGTACGTGCAAGCTG

159481AGACCGACGTTGACGAATATGACTTAGAACCGCACTAAATCCTTGCTTAGAACGCTGATT

159541ACCTACATAAAATCGTGCAGACACACATGGATTACTAAATGGACGACCATCTAATTTACT

159601TACTAAAAAATAAAATCCAGGTTTAGATAAAATATCTTTATGCGGAGTTCCCAAAAACCA

159661CTCACCACCCTTGATTGTACCAATAACAGTAGCGCCTGCATTATTCAGATCAGTAACAGT

159721CATGTATTTCATATTAATTTCCTCTAAATTATTTTCTACTCCAAGGCCGCATGAATACAC

159781GCGGCCATTAAATTAATCGTCGCAGTCGACGCTCAATTCCCAAAACTCTTCTACGGTATA

159841AGTTTCAGTATCATTTTCAATACAGAAACGTTCATTACTGTTATTTGCTAAAGTAGCATT

159901AACTGTCATTTTTTCGCTAGTGCTCTTAAGAGGTGAAATACGAATTAACTGATCACCGTT

159961ATCTAAACAAAAAATTTCACCAACTTTTACATCTTTAAAACTTTTCATAATTCACCTCAA

160021GGAGTATAAAATCCAAACGCAGTTGTTGACCATCCCATCCAATATGGAAAATTTGCACCA

160081ATGTAAAACATAAGAATATAAAACCAACCGCTCAGCAAATTCATTATTTTACACCATTCC

160141AAATTGTTTCAACCACGGATTTTAAACCATTTTGATGAATATCAATTCCGACTACTGTCA

160201TCAAATAGATTCCAACTACAACTGAACCTAAGGCAAAAATCAGCATGAAAATGAATAAAG

160261CCGGAAAAATATTATCGAAAAACCATTCAATAAATGTAAAAGCACTGCGTTTACGCTTCA

160321TATTTTCCTCACATAAATCCAAAGTAAGCGTTTAATACATCAATCATTAAAACGATTGGG

160381AATATACTCAAAACTACTAGTATTATAACTACATTCCATATAGCTTTAACAATCTTTTTC

160441ATTTTCTGTTCCTCCGTAGTTGATAGTTGTATAGTACCACAGAGGAACGGTCTTGTAAAC

160501AACTTTTTTAAAAATATTCGTAATAAATGTGAATACCAACTACTACCGCTGAAACCTGTG

160561CAACCCACCACGCACAAGCAATAAGTACAGAATTCAAAATTTTCATAATAACCTCATTAC

160621AAAAGTAAATGTTAAACAAATTACTGGAATACTAATTAACCAAACAAAACACCAACATAA

160681TGAACTCATAGTTCAATCTCAACGATTTCCAAGTGTTTTTGTTTTATTGCCTTTTTAACA

160741CAATCATGGACTAAATATGACCATGAATCATTATATTCTCCATTAACTAATCGTTGGAGA

160801TCTTCACGTGATGATAAGTAAGGTTGATCTGATTCCCATTTTGATAAAAATACTGCAGTA

160861AAAATATCTCCTTCATCTGTAGTTGAAGTCGTAAATGCAATAAATTCACCGGTAATTTTA

160921TGTTTAGCGTAATAAAATTTAAATTTCATTTTTACTCCTCCGTAGCTGATAGTTGTATAG

160981TACCACGGTCCTTGTGGTATGTAAACTGTTTTGTGAAAATTTTTTAAATGGAAAGATACC

161041ATCCATTGTAGTTGCTTTTTCTTACAACCTTACGAAGGTCTTCTCTGTCACCGATGAACT

161101TCGGAGTGTACTGGATAACACCTGGATGAATTTCTTTAGTGTTGAATATAATTATACAGT

161161CAGCGACTTGATGATTTAGAATGGGCCCTAGATTTATTCCAGAACCGTATGGATACTCTC

161221CGCTGCATCCTGTTGTTACAGAAATCCAACGTGAGCCAGTTTGATGTGTCTTAACTTCTA

161281CACGAAGCCCACAGTATTTTGGATGTGCTAAAACATCCCACGCGTATGTATATGGATCAT

161341CGACATCTTCTTGACCTTTATTAACATACCCGCTTAGCCAATCTGCTACAAAAAACTCTG

161401CGTATACAGCAATACGACATCTTTCAATAACTTCTGCTTTATCCTGGTTCGGGTTTTGTT

161461TTAAAGAGTATCTTGCAGTATCAGCAATTTTGACCTTCATTTCGCTAGTCAAGTCACTGT

161521TCGATAGGGTAAATGTCGGAATCTGAAATAGTCTCTGTAAACCCGGATTCGTTTTCTGCA

161581TTTAGACTTTCCTTTTTACCACTGAGATAAGCGTTATATACTTTAAGAGTGCCGTAATAA

161641ATTCGGTCATTTTCATCTAAAGACTCGCGGTCAAGTTCATCGAGCTCCTTTTTATCCATG

161701ACTATAACATCATTACAGTAAAGAAACCCAAGTTTTTCGTGACCAAACTCAAATTTATTA

161761CAGTACACAATATTAGCGTGATGATTTCCGTGCAGTTTAATCTCTTTTATGTCGGAATCA

161821CCGATATTCATACAATAAATCATAATTTTTCCTTAAAACAAAAGGGCCGAAGCCCTTTAT

161881TTTACTTGAATTGTGCAATTCTTTTCTCTAGACATTCAGCATAAGATTTCATTGAGATGA

161941ACTGTGAAAGTAGCAGTTCTTGCTCAACTACACTAACTGTTAGAAACTTTGCGCTTTCTA

162001AAAATTTACTCAGTGCATTAATTTTGAGCATTAATTGATCGTATTCTTCTTTTACTCGTG

162061CTTGATAACCTAACATAATTTTCCTTAGTTAAGGGCCGAAGCCCTTATTTAAATTGTTCA

162121GTAACGTCTTCAACTACTTCGTATTGGCAGGTACGCATTTTAGCGTCGTTGTAATCAATC

162181GGAATTGATACCACATCGCGAGGATGCACTTTAACTTTTACGACTCGGCTGGTTGAACTG

162241CCAAAGTGACGAATATAAGATTTAGAACACACATGCAGACCACGAGAACAAGTTTGTGTA

162301TCATCGTCATTCACGCGAGTACGTGGCATTTTAACTACTTTACCCGGACTGTTATCAAAG

162361GTATTTGAGTGACAGTCAAAGTAATTGCTGCGAACTACTTTCCAAGCATAGAAGTAGCCA

162421TCTTCTGTAATTTCAATATCGTTTGCTACCAAGAAATCAAAGAGTCGAGATACCGCTTTT

162481TGGCTTGGGTTTTCCAACAGATTTTCCAAGAACGGAAAATAAAATTCAAAGTTTTCGCCT

162541TTTTCCATCGAGTCAAGAATACGATCAACCAAACCAGACCGCAATTCAATATTTTGATAG

162601AACAAGCTTCCACCTTCAATTCGAACATCGCCGGAAATATATTTTTCAACAGCGCGACGA

162661ACATTAATTTTTTGTGCCGCTTCTTCCAACTTATCCGCTACAAGCAGATTAAGAATTTCC

162721TGGAAGTTTGAATGAGTATTAGGAGTTGCGTTATAAGTTACACCGTCAACAGTAATTGAA

162781ATGAATTTTTTAGATGCATTCCAAATAATGTCAGATTTAGCAACTGGAGCAATAACTGCA

162841TCGCTATTAACTTTAACTGTAATATCACCACTAATAGTAACTTTAGGGCGTTTAGCTTCT

162901TCGGCATTTTTCAAAACACGACGGATTGTGTCAACCGATACACCTTGCCAATCAGCCAAT

162961TCCTGTTGGGTATAATTACCGCTTGAATACAGTTTAACAATTTCAGCTTGTTCGTTTTTG

163021GTCAGGCATTTAATATTGTACATAATTTTCCTTATTA

//
